# Bioinspired Cobalt-Catalysis Enables Generation of Nucleophilic Radicals from Oxetanes

**DOI:** 10.1021/acs.orglett.2c00355

**Published:** 2022-03-25

**Authors:** Aleksandra Potrząsaj, Michał Ociepa, Wojciech Chaładaj, Dorota Gryko

**Affiliations:** Institute of Organic Chemistry Polish Academy of Sciences, Kasprzaka 44/52, 01-224 Warsaw, Poland

## Abstract

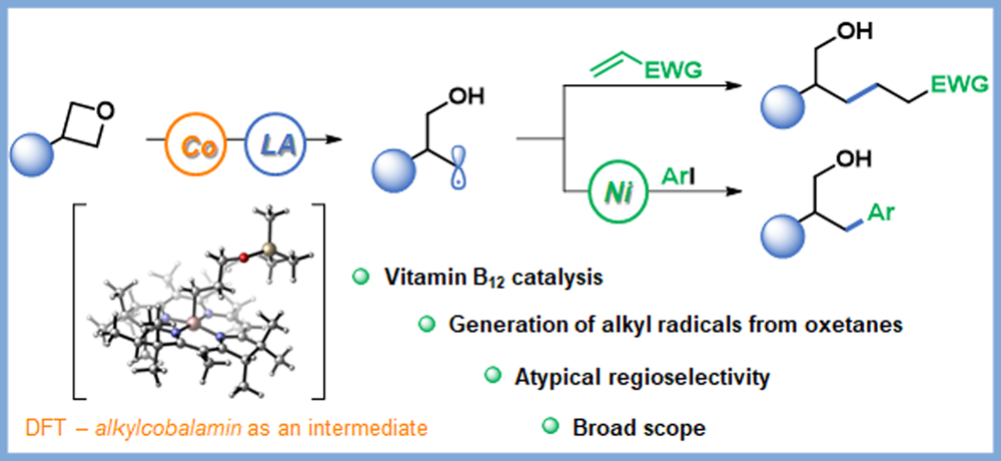

Oxetanes are valuable building blocks due to their well-explored propensity to undergo ring-opening reactions with nucleophiles. However, their application as precursors of radical species is still elusive. Herein, we present a bioinspired cobalt-catalysis-based strategy to access unprecedented modes of radical reactivity via oxetane ring-opening. This powerful approach gives access to nucleophilic radicals that engage in reactions with SOMOphiles and low-valent transition metals. Importantly, the regioselectivity of these processes complements known methodologies.

The oxetane moiety is present in many natural compounds and drug molecules. Due to their position as both carbonyl and *gem*-dimethyl group surrogates, oxetanes are important scaffolds in drug discovery (bioisosteres). They are also valuable as C-3 building blocks for the synthesis of highly functionalized organic frameworks.^1^

The high ring-strain governs the reactivity of oxetanes facilitating a plethora of transformations; among these, strategies based on breaking the C–O or C–C bonds predominate.^2a,2b^ Indeed, the most explored reaction is nucleophilic ring-opening with heteroatom nucleophiles, but there are also a few reports describing their reactions with C-nucleophiles.^1−6^ In addition, oxetanes are a convenient source of α-oxy radicals. The MacMillan group developed an efficient methodology for the deoxygenative arylation of alcohols^7^ and for the α-arylation of ethers, including oxetanes,^8^ while Ravelli et al. demonstrated their photochemical reaction with electron-deficient olefins.^9^ These examples represent radical functionalizations, where the 4-membered ring is preserved.

On the other hand, despite the significant strain energy of the oxetanes (c.a. 106 kJ/mol), their application in radical transformations initiated by opening of strained-ring systems is limited to only few examples. Grimme and Gansäuer developed a Cp_2_TiCl-catalyzed system for the generation of γ-titanoxy radicals.^10^ The authors however concluded that “γ-titanoxy radicals are not suitable for efficient formation of C–C bonds”. Similar reactivity was achieved by the Okamoto group in the presence of low-valent titanium alkoxides.^11^ They also used iron-catalysis to access 3-oxidopropylmagnesium compounds from 2-substituted oxetanes.^12^ Although the above transformation likely proceeds via an γ-oxidoradical intermediate, it is immediately intercepted by an iron catalyst, which thus precludes free-radical reactivity. *Despite the immense importance of these seminal contributions, the possibility to access various modes of radical reactivity via ring-opening of oxetanes remains challenging.*

Recently, we have reported a polarity reversal strategy enabling functionalization of strained cycloalkanes^13^ and regioselective ring-opening of epoxides (oxiranes).^14^ The crucial step of these processes involves the formation of alkyl cobalamins from vitamin B_12_ and electrophilic substrates followed by the homolytic cleavage of the Co–C leading to alkyl radicals. Although the strain energies of oxirane (112 kJ/mol) and oxetane (106 kJ/mol) rings are on a similar level, ring-opening of the latter is kinetically unfavorable due to the high activation energy of this process (Scheme 1A).

**Scheme 1:**
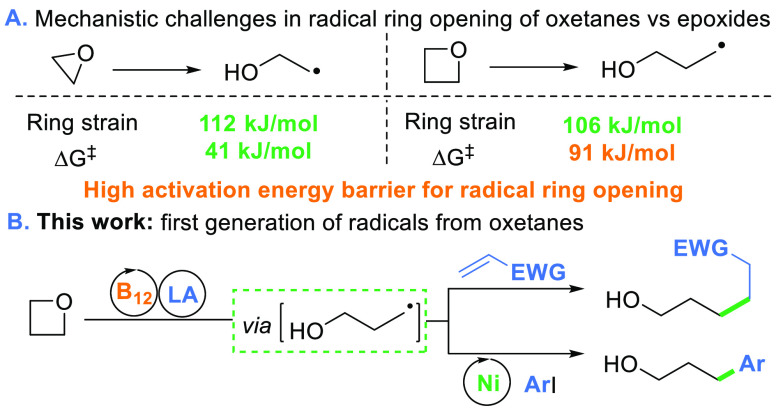
Radical Ring-Opening of Strained Ethers

We envisioned that the merger of cobalt-catalysis with a suitable oxetane’s activation mode should enable the generation of alkyl radicals from oxetanes, by overcoming the challenging kinetics of the ring-opening step (Scheme 1B). Herein, we report a general method that give access to nucleophilic C-centered radicals from oxetanes in the bioinspired vitamin B_12_-catalyzed ring-opening reaction and its application in both Ni-catalyzed cross-electrophile coupling and the Giese-type addition.

Nucleophilic radicals engage in cross-electrophile coupling with aryl halides via cooperative Co/Ni-catalysis.^15−17^ We hypothesize that radicals generated from oxetanes should react in a similar manner. To this end, our experimental investigations began with the conditions developed for epoxides.^14^ The model reaction of oxetane (**1**) with aryl iodide (**2**) did not however lead to desired product **4a** (Scheme 2).

**Scheme 2:**
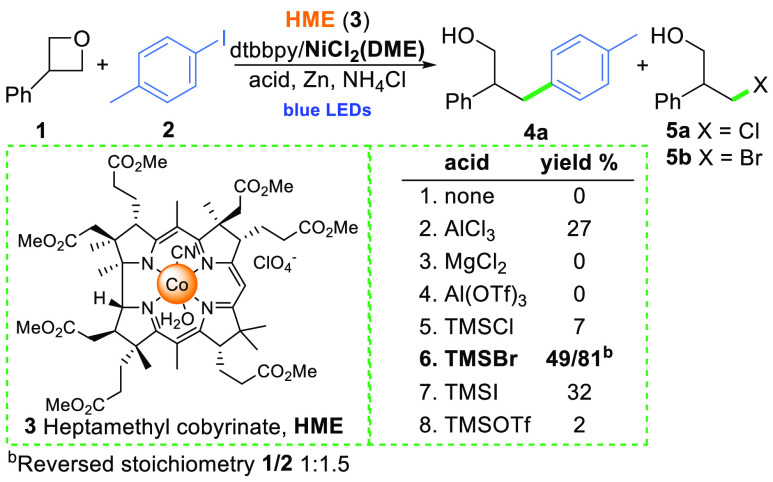
Influence of Acids on the Co/Ni Cross-Electrophile Coupling of Oxetane **1** with Aryl Iodide **2**

DFT calculations revealed that the free Gibbs energy for the ring-opening of oxetanes with the model corrin is substantially higher than the one calculated for the generation of radicals from epoxides (Scheme 3).^18,19^

**Scheme 3:**
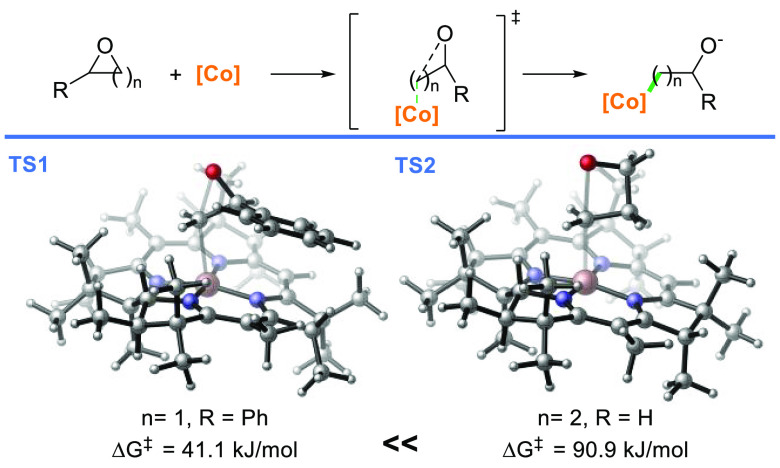
Gibbs Free Energy Barriers for the Opening of Epoxides and Oxetanes with the Co(I)-Corrin complex

Due to the Lewis basicity of the oxygen atom, oxetanes are activated by acids making them susceptible toward nucleophiles.^1,3^ However, the use of these reagents, from the standpoint of our catalytic approach, poses several challenges. Hydrophilic vitamin B_12_ bearing Lewis basic amide groups may undergo side-reactions with acids, hindering its catalytic activity. Moreover, the activated oxonium cation may be prone to reductive cleavage of the C–O bond. Thus, hydrophobic heptamethyl cobyrinate (**3**), a vitamin B_12_ derivative, in combination with various acid additives, was tested in the model reaction (see Scheme 2 and SI). In the presence of Brønsted acids (TFA, *p*TSA), only the reductive ring-opening occurred. In contrast, some Lewis acids promoted the formation of the desired product **4a**, but a notable difference in the reactivity was observed depending on the anion of the salt. DFT calculations suggested that the ring-opening of oxetanes that are activated via coordination of AlCl_3_ is practically barrierless (see SI). Indeed, in the experiment with AlCl_3_, product **4a** formed in 27% yield, and halohydrin **5a** was observed as a side product, while the use of Al(OTf)_3_ led exclusively to the products resulting from the reductive ring-opening. We hypothesized that halohydrin might act as an intermediate. On the basis of further screening of halide-containing Lewis acids, TMSBr proved the most effective activator in promoting the model reaction.

The Zultanski group has recently showed that the use of a Ni/Co dual catalytic system facilitates reaction optimizations as a Ni-catalyst activates aryl halides while Co-catalysis induces the formation of radicals from alkyl halides.^20^ Consequently, generation of reactive intermediates can be tuned separately. Our in-depth optimization studies revealed that various Ni-complexes catalyze the cross-electrophile ring-opening of oxetane **1** with aryl iodide **2**; with NiCl_2_(DME) and dtbbpy the yield increased to an appreciable 84% (see SI). To check how the optimization process leveraged the effect of the cobalt catalyst, the model reaction was performed under the optimized conditions but without HME (**3**). The reaction stopped at the bromohydrin formation step corroborating exclusive generation of alkyl radicals only in the Co-catalytic cycle.

Having identified the optimal reaction conditions, we examined the generality of the developed method (Scheme 4A). Initially, we focused our effort on 3-phenyl-substituted oxetanes of type **1**. Oxetanes with an electron-deficient phenyl ring yielded the product while those with an electron-donating substituent did not. In general, various substituents at the C3 position are well-tolerated. Not only aryl and heteroaryl substituents but also protected hydroxy and amino groups can be present at this position. Alkyl groups, on the other hand, can occupy both the C3 and C2 positions giving the corresponding products (**8**, **10**) in 65% and 76% yields, respectively.

**Scheme 4:**
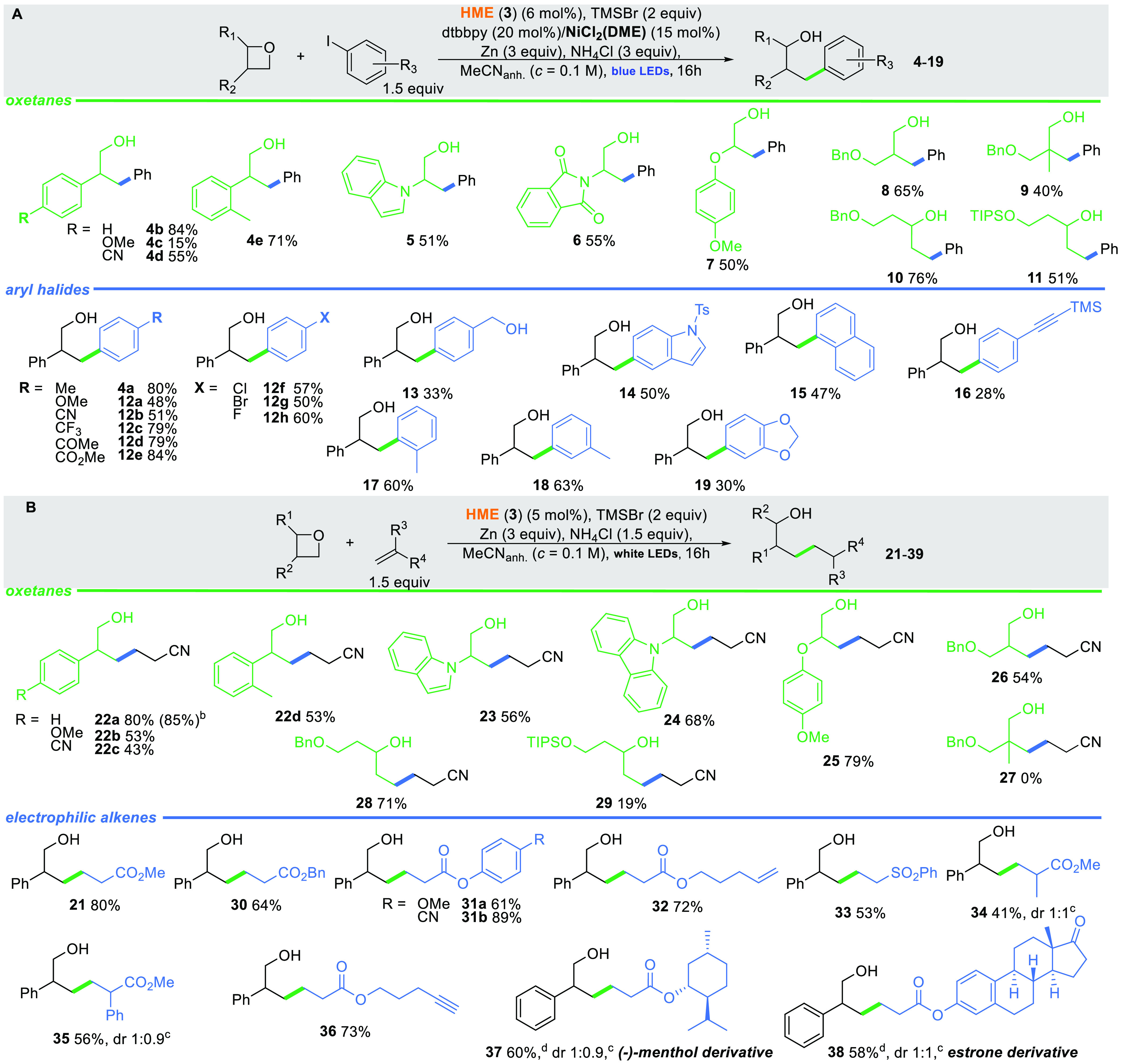
Scope of the Co/Ni-Catalyzed Cross Electrophile of Oxetanes and Aryl Iodides and the Giese Addition of Oxetanes to Electrophilic Alkenes Reaction was performed on 0.1 mmol scale. dr determined by ^13^C NMR. (*c* = 0.03 M.) Reactions were quenched with 2 equiv of citric acid, for **11** K_2_CO_3_ was used.

Regarding the aryl halide, a wide range of phenyl iodides with both electron-withdrawing and electron-donating substituents at the C-4 position are suitable starting materials (alcohols **12**). For more hindered halides, the yield slightly decreased (products **17** and **18**). It is worth mentioning that although vitamin B_12_ is a known catalyst for dehalogenation reactions,^21,22^ for aryl halides bearing Cl and Br this side-reaction was not observed. Electron-rich aryl halides are less reactive in this transformation. It is gratifying that even sensitive 1-iodo-4-(trimethylsilylethynyl)-benzene furnished desired product **16**, which can be further used in the sila-Sonogashira–Hagihara coupling.^23^

Like the described cross-coupling reaction, alkyl radicals generated from oxetanes should also engage in reactions with SOMOphiles. The model reaction of 3-phenyloxetane (**1**) with methyl acrylate (**20**) without any activator did not lead to desired product **21** (Scheme 4B). Not surprisingly, the activation with TMSBr proved also crucial in this case and led to product **21** in 57% yield. Other acids tested were less effective in catalyzing the Giese-type addition (see SI). Further systematic studies on this model reaction identified the optimal conditions under which desired product **21** formed in 80% yield. Irradiation with blue or green light yielded the desired product, though in a diminished 57% and 69% yield, respectively. Other tested cobalt complexes were less effective in catalyzing the model reaction.

The reactivity of oxetanes in the Giese-type addition follows a similar pattern as in the cross-coupling reaction (Scheme 4B). Both EDG and EWG at the 3-phenyl substituent are well-tolerated (for **22b** and **22c**, 53% and 43%, respectively), and the substitution pattern did not significantly affect the reaction yield. Both *N*-oxetanyl indole and carbazole underwent the reaction effectively giving **23** and **24** in 56% and 68% yields. Furthermore, oxetanes bearing a protected hydroxy group are well-tolerated though the protecting group must be chosen carefully, as silyloxy oxetane furnished product **29** in low yield. C2-alkyl-substituted substrates behaved similarly to oxiranes, and the ring-opening occurred at the less hindered site due to the steric hindrance, a feature characteristic for vitamin B_12_-catalyzed reactions.^14^ These results are however in contrast to reports from Grimme.^10^ Both Cp_2_TiCl_2_-catalyzed reactions predominantly lead to primary alcohols. Thus, our Co-based methodology complements the existing approaches.

A large array of electron-deficient olefins are well-tolerated for the reaction (Scheme 4B). Acrylates provide products in good to excellent yields (**21**, **30–32**, **36**). Esters **32** and **36**, containing a terminal double and triple bond, respectively, are worth mentioning as no reduction was observed at these ends. 1,2-Disubstituted olefins furnish products, though in very low yield. However, the presence of a substituent at the α-position to the ester group does not have a negative impact on the reaction (**34**, **35**). The utility of the developed method was realized when applying it to complex molecules such as an estrone derivative. Due to an issue with solubility, this reaction was performed at a lower concentration, and as a result, the yield of product **38** significantly increased from 28% to 58%.

To gain a better understanding of the developed transformations, mechanistic experiments were performed (see SI). First, control experiments revealed that the cobalt complex, Zn, and light are all crucial for the developed reaction to occur. Second, the radical nature of this process was supported by the complete shutdown of the reaction in the presence of a radical trap (Figure 1A). Third, TMSBr reacts swiftly with oxetanes, delivering bromohydrin within 10 min. It has been already reported that the addition of TMSBr to oxetanes gives silylated bromohydrins.^24^ Thus, although direct reaction of Co(I) with oxetane activated by Lewis acid seems rational and cannot be unambiguously ruled out, the dominant pathway involves TMSBr-mediated formation of bromohydrin, followed by its reaction with low-valent cobalt species. To examine the intermediacy of the bromohydrin, compound **5b** was subjected to the reaction conditions. Product **22a** formed in 77% yield, suggesting that bromohydrin is indeed involved in the catalytic cycle. The kinetic experiments clearly show that the oxetane is fully converted into bromohydrin within the first few minutes, while the product gradually forms over 10 h (Figure 1B). In addition, the MS analysis also shows the peak corresponding to the alkyl-cobalt complex. Furthermore, the model reaction performed with deuterated reagents (ND_4_Cl) showed deuterium-incorporation only at the α-position to the electron-withdrawing group originating from the olefin. This indicates the formation of an anion at this position.

**Figure 1:**
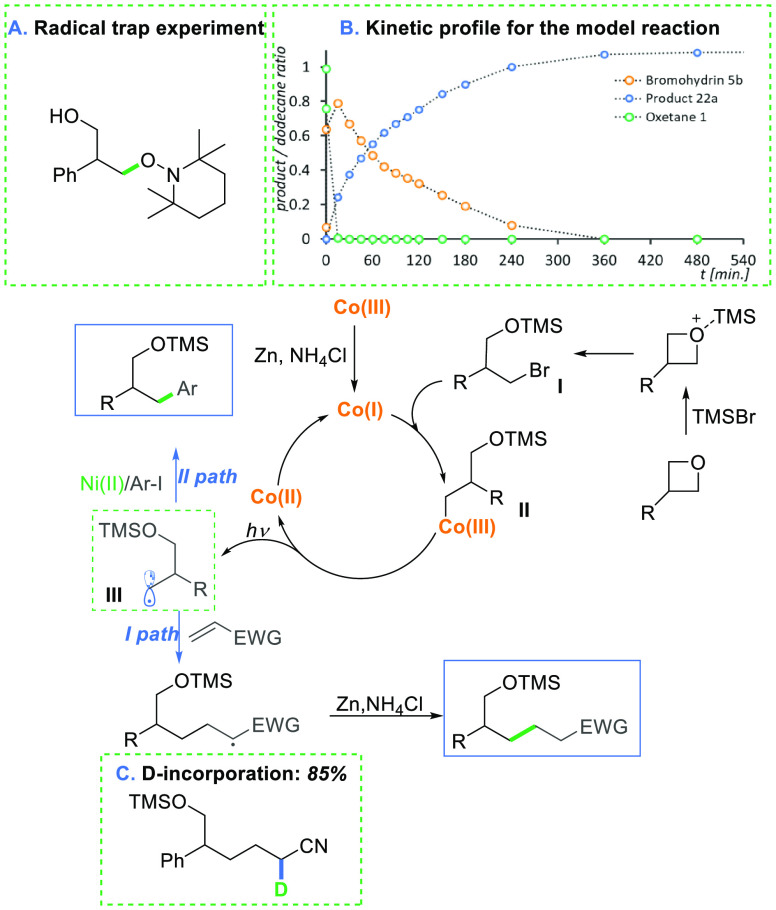
Proposed a mechanism: I path, Giese-type addition; II path, Co/Ni cross coupling.

On the basis of our mechanistic considerations and DFT calculations (see SI), we proposed the key steps in the developed transformations (Figure 1). First, oxetane undergoes exergonic ring-opening with TMSBr (Δ*G* = −51.4 kJ/mol). The resulting silyl ether of γ-bromohydrin enters a facile reaction with the nucleophilic Co(I) complex through a S_N_2 manifold^25,26^ (Δ*G*^⧧^ = 29.4 kJ/mol), giving rise to a Co(III)-alkyl intermediate **II**, featuring a relatively weak Co–C(sp^3^). This species is photoactive in the visible region, which can trigger a homolytic cleavage of the Co–C bond providing alkyl radical **III** and the Co(II) complex. As proposed by Kozlowski, photodissociation proceeds presumably from the first electronically excited state (S1) through the generation of a singlet radical pair. The generated radical **III** either is trapped by an electron-deficient olefin or enters the Ni-catalytic cycle.

Herein, we described the vitamin B_12_ assisted generation of C-centered alkyl radicals from oxetanes via an alkylated cobalt complex. Subsequent, light-induced homolysis of the Co–C bond yields radical species that engage in reactions with SOMOphiles and low-valent transition metal complexes. Thus, this useful C3 synthon can now be employed in various radical reactions such as Giese addition and cross-electrophile coupling. Both reactions tolerate a broad range of starting materials with different functional groups. The unique regioselectivity of the developed reaction complements the existing strategies; here, the less substituted C2 carbon atom is functionalized. These examples suggest that bioinspired Co-catalysis will enable other radical transformations of oxetanes to be developed.

Ultimately, we believe that the reported bioinspired activation mode for the generation of C-centered radicals opens new opportunities in radical chemistry of oxetanes and will enable broader application of this valuable C3 synthon in the construction of complex molecules.

## Supporting Information Available

Experimental details and procedures, optimization studies, mechanistic experiments, and spectral data for all new compounds (PDF)
