# Supplementary material for: Bioinspired Cobalt-Catalysis Enables Generation of Nucleophilic Radicals from Oxetanes
Source: Org Lett. 2022 Mar 25;24(13):2469–73. doi: 10.1021/acs.orglett.2c00355 (PMC9003576; doi:10.1021/acs.orglett.2c00355)

Supporting Information

Bioinspired Co-Catalysis Enables Generation of Nucleophilic Radicals from Oxetanes

Aleksandra Potrząsaj‡, Michał Ociepa‡, Wojciech Chaładaj*, Dorota Gryko*

Institute of Organic Chemistry Polish Academy of Sciences
Kasprzaka 44/52, 01-224 Warsaw, Poland

e-mail: dorota.gryko@icho.edu.pl

Table of contents

1. General Information 5

2. Setup for photoreactions 6

3. Full Optimization of the Co/Ni – catalyzed cross-electrophile coupling Parameters 7

3.1 Background experiments 7

3.2 Optimization of the substrates ratio 7

3.3 Optimization of a solvent for HME (3) – catalyzed reaction 8

3.4 Concentration of oxetane (1) 8

3.5 Screening of Lewis acid 9

3.6 Screening of the cobalt catalysts 10

3.7 HME (3) – catalyst loading 10

3.8 Screening of Ni – catalysts 11

3.9 Screening of ligands 11

3.10 The influence of Zn and NH_4Cl amounts 12

4. Full Optimization of Giese-type addition Parameters 13

4.1 Background experiments 14

4.2 Screening of Lewis acid 14

4.3 The influence of light on the model reaction 14

4.4 Optimization of the substrates ratio 15

4.5 Screening of the cobalt catalyst 15

4.6 HME (3) – catalyst loading 16

4.7 Optimization of the solvent for HME (3) catalyzed reaction 16

4.8 Concentration of oxetane (1) 16

4.9 The influence of Zn and NH_4Cl amounts 16

4.10 The influence of an amount of Lewis acid 17

5. General Procedures 18

A. General procedure for the Co/Ni-catalyzed cross-electrophile coupling 18

B. General procedure for the Giese-type reaction 18

5.1 Note 18

5.2 Graphical procedure for the Co/Ni cross-electrophile coupling 19

5.3 Graphical procedure for the Giese-type addition 20

6. Scope and characterization of new compounds 21

6.1 Substrates 21

6.2 Cross-electrophile coupling: oxetanes ... 22
6.3 Cross-electrophile coupling: aryl halides ... 26
6.4 Giese-type addition: oxetanes ... 32
6.5 Giese-type addition: Michael acceptors ... 35
7. Mechanistic consideration ... 40
7.1 Proposed mechanism ... 40
7.2 Mass spectrometry studies ... 40
7.3 Kinetic studies for the model reaction ... 41
7.4 Reactions with deuterated reagents ... 42
7.5 Experiment with a radical trap ... 43
7.6 DFT calculations ... 43
8. References ... 64
9. NMR spectra ... 67
triisopropyl(2-(oxetan-2-yl)ethoxy)silane (S14) ... 67
3-bromo-2-phenyl-propan-1-ol (**5b**) ... 68
2,3-diphenylpropan-1-ol (**4b**) ... 69
4-(1-hydroxy-3-phenylpropan-2-yl)benzonitrile (**4d**) ... 70
3-phenyl-2-(*o*-tolyl)propan-1-ol (**4e**) ... 71
2-(1*H*-indol-1-yl)-3-phenylpropan-1-ol (**5**) ... 72
2-(1-hydroxy-3-phenylpropan-2-yl)isoindoline-1,3-dione (**6**) ... 73
2-(4-methoxyphenoxy)-3-phenylpropan-1ol (**7**) ... 74
2-benzyl-3-(benzyloxy)propan-1-ol (**8**) ... 75
2-benzyl-3-(benzyloxy)-2-methylpropan-1-ol (**9**) ... 76
1-(benzyloxy)-5-phenylpentan-3-ol (**10**) ... 77
1-phenyl-5-((triisopropylsilyl)oxy)pentan-3-ol (**11**) ... 78
2-phenyl-3-(4-methylphenyl)propan-1-ol (**4a**) ... 79
3-(4-methoxyphenyl)-2-phenylpropan-1-ol (**12a**) ... 80
4-(3-hydroxy-2-phenylpropyl)benzonitrile (**12b**) ... 81
2-phenyl-3-(4-(trifluoromethyl)phenyl)propan-1-ol (**12c**) ... 82
1-(4-(3-hydroxy-2-phenylpropyl)phenyl)ethanone (**12d**) ... 84
methyl 4-(3-hydroxy-2-phenylpropyl)benzoate (**12e**) ... 85
3-(4-chlorophenyl)-2-phenylpropan-1-ol (**12f**) ... 86
3-(4-bromophenyl)-2-phenylpropan-1-ol (**12g**) ... 87
3-(4-fluorophenyl)-2-phenylpropan-1-ol (**12h**) ... 88

3-(4-(hydroxymethyl)phenyl)-2-phenylpropan-1-ol (**13**) 90
2-phenyl-3-(1-tosyl-1*H*-indol-5-yl)propan-1-ol (**14**) 91
3-(naphthalen-1-yl)-2-phenylpropan-1-ol (**15**) 92
2-phenyl-3-(4-((trimethylsilyl)ethynyl)phenyl)propan-1-ol (**16**) 93
2-phenyl-3-(2-methylphenyl)propan-1-ol (**17**) 94
2-phenyl-3-(3-methylphenyl)propan-1-ol (**18**) 95
3-(benzo[*d*][1,3]dioxol-5-yl)-2-phenylpropan-1-ol (**19**) 96
6-hydroxy-5-phenylhexanenitrile (**22a**) 97
6-hydroxy-5-(4-methoxyphenyl)hexanenitrile (**22b**) 98
4-(5-cyano-1-hydroxypentan-2-yl)benzonitrile (**22c**) 99
6-hydroxy-5-(2-methylphenyl)hexanenitrile (**22d**) 100
6-hydroxy-5-(1*H*-indol-1-yl)hexanenitrile (**23**) 101
5-(9*H*-carbazol-9-yl)-6-hydroxyhexanenitrile (**24**) 102
6-hydroxy-5-(4-methoxyphenoxy)hexanenitrile (**25**) 103
6-(benzyloxy)-5-(hydroxymethyl)hexanenitrile (**26**) 104
8-(benzyloxy)-6-hydroxyoctanenitrile (**28**) 105
6-hydroxy-8-((triisopropylsilyl)oxy)octanenitrile (**29**) 106
methyl 6-hydroxy-5-phenylhexanoate (**21**) 107
benzyl 6-hydroxy-5-phenylhexanoate (**30**) 108
4-methoxyphenyl 6-hydroxy-5-phenylhexanoate (**31a**) 109
4-cyanophenyl 6-hydroxy-5-phenylhexanoate (**31b**) 110
pent-4-en-1-yl 6-hydroxy-5-phenylhexanoate (**32**) 111
2-phenyl-5-(phenylsulfonyl)pentan-1-ol (**33**) 112
methyl 6-hydroxy-2-methyl-5-phenylhexanoate (**34**) 113
methyl 6-hydroxy-2,5-diphenylhexanoate (**35**) 114
pent-4-yn-1-yl 6-hydroxy-5-phenylhexanoate (**36**) 115
(1*R*,2*S*,5*R*)-2-isopropyl-5-methylcyclohexyl 6-hydroxy-5-phenylhexanoate (**37**) 116
(8*R*,9*S*,13*S*,14*S*)-13-methyl-17-oxo-7,8,9,11,12,13,14,15,16,17-decahydro-6*H*-cyclopenta[a]phenanthren-3-yl 6-hydroxy-5-phenylhexanoate (**38**) 117

1. General Information

General Procedures. Unless otherwise noted, reactions were performed without the exclusion of air or moisture. All the photochemical reactions were performed in 10 mL glass vials sealed with a rubber septum. Reactions were monitored by gas chromatography (GC, specification below) or thin-layer chromatography (TLC) on Merck silica gel (GF254, 0.20 mm thickness), visualizing with UV-light, potassium permanganate ($KMnO_4$), or ceric ammonium molybdate (CAM)/Hanessian's stain. Colum chromatography was performed using Merck silica gel 60 (230-400 mesh). GC yields were using dodecane as an internal standard.

Materials. Commercial reagents and solvents were purchased from Sigma-Aldrich, Acros Organics, Alfa Aesar, Fluorochem, and TCI, and used as received unless otherwise noted. Dry solvents: dimethyl sulfoxide (DMSO), dichloromethane (CH_2Cl_2), tetrahydrofuran (THF), acetonitrile (CH_3CN) were taken from the *Solvent Purification System* (SPS). Deuterated solvents ($CDCl_3$ and CD_3CN) were purchased from Eurisotop. Substrates: 3-phenyloxetane (**1**)[1], 3-(4-methoxyphenyl)oxetane[2], 4-(oxetan-3-yl)benzonitrile[1], 3-(2-methylphenyl)oxetane[2], 1-(oxetan-3-yl)-1H-indole[3], 9-(oxetan-3-yl)-9H-carbazole[3], 3-(4-methoxyphenoxy)oxetane[3], 3-((benzyloxy)methyl)oxetane[1], 2-(2-(benzyloxy)ethyl)oxetane[4], 2-(oxetan-3-yl)isoindoline-1,3-dione[1], 3-methyl-3-(3-phenyl-2-oxapropyl)oxetane[1], 3-methyl-3-(3-phenyl-2-oxapropyl)oxetane[1] and catalysts: $NiCl_2$(dtbbpy)[5], (CN)(H_2O)Cby$(OMe)_7$ (**3**)[6], were synthesized according to literature procedures.

Before the use, zinc was activated by the following method: a) washing with 10% HCl, b) grinding, c) washing with H_2O, EtOH, and Et_2O, d) drying in a vacuum.[6]

Instrumentation.

- NMR Spectroscopy: ^{1}H and ^{13}C NMR spectra were recorded at 25 °C on a Bruker 400 MHz, 500 MHz or Varian 600 MHz instrument with TMS as an internal standard. NMR chemical shifts are reported in ppm and referenced to the residual solvent peak of $CDCl_3$ (7.26 ppm - ^{1}H NMR and 77.16 ppm - ^{13}C NMR). Multiplicities are indicated by singlet (s), doublet (d), triplet (t), quartet (q), multiplet (m) and broad (br). Coupling constants (*J*) are reported in Hertz. All data analysis was performed using MestReNova software package.

- GC/MS Chromatography: GC-MS analyses were performed using Shimadzu GCMS-QP2010 SE gas chromatograph with FID detector and Zebron ZB 5MSi column.

- High Resolution Mass Spectrometry: High-resolution mass spectra (HRMS) were recorded on a Waters AutoSpec Premier instrument using electron ionization (EI) or a Waters SYNAPT G2-S HDMS instrument using electrospray ionization (ESI) with time-of-flight detector (TOF).

- Low Resolution Mass Spectrometry: Low-resolution mass spectra (LRMS) were recorded on an Applied Biosystems API 365 mass spectrometer using electrospray ionization (ESI) technique.

- Melting points: Melting points were recorded on a Marienfeld MPM-H2 melting point apparatus and are uncorrected.

- Preparative HPLC: Preparative HPLC separations were performed using Knauer HPLC chromatograph with PDA detector and Preparative column chromatography Knauer EII 100-10 Si column (250 x 20 mm).

2. Setup for photoreactions

Reactions were performed in a homemade photoreactor comprised of a 400 mL beaker with the inside covered with LED tape (Figure S1). A cooling fan with adjustable spin rate was used to maintain ambient temperature inside the photoreactor.

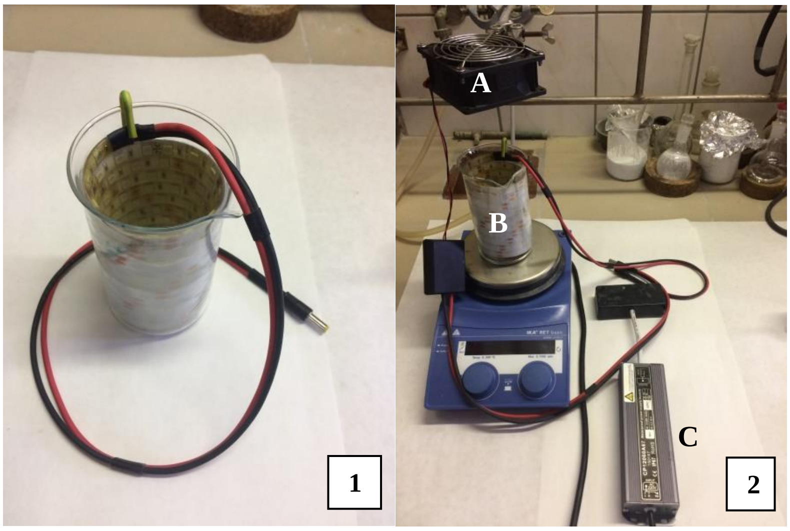

Figure S1 (**1**) The photoreactor; (**2**) Assembled photoreaction setup: **A** – cooling fan, **B** – photoreactor, **C** – 12 V power supply module for photoreactor and fan.

LED tapes characteristics:

Blue LED tape: 8 mm SMD3528 LED strip, 60 LED diodes/m

Power consumption: 4.8 W/m

Blue light: λ_{max} = 460 nm, 4.5 lm

Green LED tape: 10 mm SMD5050 LED strip, 60 LED diodes/m

Power consumption: 14.4 W/m

Green light: λ_{max} = 525 nm, 20 lm

White LED tape: 8 mm SMD3528 LED strip, 120 LED diodes/m

Power consumption: 9.6 W/m

White light: 6500 K, 30 lm

3. Full Optimization of the Co/Ni – catalyzed cross-electrophile coupling Parameters:

Model reaction:

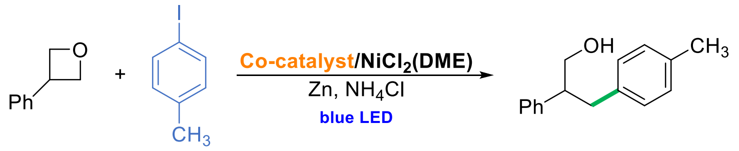

Reaction conditions: oxetane (**1**) (0.2 mmol, 1 equiv), 4-iodotoluene (**2**) (1.5 equiv), Zn (3 equiv), NH_4Cl (3 equiv), HME (**3**) (6 mol%), $NiCl_2(DME)$ (15 mol%), dtbby (20 mol%), TMSBr (2 equiv), $MeCN_{anh.}$ (c = 0.1 M), blue LEDs, 16 h; each reaction was quenched by treatment with 2 equiv of citric acid.

Table S1 Background experiments[a]

Entry	Deviation from the Initial Conditions	Yield of **4a** [%]
**1**	**none**	**29/26**[b]
2	no Co-catalyst	5
3	no Ni-catalyst	Product not observed
4	no reducing agent	Product not observed
5	no Lewis acid	Product not observed
6	no light	3
7	MeCN („wet”)	Product not observed
8	Air atmosphere	17

[a]**Reaction conditions:** oxetane (**1**) (0.1 mmol, 1 equiv), 4-iodotoluene (**2**) (1.5 equiv), Zn (3 equiv), NH_4Cl (3 equiv), HME (**3**) (5 mol%), $NiCl_2(DME)$ (20 mol%), dtbby (40 mol%), TMSBr (2 equiv), $MeCN_{anh}$ (c = 0.1 M), blue LEDs, yields determined by GC, 16 h, [b]Isolated yield.

Table S2 Optimization of the substrates ratio[a]

Entry	Oxetane **1** (equiv)	Aryl iodide **2** (equiv)	Yield of **4a** [%]
1	1	1.5	29/26[b]
2	1	3	17
**3**	**1.5**	**1**	**27**
4	3	1	21

[a]**Reaction conditions:** oxetane (**1**), 4-iodotoluene (**2**), Zn (3 equiv), NH_4Cl (3 equiv), HME (**3**) (5 mol%), $NiCl_2(DME)$ (20 mol%), dtbby (40 mol%), TMSBr (2 equiv), $MeCN_{anh}$ (c = 0.1 M), blue LEDs, 16 h, yields determined by GC, [b]Isolated yield.

Table S3 Optimization of a solvent for HME (3) – catalyzed reaction[a]

Entry	Solvent	Yield of **4a** [%]
**1**	**MeCN**	**27**
2	MeOH	0
3	DMF	0
4	DMA	3
5	NMP	0
6	THF	7
7	Ethyl acetate	0
8	Acetone	4
9	DME	16

[a]**Reaction conditions:** oxetane (**1**) (1.5 equiv), 4-iodotoluene (**2**) (0.1 mmol, 1.0 equiv), Zn (3 equiv), NH_4Cl (3 equiv), HME (**3**) (5 mol%), $NiCl_2$(DME) (20 mol%), dtbby (40 mol%), TMSBr (2 equiv), solvent (c = 0.1 M), blue LEDs, 16 h, yields determined by GC.

Table S4 Concentration of oxetane (1)[a]

Entry	[mol/dm^3]	Yield of **4a** [%]
1	0.05	8
**2**	**0.1**	**27**
3	0.2	23

[a]**Reaction conditions:** oxetane (**1**) (1.5 equiv), 4-iodotoluene (**2**) (0.1 mmol, 1.0 equiv), Zn (3 equiv), NH_4Cl (3 equiv), HME (**3**) (5 mol%), $NiCl_2$(DME) (20 mol%), dtbby (40 mol%), TMSBr (2 equiv), $MeCN_{anh}$, blue LEDs, 16 h, yields determined by GC.

Table S5 Screening of Lewis acid[a]

Entry	Lewis acid	Yield of **4a** [%]
1	$AlCl_3$	27
2	$LiBF_4$	0
3	$MgCl_2$	0
4	$In(OTf)_3$	0
5	$Al(OTf)_3$	0
6	$Fe(OTf)_2$	0
7	$Ga(OTf)_3$	0
8	$SnCl_4$	0
9	InI_3	0
10	TMSCl	7
11	$(ETO)_3SiCl$	0
12	TMSBr	49
13	TMSI	32
14	TMSOTf	2
**15[b]**	**TMSBr**	**81/78[c]**

[a]**Reaction conditions:** oxetane (**1**) (1.5 equiv), 4-iodotoluene (**2**) (0.1 mmol, 1 equiv), Zn (3 equiv), NH_4Cl (3 equiv), HME (**3**) (5 mol%), $NiCl_2$(DME) (20 mol%), dtbby (40 mol%), Lewis acid (2 equiv), $MeCN_{anh}$ (c = 0.1 M), blue LEDs, 16 h.; [b]Reversed stoichiometry: **1**/**2** 1:1.5; yields determined by GC, [c]Isolated yield.

Table S6 Screening of the cobalt catalysts[a]

Entry	Co-catalyst	Yield of **4a** [%]
**1**	**3**	**81**
2	S1	50
3	S2	29
4	S3	0

[a]**Reaction conditions:** oxetane (**1**) (0.1 mmol, 1 equiv), 4-iodotoluene (**2**) (1.5 equiv), Zn (3 equiv), NH_4Cl (3 equiv), Co-catalyst (5 mol%), $NiCl_2$(DME) (20 mol%), dtbby (40 mol%), TMSBr (2 equiv), $MeCN_{anh}$ (c = 0.1 M), blue LEDs, 16 h, yields determined by GC.

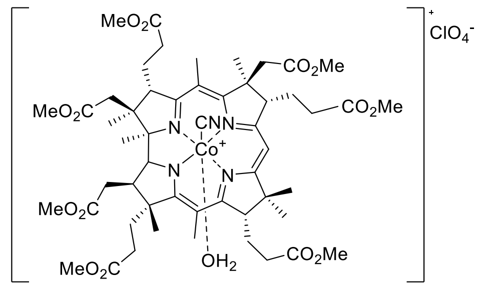

Aqua(cyano)heptamethyl cobyrinate (3)

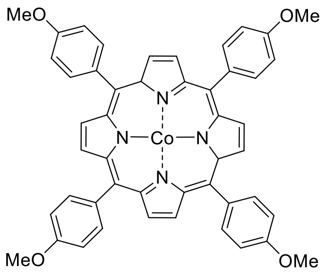

5,10,15,20-Tetrakis(4-methoxyphenyl)-21*H*,23*H*-porphine cobalt (II) (S1)

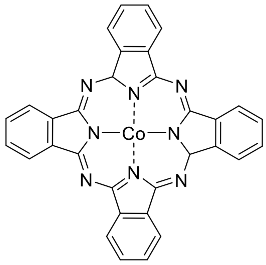

Cobalt(II) phthalocyanine (S2)

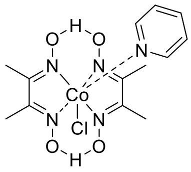

Chloro(pyridine)bis (dimethylglyoximato)cobalt(III) (S3)

Table S7 HME (3) – catalyst loading[a]

Entry	Catalyst loading [%]	Yield of **4a** [%]
1	2.5	32
**2**	**5**	**81**
3	7.5	25

[a]**Reaction conditions:** oxetane (**1**) (0.1 mmol, 1 equiv), 4-iodotoluene (**2**) (1.5 equiv), Zn (3 equiv), NH_4Cl (3 equiv), HME (**3**), $NiCl_2$(DME) (20 mol%), dtbby (40 mol%), TMSBr (2 equiv), $MeCN_{anh}$ (c = 0.1 M), blue LEDs, 16 h, yields determined by GC.

Table S8 Screening of Ni – catalysts[a]

Entry	Catalyst	Catalyst loading [mol%]	Yield of **4a** [%]
1	$NiCl_2$(DME)	20	81/78[b]
2	$NiBr_2$(DME)	20	66
3	$NiBr_2$	20	77
4	NiI_2	20	56
5	$Ni(OTf)_2$	20	76
6	$NiCl_2$(dtbbpy)	20	43
**7**	**$NiCl_2$(DME)**	**15**	**82/80[b]**
8	$NiCl_2$(DME)	10	78
9	$NiCl_2$(DME)	5	66

[a]**Reaction conditions:** oxetane (**1**) (0.1 mmol, 1 equiv), 4-iodotoluene (**2**) (1.5 equiv), Zn (3 equiv), NH_4Cl (3 equiv), HME (**3**) (5 mol%), Ni-catalyst, dtbby (40 mol%), TMSBr (2 equiv), $MeCN_{anh}$ (c = 0.1 M), blue LEDs, 16h, yield was determined by GC; [b]Isolated yield.

Screening of ligands[a]

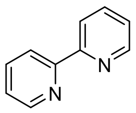

S4 65%

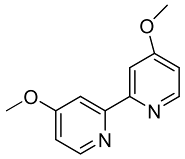

S5 73%

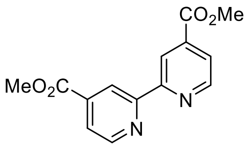

S6 25%

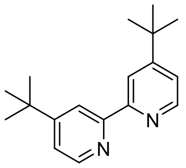

S7 78%

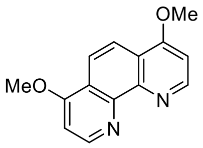

S8 72%

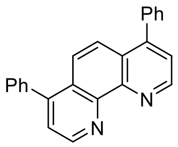

S9 80%

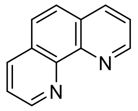

S10 84%

Table S9 The amount of the ligand added:

Entry	Ligand [mol%]	Yield of **4a** [%]
1	40	78
2	30	90
**3**	**20**	**90/87[b]**
4	15	70

[a]**Reaction conditions:** oxetane (**1**) (0.1 mmol, 1 equiv), 4-iodotoluene (**2**) (1.5 equiv), Zn (3 equiv), NH_4Cl (3 equiv), HME (**3**) (5 mol%), $NiCl_2$(DME) (15 mol%), ligand, TMSBr (2 equiv), $MeCN_{anh}$ (c = 0.1 M), blue LEDs, 16h, yield was determined by GC; [b]Isolated yield.

Table S10 The influence of Zn and NH_4Cl amounts[a]

Entry	Zn (equiv)	NH_4Cl (equiv)	Yield of **4a** [%]
**1**	**3**	**3**	**90/87[b]**
2	4	3	65
3	2	3	33
4	3	1.5	49
5	3	0	23

[a]**Reaction conditions:** oxetane (**1**) (0.1 mmol, 1 equiv), 4-iodotoluene (**2**) (1.5 equiv), Zn, NH_4Cl, HME (**3**) (5 mol%), $NiCl_2$(DME) (15 mol%), dtbby (20 mol%), TMSBr (2 equiv), $MeCN_{anh}$ (c = 0.1 M), blue LEDs, 16h, yield was determined by GC; [b]Isolated yield.

4. Full Optimization of Giese-type addition Parameters

<u>Initial studies:</u>

According to our previous experience with epoxides we decided to conduct the initial experiment with oxetane under the developed conditions.

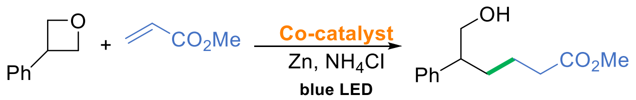

1 20 21

Reaction conditions: oxetane **1** (0.2 mmol, 1 equiv), methyl acrylate (**20**) (1.5 equiv), Zn (1.5 equiv), NH_4Cl (3 equiv), HME (**3**) (5 mol%), $MeCN_{anh}$ (c = 0.1 M), blue LEDs (single diode, 10 W), 30 min, dodecane as an internal standard.

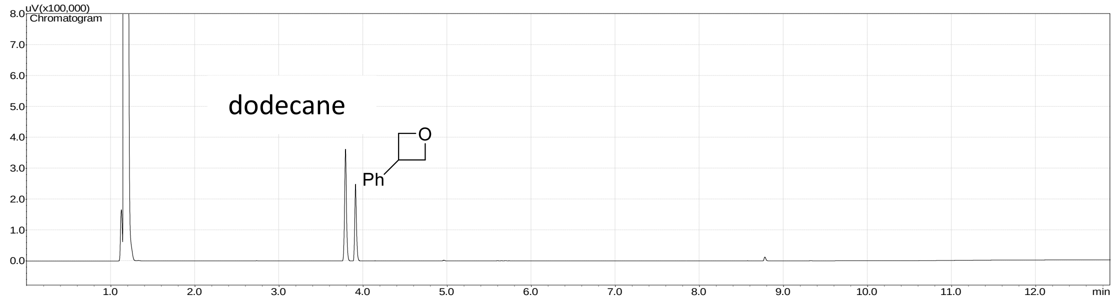

The reaction performed utilizing the conditions reported for epoxides conditions does not proceed. The GC chromatogram indicates two peaks which corresponds to dodecane (internal standard) and the unreacted substrate. Thus, the model reaction with the addition of a Lewis acid (TMSBr) was carried out.

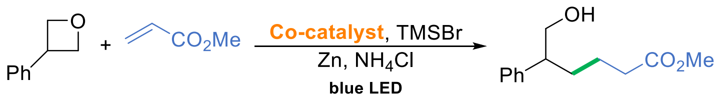

1 20 21

Reaction conditions: oxetane (**1**) (0.2 mmol, 1 equiv), methyl acrylate (**20**) (1.5 equiv), Zn (1.5 equiv), NH_4Cl (3 equiv), HME (**3**) (5 mol%), $MeCN_{anh}$ (c = 0.1 M), blue LEDs (single diode, 10 W), 30 min, TMSBr (2 equiv), dodecane as an internal standard.

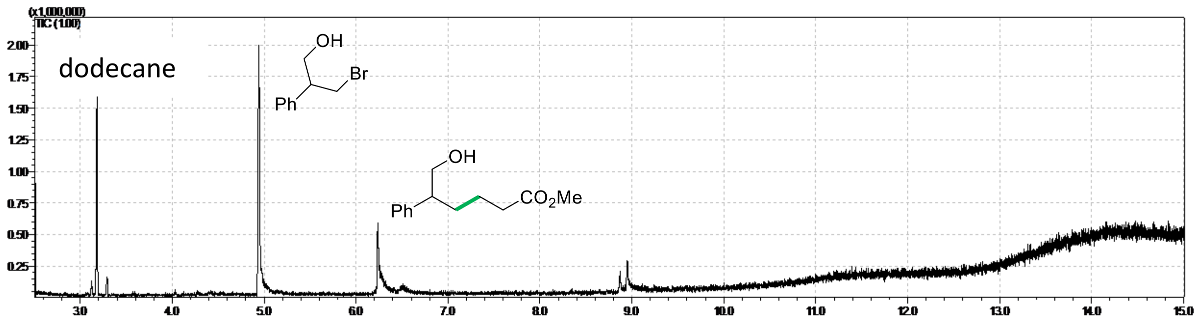

The reaction with the addition of a Lewis acid affords the desired product. The GC chromatogram shows three peaks which corresponds to dodecane (internal standard), bromohydrin ***5b*** *and product* ***21****.*

<u>*Conclusion*</u>*: The reaction requires the use of a Lewis acid.*

Model reaction:

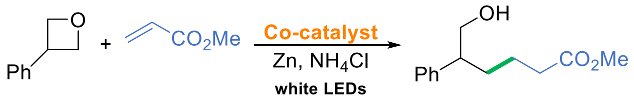

Reaction conditions: oxetane (**1**) (0.2 mmol, 1 equiv), methyl acrylate (**20**) (1.5 equiv), Zn (3 equiv), NH_4Cl (1.5 equiv), HME (**3**) (5 mol%), $MeCN_{anh}$ (c = 0.1 M), white LEDs, 16 h; each reaction was quenched by treatment with 2 equiv of citric acid.

Table S11 Background experiments[a]

Entry	Deviation from the Initial Conditions	Yield of **21** [%]
**1**	**none**	**59/57**[b]
2	no Co-catalyst	Product not observed
3	no reducing agent	Product not observed
4	no light	44
5	no light, 30 °C	38
6	MeCN („wet")	24

[a]**Reaction conditions:** oxetane (**1**) (0.1 mmol, 1 equiv), methyl acrylate (**20**) (2 equiv), Zn (3 equiv), NH_4Cl (3 equiv), HME (**3**) (5 mol%), MeCN (*c* = 0.1 M), blue LEDs, 16 h, yields determined by GC; [b]Isolated yield.

Table S12 Screening of Lewis acid[a]

Entry	Lewis acid	Yield of **21** [%]
**1**	**TMSBr**	**57**
2	TMSI	0
3	TMSCl	0
4	$AlCl_3$	0
5	ZnOTf	0
6	$MgCl_2$	0
7	$(EtO)_3SiCl$	6

[a]**Reaction conditions:** oxetane (**1a**) (0.1 mmol, 1 equiv), methyl acrylate (**2a**) (2 equiv), Zn (3 equiv), NH_4Cl (3 equiv), HME (**3**) (5 mol%), Lewis acid (2 equiv), $MeCN_{anh}$ (*c* = 0.1 M), blue LEDs, 16 h, yields determined by GC; [b]Isolated yield.

Table S13 The influence of light on the model reaction[a]

Entry	Light	Yield of **21** [%]
1	Blue LEDs (tape)	57
2	Green LEDs (tape)	65
**3**	**White LEDs (tape)**	**69**

[a]**Reaction conditions:** oxetane (**1**) (0.1 mmol, 1 equiv), methyl acrylate (**20**) (2 equiv), Zn (3 equiv), NH_4Cl (3 equiv), HME (**3**) (5 mol%), TMSBr (2 equiv), $MeCN_{anh}$ (*c* = 0.1 M), 16 h, yields determined by GC.

Table S14 Optimization of the substrates ratio[a]

Entry	Oxetane **1** (equiv)	Olefin **20** (equiv)	Yield of **21** [%]
1	1	2	69
2	2	1	24
3	3	1	32
4	1	3	18
**5**	**1**	**1.5**	**77**

[a]**Reaction conditions:** oxetane (**1**), methyl acrylate (**20**) (2 equiv), Zn (3 equiv), NH_4Cl (3 equiv), HME (**3**) (5 mol%), TMSBr (2 equiv), $MeCN_{anh}$ (c = 0.1 M), white LEDs, 16 h, yields determined by GC.

Table S15 Screening of the cobalt catalyst[a]

Entry	Co-catalyst	Yield of **21** [%]
**1**	**3**	**77**
2	S1	16
3	S2	31
4	S3	0

[a]**Reaction conditions:** oxetane (**1**) (0.1 mmol, 1 equiv), methyl acrylate (**20**) (1.5 equiv), Zn (3 equiv), NH_4Cl (3 equiv), Co-catalyst (5 mol%), TMSBr (2 equiv), $MeCN_{anh}$ (c = 0.1 M), white LEDs, 16 h, yields determined by GC.

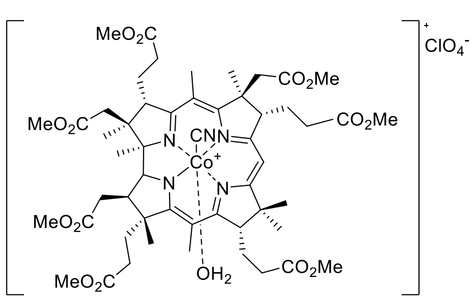

Aqua(cyano)heptamethyl cobyrinate (3)

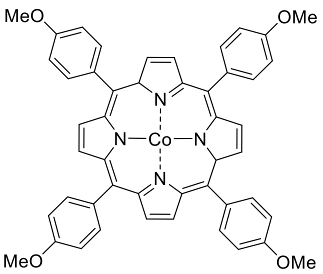

5,10,15,20-Tetrakis(4-methoxyphenyl)-21*H*,23*H*-porphine cobalt (II) (S1)

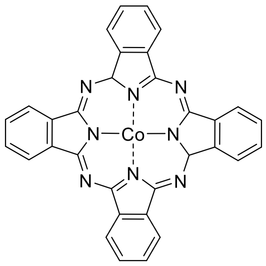

Cobalt(II) phthalocyanine (S2)

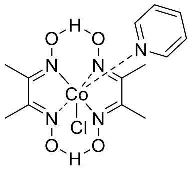

Chloro(pyridine)bis (dimethylglyoximato)cobalt(III) (S3)

Table S16 HME (3) – catalyst loading[a]

Entry	Catalyst loading [%]	Yield of **21** [%]
1	2.5	67
**2**	**5**	**77**
3	7.5	61

[a]**Reaction conditions:** oxetane (**1**) (0.1 mmol, 1 equiv), methyl acrylate (**20**) (1.5 equiv), Zn (3 equiv), NH_4Cl (3 equiv), HME (**3**), TMSBr (2 equiv), $MeCN_{anh}$ (c = 0.1 M), white LEDs, 16 h, yields determined by GC.

Table S17 Optimization of the solvent for HME (3) catalyzed reaction[a]

Entry	Solvent	Yield of **21** [%]
**1**	**MeCN**	**77**
2	DMA	23
3	DME	53
4	THF	44

[a]**Reaction conditions:** oxetane (**1**) (0.1 mmol, 1 equiv), methyl acrylate (**20**) (1.5 equiv), Zn (3 equiv), NH_4Cl (3 equiv), HME (**3**) (5 mol%), TMSBr (2 equiv), solvent (c = 0.1 M), white LEDs, 16 h, yields determined by GC.

Table S18 Concentration of oxetane (1)[a]

Entry	[mol/dm^3]	Yield of **21** [%]
1	0.05	54
**2**	**0.1**	**77**
3	0.2	55

[a]**Reaction conditions:** oxetane (**1**) (0.1 mmol, 1 equiv), methyl acrylate (**20**) (1.5 equiv), Zn (3 equiv), NH_4Cl (3 equiv), HME (**3**) (5 mol%), TMSBr (2 equiv), $MeCN_{anh}$, white LEDs, 16 h, yields determined by GC.

Table S19 The influence of Zn and NH_4Cl amounts[a]

Entry	Zn (equiv)	NH_4Cl (equiv)	Yield of **21** [%]
1	3	3	77
2	1	1	14
3	3	1	74
4	1	3	18
**5**	**3**	**1.5**	**82**
6	4	3	65

[a]**Reaction conditions:** oxetane (**1**) (0.1 mmol, 1 equiv), methyl acrylate (**20**) (1.5 equiv), Zn, NH_4Cl, HME (**3**) (5 mol%), TMSBr (2 equiv), $MeCN_{anh}$ (c = 0.1 M), white LEDs, 16 h, yields determined by GC.

Table S20 The influence of an amount of Lewis acid[a]

Entry	TMSBr (equiv)	Yield of **21** [%]
1	1	39
**2**	**2**	**82/80[b]**
3	3	60

[a]**Reaction conditions:** oxetane (**1**) (0.1 mmol, 1 equiv), methyl acrylate (**20**) (1.5 equiv), Zn (3 equiv), NH_4Cl (1.5 equiv), HME (**3**) (5 mol%), TMSBr, $MeCN_{anh}$ (c = 0.1 M), white LEDs, 16 h, yields determined by GC, [b]Isolated yield.

5. General Procedures

A. General procedure for the Co/Ni-catalyzed cross-electrophile coupling

A glass reaction tube (10 mL) equipped with a magnetic stirring bar was charged with activated Zn^0 dust (39 mg, 0.6 mmol, 3.0 equiv), NH_4Cl (32 mg, 0.6 mmol, 3.0 equiv), catalyst **3** (6.0 mol%, 14.4 mg), dttbpy (20.0 mol%, 12.3 mg), aryl iodide (0.35 mmol, 1.75 equiv), $NiCl_2$(DME) (15.0 mol%, 7 mg) and was sealed with a rubber septum. Then dry MeCN (1 mL) was added, and the resulting mixture was degassed by three freeze-thaw cycles and backfilled with argon. An oxetane (0.2 mmol, 1.0 equiv) followed by TMSBr (52 μL, 2.0 equiv) were added dropwise via a syringe. The resulting mixture was irradiated with blue LED light for 16h at room temperature. The resulting mixture was diluted with MeOH (2 mL) and citric acid was added (77 mg, 2.0 equiv). After 30 min, the reaction mixture was filtered through cotton wool and concentrated *in vacuo*. The crude product was purified by column chromatography.

B. General procedure for the Giese-type reaction

A glass reaction tube (10 mL) equipped with a magnetic stirring bar was charged with activated Zn^0 dust (39 mg, 0.6 mmol, 3.0 equiv), NH_4Cl (16 mg, 0.3 mmol, 1.5 equiv), catalyst **3** (5.0 mol%, 12.4 mg) and was sealed with a rubber septum. Then dry MeCN (2 mL) was added, and the resulting mixture was degassed by three freeze-thaw cycles and backfilled with argon. An oxetane (0.2 mmol, 1.0 equiv) followed by a Michael acceptor (0.3 mmol, 1.5 equiv) and TMSBr (52 μL, 2.0 equiv) were added dropwise via a syringe. The resulting mixture was irradiated with white LEDs light for 16 h at room temperature. The resulting mixture was diluted with MeOH (2 mL) and citric acid was added (77 mg, 2.0 equiv). After 30 min, the reaction mixture was filtered through cotton wool and concentrated *in vacuo*. The crude product was purified by column chromatography.

5.1 Note:

- Both reactions can be easily monitored by TLC chromatography (AcOEt/Hexane) using UV visualization, $KMnO_4$ or the Hanessian's stain;
- Reactions require using activated zinc (unactivated zinc gives low yields);
- A mixture containing Zn, NH_4Cl, HME and MeCN should turn from red to dark green and finally brown. The color indicates the reduction of the cobalt cation from Co(III) to Co(I) oxidation state;
- If the color of the reaction does not change (from red to dark brown/green), we highly recommend repeating the zinc activation step;

5.2 Graphical procedure for the Co/Ni cross-electrophile coupling

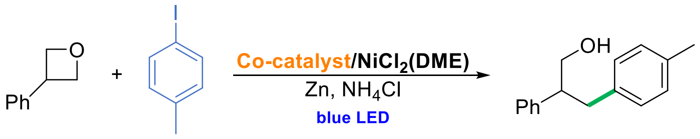

1 **2** **4a**

1. Weighted samples of reagents: A – activated zinc, B – NH_4Cl, C – dtbbpy, D – HME (**3**), E – $NiCl_2$(DME);

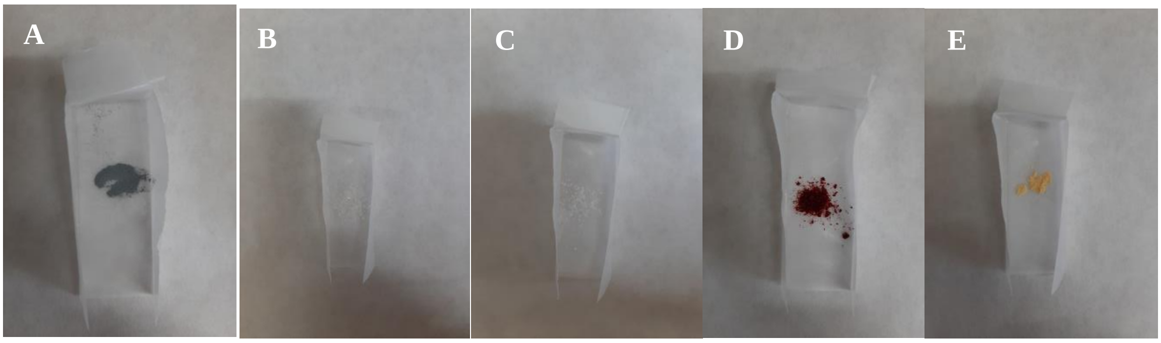

2. Glass reaction tube charged with all reagents;

3. Reagents dissolved in MeCN (*red color of the reaction mixture*);

4. Reaction mixture degassed by three freeze-thaw cycles and backfilled with argon (*note color change to brown*);

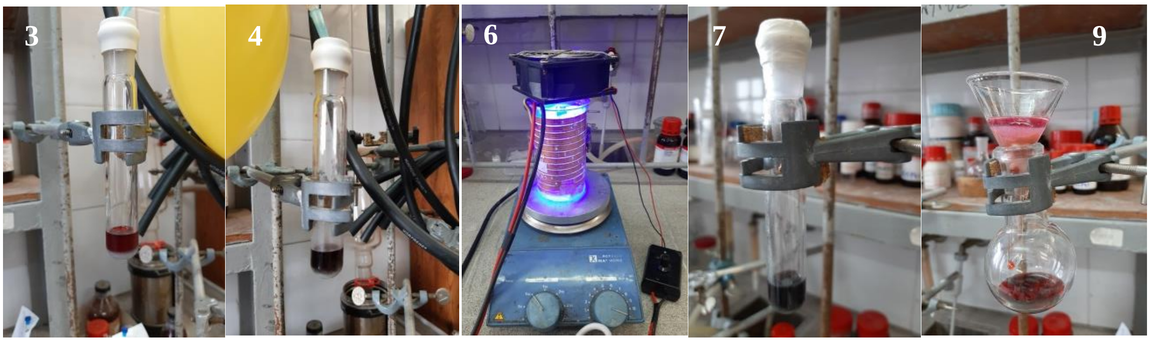

5. Oxetane, aryl halide and TMSBr were added (*if the reagents are solids, they are added on the beginning*);

6. The reaction vessel placed in a photoreactor and irradiated with blue LEDs;

7. The reaction mixture after 16 h (*color change to dark green usually indicates completion of the reaction*);

8. The reaction after 16 h is diluted with MeOH and citric acid was then added;

9. After 30 min of stirring, the reaction mixture is filtered via a cotton pad;

10. The filtrate is concentrated *in vacuo* and purified by column chromatography.

5.3 Graphical procedure for the Giese-type addition

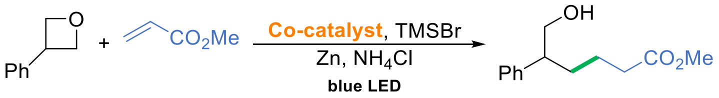

1 **20** **21**

1. Weighted samples of reagents: A – activated zinc, B – NH_4Cl, C – HME (**3**);

2. Glass reaction tube charged with reagents;

3. Reagents dissolved in MeCN (*red color of the reaction mixture*);

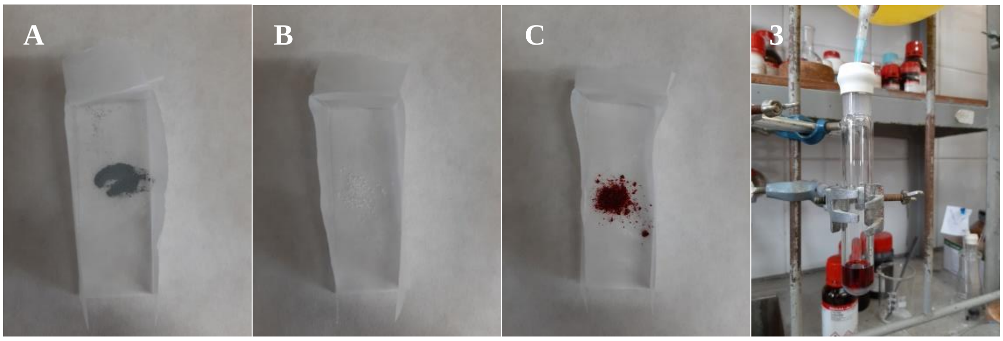

4. Reaction mixture degassed by three freeze-thaw cycles and backfilled with argon (*note color change to brown*);

5. Oxetane, Michael acceptors and TMSBr were added (*if the reagents are solids, they are added on the beginning*);

6. The reaction vessel placed in a photoreactor and irradiated with white LEDs;

7. The reaction mixture after 16 h (*color change to dark green usually indicates completion of the reaction*);

8. The reaction after 16 h is diluted with MeOH and citric acid was then added;

9. After 30 min of stirring, the reaction mixture is filtered via a cotton pad;

10. The filtrate is concentrated *in vacuo* and purified by column chromatography.

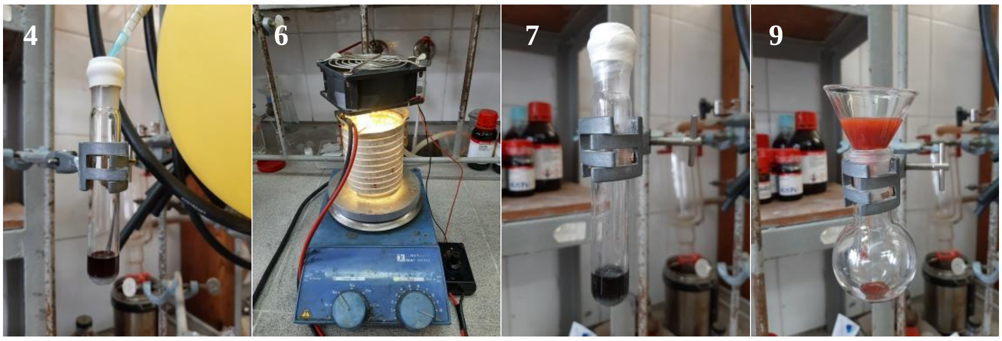

6. Scope and characterization of new compounds

6.1 Substrates

triisopropyl(2-(oxetan-2-yl)ethoxy)silane (S14)

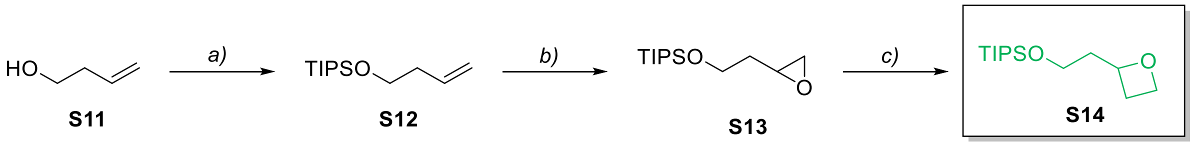

Products **S12** and **S13** (*steps a* and *b*) were synthesized according to literature procedures.[7,8]

General procedure for *step c*:

A mixture of KOBu-*t* (1.1 g, 10.0 mmol, 2.0 equiv) and trimethyloxosulfonium iodide (2.2 g, 10 mmol, 2.0 equiv) in dry *t*-BuOH (13 mL) was stirred magnetically at 50 °C for 1 h. A solution of epoxide **S13** (1.2 g, 5 mmol, 1.0 equiv) in dry *t*-BuOH (10 mL) was then added dropwise and stirred for 24 h. The reaction was concentrated under reduced pressure, and water was added to the residual suspension. The resulting mixture was extracted with DCM, dried over Na_2SO_4, and concentrated in *vacuo* to give the crude product **S14**, which was purified by column chromatography (5:95 EtOAc:Hexane) to afford the title compound as colorless oil, (yield = **56%**).

^{1}H NMR (400 MHz, $CDCl_3$) δ 5.04 – 4.97 (m, 1H), 4.67 (dt, *J* = 8.0, 5.9 Hz, 1H), 4.52 (dt, *J* = 9.1, 5.8 Hz, 1H), 3.81 – 3.71 (m, 2H), 2.73 – 2.65 (m, 1H), 2.45 – 2.37 (m, 1H), 2.08 – 2.00 (m, 1H), 1.96 – 1.88 (m, 1H), 1.12 – 0.99 (m, 3H), 1.04 (d, *J* = 4.3 Hz, 18H).

^{13}C NMR (100 MHz, $CDCl_3$) δ 80.5, 68.6, 59.2, 41.3, 28.0, 18.1, 12.1.

HRMS (ESI) $[M+Na]^+$ calculated for $C_{14}H_{30}O_2SiNa$: 281.1913, found: 281.1911.

3-bromo-2-phenyl-propan-1-ol (5b)

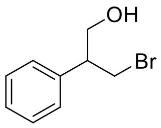

Product **5b** was synthesized according to literature procedure.

NMR data matched those reported in the literature.[9]

^{1}H NMR (500 MHz, $CDCl_3$): δ 7.39 – 7.23 (m, 5H), 3.96 (d, *J* = 6.0 Hz, 2H), 3.75 (dd, *J* = 10.1, 7.6 Hz, 1H), 3.64 (dd, *J* = 10.2, 6.5 Hz, 1H), 3.21 (p, *J* = 6.2 Hz, 1H), 1.51 (s, 1H).

^{13}C NMR (125 MHz, $CDCl_3$): δ 139.7, 128.8, 127.9, 127.6, 64.9, 49.9, 34.1.

6.2 Cross-electrophile coupling: oxetanes

2,3-diphenylpropan-1-ol (4b)

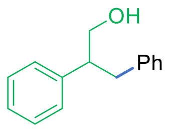

Following the general procedure **5A** compound **4b** was obtained from 3-phenyloxetane (**1**) (27 mg, 0.20 mmol) and iodobenzene (40 µL, 0.35 mmol). The crude product was purified by column chromatography (10:90 AcOEt/Hexane) to afford 36 mg of compound 2,3-diphenylpropan-1-ol (**4b**) as colorless oil, (yield **= 84%**).

NMR data matched those reported in the literature.[10]

^{1}H NMR (400 MHz, $CDCl_3$): δ 7.34 – 7.30 (m, 2H), 7.26 – 7.20 (m, 5H), 7.19 – 7.14 (m, 1H), 7.12 – 7.08 (m, 2H), 3.80 (d, J = 6.3 Hz, 2H), 3.12 – 3.02 (m, 2H), 2.92 (dd, J = 13.3, 7.3 Hz, 1H), 1.34 (br s, 1H).
^{13}C NMR (100 MHz, $CDCl_3$): δ 142.1, 140.1, 129.2, 128.8, 128.4, 128.2, 127.0, 126.2, 66.5, 50.3, 38.9.

4-(1-hydroxy-3-phenylpropan-2-yl)benzonitrile (4d)

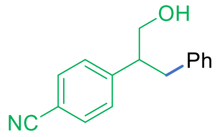

Following the general procedure **5A** compound **4d** was obtained from 4-(oxetan-3-yl)benzonitrile (32 mg, 0.20 mmol) and iodobenzene (40 µL, 0.35 mmol). The crude product was purified by column chromatography (10:40:50 AcOEt/Hexane/DCM) to afford 26 mg of 4-(1-hydroxy-3-phenylpropan-2-yl)benzonitrile (**4d**) as pale yellow oil, (yield **= 71%**).

^{1}H NMR (400 MHz, $CDCl_3$): δ 7.57 (d, J = 8.3 Hz, 2H), 7.30 (d, J = 8.3 Hz, 2H), 7.24 – 7.15 (m, 3H), 7.05 – 7.03 (m, 2H), 3.87 – 3.79 (m, 2H), 3.20 – 3.13 (m, 1H), 3.09 (dd, J = 13.5, 7.0 Hz, 1H), 2.87 (dd, J = 13.5, 7.9 Hz, 1H), 1.46 (br s, 1H).
^{13}C NMR (100 MHz, $CDCl_3$): δ 148.1, 139.1, 132.4, 129.10, 129.08, 128.5, 126.5, 119.0, 110.7, 65.9, 50.5, 38.5.
HRMS (EI) $[M]^+$ calculated for $C_{16}H_{15}NO$: 237.1154, found: 237.1156.

3-phenyl-2-(2-methylphenyl)propan-1-ol (4e)

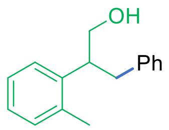

Following the general procedure **5A** compound **4e** was obtained from 3-(2-methylphenyl)oxetane (30 mg, 0.20 mmol) and iodobenzene (40 µL, 0.35 mmol). The crude product was purified by column chromatography (2:48:50 AcOEt/Hexane/DCM) to afford 32 mg of 3-phenyl-2-(2-methylphenyl)propan-1-ol (**4e**) as colorless oil, (yield = **71%**).

NMR data matched those reported in the literature.[11]

^{1}H NMR (400 MHz, CDCl$_3$): δ 7.32 – 7.28 (m, 1H), 7.25 – 7.19 (m, 3H), 7.18 – 7.11 (m, 3H), 7.09 – 7.06 (m, 2H), 3.82 (t, *J* = 5.8 Hz, 2H), 3.49 – 3.39 (m, 1H), 3.03 (dd, *J* = 13.5, 7.2 Hz, 1H), 2.86 (dd, *J* = 13.5, 7.5 Hz, 1H), 2.18 (s, 3H), 1.30 (t, *J* = 5.8 Hz, 1H).

^{13}C NMR (100 MHz, CDCl$_3$): δ 140.2, 137.2, 130.7, 129.1, 128.4, 126.5, 126.4, 126.2, 126.1, 66.0, 45.0, 39.1, 19.8. *(In the aromatic region signals overlap).*

2-(1*H*-indol-1-yl)-3-phenylpropan-1-ol (5)

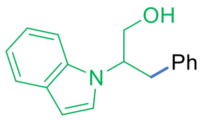

Following the general procedure **5A** compound **5** was obtained from 1-(oxetan-3-yl)-1*H*-indole (35 mg, 0.20 mmol) and iodobenzene (40 µL, 0.35 mmol). The crude product was purified by column chromatography (5:45:50 AcOEt/Hexane/DCM) to afford 26 mg of 2-(1*H*-indol-1-yl)-3-phenylpropan-1-ol (**5**) as colorless oil, (yield = **51%**).

^{1}H NMR (400 MHz, CDCl$_3$): δ 7.63 (d, *J* = 7.8 Hz, 1H), 7.33 (d, *J* = 8.2 Hz, 1H), 7.26 – 7.16 (m, 5H), 7.13 – 7.08 (m, 3H), 6.56 (d, *J* = 3.2 Hz, 1H), 4.73 – 4.66 (m, 1H), 4.00 – 3.90 (m, 2H), 3.25 (dd, *J* = 13.8, 7.3 Hz, 1H), 3.19 (dd, *J* = 13.8, 7.3 Hz, 1H), 1.49 (br s, 1H).

^{13}C NMR (100 MHz, CDCl$_3$): δ 137.7, 136.5, 129.1, 128.8, 128.7, 126.9, 125.0, 121.8, 121.2, 119.8, 109.6, 102.4, 64.2, 59.7, 37.7.

HRMS (ESI) $[M+Na]^+$ calculated for $C_{17}H_{17}NONa$: 274.1208, found: 274.1204.

2-(1-hydroxy-3-phenylpropan-2-yl)isoindoline-1,3-dione (6)

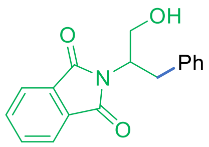

Following the general procedure **5A** compound **6** was obtained from 2-(oxetan-3-yl)isoindoline-1,3-dione (41 mg, 0.20 mmol) and iodobenzene (40 µL, 0.35 mmol). The crude product was purified by column chromatography (15:35:50 AcOEt/Hexane/DCM) to afford 31 mg of 2-(1-hydroxy-3-phenylpropan-2-yl)isoindoline-1,3-dione (**6**) as white solid, (yield = **55%**).

<u>NMR data matched those reported in the literature.</u>[12]

^{1}H NMR (400 MHz, CDCl$_3$) δ 7.80 – 7.75 (m, 2H), 7.70 – 7.65 (m, 2H), 7.22 – 7.19 (m, 4H), 7.18 – 7.12 (m, 1H), 4.67 – 4.60 (m, 1H), 4.12 – 4.04 (m, 1H), 3.96 – 3.91 (m, 1H), 3.20 (d, *J* = 8.2 Hz, 2H), 2.82 (dd, *J* = 8.2, 3.2 Hz, 1H).

^{13}C NMR (100 MHz, CDCl$_3$) δ 169.1, 137.6, 134.2, 131.8, 129.2, 128.7, 126.8, 123.4, 63.0, 55.4, 34.9.

2-(4-methoxyphenoxy)-3-phenylpropan-1ol (7)

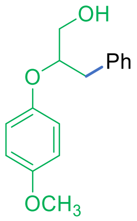

Following the general procedure **5A** compound **7** was obtained from 3-(4-methoxyphenoxy)oxetane (36 mg, 0.20 mmol) and iodobenzene (40 µL, 0.35 mmol). The crude product was purified by column chromatography (5:45:50 AcOEt/Hexane/DCM) to afford 26 mg of 2-(4-methoxyphenoxy)-3-phenylpropan-1ol (**7**) as colorless oil, (yield = **50%**).

^{1}H NMR (400 MHz, CDCl$_3$): δ 7.32 – 7.27 (m, 2H), 7.25 – 7.20 (m, 3H), 6.88 (d, *J* = 9.3 Hz, 2H), 6.83 (d, *J* = 9.3 Hz, 2H), 4.43 – 4.37 (m, 1H), 3.80 – 3.76 (m, 1H), 3.77 (s, 3H), 3.67 (dd, *J* = 11.7, 5.6 Hz, 1H), 3.05 (dd, *J* = 13.8, 5.6 Hz, 1H), 2.92 (dd, *J* = 13.8, 7.6 Hz, 1H), 2.03 (br s, 1H).

^{13}C NMR (100 MHz, CDCl$_3$): δ 154.6, 151.9, 137.6, 129.6, 128.6, 126.7, 118.1, 114.9, 81.3, 63.7, 55.8, 37.0.

HRMS (ESI) $[M+Na]^+$ calculated for $C_{16}H_{18}O_3Na$: 281.1154, found: 281.1145.

2-benzyl-3-(benzyloxy)propan-1-ol (8)

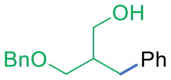

Following the general procedure **5A** compound **8** was obtained from 3-((benzyloxy)methyl)oxetane (36 mg, 0.20 mmol) and iodobenzene (40 µL, 0.35 mmol). The crude product was purified by column chromatography (5:45:50 AcOEt/Hexane/DCM) to afford 33 mg of 2-benzyl-3-(benzyloxy)propan-1-ol (**8**) as colorless oil, (yield = **65%**).

<u>NMR data matched those reported in the literature.</u>[13]

^{1}H NMR (400 MHz, CDCl$_3$): δ 7.36 – 7.24 (m, 7H), 7.21 – 7.14 (m, 3H), 4.51 (d, *J* = 11.9 Hz, 1H), 4.46 (d, *J* = 11.9 Hz, 1H), 3.76 – 3.70 (m, 1H), 3.66 – 3.60 (m, 1H), 3.58 (dd, *J* = 9.1, 4.2 Hz, 1H), 3.48 (dd, *J* = 9.1, 6.7 Hz, 1H), 2.70 – 2.61 (m, 2H), 2.41 (br s, 1H), 2.18 – 2.09 (m, 1H).

^{13}C NMR (100 MHz, CDCl$_3$): δ 140.1, 138.2, 129.2, 128.6, 128.5, 127.9, 127.8, 126.2, 73.6, 72.9, 65.4, 42.8, 34.7.

2-benzyl-3-(benzyloxy)-2-methylpropan-1-ol (9)

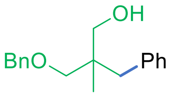

Following the general procedure **5A** compound **9** was obtained from 3-methyl-3-(3-phenyl-2-oxapropyl)oxetane (38 mg, 0.20 mmol) and iodobenzene (40 µL, 0.35 mmol). The crude product was purified by column chromatography (2:48:50 AcOEt/Hexane/DCM) to afford 22 mg of 2-benzyl-3-(benzyloxy)-2-methylpropan-1-ol (**9**) as colorless oil, (yield = **40%**).

^{1}H NMR (400 MHz, $CDCl_3$): δ 7.39 – 7.28 (m, 5H), 7.27 – 7.22 (m, 2H), 7.21 – 7.14 (m, 3H), 4.52 (s, 2H), 3.55 (d, J = 10.8 Hz, 1H), 3.47 (dd, J = 10.8, 5.3 Hz, 1H), 3.32 (s, 2H), 2.77 (d, J = 13.2 Hz, 1H), 2.72 (d, J = 13.2 Hz, 1H), 2.68 (br s, 1H), 0.75 (s, 3H).

^{13}C NMR (100 MHz, $CDCl_3$): δ 138.1, 130.8, 128.7, 128.0, 127.9, 127.8, 126.2, 77.6, 73.7, 70.4, 40.2, 40.1, 19.1. *(In the aromatic region signals overlap).*

HRMS (ESI) $[M+Na]^+$ calculated for $C_{18}H_{22}O_2Na$: 293.1517, found: 293.1521.

1-(benzyloxy)-5-phenylpentan-3-ol (10)

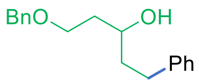

Following the general procedure **5A** compound **10** was obtained from 2-(2-(benzyloxy)ethyl)oxetane (38 mg, 0.20 mmol) and iodobenzene (40 µL, 0.35 mmol). The crude product was purified by column chromatography (5:45:50 AcOEt/Hexane/DCM) to afford 41 mg of 1-(benzyloxy)-5-phenylpentan-3-ol (**10**) as colorless oil, (yield = **76%**).

NMR data matched those reported in the literature.[14]

^{1}H NMR (400 MHz, $CDCl_3$): δ 7.36 – 7.24 (m, 7H), 7.20 – 7.15 (m, 3H), 4.51 (s, 2H), 3.87 – 3.81 (m, 1H), 3.75 – 3.70 (m, 1H), 3.67 – 3.62 (m, 1H), 2.91 (d, J = 3.2 Hz, 1H), 2.80 (ddd, J = 13.9, 9.9, 5.7 Hz, 1H), 2.67 (ddd, J = 13.9, 9.7, 6.7 Hz, 1H), 1.86 – 1.69 (m, 4H).

^{13}C NMR (100 MHz, $CDCl_3$): δ 142.4, 138.0, 128.60, 128.58, 128.5, 127.9, 127.8, 125.9, 73.5, 70.9, 69.3, 39.3, 36.6, 32.1.

1-phenyl-5-((triisopropylsilyl)oxy)pentan-3-ol (11)

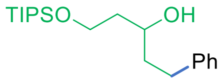

Following the general procedure **5A** compound **11** was obtained from triisopropyl(2-(oxetan-2-yl)ethoxy)silane (51 mg, 0.20 mmol) and iodobenzene (40 µL, 0.35 mmol). The crude product was purified by column chromatography (5:95 AcOEt/Hexane) to afford 34 mg of 1-phenyl-5-((triisopropylsilyl)oxy)pentan-3-ol (**11**) as colorless oil, (yield = **51%**).

^{1}H NMR (400 MHz, $CDCl_3$) δ 7.29 – 7.25 (m, 2H), 7.23 – 7.15 (m, 3H), 4.03 – 3.98 (m, 1H), 3.94 – 3.86 (m, 2H), 3.62 (d, J = 2.1 Hz, 1H), 2.82 (ddd, J = 13.9, 10.2, 5.5 Hz, 1H), 2.69 (ddd, J = 13.9, 10.0, 6.5 Hz, 1H), 1.85 (dddd, J = 13.6, 10.0, 8.0, 5.5 Hz, 1H), 1.76 – 1.63 (m, 3H), 1.16 – 1.05 (m, 3H), 1.08 (d, J = 5.2 Hz, 18H).

^{13}C NMR (100 MHz, $CDCl_3$) δ 142.6, 128.6, 128.5, 125.8, 71.9, 63.6, 39.4, 38.5, 32.1, 18.1, 11.9.

HRMS (ESI) $[M+Na]^+$ calculated for $C_{20}H_{36}O_2SiNa$: 359.2382, found: 359.2388.

6.3 Cross-electrophile coupling: aryl halides

2-phenyl-3-(4-methylphenyl)propan-1-ol (4a)

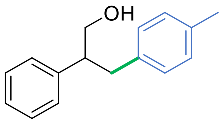

Following the general procedure **5A** compound **4a** was obtained from 3-phenyloxetane (**1**) (27 mg, 0.20 mmol) and 4-iodotoluene (**2**) (76 mg, 0.35 mmol). The crude product was purified by column chromatography (15:85 AcOEt/Hexane) to afford 36 mg of 2-phenyl-3-(4-methylphenyl)propan-1-ol (**4a**) as colorless oil, (yield **= 80%**).

<u>NMR data matched those reported in the literature.</u>[15]

^{1}H NMR (400 MHz, $CDCl_3$): δ 7.35 – 7.30 (m, 2H), 7.26 – 7.20 (m, 3H), 7.04 (d, *J* = 8.0 Hz, 2H), 6.99 (d, *J* = 8.0 Hz, 2H), 3.79 (dd, *J* = 6.1, 2.6 Hz, 2H), 3.11 – 3.05 (m, 1H), 2.99 (dd, *J* = 13.6, 7.4 Hz, 1H), 2.89 (dd, *J* = 13.6, 7.5 Hz, 1H), 2.30 (s, 3H), 1.31 (br s, 1H).

^{13}C NMR (100 MHz, $CDCl_3$): δ 142.2, 136.9, 135.2, 129.00, 128.96, 128.7, 128.2, 126.9, 66.5, 50.3, 38.4, 21.0.

3-(4-methoxyphenyl)-2-phenylpropan-1-ol (12a)

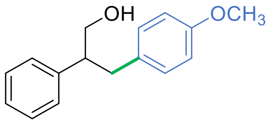

Following the general procedure **5A** compound **12a** was obtained from 3-phenyloxetane (**1**) (27 mg, 0.20 mmol) and 4-iodoanisole (82 mg, 0.35 mmol). The crude product was purified by column chromatography (5:45:50 AcOEt/Hexane/DCM) to afford 23 mg of 3-(4-methoxyphenyl)-2-phenylpropan-1-ol (**12a**) as colorless oil, (yield **= 48%**).

<u>NMR data matched those reported in the literature.</u>[15]

^{1}H NMR (400 MHz, $CDCl_3$): δ 7.34 – 7.29 (m, 2H), 7.25 – 7.18 (m, 3H), 7.00 (d, *J* = 8.7 Hz, 2H), 6.77 (d, *J* = 8.7 Hz, 2H), 3.81 – 3.76 (m, 2H), 3.76 (s, 3H), 3.09 – 3.02 (m, 1H), 2.97 (dd, *J* = 13.6, 7.3 Hz, 1H), 2.86 (dd, *J* = 13.6, 7.5 Hz, 1H), 1.29 (br s, 1H).

^{13}C NMR (100 MHz, $CDCl_3$): δ 158.1, 142.2, 132.1, 130.1, 128.8, 128.3, 127.0, 113.8, 66.5, 55.4, 50.5, 38.0.

4-(3-hydroxy-2-phenylpropyl)benzonitrile (12b)

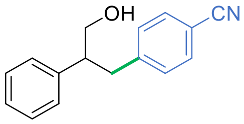

Following the general procedure **5A** compound **12b** was obtained from 3-phenyloxetane (**1**) (27 mg, 0.20 mmol) and 4-iodobenzonitrile (80 mg, 0.35 mmol). The crude product was purified by column chromatography (10:40:50 AcOEt/Hexane/DCM) to afford 24 mg 4-(3-hydroxy-2-phenylpropyl)benzonitrile (**12b**) as white solid, (yield **= 51%**).

m.p. 62.0 – 62.9 ˚C

^{1}H NMR (400 MHz, $CDCl_3$) δ 7.48 (d, *J* = 8.4 Hz, 2H), 7.32 – 7.21 (m, 3H), 7.15 – 7.11 (m, 4H), 3.82 – 3.79 (m, 2H), 3.17 (dd, *J* = 13.1, 6.1 Hz, 1H), 3.10 – 3.04 (m, 1H), 2.94 (dd, *J* = 13.1, 8.4 Hz, 1H), 1.43 (br s, 1H).

^{13}C NMR (100 MHz, CDCl$_3$) δ 145.8, 140.9, 132.1, 130.0, 128.9, 128.1, 127.3, 119.1, 110.1, 66.4, 50.1, 38.8.

HRMS (ESI) $[M+Na]^+$ calculated for $C_{16}H_{15}NONa$: 260.1051, found: 260.1046.

2-phenyl-3-(4-(trifluoromethyl)phenyl)propan-1-ol (12c)

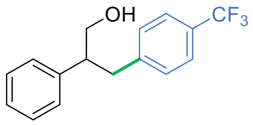

Following the general procedure **5A** compound **12c** was obtained from 3-phenyloxetane (**1**) (27 mg, 0.20 mmol) and 4-iodobenzotrifluoride (51 µL, 0.35 mmol). The crude product was purified by column chromatography (5:45:50 AcOEt/Hexane/DCM) to afford 44 mg of 2-phenyl-3-(4-(trifluoromethyl)phenyl)propan-1-ol (**12c**) as white solid, (yield = **79%**).

m.p. 60.6 – 61.6 ˚C

^{1}H NMR (400 MHz, CDCl$_3$): δ 7.47 (d, *J* = 8.0 Hz, 2H), 7.35 – 7.28 (m, 2H), 7.25 – 7.22 (m, 1H), 7.20 – 7.15 (m, 4H), 3.80 (d, *J* = 6.0 Hz, 2H), 3.17 – 3.07 (m, 2H), 2.96 (dd, *J* = 12.4, 7.2 Hz, 1H), 1.46 (br s, 1H).

^{13}C NMR (100 MHz, CDCl$_3$): δ 144.2 (q, *J* = 1.1 Hz), 141.3, 129.5, 128.9, 128.7, 128.5 (q, *J* = 32.1 Hz), 128.2, 127.2, 125.3 (q, *J* = 3.8 Hz), 124.4 (q, *J* = 270.2 Hz), 66.4, 50.1, 38.5.

^{19}F NMR (376 MHz, CDCl$_3$): δ -62.36.

HRMS (ESI) $[M-H]^-$ calculated for $C_{16}H_{14}F_3O$: 279.0997, found: 279.0995.

1-(4-(3-hydroxy-2-phenylpropyl)phenyl)ethanone (12d)

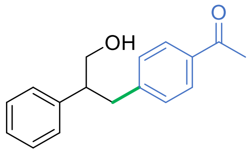

Following the general procedure **5A** compound **12d** was obtained from 3-phenyloxetane (**1**) (27 mg, 0.20 mmol) and 4'-iodoacetophenone (86 mg, 0.35 mmol). The crude product was purified by column chromatography (10:40:50 AcOEt/Hexane/DCM) to afford 40 mg of 1-(4-(3-hydroxy-2-phenylpropyl)phenyl)ethanone (**12d**) as colorless semi-solid, (yield = **79%**).

^{1}H NMR (400 MHz, CDCl$_3$): δ 7.80 (d, *J* = 8.4 Hz, 2H), 7.32 – 7.27 (m, 2H), 7.25 – 7.20 (m, 1H), 7.18 – 7.13 (m, 4H), 3.80 (d, *J* = 5.5 Hz, 2H), 3.17 – 3.08 (m, 2H), 2.99 – 2.91 (m, 1H), 2.54 (s, 3H), 1.45 (br s, 1H).

^{13}C NMR (100 MHz, CDCl$_3$): δ 198.0, 145.9, 141.4, 135.4, 129.4, 128.8, 128.5, 128.2, 127.2, 66.5, 50.1, 38.7, 26.6.

HRMS (ESI) $[M+Na]^+$ calculated for $C_{17}H_{18}O_2Na$: 277.1204, found: 277.1198.

methyl 4-(3-hydroxy-2-phenylpropyl)benzoate (12e)

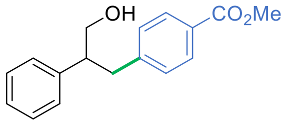

Following the general procedure **5A** compound **12e** was obtained from 3-phenyloxetane (**1**) (27 mg, 0.20 mmol) and methyl 4-iodobenzoate (92 mg, 0.35 mmol). The crude product was purified by column chromatography (gradually from AcOEt/Hexane/DCM 5:45:50 to 8:42:50) to afford 45 mg of methyl 4-(3-hydroxy-2-phenylpropyl)benzoate (**12e**) as colorless oil, (yield = **84%**).

^{1}H NMR (400 MHz, CDCl$_3$): δ 7.88 (d, *J* = 8.3 Hz, 2H), 7.32 – 7.27 (m, 2H), 7.24 – 7.19 (m, 1H), 7.17 – 7.15 (m, 2H), 7.13 (d, *J* = 8.3 Hz, 2H), 3.88 (s, 3H), 3.80 (d, *J* = 5.2 Hz, 2H), 3.16 – 3.07 (m, 2H), 2.97 – 2.90 (m, 1H), 1.43 (br s, 1H).

^{13}C NMR (100 MHz, CDCl$_3$): δ 167.2, 145.6, 141.4, 129.7, 129.2, 128.8, 128.2, 128.1, 127.1, 66.4, 52.1, 50.1, 38.8.

HRMS (ESI) $[M+Na]^+$ calculated for $C_{17}H_{18}O_3Na$: 293.1154, found: 293.1150.

3-(4-chlorophenyl)-2-phenylpropan-1-ol (12f)

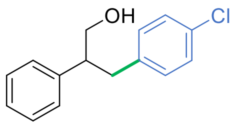

Following the general procedure **5A** compound **12f** was obtained from 3-phenyloxetane (**1**) (27 mg, 0.20 mmol) and 4-chloroiodobenzene (83 mg, 0.35 mmol). The crude product was purified by column chromatography (2:48:50 AcOEt/Hexane/DCM) to afford 28 mg 3-(4-chlorophenyl)-2-phenylpropan-1-ol (**12f**) as colorless oil, (yield = **57%**).

<u>NMR data matched those reported in the literature.</u>[15]

^{1}H NMR (400 MHz, CDCl$_3$): δ 7.33 – 7.28 (m, 2H), 7.25 – 7.21 (m, 1H), 7.18 – 7.15 (m, 4H), 6.99 (d, *J* = 8.4 Hz, 2H), 3.79 (d, *J* = 5.9 Hz, 2H), 3.09 – 3.01 (m, 2H), 2.90 – 2.82 (m, 1H), 1.36 (br s, 1H).

^{13}C NMR (100 MHz, CDCl$_3$): δ 141.5, 138.5, 131.9, 130.5, 128.8, 128.5, 128.2, 127.1, 66.4, 50.3, 38.1.

3-(4-bromophenyl)-2-phenylpropan-1-ol (12g)

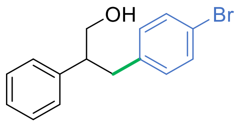

Following the general procedure **5A** compound **12g** was obtained from 3-phenyloxetane (**1**) (27 mg, 0.20 mmol) and 4-bromoiodobenzene (99 mg, 0.35 mmol). The crude product was purified by column chromatography (2:48:50 AcOEt/Hexane/DCM) to afford 29 mg of 3-(4-bromophenyl)-2-phenylpropan-1-ol (**12g**) as colorless oil, (yield = **50%**).

^{1}H NMR (400 MHz, CDCl$_3$) δ 7.34 – 7.28 (m, 4H), 7.26 – 7.21 (m, 1H), 7.18 – 7.16 (m, 2H), 6.93 (d, *J* = 8.3 Hz, 2H), 3.78 (d, *J* = 6.0 Hz, 2H), 3.08 – 2.99 (m, 2H), 2.88 – 2.82 (m, 1H), 1.39 (br s, 1H).

^{13}C NMR (100 MHz, CDCl$_3$) δ 141.5, 139.0, 131.4, 130.9, 128.8, 128.2, 127.1, 120.0, 66.4, 50.2, 38.1.

HRMS (EI) $[M]^+$ calculated for $C_{15}H_{15}BrO$: 290.0306, found: 290.0313.

3-(4-fluorophenyl)-2-phenylpropan-1-ol (12h)

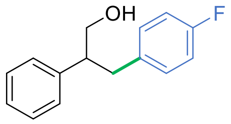

Following the general procedure **5A** compound **12h** was obtained from 3-phenyloxetane (**1**) (27 mg, 0.20 mmol) and 4-fluoroiodobenzene (41 µL, 0.35 mmol). The crude product was purified by column chromatography (2:48:50

AcOEt/Hexanes/DCM) to afford 28 mg of 3-(4-fluorophenyl)-2-phenylpropan-1-ol (**12h**) as colorless oil, (yield = **60%**).

^{1}H NMR (400 MHz, CDCl$_3$): δ 7.34 – 7.28 (m, 2H), 7.26 – 7.21 (m, 1H), 7.20 – 7.15 (m, 2H), 7.03 – 6.99 (m, 2H), 6.92 – 6.86 (m, 2H), 3.79 (d, *J* = 5.9 Hz, 2H), 3.08 – 3.01 (m, 2H), 2.90 – 2.84 (m, 1H), 1.36 (br s, 1H).
^{13}C NMR (100 MHz, CDCl$_3$): δ 161.5 (d, *J* = 243.7 Hz), 141.7, 135.6 (d, *J* = 3.3 Hz), 130.5 (d, *J* = 7.8 Hz), 128.8, 128.2, 127.1, 115.1 (d, *J* = 21.1 Hz), 66.4, 50.5 (d, *J* = 0.6 Hz), 38.0.
^{19}F NMR (376 MHz, CDCl$_3$): δ -117.35.
HRMS (EI) $[M]^+$ calculated for $C_{15}H_{15}FO$: 230.1107, found: 230.1106.

3-(4-(hydroxymethyl)phenyl)-2-phenylpropan-1-ol (13)

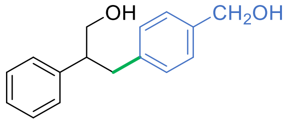

Following modified general procedure **5A** compound **13** was obtained from 3-phenyloxetane (**1**) (27 mg, 0.20 mmol) and 4-iodobenzyl alcohol (82 mg, 0.35 mmol) using 4.0 equiv of TMSBr. The crude product was purified by column chromatography (15:85 AcOEt/DCM) to afford 16 mg of 3-(4-(hydroxymethyl)phenyl)-2-phenylpropan-1-ol (**13**) as colorless oil, (yield = **33%**).

^{1}H NMR (400 MHz, CDCl$_3$): δ 7.34-7.29 (m, 2H), 7.25-7.18 (m, 5H), 7.08 (d, *J* = 8.0 Hz, 2H), 4.62 (s, 2H), 3.79 (d, *J* = 5.8 Hz, 2H), 3.12-3.01 (m, 2H), 2.91 (dd, *J* = 13.0, 7.2 Hz, 1H), 1.65 (br s, 1H), 1.36 (br s, 1H).
^{13}C NMR (100 MHz, CDCl$_3$): δ 141.9, 139.6, 138.7, 129.4, 128.8, 128.2, 127.2, 127.0, 66.5, 66.3, 50.3, 38.5.
HRMS (ESI) $[M+Na]^+$ calculated for $C_{16}H_{18}O_2Na$: 265.1204, found: 265.1196.

2-phenyl-3-(*N*-tosyl-1*H*-indol-5-yl)propan-1-ol (14)

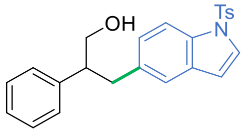

Following the general procedure **5A** compound **14** was obtained from 3-phenyloxetane (**1**) (27 mg, 0.20 mmol) and 5-iodo-1-(4-methylphenylsulfonyl)indole (138 mg, 0.35 mmol). The crude product was purified by column chromatography (gradually from AcOEt/Hexanes/DCM 2:48:50 to 5:45:50) to afford 41 mg of 2-phenyl-3-(*N*-tosyl-1*H*-indol-5-yl)propan-1-ol (**14**) as white solid, (yield = **50%**).
m.p. 121.9 – 122.3 ˚C
^{1}H NMR (400 MHz, CDCl$_3$) δ 7.84 (d, *J* = 8.5 Hz, 1H), 7.74 (d, *J* = 8.4 Hz, 2H), 7.50 (d, *J* = 3.6 Hz, 1H), 7.31 – 7.16 (m, 8H), 7.05 (dd, *J* = 8.5, 1.5 Hz, 1H), 6.55 (d, *J* = 3.6 Hz, 1H), 3.76 (d, *J* = 5.3 Hz, 2H), 3.11 – 3.05 (m, 2H), 3.00 – 2.94 (m, 1H), 2.33 (s, 3H), 1.35 (br s, 1H).
^{13}C NMR (100 MHz, CDCl$_3$) δ 144.9, 142.1, 135.5, 135.2, 133.6, 131.0, 130.0, 128.8, 128.2, 127.0, 126.9, 126.6, 126.0, 121.6, 113.4, 109.0, 66.4, 50.5, 38.6, 21.7.
HRMS (ESI) $[M+Na]^+$ calculated for $C_{24}H_{23}NO_3SNa$: 428.1296, found: 428.1299.

3-(naphthalen-1-yl)-2-phenylpropan-1-ol (15)

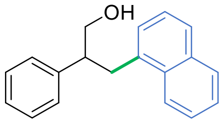

Following the general procedure **5A** compound **14** was obtained from 3-phenyloxetane (**1**) (27 mg, 0.20 mmol) and 1-iodonaphthalene (51 µL, 0.35 mmol). The crude product was purified by column chromatography (2:48:50 AcOEt/Hexane/DCM) to afford 25 mg of 3-(naphthalen-1-yl)-2-phenylpropan-1-ol (**15**) as colorless oil, (yield = **47%**).

^{1}H NMR (400 MHz, CDCl$_3$): δ 8.05 (d, *J* = 8.2 Hz, 1H), 7.86 – 7.83 (m, 1H), 7.69 (d, *J* = 8.2 Hz, 1H), 7.52 – 7.45 (m, 2H), 7.33 – 7.20 (m, 6H), 7.15 (d, *J* = 6.9 Hz, 1H), 3.86 – 3.80 (m, 2H), 3.52 (dd, *J* = 13.5, 7.5 Hz, 1H), 3.35 – 3.24 (m, 2H), 1.33 (br s, 1H).

^{13}C NMR (100 MHz, CDCl$_3$): δ 142.5, 136.1, 134.1, 132.1, 129.0, 128.8, 128.2, 127.4, 127.10, 127.05, 126.1, 125.6, 125.4, 124.0, 66.6, 49.2, 36.0.

HRMS (ESI) $[M+Na]^+$ calculated for $C_{19}H_{18}ONa$: 285.1255, found: 249.1248.

2-phenyl-3-(4-((trimethylsilyl)ethynyl)phenyl)propan-1-ol (16)

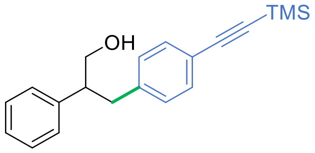

Following the general procedure **5A** compound **16** was obtained from 3-phenyloxetane (**1**) (27 mg, 0.20 mmol) and (4-iodophenylethynyl)trimethylsilane (105 mg, 0.35 mmol). The crude product was purified by column chromatography (2:48:50 AcOEt/Hexane/DCM) to afford 17 mg of 2-phenyl-3-(4-((trimethylsilyl)ethynyl)phenyl)propan-1-ol (**16**) as yellow oil, (yield = **28%**).

^{1}H NMR (400 MHz, CDCl$_3$) δ 7.31 – 7.25 (m, 4H), 7.23 – 7.18 (m, 1H), 7.15 – 7.13 (m, 2H), 6.98 (d, *J* = 8.1 Hz, 2H), 3.77 (d, *J* = 5.7 Hz, 2H), 3.08 – 3.01 (m, 2H), 2.87 – 2.82 (m, 1H), 1.32 (br s, 1H), 0.23 (s, 9H).

^{13}C NMR (100 MHz, CDCl$_3$) δ 141.6, 140.7, 132.0, 129.1, 128.8, 128.2, 127.1, 120.9, 105.3, 93.8, 66.5, 50.3, 38.8, 0.1.

HRMS (ESI) $[M+Na]^+$ calculated for $C_{20}H_{24}OSiNa$: 331.1494, found: 331.1495.

2-phenyl-3-(2-methylphenyl)propan-1-ol (17)

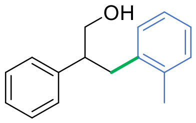

Following the general procedure **5A** compound **17** was obtained from 3-phenyloxetane (**1**) (27 mg, 0.20 mmol) and 2-iodotoluene (45 µL, 0.35 mmol). The crude product was purified by column chromatography (2:48:50 AcOEt/Hexane/DCM) to afford 27 mg of 2-phenyl-3-(2-methylphenyl)propan-1-ol (**17**) as colorless oil, (yield = **60%**).

NMR data matched those reported in the literature.[16]

^{1}H NMR (400 MHz, CDCl$_3$): δ 7.35 – 7.30 (m, 2H), 7.27 – 7.21 (m, 3H), 7.14 – 7.03 (m, 3H), 7.02 – 6.99 (m, 1H), 3.83 (d, *J* = 5.9 Hz, 2H), 3.11 – 3.03 (m, 2H), 2.92 – 2.84 (m, 1H), 2.28 (s, 3H), 1.34 (br s, 1H).

^{13}C NMR (100 MHz, CDCl$_3$): δ 142.4, 138.3, 136.4, 130.4, 130.0, 128.8, 128.1, 127.0, 126.3, 125.9, 66.4, 49.1, 36.3, 19.6.

2-phenyl-3-(3-methylphenyl)propan-1-ol (18)

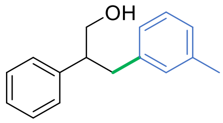

Following the general procedure **5A** compound **18** was obtained from 3-phenyloxetane (**1**) (27 mg, 0.20 mmol) and 3-iodotoluene (45 µL, 0.35 mmol). The crude product was purified by column chromatography (2:48:50 AcOEt/Hexane/DCM) to afford 28 mg of 2-phenyl-3-(3-methylphenyl)propan-1-ol (**18**) as colorless oil, (yield = **63%**).

^{1}H NMR (400 MHz, CDCl$_3$): δ 7.33 – 7.27 (m, 2H), 7.25 – 7.18 (m, 3H), 7.12 – 7.08 (m, 1H), 6.99 – 6.94 (m, 1H), 6.93 – 6.88 (m, 2H), 3.76 (d, *J* = 6.9 Hz, 2H), 3.11 – 3.04 (m, 1H), 2.96 (dd, *J* = 13.5, 7.7 Hz, 1H), 2.87 (dd, *J* = 13.5, 7.3 Hz, 1H), 2.28 (s, 3H), 1.32 (br s, 1H).

^{13}C NMR (100 MHz, CDCl$_3$): δ 142.3, 140.0, 137.9, 130.0, 128.7, 128.3, 128.2, 126.93, 126.91, 126.2, 66.5, 50.2, 38.8, 21.5.

HRMS (ESI) $[M+Na]^+$calculated for $C_{16}H_{18}ONa$: 249.1255, found: 249.1244.

3-(benzo[*d*][1,3]dioxol-5-yl)-2-phenylpropan-1-ol (19)

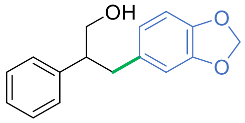

Following the general procedure **5A** compound **19** was obtained from 3-phenyloxetane (**1**) (27 mg, 0.20 mmol) and 5-iodo-1,3-benzodioxole (45 µL, 0.35 mmol). The crude product was purified by column chromatography (gradually from AcOEt/Hexanes/DCM 2:48:50 to 8:42:50) to afford 15 mg of 3-(benzo[*d*][1,3]dioxol-5-yl)-2-phenylpropan-1-ol (**19**) as colorless oil, (yield **= 30%**).

^{1}H NMR (400 MHz, CDCl$_3$): δ 7.34 – 7.29 (m, 2H), 7.25 – 7.17 (m, 3H), 6.66 (d, *J* = 7.9 Hz, 1H), 6.59 (d, *J* = 1.6 Hz, 1H), 6.54 (dd, *J* = 7.9, 1.6 Hz, 1H), 5.89 (s, 2H), 3.78 (d, *J* = 6.7 Hz, 2H), 3.08 – 3.00 (m, 1H), 2.96 (dd, *J* = 13.6, 7.3 Hz, 1H), 2.83 (dd, *J* = 13.6, 7.5 Hz, 1H), 1.34 (br s, 1H).

^{13}C NMR (100 MHz, CDCl$_3$): δ 147.6, 145.9, 142.0, 133.8, 128.8, 128.2, 127.0, 122.1, 109.5, 108.2, 100.9, 66.5, 50.5, 38.5.

HRMS (ESI) $[M+Na]^+$ calculated for $C_{16}H_{16}O_3Na$: 279.0997, found: 279.0994.

6.4 Giese-type addition: oxetanes

6-hydroxy-5-phenylhexanenitrile (22a)

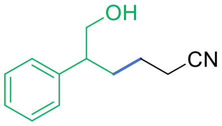

Following the general procedure **5B** compound **22a** was obtained from 3-phenyloxetane (**1**) (27 mg, 0.20 mmol) and acrylonitrile (**39**) (16 mg, 0.30 mmol). The crude product was purified by column chromatography (gradually from AcOEt/Hexane 20:80 to 30:70) to afford 30 mg of 6-hydroxy-5-phenylhexanenitrile (**22a**) as colorless oil, (yield = **80%**).

NMR data matched those reported in the literature.[17]

^{1}H NMR (500 MHz, $CDCl_3$): δ 7.35 – 7.31 (m, 2H), 7.29 – 7.23 (m, 1H), 7.21 – 7.17 (m, 2H), 3.74 (d, *J* = 6.7 Hz, 2H), 2.80 – 2.77 (m, 1H), 2.28 (t, *J* = 7.1 Hz, 2H), 1.94 – 1.87 (m, 1H), 1.77 – 1.69 (m, 1H), 1.62 – 1.50 (m, 3H).

^{13}C NMR (125 MHz, $CDCl_3$): δ 141.2, 128.9, 127.9, 127.2, 119.5, 67.3, 48.1, 30.9, 23.3, 17.2.

6-hydroxy-5-(4-methoxyphenyl)hexanenitrile (22b)

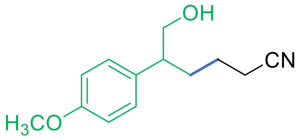

Following the general procedure **5B** compound **22b** was obtained from 3-(4-methoxyphenyl)oxetane (33 mg, 0.20 mmol) and acrylonitrile (**39**) (16 mg, 0.30 mmol). The crude product was purified by column chromatography (20:80 AcOEt/Hexane) to afford 23 mg of 6-hydroxy-5-(4-methoxyphenyl)hexanenitrile (**22b**) as colorless oil, (yield = **53%**).

^{1}H NMR (400 MHz, $CDCl_3$): δ 7.14 – 7.09 (m, 2H), 6.91 – 6.86 (m, 2H), 3.80 (s, 3H), 3.71 (dd, *J* = 6.7, 4.2 Hz, 2H), 2.77 – 2.70 (m, 1H), 2.27 (t, *J* = 7.0 Hz, 2H), 1.93 – 1.83 (m, 1H), 1.74 – 1.65 (m, 1H), 1.61 – 1.51 (m, 2H), 1.38 (br s, 1H).

^{13}C NMR (100 MHz, $CDCl_3$): δ 158.7, 132.9, 128.8, 119.5, 114.4, 67.4, 55.3, 47.2, 31.1, 23.3, 17.2.

HRMS (EI) $[M]^+$ calculated for $C_{13}H_{17}NO_2$: 219.1259, found: 219.1261.

4-(5-cyano-1-hydroxypentan-2-yl)benzonitrile (22c)

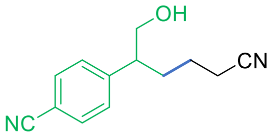

Following the general procedure **5B** compound **22c** was obtained from 4-(oxetan-3-yl)benzonitrile (32 mg, 0.20 mmol) and acrylonitrile (**39**) (16 mg, 0.30 mmol). The crude product was purified by column chromatography (50:50 AcOEt/Hexane) to afford 19 mg of 4-(5-cyano-1-hydroxypentan-2-yl)benzonitrile (**22c**) as colorless oil, (yield = **45%**).

^{1}H NMR (400 MHz, CDCl$_3$): δ 7.63 (d, *J* = 7.9 Hz, 2H), 7.34 (d, *J* = 7.8 Hz, 2H), 3.78 (d, *J* = 6.3 Hz, 2H), 2.80 – 2.83 (m, 1H), 2.32 (t, *J* = 6.9 Hz, 2H), 2.01 – 1.91 (m, 1H), 1.81 – 1.70 (m, 1H), 1.64 – 1.44 (m, 3H).

^{13}C NMR (100 MHz, CDCl$_3$): δ 147.6, 132.7, 128.9, 119.3, 118.8, 111.0, 66.6, 48.2, 30.8, 23.4, 17.3.

HRMS (EI) [M]$^+$ calculated for $C_{13}H_{14}N_2O$: 214.1106, found: 214.1100.

6-hydroxy-5-(2-methylphenyl)hexanenitrile (22d)

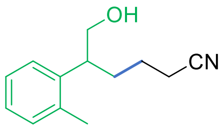

Following the general procedure **5B** compound **22d** was obtained from 3-(2-methylphenyl)oxetane (15 mg, 0.10 mmol) and acrylonitrile (**39**) (8 mg, 0.15 mmol). The crude product was purified by column chromatography (20:80 AcOEt/Hexane) to afford 11 mg of 6-hydroxy-5-(2-methylphenyl)hexanenitrile (**22d**) as colorless oil, (yield = **53%**).

^{1}H NMR (400 MHz, CDCl$_3$): δ 7.23 – 7.12 (m, 4H), 3.73 (d, *J* = 6.6 Hz, 2H), 3.22 – 3.12 (m, 1H), 2.35 (s, 3H), 2.28 (t, *J* = 7.1 Hz, 2H), 1.99 – 1.89 (m, 1H), 1.81 – 1.70 (m, 1H), 1.66 – 1.49 (m, 2H), 1.39 (br s, 1H).

^{13}C NMR (100 MHz, CDCl$_3$): δ 139.3, 136.9, 130.8, 126.7, 126.6, 125.7, 119.4, 67.0, 42.4, 31.1, 23.3, 20.0, 17.4.

HRMS (ESI) [M+Na]$^+$ calculated for $C_{13}H_{17}NONa$: 226.1208, found: 226.1199.

6-hydroxy-5-(1H-indol-1-yl)hexanenitrile (23)

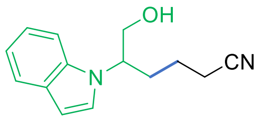

Following the general procedure **5B** compound **23** was obtained from 1-(oxetan-3-yl)-1H-indole (35 mg, 0.20 mmol) and acrylonitrile (**39**) (16 mg, 0.30 mmol). The crude product was purified by column chromatography (30:70 AcOEt/Hexane) to afford 25 mg of 6-hydroxy-5-(1H-indol-1-yl)hexanenitrile (**23**) as colorless oil, (yield = **56%**).

^{1}H NMR (400 MHz, CDCl$_3$): δ 7.64 (d, *J* = 8.0 Hz, 1H), 7.36 (d, *J* = 8.1 Hz, 1H), 7.25 – 7.10 (m, 3H), 6.59 (d, *J* = 3.8 Hz, 1H), 4.49 – 4.43 (m, 1H), 3.95 – 3.86 (m, 2H), 2.32 – 2.16 (m, 2H), 2.07 – 2.04 (m, 2H), 1.68 – 1.52 (m, 2H), 1.47 – 1.36 (m, 1H).

^{13}C NMR (100 MHz, CDCl$_3$): δ 136.6, 128.6, 124.1, 122.0, 121.3, 119.9, 119.0, 109.2, 103.0, 65.3, 57.4, 30.2, 22.1, 16.9.

HRMS (ESI) [M+H]$^+$ calculated for $C_{14}H_{17}N_2O$: 229.1341, found: 229.1337.

5-(9*H*-carbazol-9-yl)-6-hydroxyhexanenitrile (24)

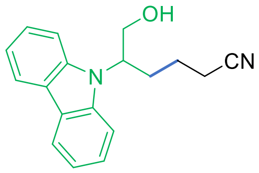

Following the general procedure **5B** compound **24** was obtained from 9-(oxetan-3-yl)-9*H*-carbazole (45 mg, 0.20 mmol) and acrylonitrile (**39**) (16 mg, 0.30 mmol). The crude product was purified by column chromatography

(20:80 AcOEt/Hexane) to afford 38 mg of 5-(9*H*-carbazol-9-yl)-6-hydroxyhexanenitrile (**24**) as colorless oil, (yield = **68%**).

^{1}H NMR (500 MHz, CDCl$_3$): δ 8.11 (d, *J* = 7.8 Hz, 2H), 7.46 – 7.42 (m, 4H), 7.30 – 7.22 (m, 2H), 4.80 – 4.72 (m, 1H), 4.31 (dd, *J* = 11.4, 8.5 Hz, 1H), 4.02 (dd, *J* = 11.3, 5.0 Hz, 1H), 2.51 – 2.39 (m, 1H), 2.27 – 2.06 (m, 3H), 1.63 (s, 1H), 1.59 – 1.46 (m, 1H), 1.37 – 1.25 (m, 1H).

^{13}C NMR (125 MHz, CDCl$_3$): δ 126.0, 120.5, 119.5, 119.0, 63.5, 57.6, 28.1, 22.2, 16.9.

HRMS (ESI) [M+Na]$^+$ calculated for $C_{18}H_{18}N_2ONa$: 301.1317, found: 301.1330.

6-hydroxy-5-(4-methoxyphenoxy)hexanenitrile (25)

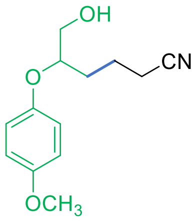

Following the general procedure **5B** compound **25** was obtained from 3-(4-methoxyphenoxy)oxetane (36 mg, 0.20 mmol) and acrylonitrile (**39**) (16 mg, 0.30 mmol). The crude product was purified by column chromatography (10:90 AcOEt/Hexane) to afford 37 mg of 6-hydroxy-5-(4-methoxyphenoxy)hexanenitrile (**25**) as colorless oil, (yield = **79%**).

^{1}H NMR (500 MHz, CDCl$_3$): δ 6.90 – 6.86 (m, 2H), 6.85 – 6.80 (m, 2H), 4.27 – 4.20 (m, 1H), 3.77 (s, 3H), 3.70 (dd, *J* = 11.7, 5.5 Hz, 1H), 2.36 (t, *J* = 6.6 Hz, 2H), 1.93 – 1.73 (m, 5H), 1.60 (br s, 1H).

^{13}C NMR (125 MHz, CDCl$_3$): δ 154.6, 151.9, 119.3, 117.7, 114.9, 79.1, 64.0, 55.7, 30.1, 21.6, 17.3.

HRMS (ESI) [M+Na]$^+$ calculated for $C_{13}H_{17}NO_3Na$: 258.1106, found: 258.1104.

6-(benzyloxy)-5-(hydroxymethyl)hexanenitrile (26)

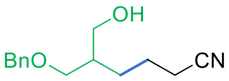

Following the general procedure **5B** compound **26** was obtained from 3-((benzyloxy)methyl)oxetane (36 mg, 0.20 mmol) and acrylonitrile (**39**) (16 mg, 0.30 mmol). The crude product was purified by column chromatography (20:80 AcOEt/Hexane) to afford 26 mg of 6-(benzyloxy)-5-(hydroxymethyl)hexanenitrile (**26**) as colorless oil, (yield = **56%**).

^{1}H NMR (400 MHz, CDCl$_3$): δ 7.39 – 7.27 (m, 5H), 4.56 – 4.46 (m, 2H), 3.73 – 3.70 (m, 1H), 3.67 – 3.61 (m, 1H), 3.60 – 3.55 (m, 1H), 3.51 – 3.45 (m, 1H), 2.33 (t, *J* = 8.0 Hz, 3H), 1.89 – 1.78 (m, 1H), 1.76 – 1.61 (m, 2H), 1.57 – 1.42 (m, 2H).

^{13}C NMR (125 MHz, CDCl$_3$): δ 137.8, 128.5, 127.9, 127.7, 119.5, 73.5, 73.0, 65.2, 40.2, 27.4, 23.3, 17.4.

HRMS (ESI) [M+Na]$^+$ calculated for $C_{14}H_{19}NO_2Na$: 256.1313, found: 256.1308.

8-(benzyloxy)-6-hydroxyoctanenitrile (28)

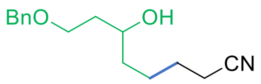

Following the general procedure **5B** compound **28** was obtained from 2-(2-(benzyloxy)ethyl)oxetane (38 mg, 0.20 mmol) and acrylonitrile (**39**) (16 mg, 0.30 mmol). The crude product was purified by column chromatography (20:80 AcOEt/Hexane) to afford 35 mg of 8-(benzyloxy)-6-hydroxyoctanenitrile (**28**) as colorless oil, (yield **= 71%**).

^{1}H NMR (400 MHz, CDCl$_3$): δ 7.38 – 7.27 (m, 5H), 4.52 (s, 2H), 3.85 – 3.80 (m, 1H), 3.76 – 3.69 (m, 1H), 3.68 – 3.62 (m, 1H), 2.98 (br s, 1H), 2.33 (t, *J* = 7.0 Hz, 2H), 1.82 – 1.41 (m, 8H).

^{13}C NMR (100 MHz, CDCl$_3$): δ 137.8, 128.5, 127.8, 127.7, 119.7, 73.4, 71.1, 69.3, 36.4, 36.4, 25.4, 24.8, 17.1.

HRMS (ESI) [M+Na]$^+$ calculated for $C_{15}H_{21}NO_2Na$: 270.1470, found: 270.1465.

6-hydroxy-8-((triisopropylsilyl)oxy)octanenitrile (29)

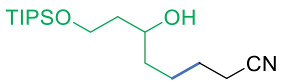

Following the general procedure **5B** compound **29** was obtained from triisopropyl(2-(oxetan-2-yl)ethoxy)silane (52 mg, 0.20 mmol) and acrylonitrile (**39**) (16 mg, 0.30 mmol). The crude product was purified by column chromatography (15:85 AcOEt/Hexane) to afford 12 mg of 6-hydroxy-8-((triisopropylsilyl)oxy)octanenitrile (**29**) as colorless oil, (yield **= 19%**).

^{1}H NMR (500 MHz, CDCl$_3$): δ 4.03 – 3.98 (m, 1H), 3.94 – 3.83 (m, 2H), 3.70 (br s, 1H), 2.35 (t, *J* = 7.1 Hz, 2H), 1.75 – 1.42 (m, 9H), 1.11 – 1.09 (m, 20H).

^{13}C NMR (125 MHz, CDCl$_3$): δ 119.7, 72.1, 63.6, 38.2, 36.6, 25.5, 24.8, 17.9, 17.1, 11.7.

HRMS (ESI) [M+Na]$^+$ calculated for $C_{17}H_{35}NO_2SiNa$: 336.2335, found: 336.2330.

6.5 Giese-type addition: Michael acceptors

methyl 6-hydroxy-5-phenylhexanoate (21)

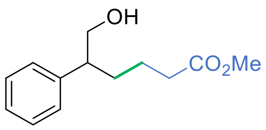

Following the general procedure **5B** compound **21** was obtained from 3-phenyloxetane (**1**) (27 mg, 0.20 mmol) and methyl acrylate (**20**) (26 mg, 0.30 mmol). The crude product was purified by column chromatography (20:80 AcOEt/Hexane) to afford 36 mg of methyl 6-hydroxy-5-phenylhexanoate (**21**) as colorless oil, (yield **= 80%**).

^{1}H NMR (400 MHz, CDCl$_3$): δ 7.34 – 7.30 (m, 2H), 7.25 – 7.19 (m, 3H), 3.73 (dd, *J* = 6.7, 4.2 Hz, 2H), 3.63 (s, 3H), 2.80 – 2.76 (m, 1H), 2.29 – 2.25 (m, 2H), 1.76 – 1.72 (m, 1H), 1.65 – 1.51 (m, 3H), 1.42 (br s, 1H).

^{13}C NMR (100 MHz, CDCl$_3$): δ 173.9, 141.9, 128.7, 128.0, 126.9, 67.4, 51.5, 48.4, 34.0, 31.4, 22.7.

HRMS (ESI) [M+Na]$^+$ calculated for $C_{13}H_{18}O_3Na$: 245.1154, found: 245.1143.

benzyl 6-hydroxy-5-phenylhexanoate (30)

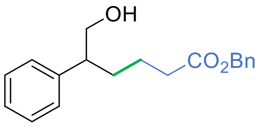

Following the general procedure **5B** compound **30** was obtained from 3-phenyloxetane (**1**) (27 mg, 0.20 mmol) and benzyl acrylate (49 mg, 0.30 mmol). The crude product was purified by column chromatography (10:90 AcOEt/Hexane) to afford 38 mg of benzyl 6-hydroxy-5-phenylhexanoate (**30**) as colorless oil, (yield = **64%**).

^{1}H NMR (400 MHz, $CDCl_3$): δ 7.39 – 7.29 (m, 7H), 7.25 – 7.21 (m, 1H), 7.20 – 7.16 (m, 2H), 5.09 (s, 2H), 3.75 – 3.67 (m, 2H), 2.81 – 2.73 (m, 1H), 2.37 – 2.30 (m, 2H), 1.80 – 1.69 (m, 1H), 1.67 – 1.53 (m, 3H), 1.42 (br s, 1H).

^{13}C NMR (125 MHz, $CDCl_3$): δ 173.2, 141.8, 136.0, 128.7, 128.5, 128.17, 128.16, 128.0, 126.8, 67.4, 66.1, 48.4, 34.2, 31.3, 22.7.

HRMS (ESI) $[M+Na]^+$ calculated for $C_{19}H_{22}O_3Na$: 321.1467, found: 321.1445.

4-methoxyphenyl 6-hydroxy-5-phenylhexanoate (31a)

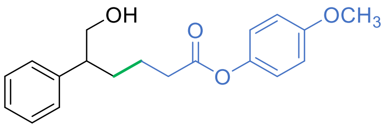

Following the general procedure **5B** compound **31a** was obtained from 3-phenyloxetane (**1**) (27 mg, 0.20 mmol) and 4-methoxyphenyl acrylate (53 mg, 0.30 mmol). The crude product was purified by column chromatography (15:85 AcOEt/Hexane) to afford 38 mg of 4-methoxyphenyl 6-hydroxy-5-phenylhexanoate (**31a**) as colorless oil, (yield = **61%**).

^{1}H NMR (500 MHz, $CDCl_3$): δ 7.36 – 7.33 (m, 2H), 7.27 – 7.21 (m, 3H), 6.97 – 6.95 (m, 2H), 6.88 – 6.86 (m, 2H), 3.79 (s, 3H), 3.75 (dd, *J* = 6.7, 4.3 Hz, 2H), 2.85 – 2.80 (m, 1H), 2.51 (td, *J* = 7.2, 2.7 Hz, 2H), 1.89 – 1.81 (m, 1H), 1.75 – 1.63 (m, 3H), 1.56 (br s, 1H).

^{13}C NMR (125 MHz, $CDCl_3$): δ 172.3, 157.2, 144.1, 141.8, 128.7, 128.0, 126.9, 122.2, 114.4, 67.4, 55.5, 48.4, 34.1, 31.3, 22.7.

HRMS (ESI) $[M+Na]^+$ calculated for $C_{19}H_{22}O_4Na$: 337.1416, found: 337.1399.

4-cyanophenyl 6-hydroxy-5-phenylhexanoate (31b)

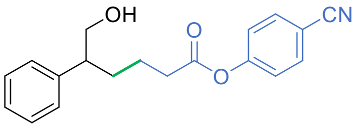

Following the general procedure **5B** compound **31b** was obtained from 3-phenyloxetane **(1)** (27 mg, 0.20 mmol) and 4-cyanophenyl acrylate (52 mg, 0.30 mmol). The crude product was purified by column chromatography (30:70 AcOEt/Hexane) to afford 55 mg of 4-cyanophenyl 6-hydroxy-5-phenylhexanoate (**31b**) as colorless oil, (yield = **89%**).

^{1}H NMR (500 MHz, $CDCl_3$): δ 7.67 (d, *J* = 5.0 Hz, 2H), 7.36 – 7.33 (m, 2H), 7.28 – 7.18 (m, 5H), 3.77 (d, *J* = 6.9 Hz, 2H), 2.84 – 2.80 (m, 1H), 2.61 – 2.50 (m, 2H), 1.90 – 1.83 (m, 1H), 1.77 – 1.61 (m, 3H), 1.31 (br s, 1H).

^{13}C NMR (125 MHz, CDCl$_3$): δ 171.0, 153.9, 141.6, 133.6, 128.8, 128.0, 127.0, 122.7, 118.2, 109.7, 67.4, 48.4, 34.2, 31.2, 22.6.

HRMS (ESI) $[M+Na]^+$ calculated for $C_{19}H_{19}NO_3Na$: 332.1263, found: 332.1271.

pent-4-en-1-yl 6-hydroxy-5-phenylhexanoate (32)

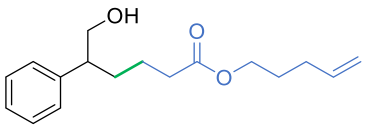

Following the general procedure **5B** compound **32** was obtained from 3-phenyloxetane (**1**) (27 mg, 0.20 mmol) and acrylic ester pent-4-enyl ester (42 mg, 0.30 mmol). The crude product was purified by column chromatography (10:90 AcOEt/Hexane) to afford 40 mg of pent-4-en-1-yl 6-hydroxy-5-phenylhexanoate (**32**) as colorless oil, (yield **= 72%**).

^{1}H NMR (400 MHz, CDCl$_3$): δ 7.34 – 7.30 (m, 2H), 7.25 – 7.17 (m, 3H), 5.78 (ddt, *J* = 16.9, 10.2, 6.6 Hz, 1H), 5.05 – 4.95 (m, 2H), 4.04 (t, *J* = 6.7 Hz, 2H), 3.73 (dd, *J* = 6.6, 3.9 Hz, 2H), 2.81 – 2.74 (m, 1H), 2.31 – 2.21 (m, 2H), 2.13 – 2.05 (m, 2H), 1.76 – 1.51 (m, 7H).

^{13}C NMR (125 MHz, CDCl$_3$): δ 173.5, 141.9, 137.5, 128.7, 128.0, 126.8, 115.3, 67.4, 63.7, 48.4, 34.2, 31.4, 30.0, 27.8, 22.8.

HRMS (ESI) $[M+Na]^+$ calculated for $C_{17}H_{24}O_3Na$: 299.1623, found: 299.1617.

2-phenyl-5-(phenylsulfonyl)pentan-1-ol (33)

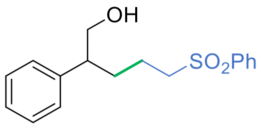

Following the general procedure **5B** compound **33** was obtained from 3-phenyloxetane (**1**) (27 mg, 0.20 mmol) and phenyl vinyl sulfone (51 mg, 0.30 mmol). The crude product was purified by column chromatography (30:70 AcOEt/Hexane) to afford 32 mg of 2-phenyl-5-(phenylsulfonyl)pentan-1-ol (**33**) as colorless oil, (yield **= 53%**).

^{1}H NMR (500 MHz, CDCl$_3$): δ 7.85 – 7.79 (m, 2H), 7.64 – 7.61 (m, 1H), 7.54 – 7.51 (m, 2H), 7.32 – 7.29 (m, 2H), 7.25 – 7.22 (m, 1H), 7.14 – 7.12 (m, 2H), 3.70 (d, *J* = 6.7 Hz, 2H), 3.09 – 2.97 (m, 2H), 2.75 – 2.68 (m, 1H), 1.87 – 1.82 (m, 1H), 1.71 – 1.61 (m, 3H), 1.35 (br s, 1H).

^{13}C NMR (125 MHz, CDCl$_3$): δ 141.1, 139.1, 133.6, 129.2, 128.9, 128.0, 127.9, 127.1, 67.1, 56.1, 48.2, 30.5, 20.7.

HRMS (ESI) $[M+Na]^+$ calculated for $C_{17}H_{20}O_3SNa$: 327.1031, found: 327.1034.

methyl 6-hydroxy-2-methyl-5-phenylhexanoate (34)

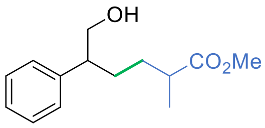

Following the general procedure **5B** compound **34** was obtained from 3-phenyloxetane (**1**) (27 mg, 0.20 mmol) and methacrylic acid methyl ester (30 mg, 0.30 mmol). The crude product was purified by column chromatography

(20:80 AcOEt/Hexane) to afford 19 mg of methyl 6-hydroxy-2-methyl-5-phenylhexanoate (**34**) as colorless oil (a *mixture of diastereoisomers*), (yield = **41%**).

^{1}H NMR (500 MHz, CDCl$_3$): δ 7.34 – 7.31 (m, 2H), 7.25 – 7.18 (m, 3H), 3.74 – 3.70 (m, 2H), 3.64 (s, 3H), 2.79 – 2.70 (m, 1H), 2.46 – 2.35 (m, 1H), 1.77 – 1.66 (m, 1H), 1.64 – 1.51 (m, 2H), 1.39 – 1.28 (m, 2H), 1.10 (dd, *J* = 8.0, 4.0 Hz, 3H).

^{13}C NMR (125 MHz, CDCl$_3$): δ 141.8, 128.7, 127.98, 127.98, 126.8, 67.5, 67.4, 51.5, 51.5, 48.7, 48.5, 39.5, 39.4, 31.5, 31.4, 29.6, 29.3, 17.1, 17.0. *(In the aromatic region signals overlap)*

HRMS (ESI) $[M+Na]^+$ calculated for $C_{14}H_{20}O_3Na$: 259.1310, found: 259.1280.

methyl 6-hydroxy-2,5-diphenylhexanoate (35)

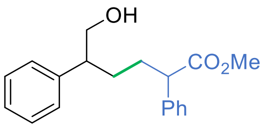

Following the general procedure **5B** compound **35** was obtained from 3-phenyloxetane (**1**) (27 mg, 0.20 mmol) and 2-phenyl-acrylic acid methyl ester (49 mg, 0.30 mmol). The crude product was purified by column chromatography (15:85 AcOEt/Hexane) to afford 33 mg of methyl 6-hydroxy-2,5-diphenylhexanoate (**35**) as colorless oil (a *mixture of diastereoisomers*), (yield = **56%**).

^{1}H NMR (500 MHz, CDCl$_3$): δ 7.36 – 7.08 (m, 10H), 3.68 – 3.65 (m, 2H), 3.61 (d, *J* = 1.4 Hz, 3H), 3.52 – 3.46 (m, 1H), 2.80 – 2.71 (m, 1H), 2.03 – 1.91 (m, 1H), 1.74 – 1.55 (m, 3H), 1.41 (br s, 1H).

^{13}C NMR (125 MHz, CDCl$_3$): δ 141.7, 128.70, 128.67, 128.60, 128.57, 128.00, 127.97, 127.89, 127.80, 127.2, 126.85, 126.82, 67.5, 67.4, 51.93, 51.89, 51.61, 51.56, 48.5, 48.4, 31.2, 31.1, 29.74, 29.66. *(In the aromatic region signals overlap)*

HRMS (ESI) $[M+Na]^+$ calculated for $C_{19}H_{22}O_3Na$: 321.1467, found: 321.1465.

pent-4-yn-1-yl 6-hydroxy-5-phenylhexanoate (36)

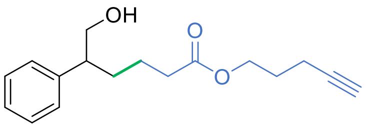

Following the general procedure **5B** compound **36** was obtained from 3-phenyloxetane (**1**) (27 mg, 0.20 mmol) and pent-4-yn-1-yl acrylate (41 mg, 0.30 mmol). The crude product was purified by column chromatography (10:90 AcOEt/Hexane) to afford 40 mg of pent-4-yn-1-yl 6-hydroxy-5-phenylhexanoate (**36**) as colorless oil, (yield = **73%**).

^{1}H NMR (500 MHz, CDCl$_3$): δ 7.34 – 7.31 (m, 2H), 7.25 – 7.22 (m, 3H), 4.14 (t, *J* = 5.9 Hz, 2H), 3.77 – 3.69 (m, 2H), 2.81 – 2.75 (m, 1H), 2.29 – 2.24 (m, 4H), 1.96 – 1.95 (m, 1H), 1.85 – 1.80 (m, 2H), 1.76 – 1.73 (m, 1H), 1.66 – 1.49 (m, 4H).

^{13}C NMR (125 MHz, CDCl$_3$): δ 173.4, 141.9, 128.7, 128.0, 126.9, 83.0, 69.0, 67.4, 62.8, 48.4, 34.1, 31.4, 27.5, 22.7, 15.2.

HRMS (ESI) $[M+Na]^+$ calculated for $C_{17}H_{22}O_3Na$: 297.1467, found: 297.1480.

(1*R*,2*S*,5*R*)-2-isopropyl-5-methylcyclohexyl 6-hydroxy-5-phenylhexanoate (37)

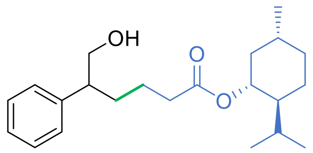

Following the general procedure **5B** compound **37** was obtained from 3-phenyloxetane (**1**) (27 mg, 0.20 mmol) and (-)-methol acrylate (63 mg, 0.30 mmol). The crude product was purified by column chromatography (10:90 AcOEt/Hexane) to afford 18 mg of (1*R*,2*S*,5*R*)-2-isopropyl-5-methylcyclohexyl 6-hydroxy-5-phenylhexanoate (**37**) as colorless oil (a *mixture of diastereoisomers*), (yield **= 26%**).

^{1}H NMR (500 MHz, CDCl$_3$): δ 7.34 – 7.31 (m, 2H), 7.26 – 7.20 (m, 3H), 4.65 (td, *J* = 10.9, 4.4 Hz, 1H), 3.76 – 3.73 (m, 2H), 2.80 – 2.77 (m, 1H), 2.25 (t, *J* = 7.2 Hz, 2H), 1.96 – 1.94 (m, 1H), 1.81 (m, 1H), 1.76 – 1.71 (m, 1H), 1.68 – 1.45 (m, 6H), 1.41 – 1.30 (m, 2H), 1.08 – 1.00 (m, 1H), 0.96 – 0.83 (m, 8H), 0.73 (d, *J* = 7.0 Hz, 3H).
^{13}C NMR (125 MHz, CDCl$_3$): δ 128.7, 128.00, 127.99, 126.8, 74.1, 74.04, 67.39, 67.35, 48.5, 48.4, 47.00, 41.0, 34.6, 34.5, 34.3, 31.44, 31.36, 26.3, 23.4, 22.92, 22.90, 22.0, 20.7, 16.3.
HRMS (ESI) $[M+Na]^+$ calculated for $C_{22}H_{34}O_3Na$: 369.2406, found: 369.2392.

(8*R*,9*S*,13*S*,14*S*)-13-methyl-17-oxo-7,8,9,11,12,13,14,15,16,17-decahydro-6*H*-cyclopenta[a]phenanthren-3-yl 6-hydroxy-5-phenylhexanoate (38)

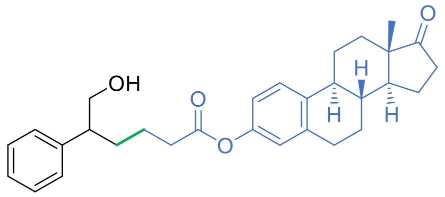

Following the general procedure **5B** compound **38** was obtained from 3-phenyloxetane (**1**) (27 mg, 0.20 mmol) and (8*R*,9*S*,13*S*,14*S*)-13-methyl-17-oxo-7,8,9,11,12,13,14,15,16,17-decahydro-6*H*-cyclopenta[a]phenanthren-3-yl acrylate (97 mg, 0.30 mmol). The crude product was purified by column chromatography (25:75 AcOEt/Hexane) to afford 26 mg of (8*R*,9*S*,13*S*,14*S*)-13-methyl-17-oxo-7,8,9,11,12,13,14,15,16,17-decahydro-6*H*-cyclopenta[a]phenanthren-3-yl 6-hydroxy-5-phenylhexanoate (**38**) as colorless oil, (yield **= 58%**).

^{1}H NMR (500 MHz, CDCl$_3$): δ 7.35 – 7.32 (m, 2H), 7.29 – 7.20 (m, 4H), 6.81 (dd, *J* = 8.5, 2.6 Hz, 1H), 6.77 (d, *J* = 2.5 Hz, 1H), 3.76 – 3.74 (m, 2H), 2.94 – 2.86 (m, 2H), 2.86 – 2.80 (m, 1H), 2.55 – 2.44 (m, 3H), 2.41 – 2.38 (m, 1H), 2.29 – 2.27 (m, 1H), 2.19 – 1.93 (m, 4H), 1.91 – 1.82 (m, 1H), 1.77 – 1.42 (m, 10H), 0.91 (s, 3H).
^{13}C NMR (125 MHz, CDCl$_3$): δ 220.7, 172.1, 148.5, 141.8, 137.9, 137.3, 128.7, 128.0, 126.9, 126.3, 121.5, 118.7, 67.4, 50.4, 48.4, 47.9, 44.1, 38.0, 35.8, 34.2, 31.5, 31.3, 29.3, 26.3, 25.7, 22.8, 21.6, 13.8.
HRMS (ESI) $[M+Na]^+$ calculated for $C_{30}H_{36}O_4Na$: 483.2511, found: 483.2510.

7. Mechanistic consideration

7.1 Proposed mechanism

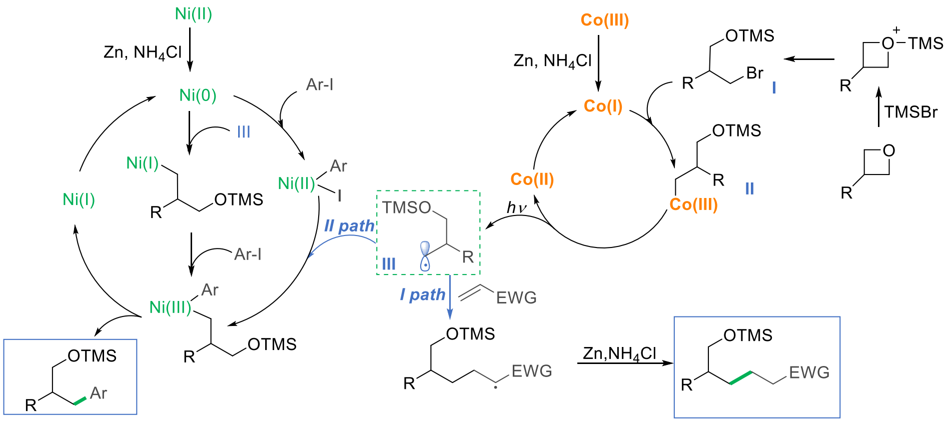

7.2 Mass spectrometry studies

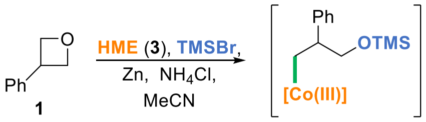

Reaction conditions: oxetane (**1**) (0.2 mmol), Zn (3 equiv), NH_4Cl (1.5 equiv), HME (**3**) (5 mol%), TMSBr (2 equiv), $MeCN_{anh}$. (c = 0.1 M), white LEDs, 16 h.

The reaction was setup according to general procedure **5B** (without the addition of methyl acrylate). After 15 minutes of light irradiation an aliquot was taken from the reaction mixture and was studied by HRMS.

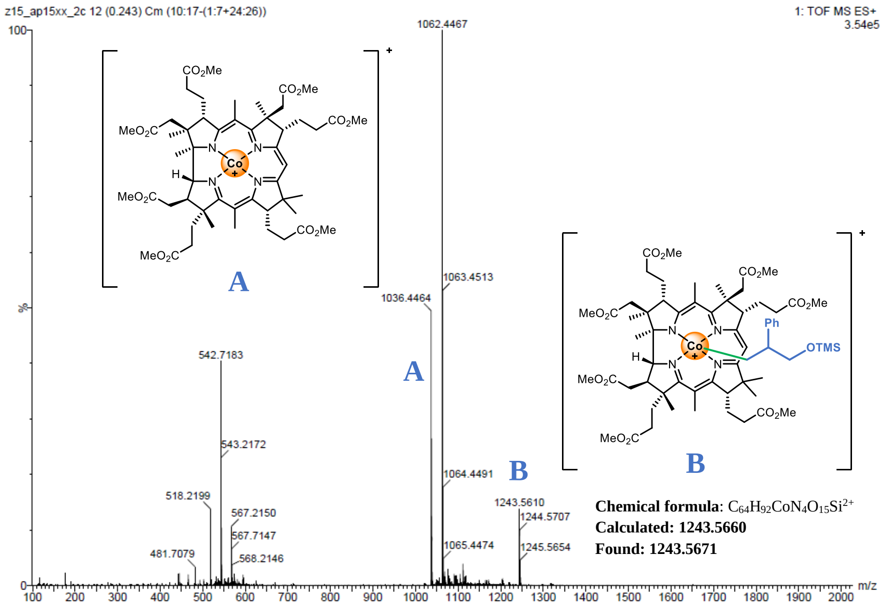

The HRMS spectrum of the reaction mixture indicates the presence of the catalyst and alkyl-cobalamin derivative: **A** and **B**. The first signal (**A**) corresponds to a catalyst without its axial ligands (H_2O and CN^-). The second signal (**B**) corresponds to the mass of the Co-alkyl complex.

<u>Conclusion</u>: *Co-alkyl complex **B** is an intermediate in the reaction via the nucleophilic attack of the Co(I) form of the catalyst on the bromohydrin.*

7.3 Kinetic studies for the model reaction

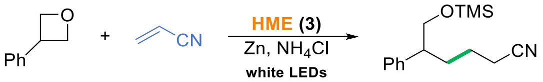

1 **39** **S15**

Reaction conditions: oxetane (**1**) (0.4 mmol), acrylonitrile (**39**) (1.5 equiv), Zn (3 equiv), NH_4Cl (1.5 equiv), HME (**3**) (5 mol%), $MeCN_{anh.}$ (c = 0.1M), white LEDs, 16h.

The reaction was setup according to general procedure **5B** on a 0.4 mmol scale with the addition of dodecane as an internal standard (65.6 mg). The reaction was monitored by GC/FID for 16h.

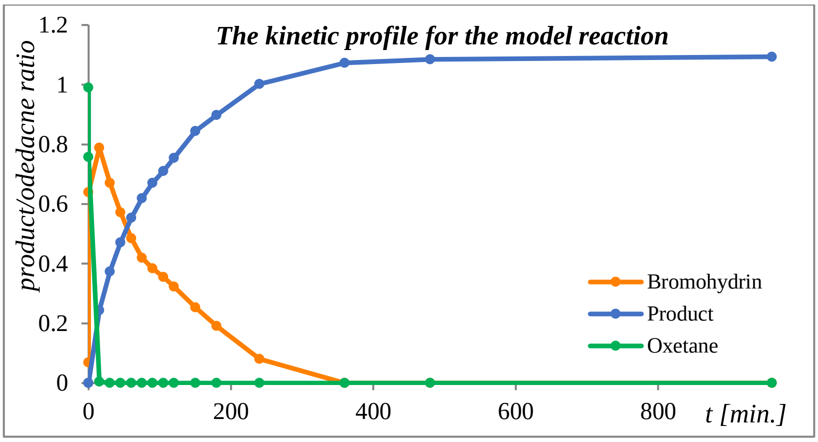

To prove the formation of bromohydrin during the reaction, another kinetics experiment was performed:

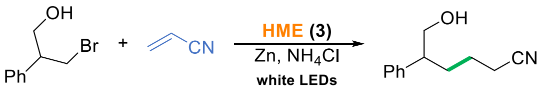

5b **39** **22a**

Reaction conditions: bromohydrin (**5b**) (0.2 mmol), acrylonitrile (**39**) (1.5 equiv), Zn (3 equiv), NH_4Cl (1.5 equiv), HME (**3**) (5 mol%), $MeCN_{anh.}$ (c = 0.1M), white LEDs, 16h.

The reaction was setup according to general procedure **5B** on 0.2 mmol scale with the addition of dodecane as an internal standard (31.0 mg). The reaction was monitored by GC/FID for 16h.

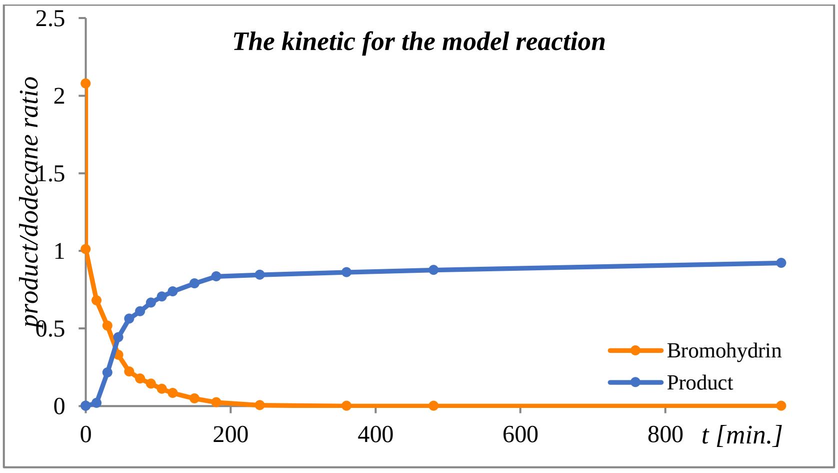

<u>Conclusion</u>: *The kinetic experiments show that oxetane* ***1*** *is fully converted into bromohydrin within 15 min, while the product gradually forms over 10h.*

7.4 Reactions with deuterated reagents

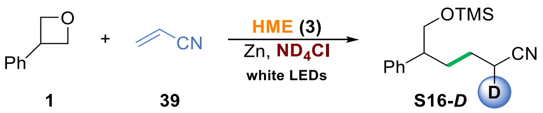

Reaction conditions: oxetane (**1**) (0.2 mmol), acrylonitrile (**39**) (1.5 equiv), Zn (3 equiv), ND_4Cl (1.5 equiv), HME (**3**) (5 mol%), TMSBr (2 equiv), MeCN (c = 0.1M), white LEDs, 16h.

Reaction was setup according to general procedure **5B** on a 0.2 mmol scale using ND_4Cl (ND_4Cl contained 98 atom% of deuterium). After 16 h of light irradiation a small portion was taken from reaction mixture and ^{1}H NMR spectrum was recorded.

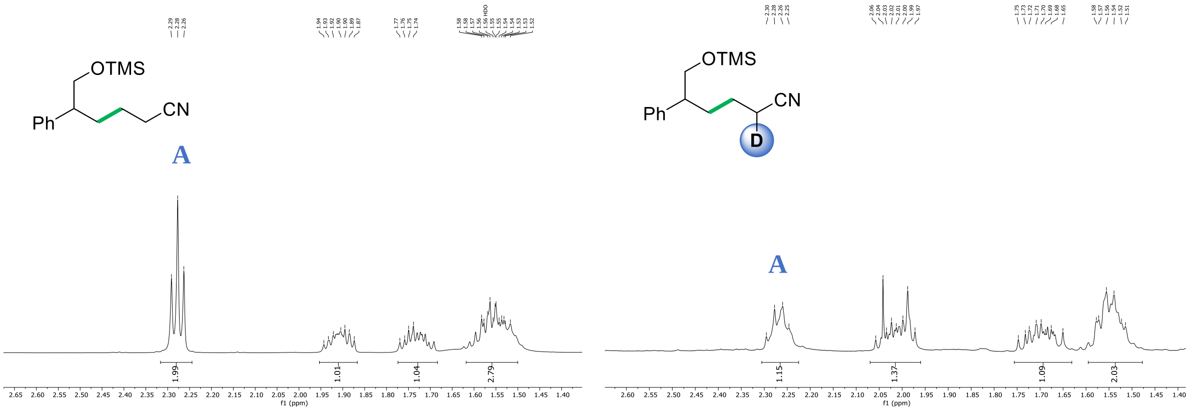

Figure S2 Left) ^{1}H NMR fragment of the spectra of product **41**; **Right)** ^{1}H NMR spectra of a mixture of products formed in the deuterium incorporation experiment.

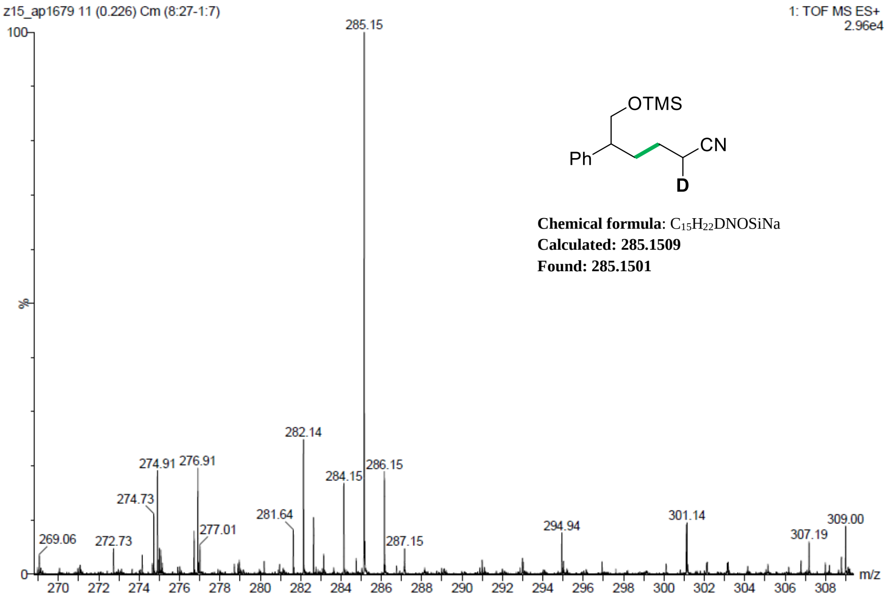

Conclusion: *The integration 1.15 ppm for signal **A** (Scheme 7.4.1) in ^{1}H NMR indicates 85%-D incorporation at the α position to the electron-withdrawing group.*

7.5 Experiment with a radical trap

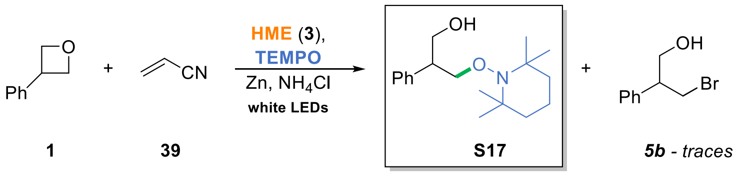

1 39 S17 *5b - traces*

Reaction conditions: oxetane (**1**) (0.2 mmol), acrylonitrile (**39**) (1.5 equiv), Zn (3 equiv), NH_4Cl (1.5 equiv), HME (**3**) (5 mol%), TMSBr (2 equiv), TEMPO (3 equiv), $MeCN_{anh.}$ (c = 0.1M), white LEDs, 16h, reaction was quenched by treatment with 2 equiv of citric acid.

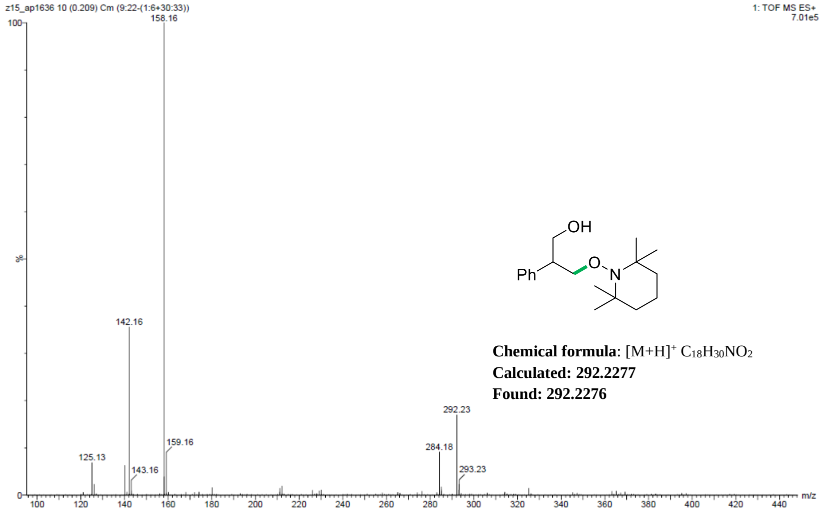

Figure S3 HRMS ESI analysis of the product obtained in the radical trap experiment.

Conclusion: *Product **40** formed in the reaction of an alkyl radical with radical trap (TEMPO). This result proves the hypothesis that reaction involves the formation of radicals as intermediates.*

7.6 DFT calculations

Computational methods

All the calculations were performed with Gaussian 16 package.[18] Geometry optimizations were computed at BP86/6-31G(d) level of theory with the D3 version of Grimme's empirical dispersion correction[19] and SMD model of solvation (acetonitrile).[20] Frequency analysis was performed at the same level to provide correction to thermodynamic functions and confirm the nature of optimized structures (minima and transition states featured zero or one imaginary frequency, respectively). Single point energies were computed at BP86/6-311++G(2df,p) level of theory with the D3 version of Grimme's empirical dispersion correction and SMD model of solvation (acetonitrile). We performed calculations approximating the structure of vitamin B_{12} with Co-corrin complex bearing 15 methyl groups, reflecting substitution pattern at a rim. Molecular structures were visualized in CYLview.[21]

Optimized geometries, energies and corrections to thermodynamic functions.

$(CH_3)_{15}$(corrin)Co(I)

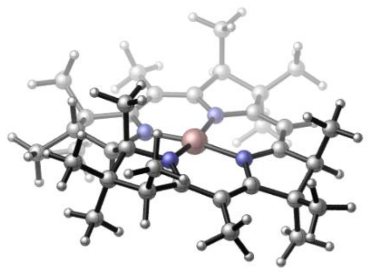

E (BP86-D3/6-31G(d)/SMD (MeCN)) = -2928.476207
E (BP86-D3/6-311++G(2df,p)/SMD (MeCN)// BP86-D3/6-31G(d))/SMD (MeCN)) = -2929.055031

Zero-point correction=	0.777035
Thermal correction to Energy=	0.816636
Thermal correction to Enthalpy=	0.817580
Thermal correction to Gibbs Free Energy=	0.712422

Charge = 0 Multiplicity = 1

C	2.89754400	-0.89013600	-0.29398400
C	4.32154900	-0.37444100	-0.60113500
C	4.26251700	1.03989500	0.04830800
C	2.78793000	1.34632300	-0.03760400
N	2.02450300	0.20488000	-0.10706500
H	4.86289700	1.76141400	-0.53748600
C	2.28356300	2.64114000	0.00498000
H	3.01207600	3.45778500	0.03219800
C	2.49413600	-2.21994600	-0.27583900
C	0.93415800	2.97238200	0.00248900
C	0.41910600	4.40302100	-0.09404600
N	-0.08337700	2.04358600	-0.00741500
C	-1.04165600	4.21004000	0.39588100
C	-1.30149000	2.74240200	0.06751400
C	1.13851500	-2.55535300	0.03153500
C	0.63021800	-3.96442100	0.29474500
N	0.17694600	-1.63450800	0.13908300
C	-0.92264700	-3.79969000	0.12573100
C	-1.13152600	-2.28213300	0.49434700
C	-2.57044200	2.18974200	-0.05150700
C	-2.73277500	0.77390300	-0.18785700
C	-4.05137400	0.00952000	-0.41850700
N	-1.67218200	-0.04465900	-0.18297900
C	-3.61787500	-1.44601100	-0.03094100
C	-2.11969200	-1.45814400	-0.35745400
Co	0.13362200	0.17300800	-0.04798000
H	-1.71270500	4.88677700	-0.16144700
H	-3.73311700	-1.51520500	1.06756000
H	0.98688200	-4.65267600	-0.49472900
H	-1.96696200	-1.71458900	-1.42140600
C	-3.78893700	3.09594100	-0.00058800
H	-4.51260000	2.84733200	-0.79522100
H	-4.32787800	3.02279100	0.96207300
H	-3.51816800	4.15315800	-0.13967700
C	-4.43499000	0.12620800	-1.91517300

H	-4.59473700	1.18281300	-2.19319500
H	-3.64244400	-0.27917700	-2.56997000
H	-5.37165400	-0.42128600	-2.12662800
C	-5.24706200	0.41866900	0.46843200
H	-5.76132200	1.32426400	0.11089700
H	-5.99325200	-0.39795700	0.46585900
H	-4.93279400	0.58478000	1.51453100
C	-4.44330000	-2.56270800	-0.67546700
H	-4.18319200	-3.54524200	-0.24688700
H	-5.52393500	-2.40586200	-0.50259000
H	-4.27887900	-2.61726600	-1.76603600
C	-1.38418300	-2.04237000	1.99797800
H	-2.32782100	-2.49966400	2.33861300
H	-0.56217400	-2.45562900	2.60334000
H	-1.42559700	-0.95557300	2.18614400
C	-1.73741900	-4.77514800	0.99036600
H	-2.82074600	-4.60186000	0.86479600
H	-1.52977000	-5.81697500	0.68372700
H	-1.50718700	-4.69174400	2.06480600
C	-1.23666100	-4.08315800	-1.36272200
H	-0.96593000	-5.12829500	-1.60087600
H	-2.30651700	-3.95686200	-1.59660100
H	-0.65542400	-3.42624000	-2.03584700
C	1.15320000	-4.52890400	1.63383800
H	0.77695500	-5.55303100	1.80500600
H	2.25635100	-4.57717000	1.61692000
H	0.86198700	-3.90447800	2.49493500
C	3.45455500	-3.35380800	-0.60036900
H	4.08338500	-3.11545400	-1.47370800
H	4.13307100	-3.60137200	0.23627300
H	2.90590000	-4.27487000	-0.85245400
C	5.51996100	-1.18744300	-0.07342800
H	5.73634900	-2.06987800	-0.69411300
H	6.42273200	-0.54938400	-0.09866600
H	5.37169800	-1.52537600	0.96623300
C	4.44598200	-0.21164200	-2.14020700
H	3.66182100	0.46143200	-2.53305900
H	5.43122100	0.21921000	-2.40072500
H	4.35025400	-1.18585500	-2.65155200
C	4.71232800	1.10853700	1.52603000
H	5.79099700	0.90176600	1.63937400
H	4.51624400	2.11854500	1.92823500
H	4.15164300	0.38263600	2.14333100
C	-1.25803500	4.44049000	1.90767300
H	-1.06176100	5.49049400	2.18832300
H	-2.30011600	4.20314400	2.18492700
H	-0.59196200	3.79098200	2.50476700
C	0.40517600	4.81255300	-1.59166000
H	-0.00858400	5.83200200	-1.70989500
H	1.42836900	4.80205200	-2.00873400
H	-0.21664700	4.11822900	-2.18660100
C	1.24219600	5.42830300	0.70249600
H	2.24270500	5.56174000	0.25290700
H	0.74306100	6.41455300	0.68979000
H	1.38283500	5.12309600	1.75350600

Styrene oxide

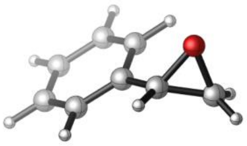

E (BP86-D3/6-31G(d)/SMD (MeCN)) = -384.864461
E (BP86-D3/6-311++G(2df,p)/SMD (MeCN)// BP86-D3/6-31G(d))/SMD (MeCN)) = -384.988582

Zero-point correction=	0.134929
Thermal correction to Energy=	0.142323
Thermal correction to Enthalpy=	0.143268
Thermal correction to Gibbs Free Energy=	0.102709

Charge = 0 Multiplicity = 1

C	2.59533100	-0.05141700	0.73801600
C	1.60678600	0.61063100	-0.15115500
H	1.84491700	1.62749300	-0.49995400
O	2.50307600	-0.41835300	-0.65813500
C	0.15006900	0.27965900	-0.09177900
C	-0.80234000	1.31743200	-0.04058400
C	-0.28915200	-1.06059200	-0.06860100
C	-2.17236100	1.02058000	0.05100700
H	-0.46668500	2.36132300	-0.07096800
C	-1.65774100	-1.35614500	0.01805800
H	0.45248500	-1.86447300	-0.13561600
C	-2.60404800	-0.31668400	0.08179000
H	-2.90347300	1.83613700	0.09201200
H	-1.98839300	-2.40108900	0.03171400
H	-3.67300400	-0.54875600	0.14830600
H	2.23992900	-0.81139600	1.45028900
H	3.51035600	0.48680400	1.02878200

TS1

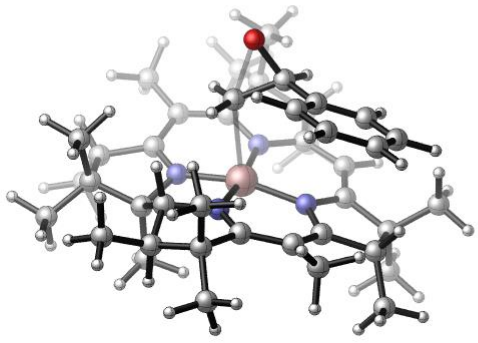

E (BP86-D3/6-31G(d)/SMD (MeCN)) =
E (BP86-D3/6-311++G(2df,p)/SMD (MeCN)// BP86-D3/6-31G(d))/SMD (MeCN)) =

Zero-point correction=	0.912264

Thermal correction to Energy= 0.960310
Thermal correction to Enthalpy= 0.961254
Thermal correction to Gibbs Free Energy= 0.838326

Charge = 0 Multiplicity = 1

C	2.66648100	-1.87273800	0.21649600
C	2.93247300	-3.33591400	0.63178100
C	1.82334700	-4.07955900	-0.17568100
C	0.79523700	-2.98895800	-0.36595800
N	1.33159100	-1.73859200	-0.21300800
H	1.41448500	-4.92054200	0.41546700
C	-0.50758900	-3.24335800	-0.78474000
H	-0.77585500	-4.29013600	-0.96091100
C	3.55881000	-0.81049800	0.31899300
C	-1.47303300	-2.27721300	-1.05262800
C	-2.80136200	-2.60621900	-1.71921300
N	-1.30412100	-0.93721000	-0.81335300
C	-3.58924500	-1.26721800	-1.53166400
C	-2.52940000	-0.29009400	-1.02157800
C	3.16863300	0.50699100	-0.09387100
C	4.11489300	1.68416600	-0.26696300
N	1.90932100	0.82428500	-0.38327400
C	3.13179800	2.91062900	-0.26049600
C	1.79469000	2.26524400	-0.79289600
C	-2.78525900	1.04573200	-0.72721100
C	-1.70930300	1.94829600	-0.44694400
C	-1.80639700	3.47471300	-0.24320800
N	-0.44824900	1.52027100	-0.34810400
C	-0.32853900	3.89655000	-0.56238700
C	0.46971400	2.66947900	-0.10900900
Co	0.35016600	-0.12122500	-0.37107000
H	-4.32496700	-1.41977100	-0.71609200
H	-0.26163900	3.96621500	-1.66531200
H	4.77716800	1.77524900	0.61418900
H	0.64370900	2.72699400	0.98084500
C	0.50315200	-0.02962000	2.05863300
C	-0.46255700	-1.00142800	2.62253300
H	-0.25795200	-2.05745800	2.33699600
O	0.19022500	-0.52529700	3.76603000
C	-1.94287100	-0.70624400	2.52123200
C	-2.84265300	-1.67841100	2.04986300
C	-2.44686400	0.54013100	2.94371700
C	-4.22094500	-1.41068200	1.98631500
H	-2.45362000	-2.64912900	1.71905700
C	-3.82009000	0.81719900	2.87305700
H	-1.75015400	1.28445500	3.34529200
C	-4.71348200	-0.15923000	2.39302800
H	-4.90965100	-2.18017100	1.61848000
H	-4.19754700	1.79413500	3.19665900
H	-5.78707000	0.05470300	2.34059600
H	0.25270600	1.03073900	2.11130700
H	1.55327200	-0.31499200	1.98922700
C	-4.22031800	1.54228700	-0.67853600
H	-4.34722600	2.27953400	0.12990200
H	-4.55728400	2.02348400	-1.61500800
H	-4.91554100	0.71782800	-0.45349700
C	-2.18224100	3.77408200	1.22920800
H	-3.16449300	3.33912200	1.47907100
H	-1.44274700	3.35139200	1.93240300
H	-2.24233700	4.86397000	1.40216700

C	-2.75932000	4.22058200	-1.19993400
H	-3.81528600	4.15367000	-0.89452100
H	-2.49431600	5.29429600	-1.21209700
H	-2.66989200	3.83830100	-2.23272600
C	0.11330000	5.23124500	0.04149400
H	1.10854300	5.52338000	-0.33340100
H	-0.58975200	6.04053300	-0.22838000
H	0.16993400	5.18434700	1.14311800
C	1.64829700	2.32022300	-2.32707900
H	1.57413700	3.35749300	-2.69350800
H	2.50684700	1.83873400	-2.82123600
H	0.73694500	1.77296600	-2.62505000
C	3.63955300	4.09610600	-1.09632300
H	2.91502500	4.92908900	-1.08297400
H	4.58860200	4.47560200	-0.67473200
H	3.82538400	3.83004500	-2.14943700
C	3.00319200	3.36364600	1.21316800
H	3.99037100	3.69924200	1.58053700
H	2.30137400	4.20582300	1.33106600
H	2.66626800	2.53758700	1.86684100
C	5.03621800	1.50347000	-1.49283100
H	5.72024400	2.36320900	-1.60293600
H	5.65530500	0.59748000	-1.36735500
H	4.46747600	1.39029200	-2.43102100
C	4.96403200	-0.99408500	0.87006400
H	4.96449400	-1.61762300	1.77873400
H	5.65396500	-1.46412800	0.14598200
H	5.40561600	-0.02630400	1.15403300
C	4.32852000	-3.92753200	0.35317700
H	5.07778100	-3.59624000	1.08746100
H	4.27136900	-5.02887700	0.42960700
H	4.69802300	-3.67493300	-0.65507800
C	2.62071200	-3.45454600	2.14807600
H	1.58791400	-3.13381800	2.37162500
H	2.73490100	-4.50411100	2.47857900
H	3.30759200	-2.82840900	2.74447300
C	2.25724500	-4.61633800	-1.55934700
H	3.00178600	-5.42651400	-1.47160800
H	1.38009700	-5.01986800	-2.09564600
H	2.69069100	-3.80900900	-2.17770600
C	-4.36594800	-0.81182300	-2.78192200
H	-5.01080800	-1.63270100	-3.14427800
H	-5.02204800	0.04562600	-2.57099200
H	-3.68584900	-0.52107200	-3.60097800
C	-3.55029000	-3.78941200	-1.07613400
H	-4.54032100	-3.90885400	-1.55506600
H	-3.00156900	-4.73998600	-1.20502700
H	-3.70963000	-3.62622100	0.00361400
C	-2.49348400	-2.95176600	-3.20065600
H	-1.84711500	-3.84666300	-3.24278200
H	-3.41593500	-3.17381300	-3.76628700
H	-1.96214500	-2.12731500	-3.70859400

Oxetane

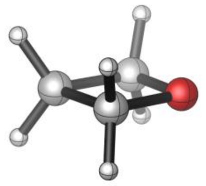

E (BP86-D3/6-31G(d)/SMD (MeCN)) = -193.111200
E (BP86-D3/6-311++G(2df,p)/SMD (MeCN)// BP86-D3/6-31G(d))/SMD (MeCN)) = -193.180285

Zero-point correction=	0.084291
Thermal correction to Energy=	0.088597
Thermal correction to Enthalpy=	0.089542
Thermal correction to Gibbs Free Energy=	0.056243

Charge = 0 Multiplicity = 1

C	-0.00008000	1.08145900	-0.00020500
C	-1.04163700	-0.05973800	0.00020000
C	1.04163900	-0.05959400	0.00020600
H	-0.00010100	1.71644700	-0.90022000
H	-0.00013500	1.71731200	0.89919900
H	-1.68501800	-0.12539100	-0.90018700
H	-1.68406300	-0.12530500	0.90129100
H	1.68500800	-0.12514600	-0.90020000
H	1.68407500	-0.12508100	0.90128800
O	0.00008800	-1.08819900	-0.00029700

TS2

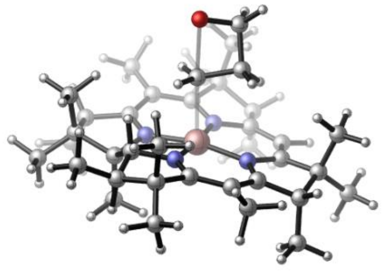

E (BP86-D3/6-31G(d)/SMD (MeCN)) = -3121.572096
E (BP86-D3/6-311++G(2df,p)/SMD (MeCN)// BP86-D3/6-31G(d))/SMD (MeCN)) = -3122.22262

Zero-point correction=	0.860382
Thermal correction to Energy=	0.905161
Thermal correction to Enthalpy=	0.906106
Thermal correction to Gibbs Free Energy=	0.790597

Charge = 0 Multiplicity = 1

C	-2.72425500	-1.21924800	-0.09687800
C	-4.20670400	-0.84451900	0.10768200
C	-4.26477900	0.51660200	-0.65130600
C	-2.83997500	0.99132500	-0.51214000
N	-1.97121500	-0.04824600	-0.31777800
H	-4.97000800	1.20899200	-0.15531700
C	-2.47436100	2.33206100	-0.58911000

H	-3.28206500	3.05969700	-0.71239900
C	-2.17469300	-2.49076200	-0.00625100
C	-1.17428800	2.81452800	-0.48433100
C	-0.83933200	4.29763400	-0.39080300
N	-0.06498000	2.01668100	-0.33927700
C	0.68031900	4.26863700	-0.70950600
C	1.06801500	2.84181000	-0.33074000
C	-0.78168800	-2.69285100	-0.28778400
C	-0.13145500	-4.04600600	-0.52317400
N	0.07852300	-1.68928800	-0.40601000
C	1.39716500	-3.71659200	-0.36011100
C	1.44982000	-2.19035200	-0.75531800
C	2.37819800	2.43001900	-0.12027500
C	2.69490400	1.03536400	-0.01672100
C	4.08991900	0.41755500	0.20828800
N	1.74521400	0.10056000	-0.07351500
C	3.82843900	-1.06200200	-0.24142700
C	2.34395500	-1.25449500	0.08591100
Co	-0.08689500	0.12441600	-0.20017700
H	1.20514800	5.01775500	-0.09182200
H	3.94507300	-1.07124500	-1.34200700
H	-0.41765100	-4.74888600	0.28128500
H	2.23184500	-1.52775500	1.15110500
C	-0.27068900	-0.21480600	2.07799200
C	-0.79869500	1.14681300	2.54276600
H	-1.58585100	1.56415300	1.88980200
C	3.48404700	3.46998300	-0.06243300
H	4.20634700	3.24490200	0.73905900
H	4.05560100	3.53393500	-1.00668300
H	3.08543900	4.47458000	0.14504900
C	4.42972700	0.51274700	1.71719900
H	4.46606200	1.56625000	2.04478200
H	3.67700800	-0.00712900	2.33713300
H	5.41794600	0.06348400	1.92348200
C	5.24068200	0.99878300	-0.63955300
H	5.64358600	1.93983400	-0.23426000
H	6.07461200	0.27273400	-0.65959000
H	4.92166600	1.17476900	-1.68249900
C	4.77742300	-2.10080000	0.36042900
H	4.62968700	-3.08843300	-0.10746300
H	5.83243200	-1.81479800	0.19583100
H	4.62263100	-2.21644300	1.44742100
C	1.67793400	-1.94625500	-2.26122100
H	2.66306800	-2.31553400	-2.59064200
H	0.90277400	-2.44738700	-2.86178500
H	1.61927900	-0.86355600	-2.46960500
C	2.30361600	-4.62161900	-1.20854600
H	3.36543800	-4.34768800	-1.08172400
H	2.19190300	-5.67353700	-0.88735500
H	2.07018100	-4.57439100	-2.28445700
C	1.73649900	-3.93346100	1.13358900
H	1.56892100	-4.99407500	1.39609200
H	2.78924900	-3.69780900	1.36091500
H	1.09385600	-3.32011700	1.79237800
C	-0.59728400	-4.68383100	-1.85007600
H	-0.11415400	-5.66424900	-2.00543600
H	-1.68870000	-4.84958200	-1.82538200
H	-0.37688000	-4.04611700	-2.72245000
C	-3.00779600	-3.70247700	0.37696400
H	-3.69920700	-3.47044600	1.20262400

H	-3.61136000	-4.09683500	-0.46059000
H	-2.36409600	-4.52457400	0.72751200
C	-5.27913400	-1.82510300	-0.40258900
H	-5.42580600	-2.67764100	0.27725700
H	-6.24660100	-1.29368500	-0.46493900
H	-5.04193700	-2.21943600	-1.40517000
C	-4.41572000	-0.57995500	1.62322700
H	-3.72410400	0.20020800	1.99048600
H	-5.45165700	-0.24062900	1.81059500
H	-4.23970000	-1.49625800	2.21365900
C	-4.63026700	0.42296000	-2.15016300
H	-5.67143300	0.08693400	-2.29649400
H	-4.52338000	1.41558800	-2.62281300
H	-3.95918100	-0.27959900	-2.67750800
C	1.03923300	4.51448600	-2.19171700
H	0.75498500	5.53301100	-2.50918500
H	2.12596100	4.40066000	-2.34746600
H	0.52410300	3.78887700	-2.84737300
C	-1.04378300	4.73634400	1.08459600
H	-0.77215600	5.80167300	1.20670600
H	-2.09812300	4.60752500	1.38824300
H	-0.41366600	4.13864100	1.76783000
C	-1.68185800	5.19718000	-1.30925800
H	-2.73403400	5.22264400	-0.97363900
H	-1.30303500	6.23489400	-1.27928400
H	-1.66881400	4.85177800	-2.35702200
H	0.03746000	1.86947100	2.56177900
H	-0.97059100	-1.04396300	1.95139400
H	0.78481300	-0.45911800	2.17221200
C	-1.22978500	0.70004100	3.94191000
H	-1.13186500	1.49431000	4.73427600
H	-2.33622900	0.45017900	3.92365200
O	-0.39644100	-0.41026900	4.12948600

TMSBr

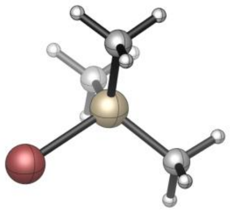

E (BP86-D3/6-31G(d)/SMD (MeCN)) = -2981.298478
E (BP86-D3/6-311++G(2df,p)/SMD (MeCN)// BP86-D3/6-31G(d))/SMD (MeCN)) = -2983.804338

Zero-point correction=	0.109642
Thermal correction to Energy=	0.118528
Thermal correction to Enthalpy=	0.119472
Thermal correction to Gibbs Free Energy=	0.075902

Charge = 0 Multiplicity = 1

Si	0.83574600	-0.00003600	-0.00008600
C	1.39015300	1.03230800	-1.47175900
H	2.49629700	1.05747800	-1.50605100
H	1.02739200	0.60311000	-2.42256300
H	1.02582400	2.07194800	-1.39274900
C	1.39066200	0.75834300	1.62973400
H	1.02780900	0.16953200	2.49085900

H	2.49683700	0.77664000	1.66772500
H	1.02703300	1.79598800	1.73420900
C	1.39013900	-1.79076500	-0.15823300
H	2.49628200	-1.83318100	-0.16151400
H	1.02641900	-2.39995400	0.68816700
H	1.02693400	-2.24185900	-1.09867400
Br	-1.43934300	0.00004300	0.00009500

Br(CH$_2$)$_3$OTMS

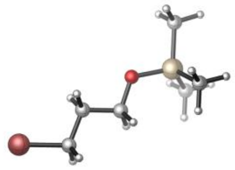

E (BP86-D3/6-31G(d)/SMD (MeCN)) = -3174.442469
E (BP86-D3/6-311++G(2df,p)/SMD (MeCN)// BP86-D3/6-31G(d))/SMD (MeCN)) = -3177.023972

Zero-point correction=	0.196034
Thermal correction to Energy=	0.210523
Thermal correction to Enthalpy=	0.211467
Thermal correction to Gibbs Free Energy=	0.151908

Charge = 0 Multiplicity = 1

C	0.93136100	-0.24491100	0.00027200
C	-0.42712700	0.47061300	-0.00001700
C	2.07259700	0.76248800	0.00022500
H	0.99508200	-0.89357300	0.89249100
H	0.99530700	-0.89397500	-0.89164200
H	-0.51017400	1.12197300	-0.89586100
H	-0.51008500	1.12297300	0.89511500
H	2.08082600	1.39643100	0.89997700
H	2.08058300	1.39668600	-0.89935700
O	-1.45593200	-0.52961400	0.00058300
Si	-3.09280600	-0.07269700	-0.00007800
C	-3.48576700	0.93849900	1.54725000
H	-2.92745700	1.89255600	1.56812800
H	-4.56417900	1.18369400	1.58395500
H	-3.23556300	0.37674600	2.46625000
C	-4.03905100	-1.70086200	0.00097200
H	-5.12909600	-1.51327100	-0.00001200
H	-3.79993200	-2.30576800	-0.89286400
H	-3.80120500	-2.30376700	0.89650100
C	-3.48572800	0.93650200	-1.54868400
H	-3.23495200	0.37413800	-2.46714400
H	-4.56438100	1.18065100	-1.58569200
H	-2.92832600	1.89106500	-1.57034400
Br	3.84578800	-0.16416800	-0.00009100

Co1b_BrPrOTMS1_ts1a

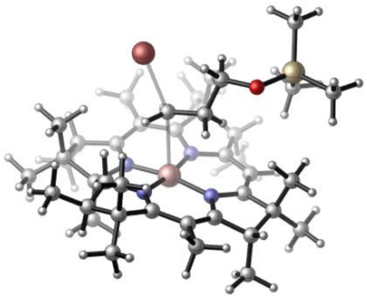

E (BP86-D3/6-31G(d)/SMD (MeCN)) = -6102.942436
E (BP86-D3/6-311++G(2df,p)/SMD (MeCN)// BP86-D3/6-31G(d))/SMD (MeCN)) = -6106.094499

Zero-point correction=	0.974500
Thermal correction to Energy=	1.029556
Thermal correction to Enthalpy=	1.030500
Thermal correction to Gibbs Free Energy=	0.891014

Charge = 0 Multiplicity = 1

C	-0.03532700	-2.51440400	-1.50012100
C	-1.31606300	-3.34534800	-1.72107500
C	-2.16924600	-2.35258000	-2.56659300
C	-1.60628100	-1.02459500	-2.12463800
N	-0.34921800	-1.14153500	-1.59409200
H	-3.23970200	-2.43751100	-2.29900900
C	-2.30013600	0.17246200	-2.27499800
H	-3.29588000	0.11360400	-2.72495900
C	1.20716900	-3.00617100	-1.12041200
C	-1.84811700	1.42086000	-1.86129800
C	-2.71680700	2.67243700	-1.89949400
N	-0.64181900	1.63159000	-1.24012900
C	-1.65445100	3.77298400	-1.62775400
C	-0.54323600	2.99546200	-0.93051000
C	2.28638500	-2.10822800	-0.83048900
C	3.72222000	-2.53338300	-0.57228500
N	2.12258900	-0.79097800	-0.74138100
C	4.31216400	-1.27993800	0.16587400
C	3.42224400	-0.11282000	-0.40918800
C	0.45659400	3.59096900	-0.17101900
C	1.58719800	2.82909700	0.26893400
C	2.75519700	3.32665600	1.14351700
N	1.70586400	1.53039700	-0.02240500
C	3.82512700	2.21674400	0.85802400
C	2.96023200	0.98533000	0.57056200
Co	0.66186200	0.29985500	-0.88716800
H	-2.08654700	4.55165500	-0.97571100
H	4.33131700	2.51428000	-0.08018100
H	3.74521100	-3.38252100	0.13555900
H	2.68570800	0.50580900	1.52696500
C	-0.01911700	-0.73015300	1.39691600
C	-1.34385900	0.00023100	1.42406600
H	-1.87273200	-0.18408700	0.47347900
H	0.87356600	-0.21827900	1.74183800
H	0.11451700	-1.60907000	0.77747600
C	0.36427700	5.07272100	0.15095200
H	0.62830500	5.27308700	1.20240700

H	1.03827600	5.68494600	-0.47607900
H	-0.65564300	5.45818900	0.00273000
C	2.29061900	3.32224100	2.62203200
H	1.44047300	4.01228200	2.76363800
H	1.96540000	2.31602200	2.94343700
H	3.10600300	3.65259100	3.29078100
C	3.35696000	4.69930700	0.77549700
H	2.77165900	5.54683700	1.16443000
H	4.36860900	4.77749600	1.21552600
H	3.45558900	4.81427600	-0.31880000
C	4.88153500	2.03848300	1.95070300
H	5.67723400	1.34912900	1.62248300
H	5.36339500	3.00274500	2.19547100
H	4.44549600	1.63128800	2.87972600
C	3.97784300	0.51795600	-1.70224400
H	4.94654800	1.01611300	-1.53210400
H	4.11409400	-0.24519900	-2.48438300
H	3.25815400	1.26476900	-2.08108100
C	5.82020400	-1.09986700	-0.06657700
H	6.19668100	-0.20184500	0.45329700
H	6.36998200	-1.97065700	0.33565300
H	6.08005000	-1.00497600	-1.13334100
C	4.06478700	-1.50454100	1.67843400
H	4.62626500	-2.39922800	2.00596200
H	4.41775200	-0.65551800	2.28761000
H	2.99733200	-1.68101300	1.91522400
C	4.42281400	-3.01568500	-1.86101600
H	5.46109900	-3.32761500	-1.65191000
H	3.88783900	-3.88899800	-2.27471200
H	4.44506000	-2.23891100	-2.64339400
C	1.45302300	-4.49719700	-0.95434600
H	0.65852400	-4.97192500	-0.35549900
H	1.50598900	-5.03298700	-1.91872600
H	2.39887600	-4.68918400	-0.42644800
C	-1.20559200	-4.71995100	-2.40722500
H	-0.84404200	-5.50337600	-1.72470000
H	-2.21223900	-5.02885300	-2.74440500
H	-0.54426800	-4.69702900	-3.28980400
C	-1.97030600	-3.50999900	-0.32084800
H	-2.17190000	-2.52598900	0.13716500
H	-2.92823100	-4.05619400	-0.40767900
H	-1.30938100	-4.06590100	0.36703600
C	-2.02525400	-2.49239000	-4.09870300
H	-2.44099000	-3.44719200	-4.46463600
H	-2.56724700	-1.67148200	-4.60119700
H	-0.96378200	-2.43487100	-4.40218300
C	-1.07893500	4.45570400	-2.88836500
H	-1.85932400	5.01355100	-3.43515500
H	-0.28443000	5.16886900	-2.60763500
H	-0.63840600	3.70980000	-3.57480800
C	-3.70515600	2.60027300	-0.70514600
H	-4.28392400	3.54011400	-0.62849300
H	-4.41575100	1.76738000	-0.84418700
H	-3.17531700	2.43723700	0.25092700
C	-3.51263200	2.84431900	-3.20420800
H	-4.27699800	2.05273700	-3.30233200
H	-4.04273900	3.81418500	-3.20541700
H	-2.86505300	2.80048300	-4.09635800
H	-1.15078000	1.08674900	1.47401900
C	-2.32309300	-0.34845000	2.56350300

H	-1.85084400	-0.16245000	3.54469800
H	-2.57230400	-1.42760400	2.52604400
O	-3.48892100	0.48702000	2.45270300
Si	-4.97741200	-0.16157900	1.96779300
C	-4.82911200	-1.05888500	0.30869300
H	-4.21615200	-0.49304600	-0.41464900
H	-4.36798900	-2.05381400	0.43767000
H	-5.83102700	-1.21223300	-0.13601700
C	-6.14000800	1.31813600	1.86639600
H	-7.15118700	0.99203800	1.55828600
H	-6.23257900	1.81507100	2.84982800
H	-5.78968000	2.06906500	1.13641700
C	-5.59577400	-1.38999600	3.26677300
H	-4.87360300	-2.21450000	3.41565500
H	-5.75594400	-0.89775800	4.24388700
H	-6.55590000	-1.84195100	2.95236900
Br	0.18405500	-2.21319500	3.26409200

Br^-

E (BP86-D3/6-31G(d)/SMD (MeCN)) = -2572.098445
E (BP86-D3/6-311++G(2df,p)/SMD (MeCN)// BP86-D3/6-31G(d))/SMD (MeCN)) = -2574.565447

Zero-point correction=	0.000000
Thermal correction to Energy=	0.001416
Thermal correction to Enthalpy=	0.002360
Thermal correction to Gibbs Free Energy=	-0.016176

Charge = -1 Multiplicity = 1

Br	0.00000000	0.00000000	0.00000000

$(CH_3)_{15}$(corrin)Co(III)-$(CH_2)_3$-OTMS - II

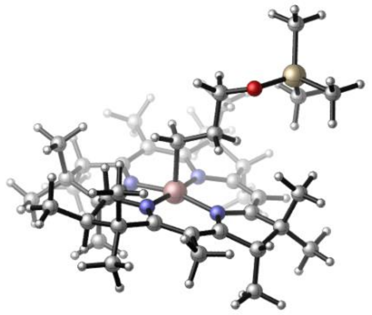

E (BP86-D3/6-31G(d)/SMD (MeCN)) = -3530.848806
E (BP86-D3/6-311++G(2df,p)/SMD (MeCN)// BP86-D3/6-31G(d))/SMD (MeCN)) = -3531.554037

Zero-point correction=	0.975627
Thermal correction to Energy=	1.028655
Thermal correction to Enthalpy=	1.029599
Thermal correction to Gibbs Free Energy=	0.895520

Charge = 1 Multiplicity = 1

C	-0.02249300	2.94578400	0.56731500
C	-1.26055000	3.83448300	0.79396800
C	-1.99019900	3.03923100	1.91991900

C	-1.49916200	1.63849000	1.66095600
N	-0.30972500	1.63282000	0.99226900
H	-3.08635900	3.10245700	1.79515000
C	-2.19102000	0.50613700	2.08333600
H	-3.14596300	0.67352300	2.58902200
C	1.16015500	3.32000300	-0.05197900
C	-1.78157500	-0.80908300	1.89097400
C	-2.65022200	-2.00915400	2.24207300
N	-0.61752400	-1.17019800	1.26632900
C	-1.60458100	-3.15689300	2.16810600
C	-0.52570600	-2.56653700	1.26708200
C	2.23928900	2.37700300	-0.17685400
C	3.67138500	2.73719100	-0.53537800
N	2.09447200	1.08357500	0.05215500
C	4.28951400	1.34095100	-0.91556800
C	3.39537900	0.34483500	-0.08222000
C	0.44897400	-3.32175100	0.62946200
C	1.57553200	-2.68512600	0.00302700
C	2.75241100	-3.38139100	-0.71175100
N	1.69954900	-1.36398600	-0.04169900
C	3.81611500	-2.22674000	-0.71891000
C	2.94333500	-0.96783500	-0.75841200
Co	0.62688600	0.05387000	0.47651300
H	-2.06725900	-4.05247300	1.72018600
H	4.31950800	-2.26888600	0.26558500
H	3.67918000	3.37333700	-1.43944100
H	2.65059700	-0.74583300	-1.80068600
C	-0.10987800	0.23962100	-1.34562800
C	-1.50413400	-0.34953400	-1.42370900
H	-2.14990600	0.07387000	-0.63518200
H	0.57962800	-0.27009800	-2.04186100
H	-0.10151000	1.32261300	-1.56994600
C	0.35299800	-4.83619000	0.66410100
H	0.61322600	-5.27912400	-0.31020700
H	1.02825200	-5.28117600	1.41708200
H	-0.66741600	-5.17123400	0.90179200
C	2.29087300	-3.75064000	-2.14447900
H	1.44390300	-4.45724000	-2.11639300
H	1.96903400	-2.85890200	-2.71181100
H	3.11396200	-4.23586000	-2.69849300
C	3.35453800	-4.61111800	-0.00189600
H	2.77525700	-5.53226600	-0.16733400
H	4.36884000	-4.79105500	-0.40291300
H	3.44659300	-4.44256200	1.08574600
C	4.87529600	-2.32667400	-1.81844900
H	5.66173000	-1.56671700	-1.67771300
H	5.36792300	-3.31570700	-1.80146400
H	4.43976300	-2.17985900	-2.82200200
C	3.92549200	0.06795500	1.33993700
H	4.89914200	-0.44754100	1.31464200
H	4.04352700	1.00644900	1.90367200
H	3.20640600	-0.56735100	1.88619300
C	5.79129600	1.24140300	-0.60847700
H	6.17762200	0.23879600	-0.86033300
H	6.34861000	1.97631700	-1.21732500
H	6.02593300	1.43705600	0.45004000
C	4.07745900	1.16371400	-2.43730600
H	4.62863600	1.95561600	-2.97619800
H	4.45277600	0.19244300	-2.79924300
H	3.01076800	1.25009800	-2.71612800

C	4.35294000	3.54980500	0.58657000
H	5.38644800	3.81039100	0.30114200
H	3.80504000	4.49251900	0.75878200
H	4.38414200	3.00013400	1.54182900
C	1.37067800	4.71947400	-0.60251600
H	0.48537300	5.07070600	-1.15589000
H	1.58628400	5.46063300	0.18744300
H	2.21409800	4.74171300	-1.30932400
C	-1.04015600	5.30632300	1.18693300
H	-0.76652800	5.93339500	0.32570600
H	-1.98634200	5.70959200	1.59131700
H	-0.26219800	5.41988500	1.96038800
C	-2.10641300	3.74911900	-0.50600800
H	-2.32231700	2.69751500	-0.76592200
H	-3.06720500	4.27798100	-0.36787900
H	-1.57465600	4.21043400	-1.35608000
C	-1.62490000	3.44699800	3.36530800
H	-1.98089000	4.46494200	3.59737700
H	-2.09527100	2.74947900	4.08059000
H	-0.53147500	3.41313700	3.52201300
C	-0.97017700	-3.55153100	3.52053000
H	-1.72823100	-3.96567900	4.20717000
H	-0.19174400	-4.31895300	3.37022500
H	-0.49879000	-2.67780700	4.00572800
C	-3.68983900	-2.18515000	1.10078900
H	-4.29968900	-3.08808200	1.28805100
H	-4.36459600	-1.31317600	1.05026600
H	-3.19618800	-2.29685400	0.11931100
C	-3.38624100	-1.87969900	3.58536800
H	-4.15560700	-1.08916100	3.53361000
H	-3.90275700	-2.82599600	3.82612500
H	-2.70119900	-1.63477300	4.41445600
H	-1.46279900	-1.44072000	-1.25570700
C	-2.19901700	-0.11390700	-2.77647000
H	-1.55900700	-0.47391100	-3.60581800
H	-2.36503100	0.97236600	-2.93763600
O	-3.42683200	-0.85897400	-2.82521500
Si	-4.93994400	-0.18764900	-2.45862400
C	-4.84144000	1.05598000	-1.03800700
H	-4.34345100	0.64458000	-0.14216600
H	-4.29781200	1.96785600	-1.34206700
H	-5.86378400	1.36459200	-0.74639100
C	-6.02133900	-1.66086600	-1.99948300
H	-7.05849600	-1.33345500	-1.79777600
H	-6.05617500	-2.40439500	-2.81702900
H	-5.64283900	-2.16752800	-1.09302400
C	-5.62143500	0.69790700	-3.98390700
H	-4.94735600	1.51532700	-4.30234900
H	-5.73857800	0.00303000	-4.83600300
H	-6.61132400	1.14497200	-3.77175800

$(CH_3)_{15}$(corrin)Co(II)

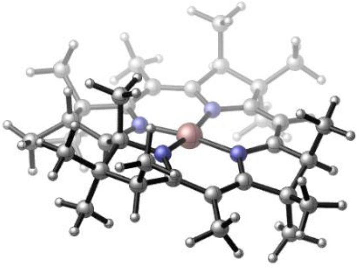

E (BP86-D3/6-31G(d)/SMD (MeCN)) = -2928.348697
E (BP86-D3/6-311++G(2df,p)/SMD (MeCN)// BP86-D3/6-31G(d))/SMD (MeCN)) = -2928.92078

Zero-point correction=	0.778592
Thermal correction to Energy=	0.818236
Thermal correction to Enthalpy=	0.819181
Thermal correction to Gibbs Free Energy=	0.713227

Charge = 1 Multiplicity = 2

C	2.90498200	-0.90195900	-0.30446300
C	4.33074400	-0.38391700	-0.59130600
C	4.26947300	1.02558600	0.07486500
C	2.79671400	1.33400800	-0.00989800
N	2.04706500	0.19558500	-0.09529300
H	4.86572800	1.75674000	-0.50045800
C	2.29062500	2.63069000	0.03041200
H	3.02054400	3.44349700	0.07654000
C	2.48974000	-2.22584700	-0.31325300
C	0.94221900	2.97223700	-0.00210200
C	0.44122000	4.40810400	-0.07837300
N	-0.08344700	2.06585400	-0.04190800
C	-1.03607500	4.22043600	0.36969000
C	-1.30022400	2.75671600	0.03320100
C	1.12923900	-2.56176500	0.01082000
C	0.62396100	-3.96906400	0.28788100
N	0.16984700	-1.65986700	0.13702400
C	-0.93442400	-3.80651400	0.14738100
C	-1.14172000	-2.28414600	0.50344300
C	-2.56676400	2.20189600	-0.08441900
C	-2.73603700	0.77819600	-0.20907000
C	-4.06283500	0.01647400	-0.40895700
N	-1.69976200	-0.05101600	-0.21148100
C	-3.63172100	-1.44177500	-0.01861200
C	-2.13591700	-1.46734800	-0.35957600
Co	0.13802700	0.17678200	-0.07992700
H	-1.68965200	4.89938500	-0.20384900
H	-3.73428700	-1.50390900	1.08110400
H	0.97070400	-4.65652700	-0.50509300
H	-1.98750900	-1.73777600	-1.42051800
C	-3.78371400	3.10797400	-0.03237600
H	-4.51296900	2.84977100	-0.81770300
H	-4.31049500	3.04383200	0.93685100
H	-3.51184000	4.16230400	-0.18678500
C	-4.45785200	0.12971400	-1.90262600
H	-4.61600400	1.18456200	-2.18636600
H	-3.67608400	-0.28674200	-2.56295600

H	-5.39974800	-0.41456800	-2.09363500
C	-5.23870700	0.44258500	0.49456000
H	-5.75774300	1.34316800	0.13279600
H	-5.98363800	-0.37414700	0.51492200
H	-4.90516500	0.62044600	1.53235500
C	-4.46922800	-2.55690100	-0.64863400
H	-4.20807800	-3.53705100	-0.21540900
H	-5.54618100	-2.39245300	-0.46315200
H	-4.31688300	-2.61785200	-1.74030100
C	-1.37903300	-2.01522200	2.00377500
H	-2.32577500	-2.45612900	2.35496000
H	-0.55804400	-2.43019200	2.60880200
H	-1.41152100	-0.92535600	2.18025600
C	-1.73310700	-4.76764100	1.04086800
H	-2.81756100	-4.59258600	0.92956400
H	-1.53149300	-5.81318300	0.74483700
H	-1.48357600	-4.66785900	2.10943700
C	-1.27497500	-4.10670500	-1.33166100
H	-1.00784400	-5.15453200	-1.55983800
H	-2.34909500	-3.98432700	-1.54604700
H	-0.70651700	-3.45963700	-2.02503500
C	1.17610900	-4.51409200	1.62272700
H	0.79969000	-5.53465900	1.80844200
H	2.27818800	-4.56482200	1.58487200
H	0.89894100	-3.87907900	2.48041300
C	3.43684800	-3.36093000	-0.66486100
H	4.07101000	-3.10421000	-1.52812200
H	4.10597000	-3.63557600	0.17016700
H	2.87792000	-4.26728400	-0.94426000
C	5.51724400	-1.21131600	-0.06260000
H	5.73246900	-2.08537600	-0.69475900
H	6.42237400	-0.57718700	-0.07214800
H	5.35620000	-1.56127200	0.97080300
C	4.46260900	-0.20144200	-2.12826400
H	3.68551900	0.48069300	-2.51897700
H	5.45203200	0.22820600	-2.37047600
H	4.36788000	-1.16811600	-2.65270800
C	4.70791000	1.07920500	1.55658200
H	5.78559800	0.86975500	1.66461000
H	4.51320500	2.08541000	1.96772900
H	4.14579800	0.34613100	2.16315000
C	-1.29103600	4.44175700	1.87750100
H	-1.09133000	5.48869500	2.16374600
H	-2.34196700	4.21469800	2.12597000
H	-0.64617800	3.78378700	2.48783700
C	0.47611400	4.84116100	-1.56919500
H	0.07655000	5.86680100	-1.67211200
H	1.51081300	4.82888700	-1.95529400
H	-0.13671500	4.16707600	-2.19528600
C	1.25567500	5.40423800	0.76234300
H	2.26529700	5.54166500	0.33636200
H	0.76088200	6.39192700	0.76169300
H	1.36958800	5.07162000	1.80787100

$(CH_2)_3OTMS$

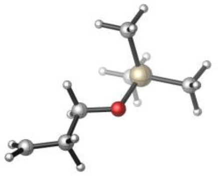

E (BP86-D3/6-31G(d)/SMD (MeCN)) = -602.403478
E (BP86-D3/6-311++G(2df,p)/SMD (MeCN)// BP86-D3/6-31G(d))/SMD (MeCN)) = -602.549784

Zero-point correction=	0.189658
Thermal correction to Energy=	0.202598
Thermal correction to Enthalpy=	0.203542
Thermal correction to Gibbs Free Energy=	0.150106

Charge = 0 Multiplicity = 2

C	3.96232800	-0.22748700	0.02902600
C	2.72313500	0.59571600	-0.08527000
H	2.70404100	1.39475100	0.68063500
H	2.67767000	1.11908500	-1.07029400
C	1.44093000	-0.24378400	0.03268000
H	1.45521100	-1.05167700	-0.73025800
H	1.40476900	-0.72994700	1.03046200
O	0.30612600	0.61458500	-0.16174100
Si	-1.26904200	0.00254500	0.00065300
C	-1.55187500	-1.40173300	-1.23244800
H	-0.89272200	-2.26566900	-1.02840300
H	-2.59714700	-1.76049900	-1.17642900
H	-1.36445800	-1.06772400	-2.26982300
C	-1.54961300	-0.62229300	1.76239500
H	-1.36859000	0.17729500	2.50443300
H	-2.59321900	-0.96869600	1.88665400
H	-0.88647900	-1.47203200	2.00854900
C	-2.39330100	1.46597000	-0.37709400
H	-2.21951700	2.29952500	0.32800300
H	-2.23059900	1.84531500	-1.40262700
H	-3.45524900	1.16842600	-0.29203300
H	4.02787700	-1.20459000	-0.46410100
H	4.86636300	0.16580600	0.50429800

TD DFT calculations

Three lowest singlet exited states for intermediate **II** were calculated at TD-BP86-D3/6-311++G(2df,p) level of theory including solvation (acetonitrile) with SMD model.

$(CH_3)_{15}$(corrin)Co(III)-$(CH_2)_3$-OTMS – II *(vertical excitation)*

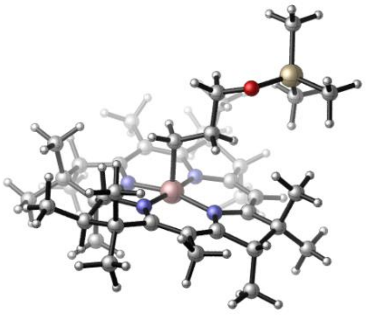

Excitation energies and oscillator strengths:

```
 Excited State   1:      Singlet-A      2.2335 eV  555.11 nm  f=0.0225  <S**2>=0.000
     189 -> 193       -0.13359
     190 -> 192        0.31747
     191 -> 192        0.50814
     191 -> 193       -0.32300
 Total Energy, E(TD-HF/TD-DFT) =  -3531.47195770

 Excited State   2:      Singlet-A      2.2995 eV  539.18 nm  f=0.0211  <S**2>=0.000
     189 -> 193       -0.13850
     190 -> 192       -0.21775
     191 -> 192        0.40684
     191 -> 193        0.49373

 Excited State   3:      Singlet-A      2.3412 eV  529.57 nm  f=0.0161  <S**2>=0.000
     189 -> 192        0.18852
     190 -> 192        0.56898
     191 -> 192       -0.11554
     191 -> 193        0.32439
     191 -> 194       -0.12033
```

Simulated UV spectrum of II calculated for 20 excited states at TD-BP86/6-311++G(2df,p)level of theory

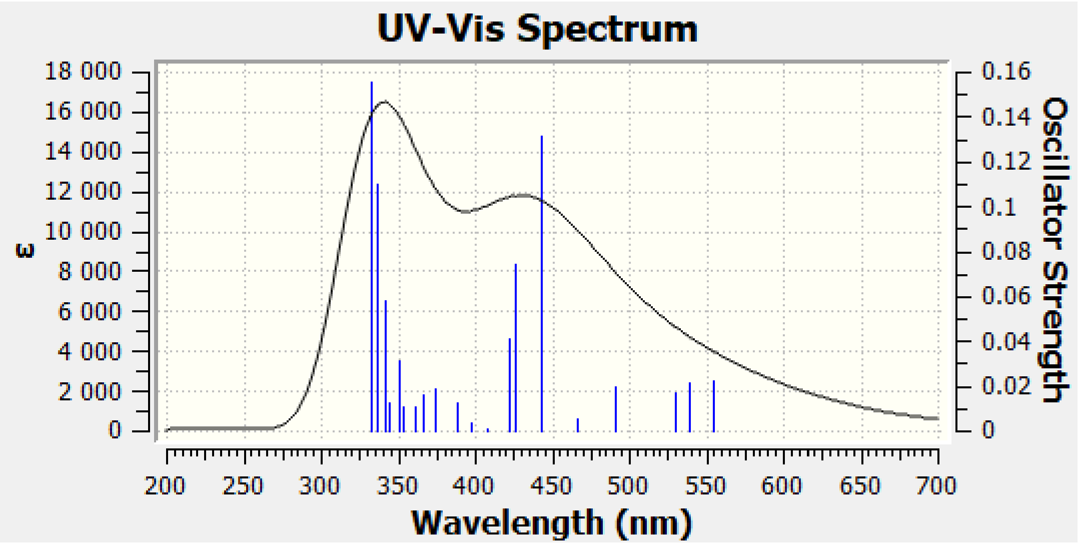

$(CH_3)_{15}$(corrin)Co(III)-$(CH_2)_3$-OTMS – II *(Relaxed)*

Geometry of the II was optimized at TD-BP86-D3/6-31G(d) (root=1) including solvation (acetone) with SMD model. Frequency analysis was performed at the same level to provide correction to thermodynamic functions Then the energy of the lowest singlet exited state was calculated employing TD-BP86-D3/6-311++G(2df,p).

E (TD-BP86-D3/6-31G(d)/SMD (MeCN)) = -3530.776482
E (TD-BP86-D3/6-311++G(2df,p)/SMD (MeCN)// TD-BP86-D3/6-31G(d))/SMD (MeCN)) = -3531.471958

Zero-point correction=	0.972234
Thermal correction to Energy=	1.026142
Thermal correction to Enthalpy=	1.027087
Thermal correction to Gibbs Free Energy=	0.890414

Charge = 0 Multiplicity = 1

C	-0.05597300	2.95315800	0.51546400
C	-1.25993200	3.87600000	0.78692900
C	-1.99762400	3.07513100	1.90783500
C	-1.55005400	1.66692400	1.61765700
N	-0.36947900	1.66558000	0.89620700
H	-3.09307800	3.17258000	1.79710400
C	-2.20301800	0.52747200	2.06337300
H	-3.16008300	0.68409000	2.57039200
C	1.16713300	3.31830700	-0.08187300
C	-1.77739600	-0.79239700	1.90868300
C	-2.64637500	-2.00120800	2.23338800
N	-0.57321200	-1.17858900	1.35501900
C	-1.58050100	-3.13648400	2.23810900
C	-0.48976600	-2.56155600	1.34147900
C	2.22604300	2.37490400	-0.14438300
C	3.65744300	2.72325500	-0.51844500
N	2.11913500	1.07964200	0.18125100
C	4.27985300	1.33021700	-0.88594700
C	3.40347700	0.32734900	-0.03346700
C	0.47318100	-3.33208100	0.67083200
C	1.56450100	-2.70093200	0.01437600
C	2.74212900	-3.38597800	-0.70763400

N	1.68264000	-1.36888500	-0.05812700
C	3.80329900	-2.22863500	-0.71135400
C	2.93261700	-0.96386400	-0.74307600
Co	0.54621400	0.06131100	0.37220200
H	-2.01988600	-4.05485500	1.81377100
H	4.30699200	-2.27769300	0.27211800
H	3.67207100	3.36061400	-1.42055500
H	2.66746800	-0.71885500	-1.78778200
C	-0.06985200	0.26197000	-1.45820900
C	-1.45791300	-0.35823300	-1.45747300
H	-2.09475000	0.08192200	-0.67115100
H	0.61606800	-0.26193400	-2.14333600
H	-0.08474800	1.34616800	-1.67555700
C	0.35610100	-4.84532400	0.70809600
H	0.64473900	-5.29931600	-0.25278400
H	0.99176200	-5.30085200	1.49026600
H	-0.67878000	-5.16375700	0.90717200
C	2.27591500	-3.74673300	-2.14076100
H	1.43119100	-4.45645500	-2.11111000
H	1.94702300	-2.85136800	-2.69863700
H	3.09684600	-4.22456600	-2.70482500
C	3.34981200	-4.61818500	-0.00767800
H	2.76733700	-5.53793400	-0.17047400
H	4.36102300	-4.79983700	-0.41592800
H	3.44894400	-4.45293900	1.07988200
C	4.86374300	-2.32429600	-1.81009200
H	5.64993400	-1.56447600	-1.66623800
H	5.35710900	-3.31304500	-1.79555900
H	4.42969000	-2.17526800	-2.81404300
C	3.97404300	0.01282700	1.36360000
H	4.94238100	-0.50980400	1.30109900
H	4.11635900	0.93691700	1.94574300
H	3.26313700	-0.63106400	1.91143900
C	5.78530900	1.24408100	-0.59157900
H	6.17799700	0.24564200	-0.85000900
H	6.33082600	1.98568200	-1.20302500
H	6.02715000	1.43843900	0.46550800
C	4.05964400	1.15067000	-2.40687100
H	4.60514700	1.94656000	-2.94582500
H	4.43758600	0.18276100	-2.77411100
H	2.99162700	1.23525600	-2.68139400
C	4.32515200	3.53341800	0.61546100
H	5.36495500	3.78363600	0.34451700
H	3.78276400	4.48066500	0.77766300
H	4.33483600	2.98215800	1.57016000
C	1.39001300	4.71238000	-0.63692900
H	0.50315600	5.07268100	-1.18271200
H	1.62251600	5.45678900	0.14631100
H	2.22640900	4.72428100	-1.35283700
C	-0.98382400	5.33087000	1.20464100
H	-0.69911200	5.96274400	0.35047200
H	-1.91045200	5.75884900	1.62803500
H	-0.19281900	5.40323200	1.96966500
C	-2.12434500	3.84701800	-0.50422300
H	-2.37963800	2.80856600	-0.78061300
H	-3.06413100	4.40531900	-0.33987400
H	-1.58913400	4.30847300	-1.35205300
C	-1.60470200	3.45300600	3.35478700
H	-1.92173000	4.47957800	3.60560500
H	-2.09280400	2.76133600	4.06373000

H	-0.51166800	3.37795000	3.50005700
C	-0.97720000	-3.47291500	3.61946700
H	-1.74433500	-3.87950700	4.30122300
H	-0.18060100	-4.22965700	3.51203300
H	-0.53479200	-2.57494100	4.08778600
C	-3.62324900	-2.22046100	1.04607200
H	-4.21534200	-3.13974100	1.21220600
H	-4.31926800	-1.36903700	0.95452700
H	-3.08110800	-2.32771900	0.08981400
C	-3.45006500	-1.86072400	3.53583200
H	-4.22707800	-1.08230300	3.43433800
H	-3.96429000	-2.81107700	3.76605300
H	-2.80960000	-1.59326800	4.39330000
H	-1.38928900	-1.44290900	-1.26413600
C	-2.18853400	-0.16740600	-2.80811600
H	-1.56157400	-0.55501500	-3.63368900
H	-2.35882300	0.91327500	-2.99407600
O	-3.40710500	-0.91616800	-2.79325000
Si	-4.91626500	-0.21888200	-2.43240000
C	-4.79215500	1.02590400	-1.01614500
H	-4.29528800	0.61109800	-0.12148400
H	-4.23730100	1.92864500	-1.32726600
H	-5.80795300	1.35106000	-0.71993400
C	-6.01434800	-1.67927200	-1.97826600
H	-7.04335900	-1.33731800	-1.76017100
H	-6.07056900	-2.41058100	-2.80547900
H	-5.63378900	-2.20397200	-1.08330000
C	-5.56730200	0.67280800	-3.96579300
H	-4.87317900	1.47329600	-4.28386600
H	-5.69536300	-0.02404200	-4.81449900
H	-6.54781500	1.14299800	-3.76057000

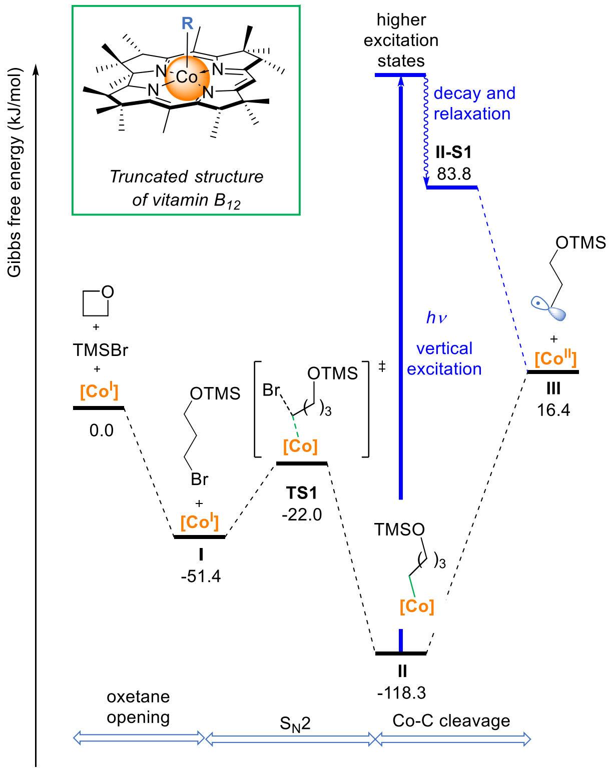

Figure S4 Calculated Gibbs free energy profile for the reaction of oxetanes with the Co(I)-corrin complex in the presence of TMSBr

8. References

(1) Strassfeld, D. A.; Wickens, Z. K.; Picazo, E.; Jacobsen, E. N. Highly Enantioselective, Hydrogen-Bond-Donor Catalyzed Additions to Oxetanes. *J. Am. Chem. Soc.* **2020**, *142* (20), 9175–9180. https://doi.org/10.1021/jacs.0c03991.

(2) Altenbach, Robert J.; Bogdan, Andrew; Couty, Sylvain; Desroy, Nicolas; Gfesser, Gregory A.; Housseman, Christopher Gaëtan; Kym, Philip R.; Liu, Bo; Mai, Thi Thu Trang; Malagu, Karine Fabienne; Merayo Merayo, Nuria; Picolet, Olivier Laurent; Pizzonero, Mat, M. C. Modulators of the Cystic Fibrosis Transmembrane Conductance Regulator Protein and Methods of Use. US2019/77784, 2019, A1.

(3) Wang, Z.; Chen, Z.; Sun, J. Catalytic Enantioselective Intermolecular Desymmetrization of 3-Substituted Oxetanes. *Angew. Chemie Int. Ed.* **2013**, *52* (26), 6685–6688. https://doi.org/10.1002/anie.201300188.

(4) Kwon, D. W.; Kim, Y. H.; Lee, K. Highly Regioselective Cleavages and Iodinations of Cyclic Ethers Utilizing SmI 2. *J. Org. Chem.* **2002**, *67* (26), 9488–9491. https://doi.org/10.1021/jo020179r.

(5) Singh, A.; Anandhi, U.; Cinellu, M. A.; Sharp, P. R. Diimine Supported Group 10 Hydroxo, Oxo, Amido, and Imido Complexes. *Dalt. Trans.* **2008**, No. 17, 2314–2327. https://doi.org/10.1039/b715663d.

(6) Ociepa, M.; Wierzba, A. J.; Turkowska, J.; Gryko, D. Polarity-Reversal Strategy for the Functionalization of Electrophilic Strained Molecules via Light-Driven Cobalt Catalysis. *J. Am. Chem. Soc.* **2020**, *142* (11), 5355–5361. https://doi.org/10.1021/jacs.0c00245.

(7) Cheung, L. L.; Marumoto, S.; Anderson, C. D.; Rychnovsky, S. D. Assignment of Absolute Configuration to SCH 351448 via Total Synthesis. *Org. Lett.* **2008**, *10* (14), 3101–3104. https://doi.org/10.1021/ol8011474.

(8) Shen, R.; Inoue, T.; Forgac, M.; Porco, J. A. Synthesis of Photoactivatable Acyclic Analogues of the Lobatamides. *J. Org. Chem.* **2005**, *70* (9), 3686–3692. https://doi.org/10.1021/jo0477751.

(9) Westerbeek, A.; Van Leeuwen, J. G. E.; Szymański, W.; Feringa, B. L.; Janssen, D. B. Haloalkane Dehalogenase Catalysed Desymmetrisation and Tandem Kinetic Resolution for the Preparation of Chiral Haloalcohols. *Tetrahedron* **2012**, *68* (37), 7645–7650. https://doi.org/10.1016/j.tet.2012.06.059.

(10) Bettoni, L.; Gaillard, S.; Renaud, J.-L. Iron-Catalyzed β-Alkylation of Alcohols. *Org. Lett.* **2019**, *21* (20), 8404–8408. https://doi.org/10.1021/acs.orglett.9b03171.

(11) Manojveer, S.; Salahi, S.; Wendt, O. F.; Johnson, M. T. Ru-Catalyzed Cross-Dehydrogenative Coupling between Primary Alcohols to Guerbet Alcohol Derivatives: With Relevance for Fragrance Synthesis. *J. Org. Chem.* **2018**, *83* (18), 10864–10870. https://doi.org/10.1021/acs.joc.8b01558.

(12) Stach, T.; Dräger, J.; Huy, P. H. Nucleophilic Substitutions of Alcohols in High Levels of Catalytic Efficiency. *Org. Lett.* **2018**, *20* (10), 2980–2983. https://doi.org/10.1021/acs.orglett.8b01023.

(13) Lee, D.; Williamson, C. L.; Chan, L.; Taylor, M. S. Regioselective, Borinic Acid-Catalyzed Monoacylation, Sulfonylation and Alkylation of Diols and Carbohydrates: Expansion of Substrate Scope and Mechanistic Studies. *J. Am. Chem. Soc.* **2012**, *134* (19), 8260–8267. https://doi.org/10.1021/ja302549c.

(14) Fujioka, H.; Ohba, Y.; Hirose, H.; Murai, K.; Kita, Y. Mild and Efficient Removal of Hydroxyethyl Unit from 2-Hydroxyethyl Ether Derivatives Leading to Alcohols. *Org. Lett.* **2005**, *7* (15), 3303–3306. https://doi.org/10.1021/ol051135i.

(15) Cano, R.; Yus, M.; Ramón, D. J. First Practical Cross-Alkylation of Primary Alcohols with a New and Recyclable Impregnated Iridium on Magnetite Catalyst. *Chem. Commun.* **2012**, *48* (61), 7628. https://doi.org/10.1039/c2cc33101b.

(16) James, B. G.; Pattenden, G. Regiospecificity of Methylation of Unsymmetrical Stilbenes by Methylsulphinylmethanide. *J. Chem. Soc. Perkin Trans. 1* **1974**, 1195. https://doi.org/10.1039/p19740001195.

(17) Hayashi, H.; Kaga, A.; Wang, B.; Gagosz, F.; Chiba, S. Use of a Benzyl Ether as a Traceless Hydrogen Donor in the Anti-Markovnikov Hydrofunctionalization of Alkenes with Xanthates. *Chem. Commun.* **2018**, *54* (54), 7535–7538. https://doi.org/10.1039/c8cc02971g.

(18) Frisch, M. J.; Trucks, G. W.; Schlegel, H. B.; Scuseria, G. E.; Robb, M. A.; Cheeseman, J. R.; Scalmani, G.; Barone, V.; Petersson, G. A.; Nakatsuji, H.; Li, X.; Caricato, M.; Marenich, A. V.; Bloino, J.; Janesko, B. G.; Gomperts, R.; Mennucci, B.; Hratchian, H. P.; Ortiz, J. V.; Izmaylov, A. F.; Sonnenberg, J. L.; Williams-Young, D.; Ding, F.; Lipparini, F.; Egidi, F.; Goings, J.; Peng, B.; Petrone, A.; Henderson, T.; Ranasinghe, D.; Zakrzewski, V. G.; Gao, J.; Rega, N.; Zheng, G.; Liang, W.; Hada, M.; Ehara, M.; Toyota, K.; Fukuda, R.; Hasegawa, J.; Ishida, M.; Nakajima, T.; Honda, Y.; Kitao, O.; Nakai, H.; Vreven, T.; Throssell, K.; Montgomery, J. A., Jr.; Peralta, J. E.; Ogliaro, F.; Bearpark, M. J.; Heyd, J. J.; Brothers, E. N.; Kudin, K. N.; Staroverov, V. N.; Keith, T. A.; Kobayashi, R.; Normand, J.; Raghavachari, K.; Rendell, A. P.; Burant, J. C.; Iyengar, S. S.; Tomasi, J.; Cossi, M.; Millam, J. M.; Klene, M.; Adamo, C.; Cammi, R.; Ochterski, J. W.; Martin, R. L.; Morokuma, K.; Farkas, O.; Foresman, J. B.; Fox, D. J. Gaussian 16, Revision B.01; Gaussian, Inc.: Wallingford, CT, 2016

(19) Grimme, S.; Antony, J.; Ehrlich, S.; Krieg, H. A Consistent and Accurate Ab Initio Parametrization of Density Functional Dispersion Correction (DFT-D) for the 94 Elements H-Pu. *J. Chem. Phys.* **2010**, *132* (15). https://doi.org/10.1063/1.3382344.

(20) Marenich, A. V.; Cramer, C. J.; Truhlar, D. G. Universal Solvation Model Based on Solute Electron Density and on a Continuum Model of the Solvent Defined by the Bulk Dielectric Constant and Atomic Surface Tensions. *J. Phys. Chem. B* **2009**, *113* (18), 6378–6396. https://doi.org/10.1021/jp810292n.

(21) Legault, C. Y., CYLview, 1.0b; Université de Sherbrooke, 2009, http://www.cylview.org.

9. NMR spectra

^{1}H NMR (400 MHz, $CDCl_3$) and ^{13}C NMR (100 MHz, $CDCl_3$); triisopropyl(2-(oxetan-2-yl)ethoxy)silane (S14)

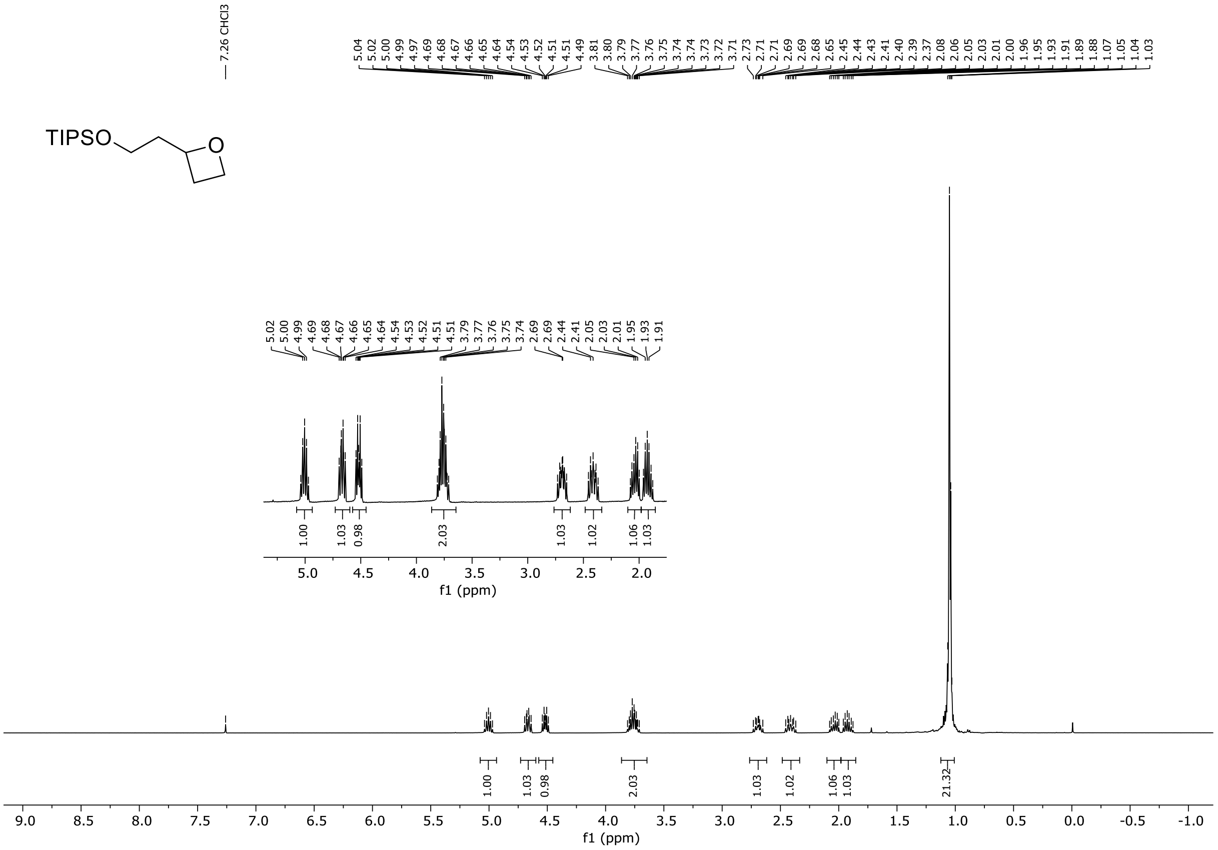

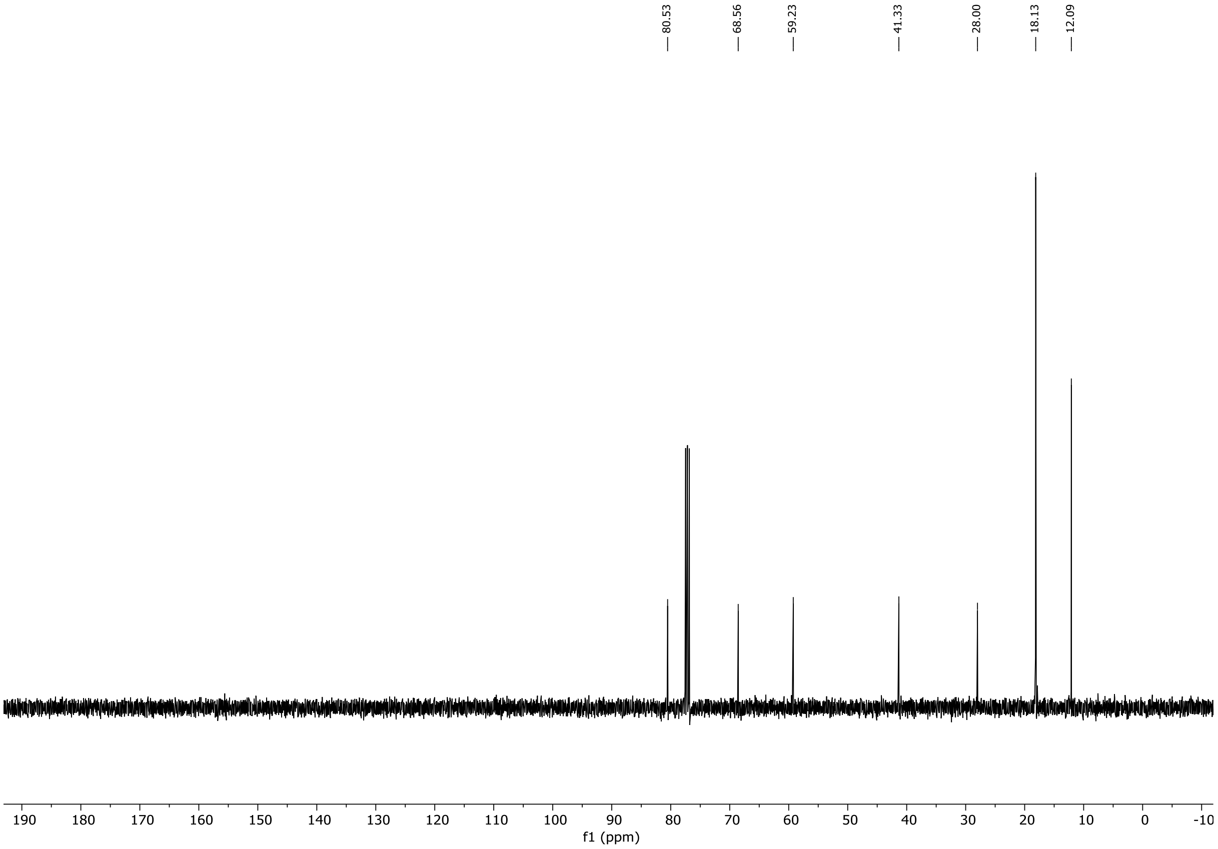

^{1}H NMR (500 MHz, $CDCl_3$) and ^{13}C NMR (125 MHz, $CDCl_3$); 3-bromo-2-phenyl-propan-1-ol (5b)

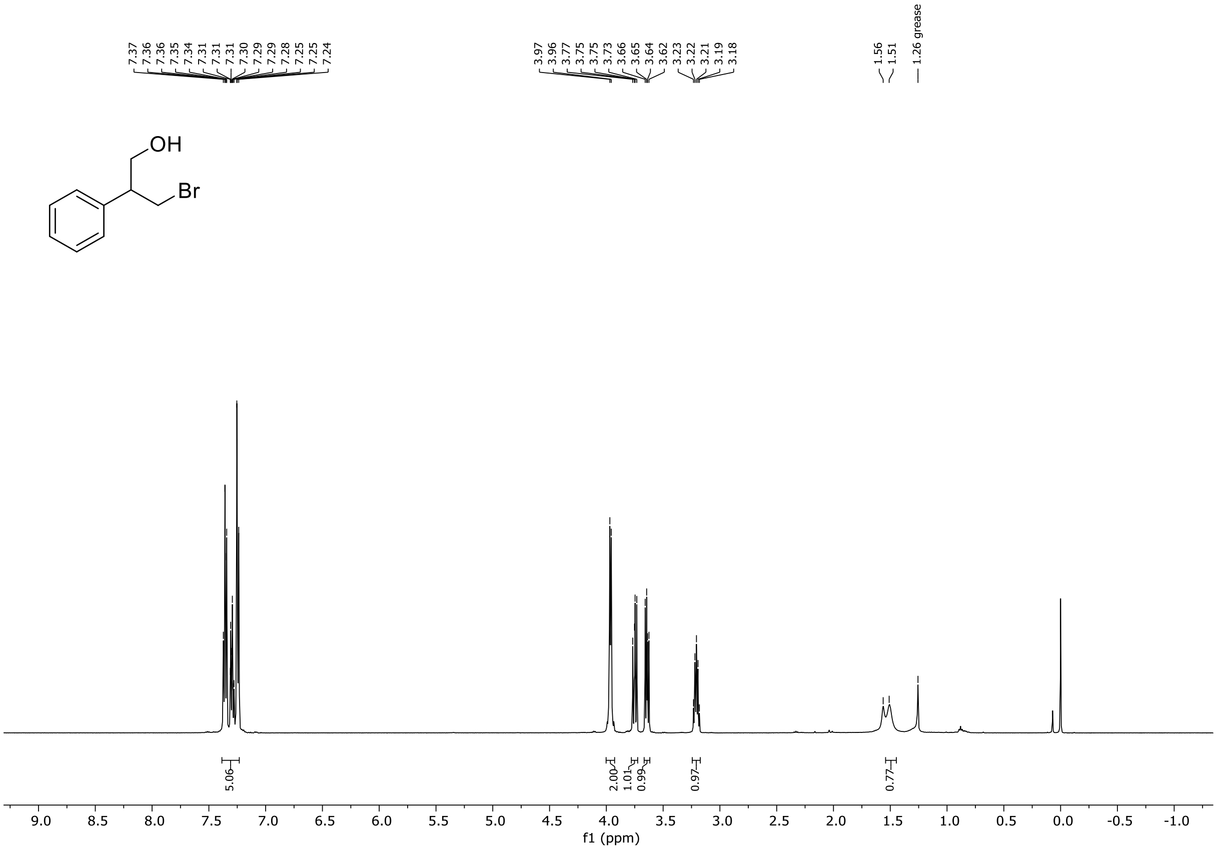

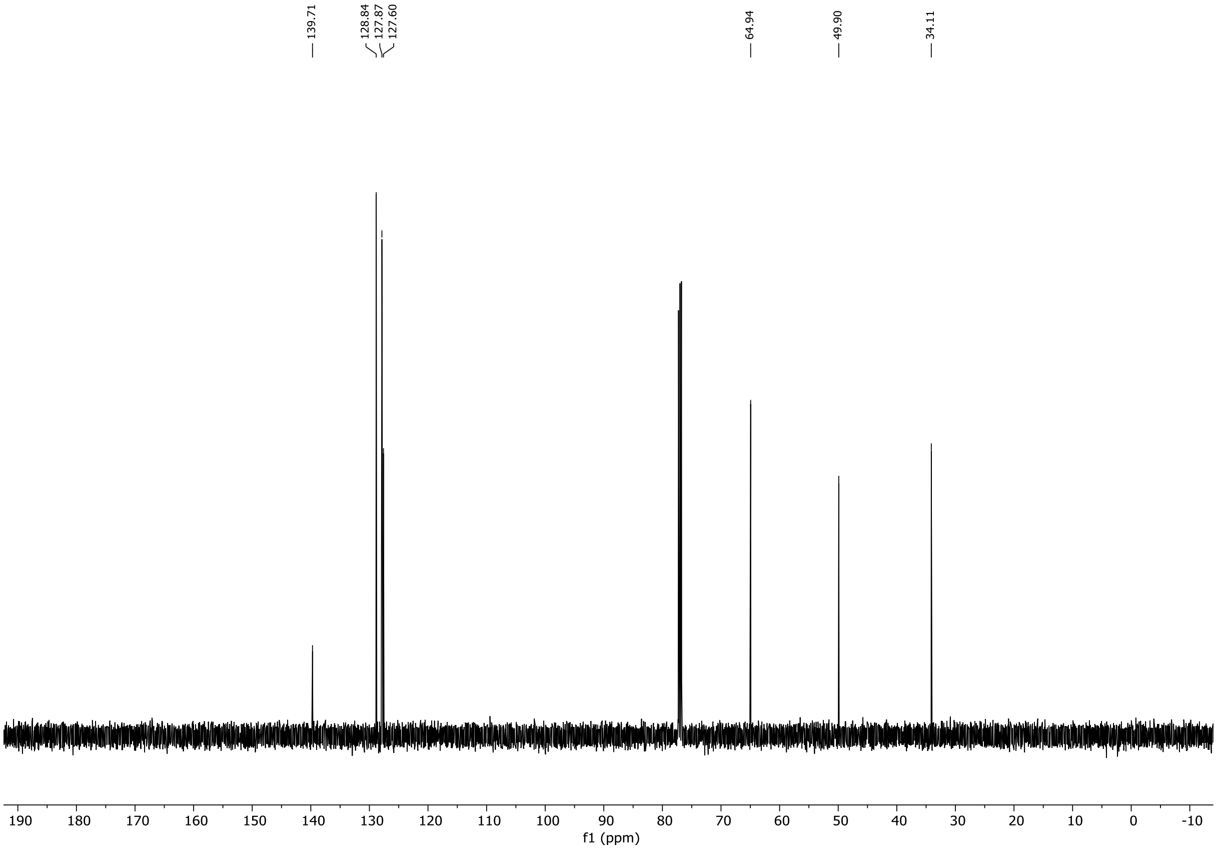

^{1}H NMR (400 MHz, $CDCl_3$) and ^{13}C NMR (100 MHz, $CDCl_3$); 2,3-diphenylpropan-1-ol (4b)

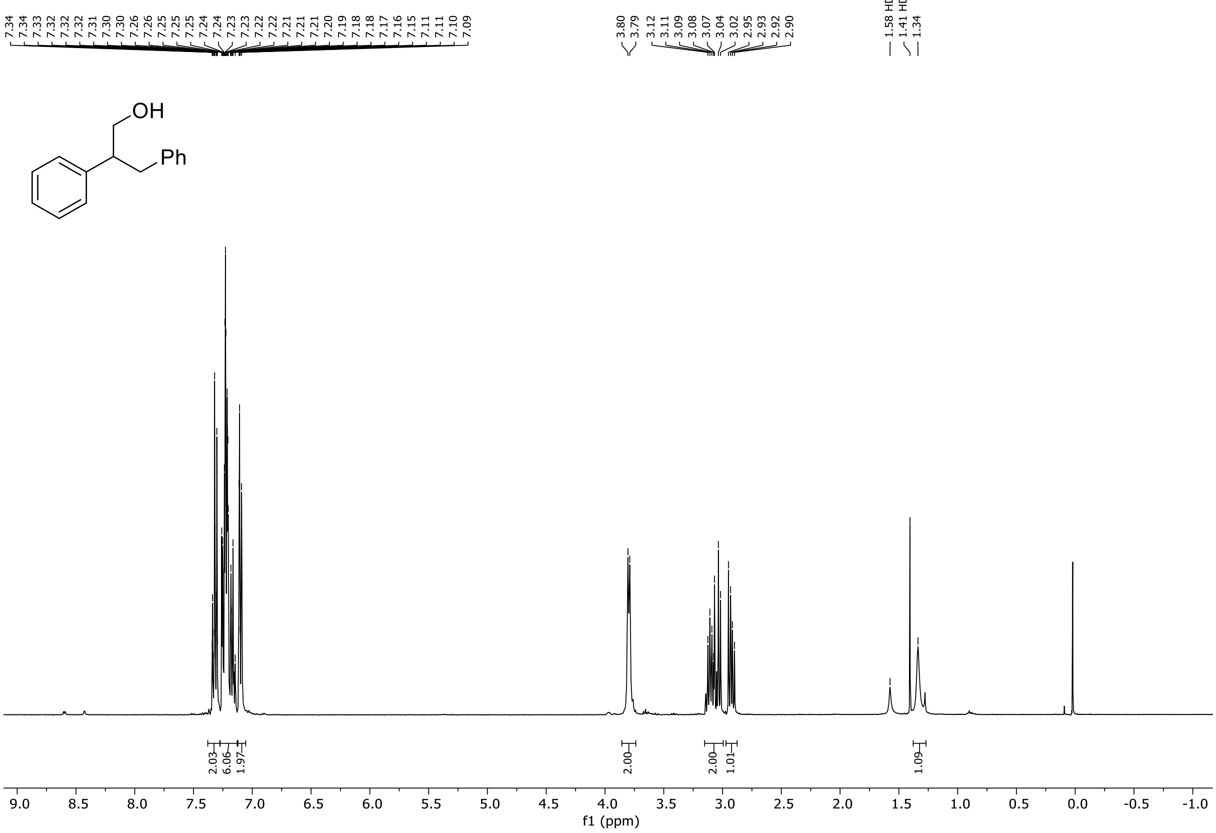

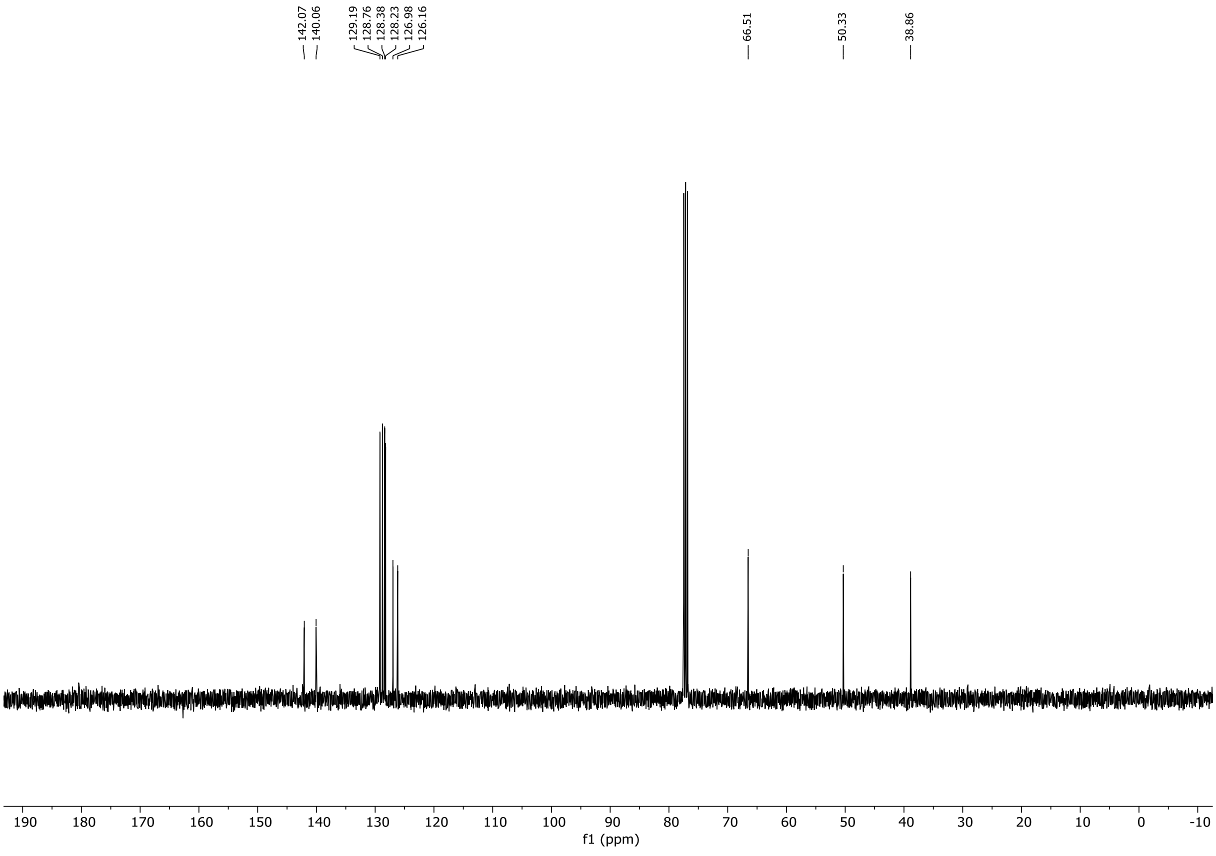

^{1}H NMR (400 MHz, $CDCl_3$) and ^{13}C NMR (100 MHz, $CDCl_3$); 4-(1-hydroxy-3-phenylpropan-2-yl)benzonitrile (4d)

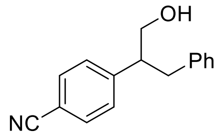

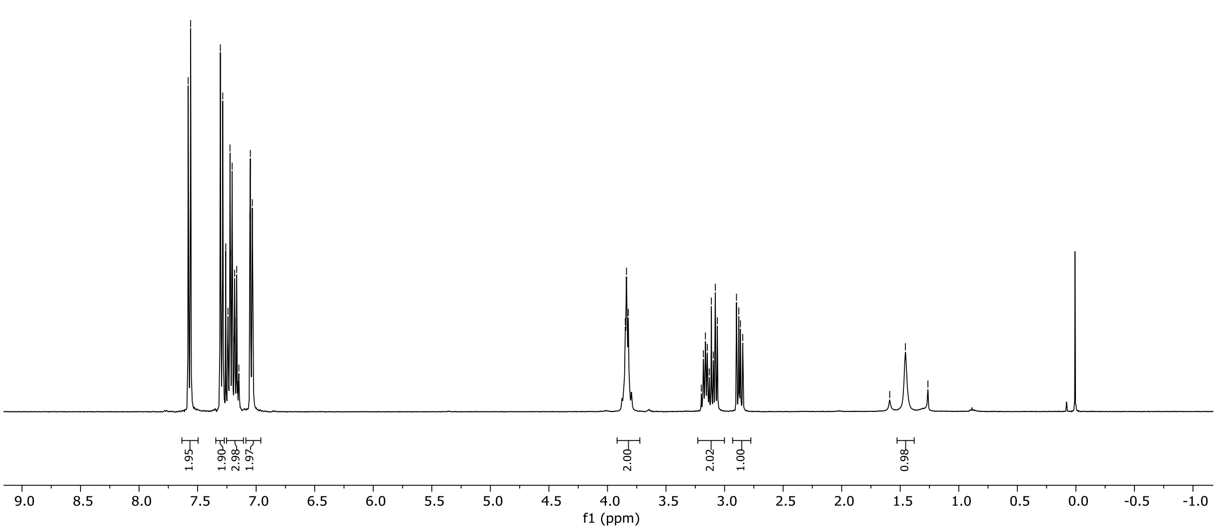

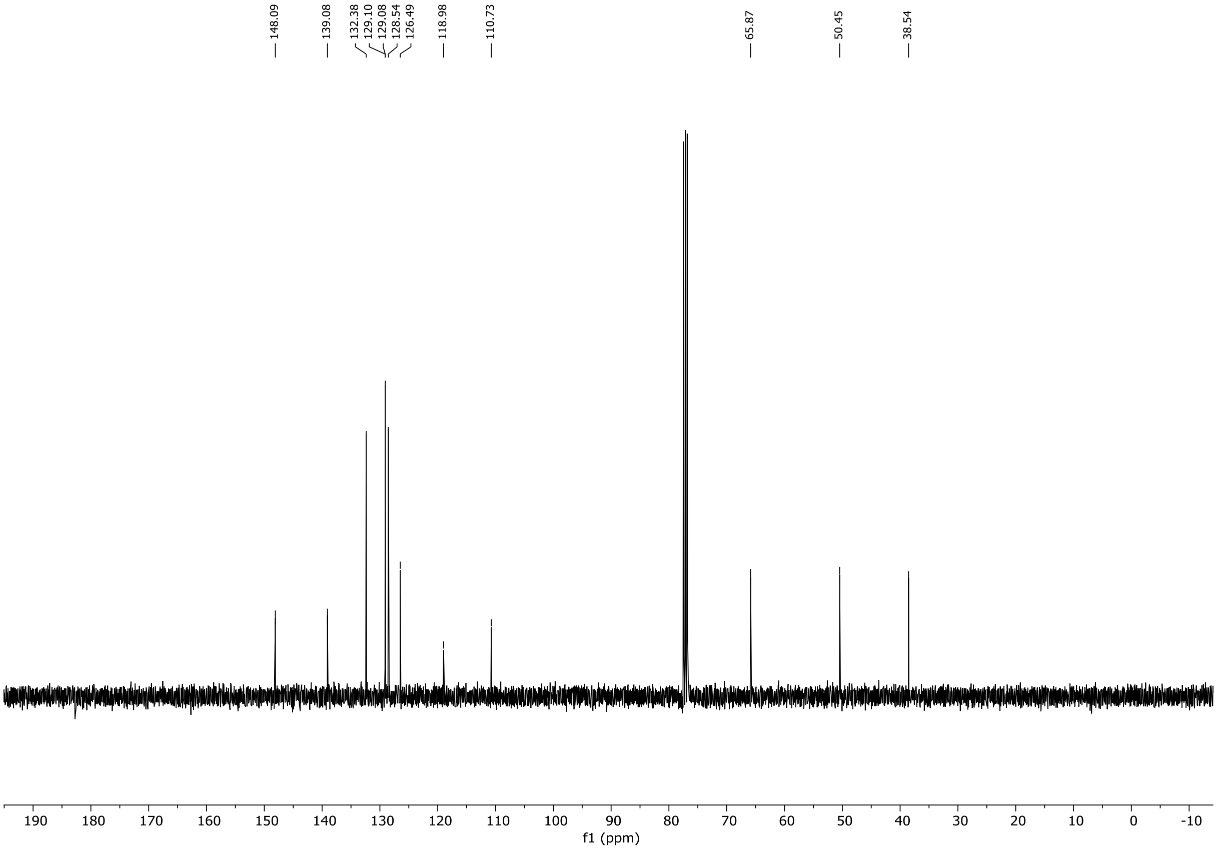

^{1}H NMR (400 MHz, $CDCl_3$) and ^{13}C NMR (100 MHz, $CDCl_3$); 3-phenyl-2-(*o*-tolyl)propan-1-ol (4e)

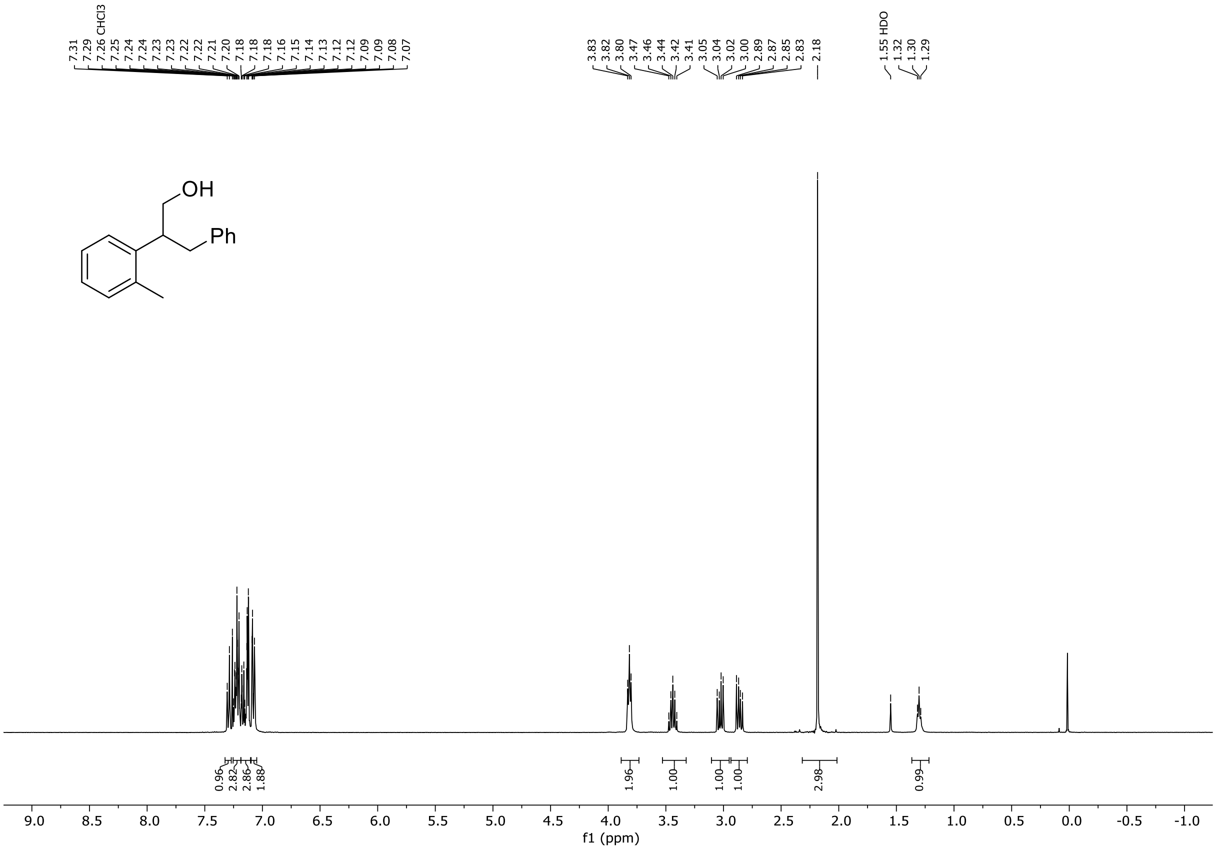

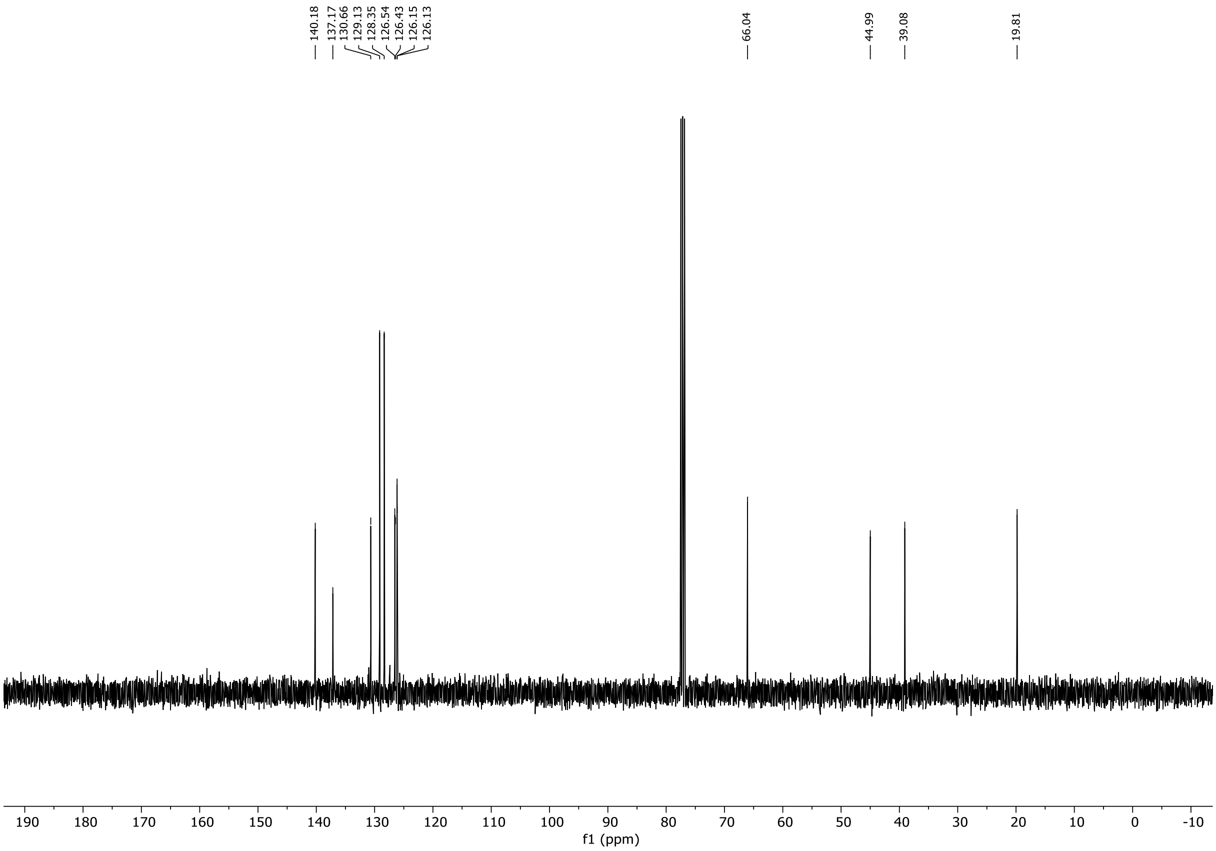

^{1}H NMR (400 MHz, $CDCl_3$) and ^{13}C NMR (100 MHz, $CDCl_3$); 2-(1*H*-indol-1-yl)-3-phenylpropan-1-ol (5)

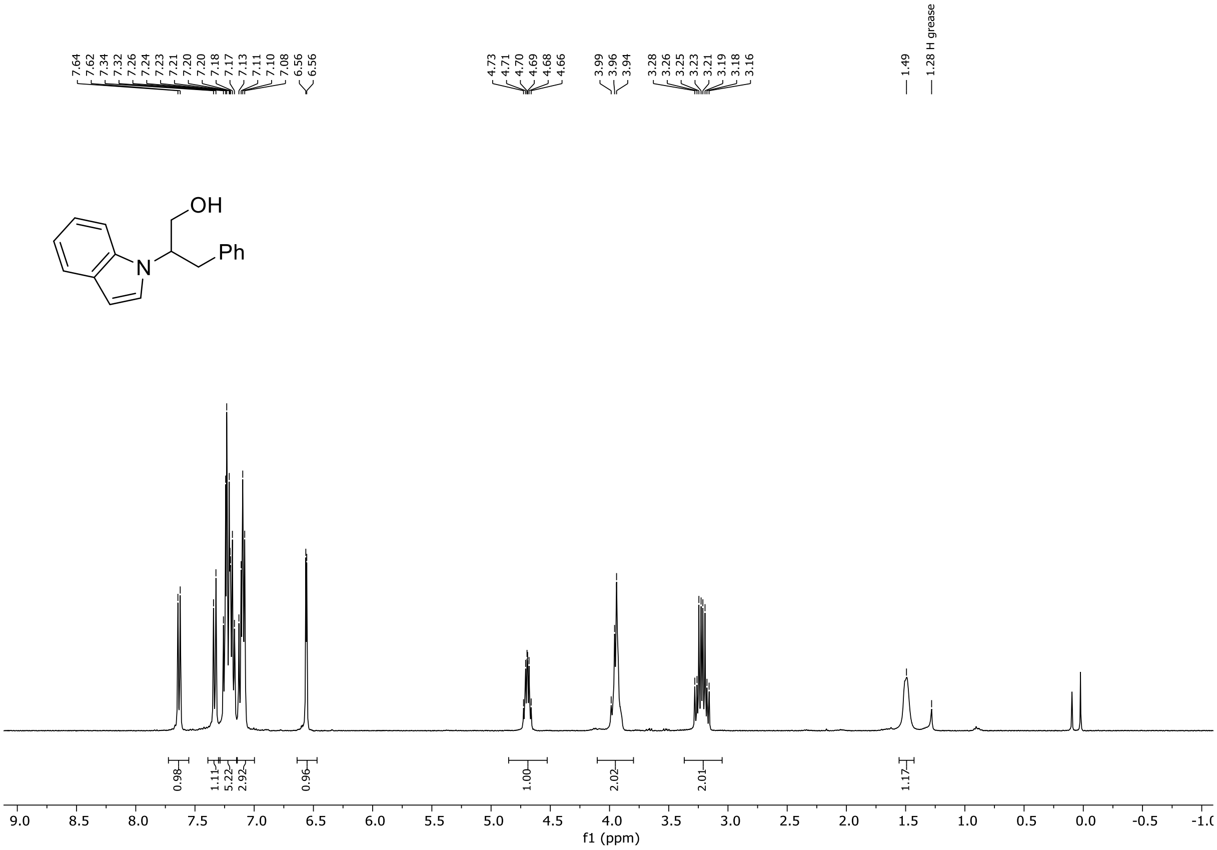

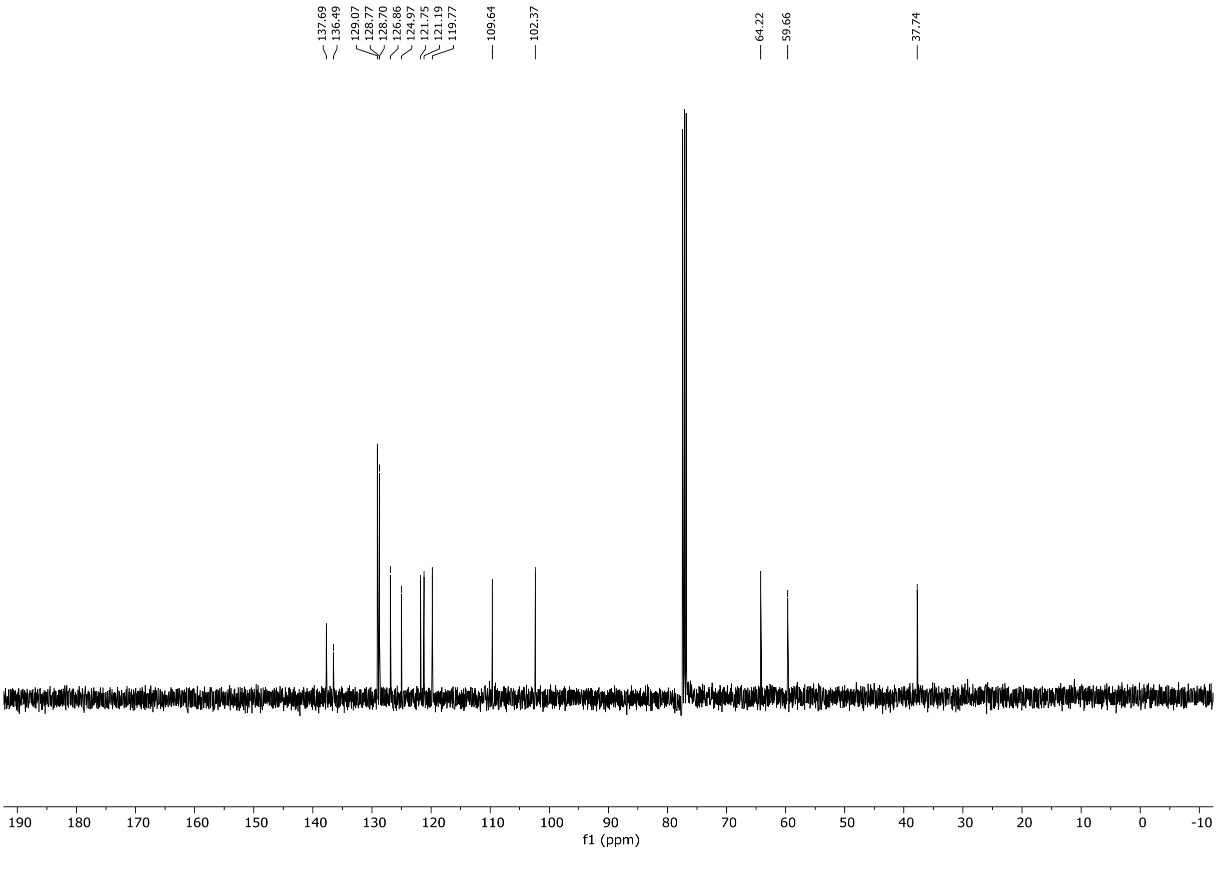

^{1}H NMR (400 MHz, $CDCl_3$) and ^{13}C NMR (100 MHz, $CDCl_3$); 2-(1-hydroxy-3-phenylpropan-2-yl)isoindoline-1,3-dione (6)

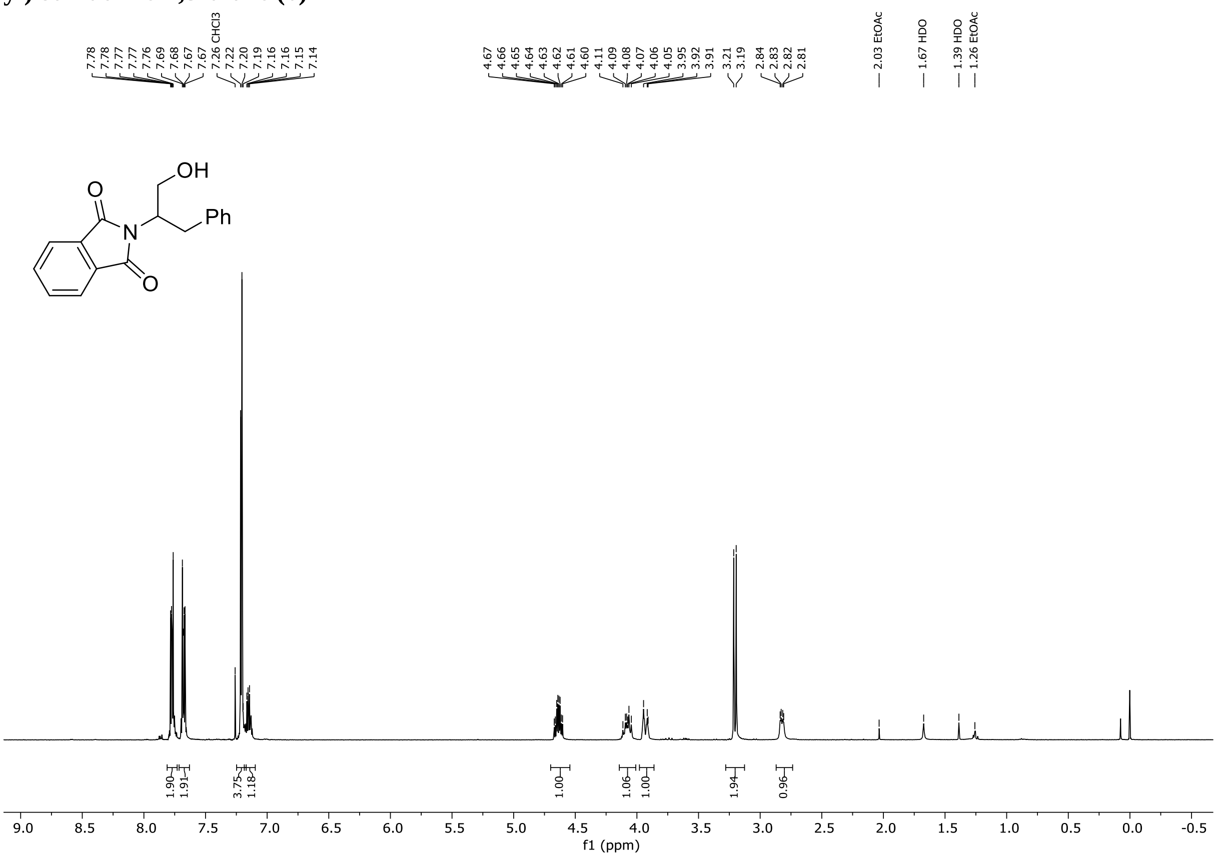

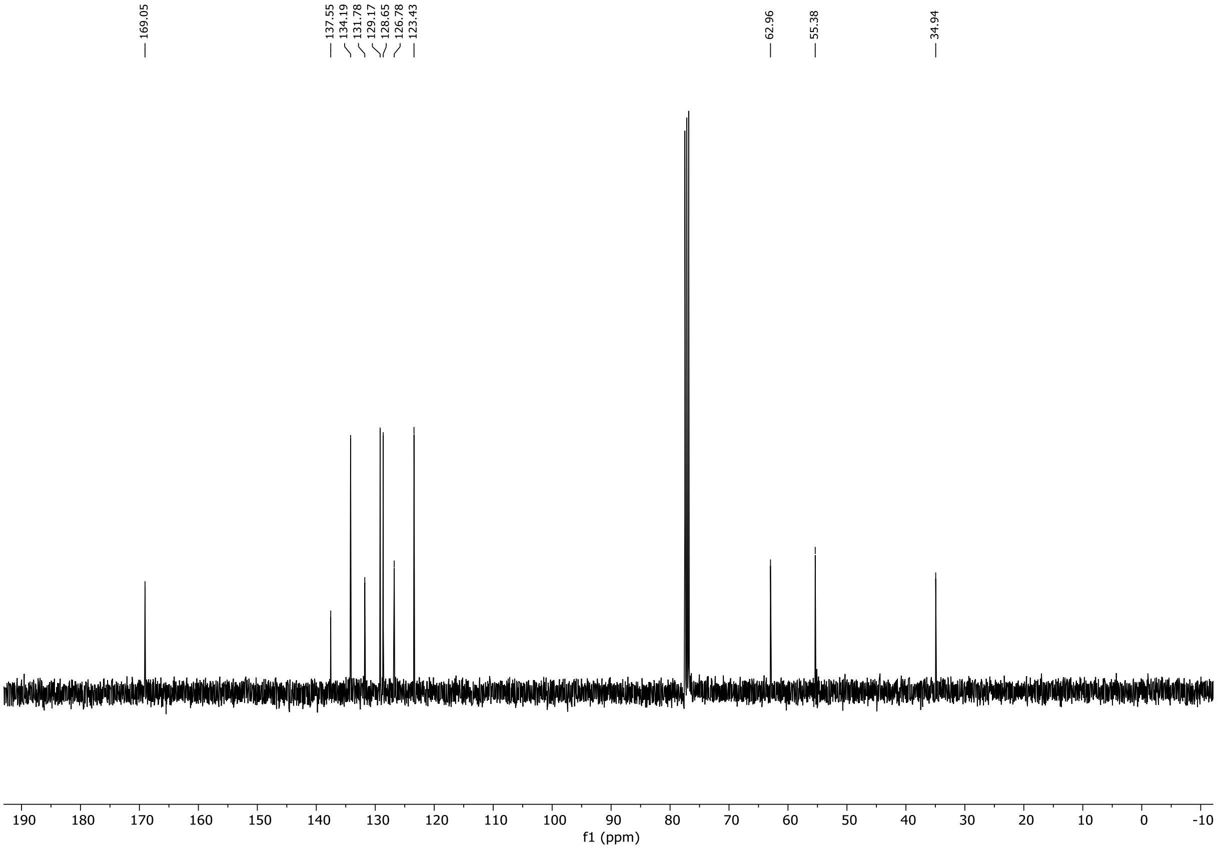

^{1}H NMR (400 MHz, CDCl$_3$) and ^{13}C NMR (100 MHz, CDCl$_3$); 2-(4-methoxyphenoxy)-3-phenylpropan-1ol (7)

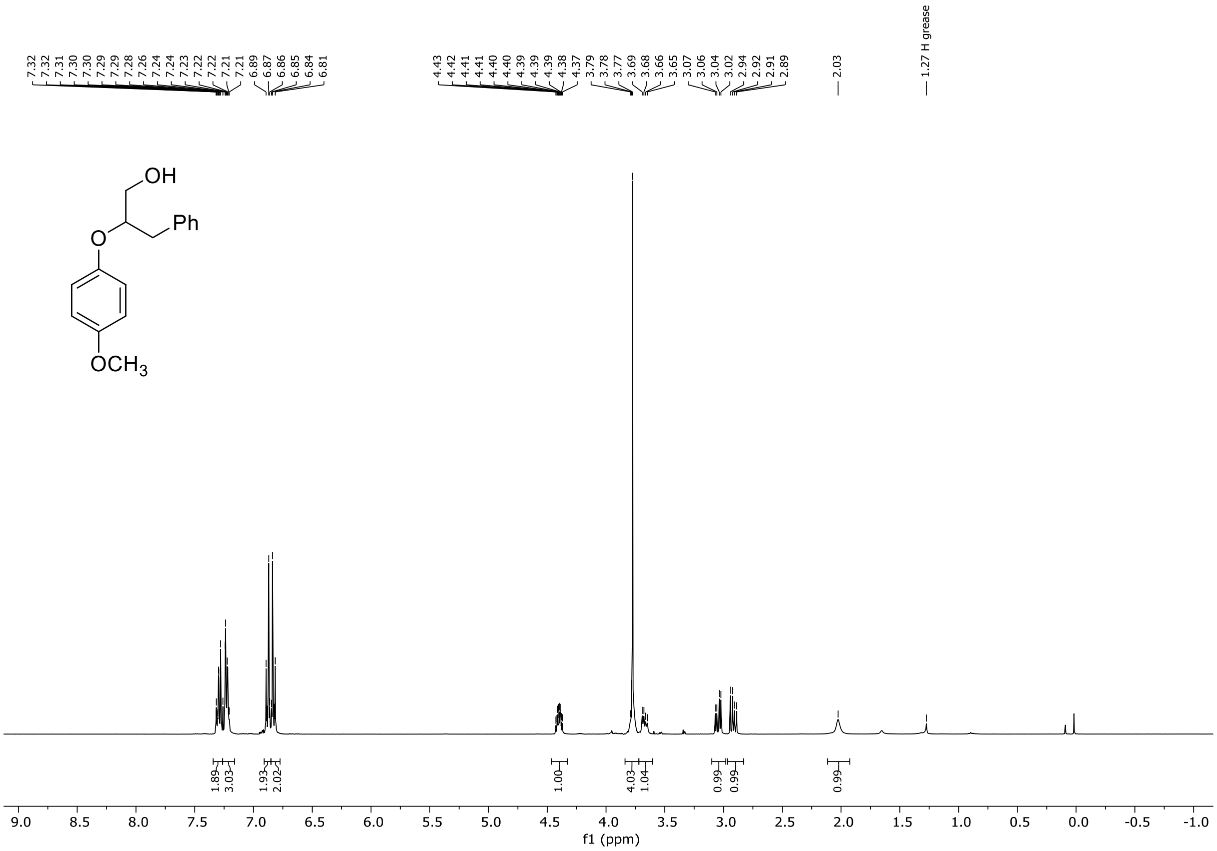

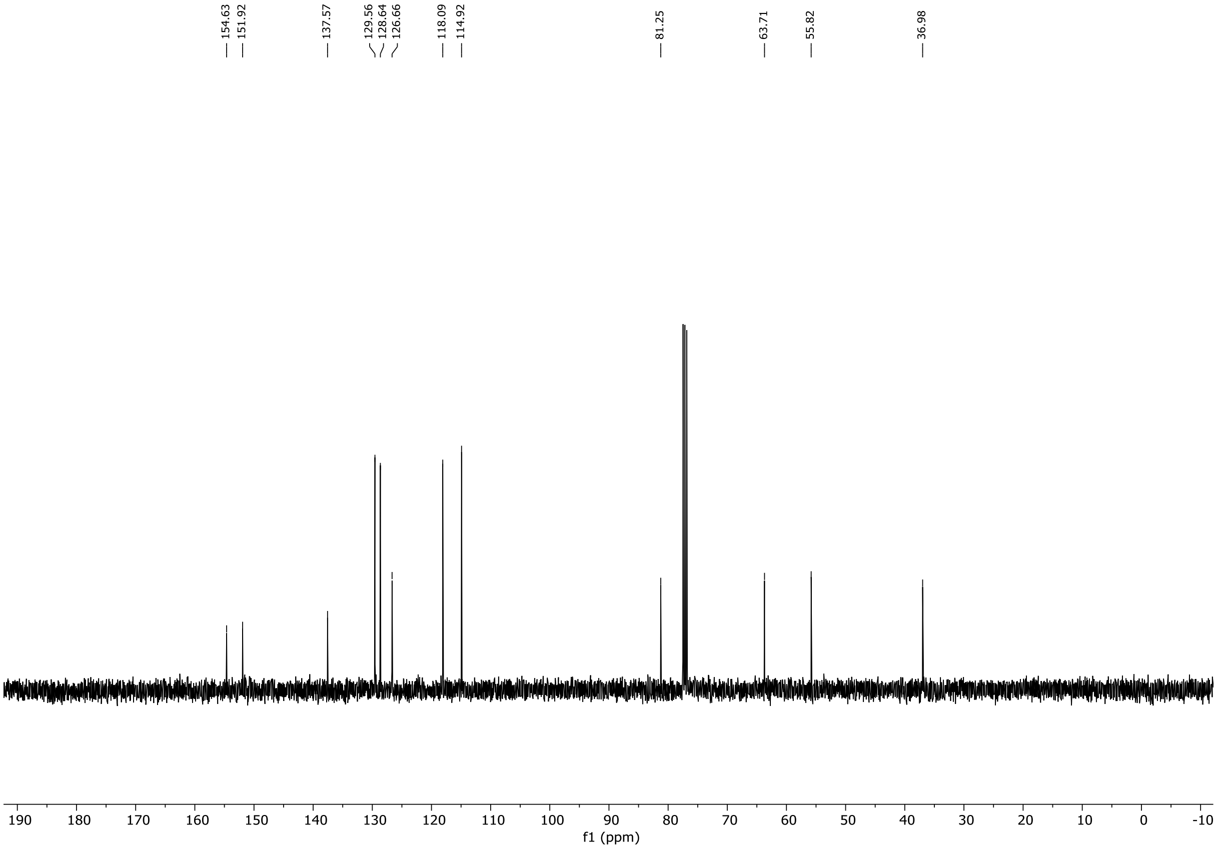

^{1}H NMR (400 MHz, $CDCl_3$) and ^{13}C NMR (100 MHz, $CDCl_3$); 2-benzyl-3-(benzyloxy)propan-1-ol (8)

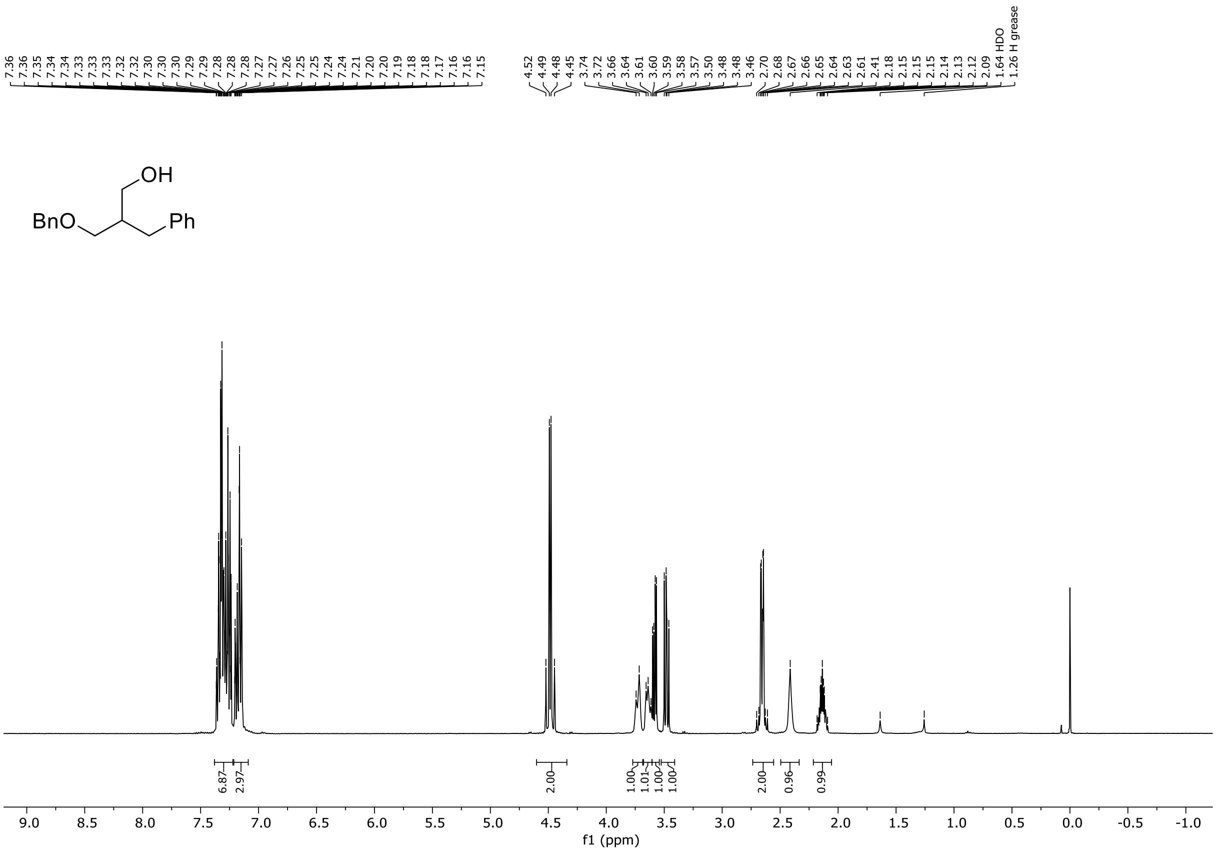

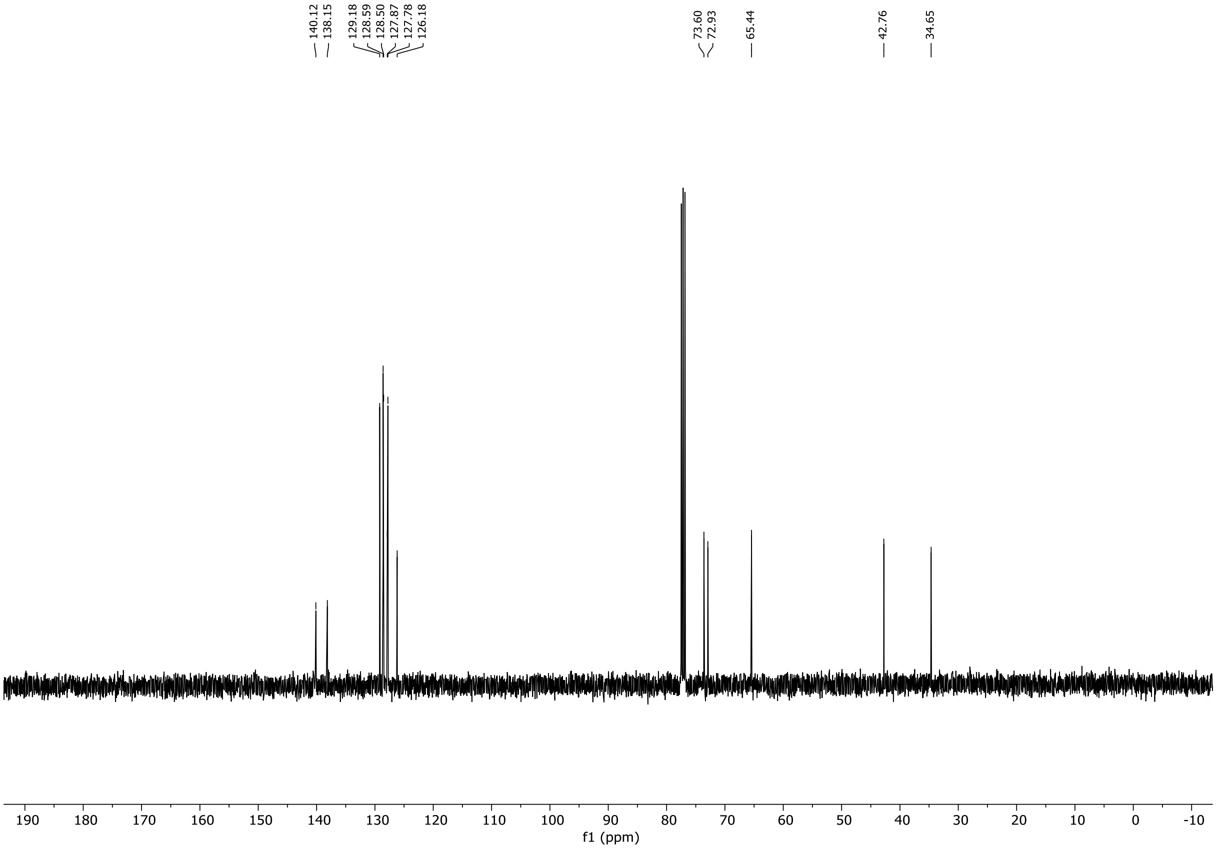

^{1}H NMR (400 MHz, $CDCl_3$) and ^{13}C NMR (100 MHz, $CDCl_3$); 2-benzyl-3-(benzyloxy)-2-methylpropan-1-ol (9)

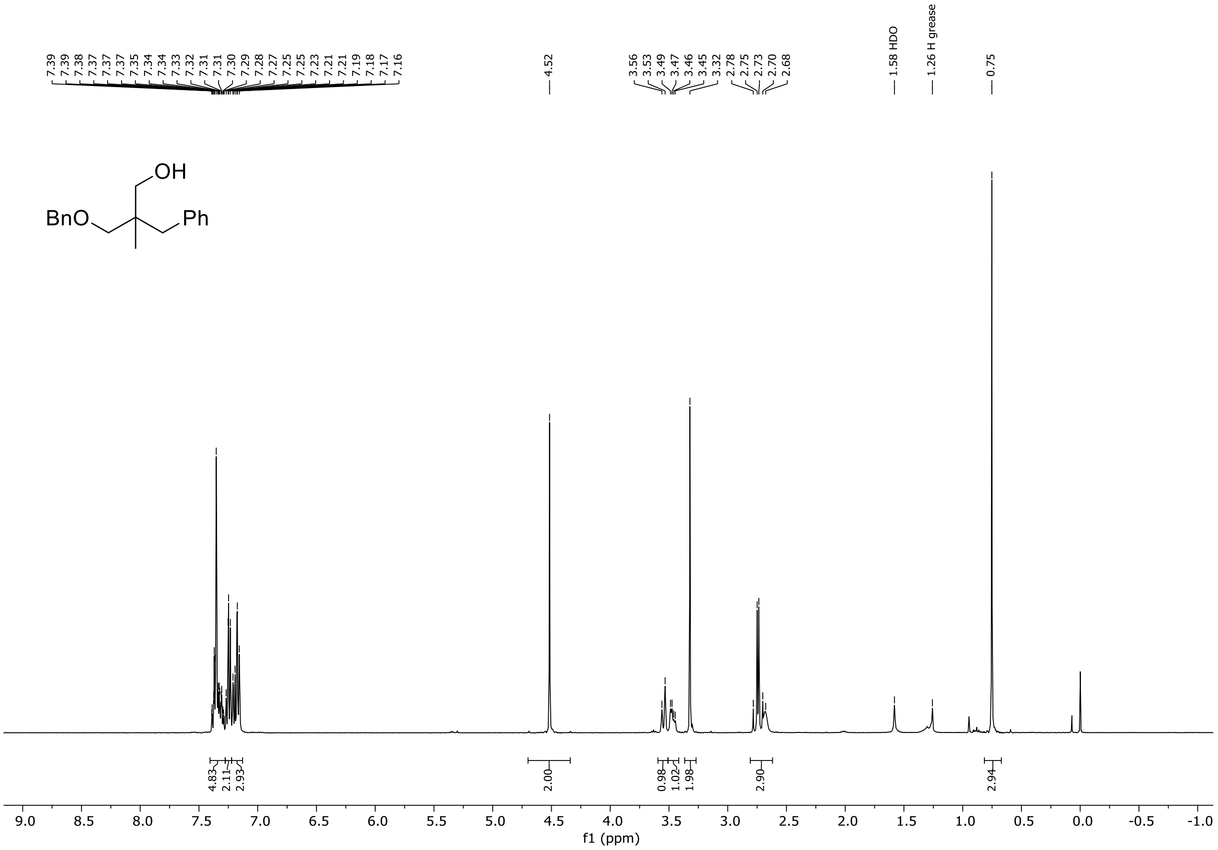

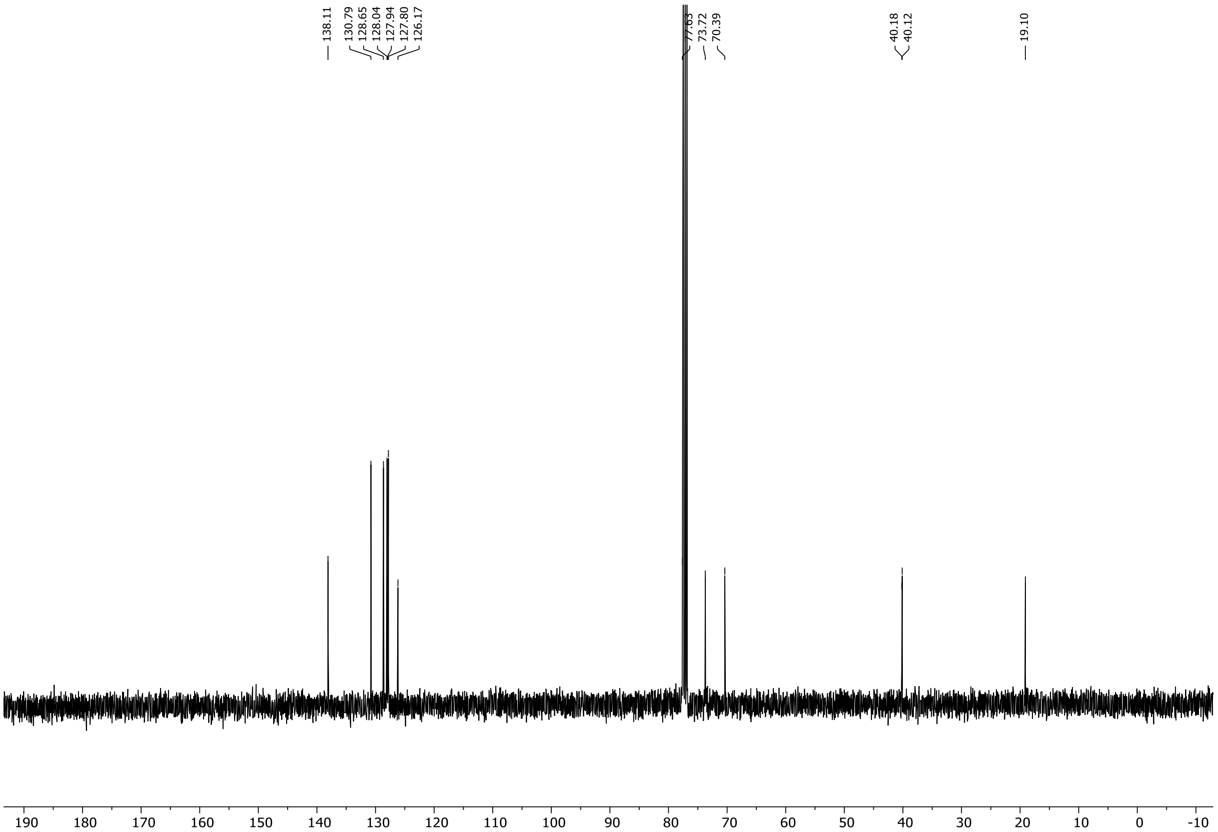

^{1}H NMR (400 MHz, $CDCl_3$) and ^{13}C NMR (100 MHz, $CDCl_3$); 1-(benzyloxy)-5-phenylpentan-3-ol (10)

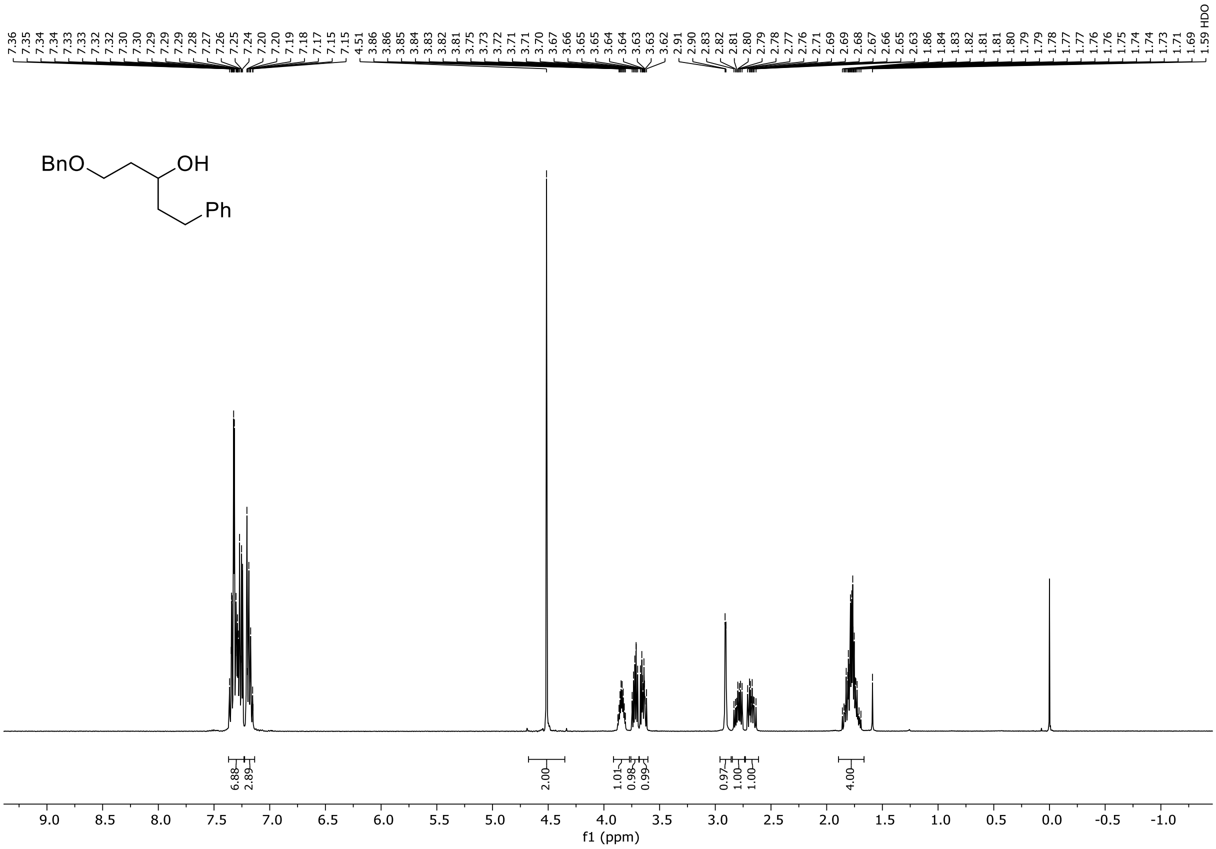

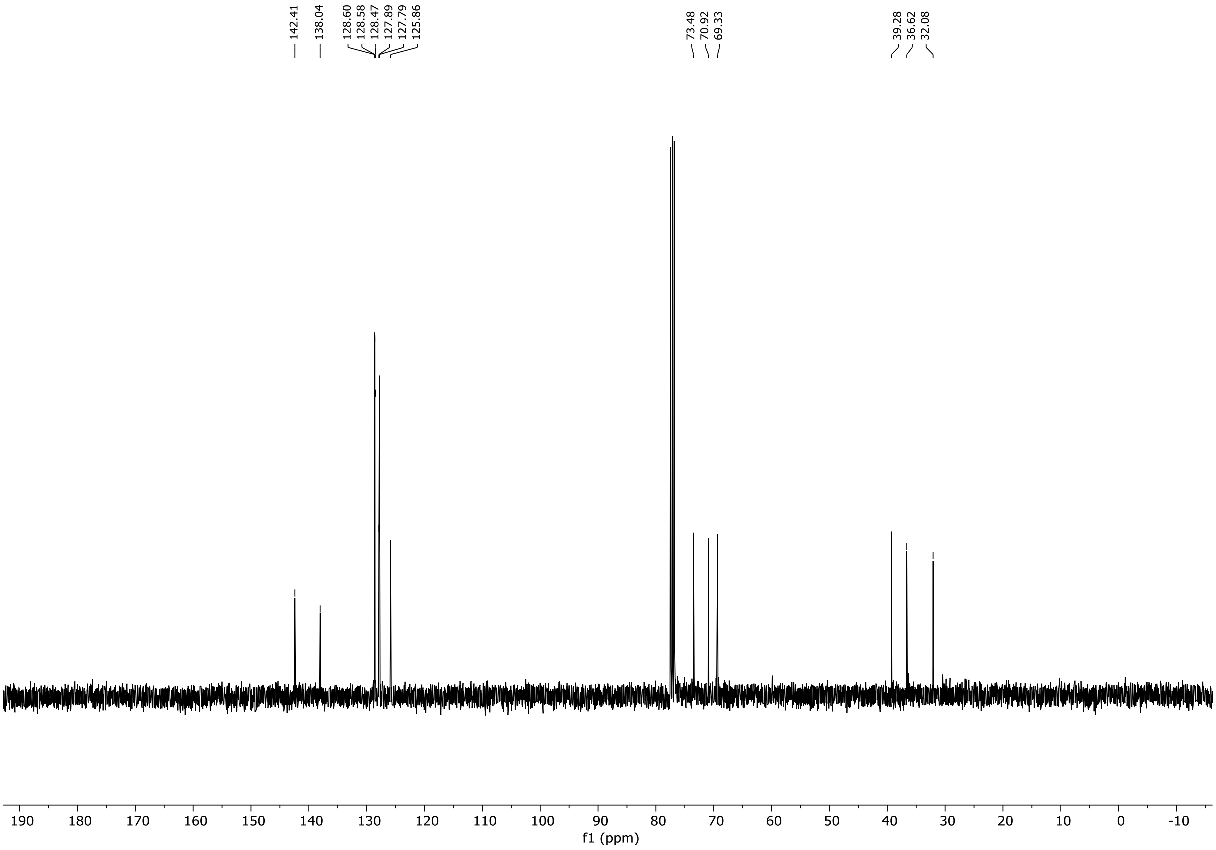

^{1}H NMR (400 MHz, CDCl$_3$) and ^{13}C NMR (100 MHz, CDCl$_3$); 1-phenyl-5-((triisopropylsilyl)oxy)pentan-3-ol (11)

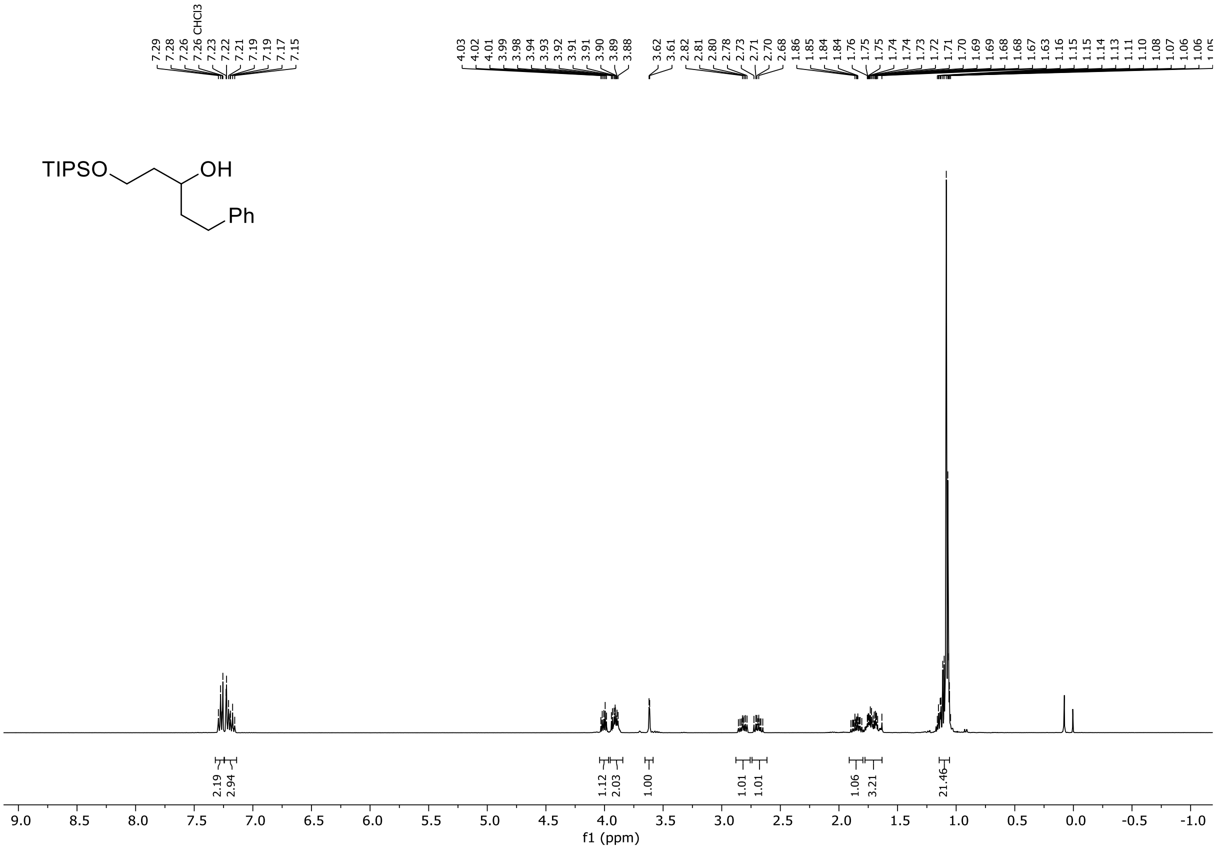

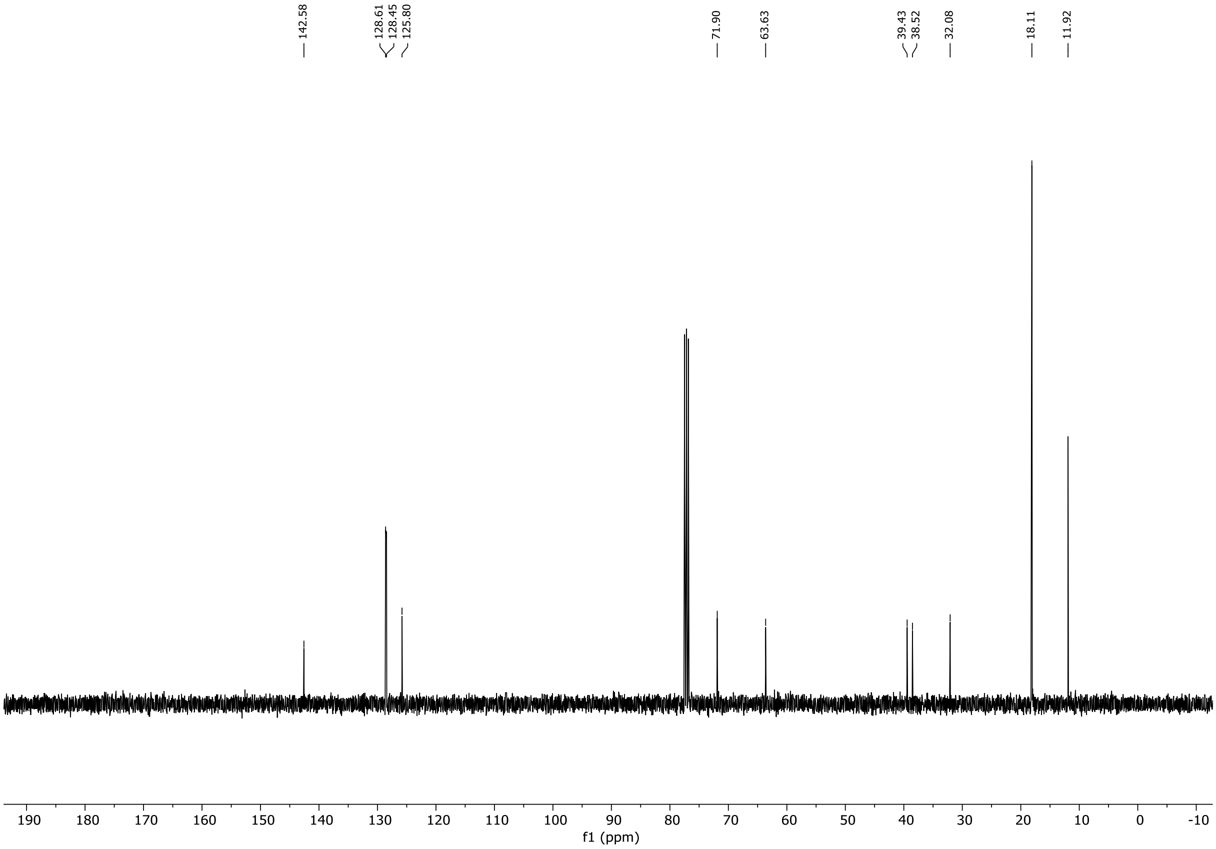

^{1}H NMR (400 MHz, CDCl$_3$) and ^{13}C NMR (100 MHz, CDCl$_3$); 2-phenyl-3-(4-methylphenyl)propan-1-ol (4a)

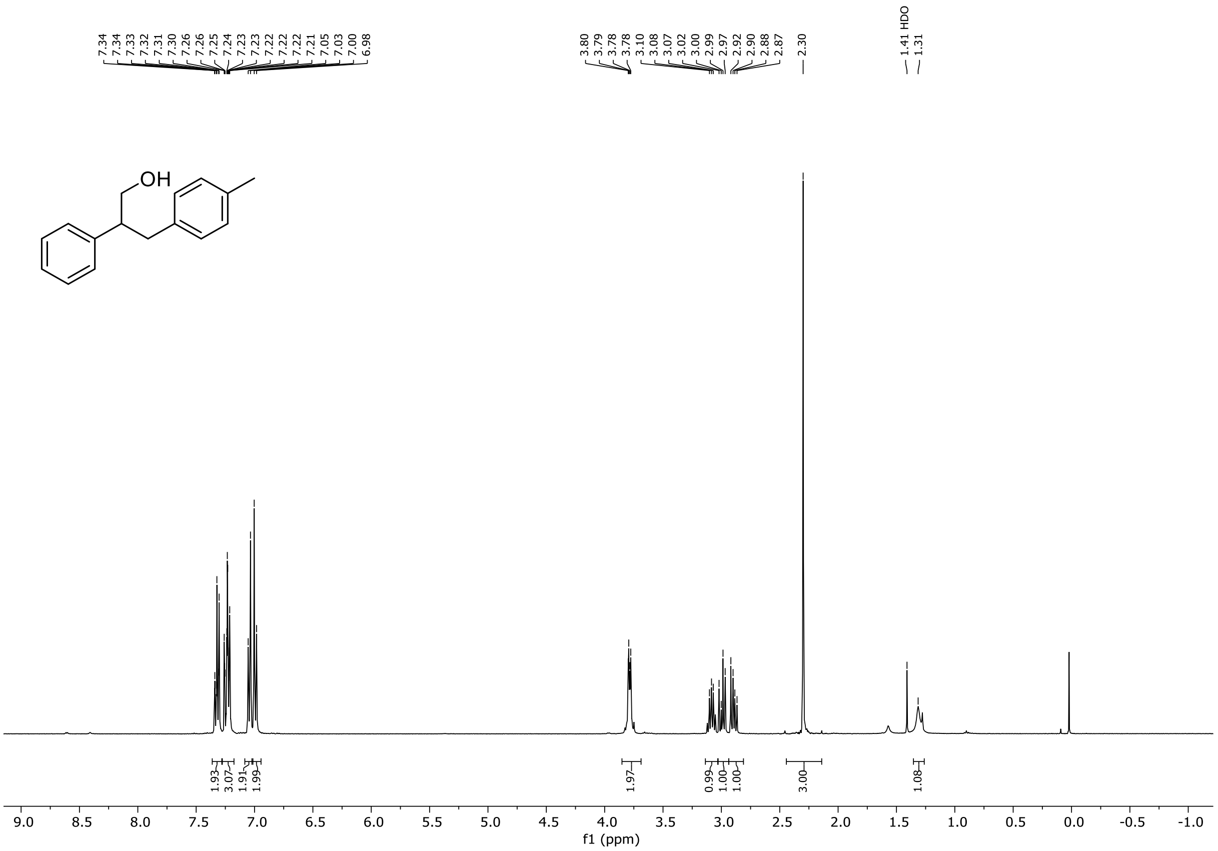

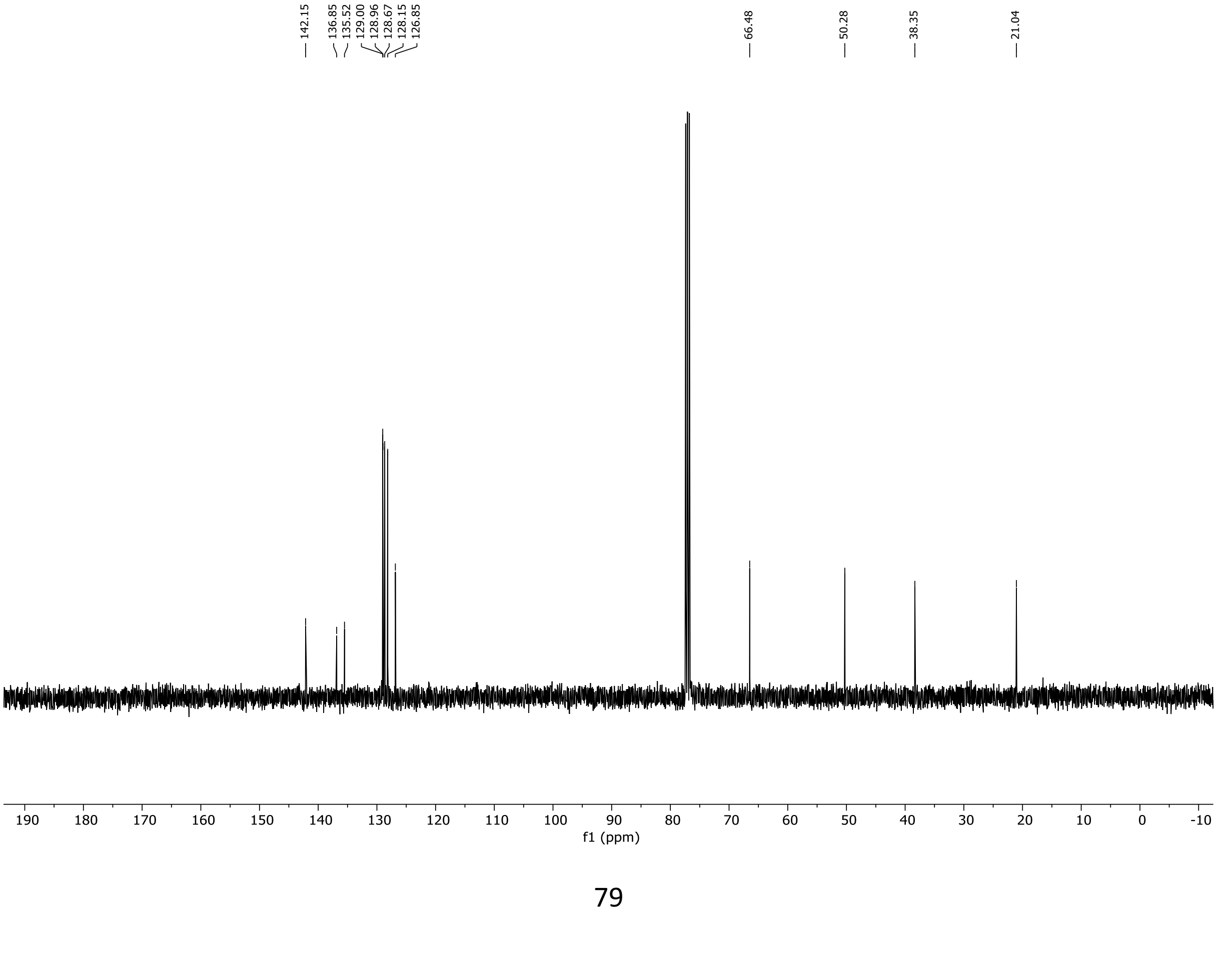

^{1}H NMR (400 MHz, CDCl3) and ^{13}C NMR (100 MHz, CDCl3); 3-(4-methoxyphenyl)-2-phenylpropan-1-ol (12a)

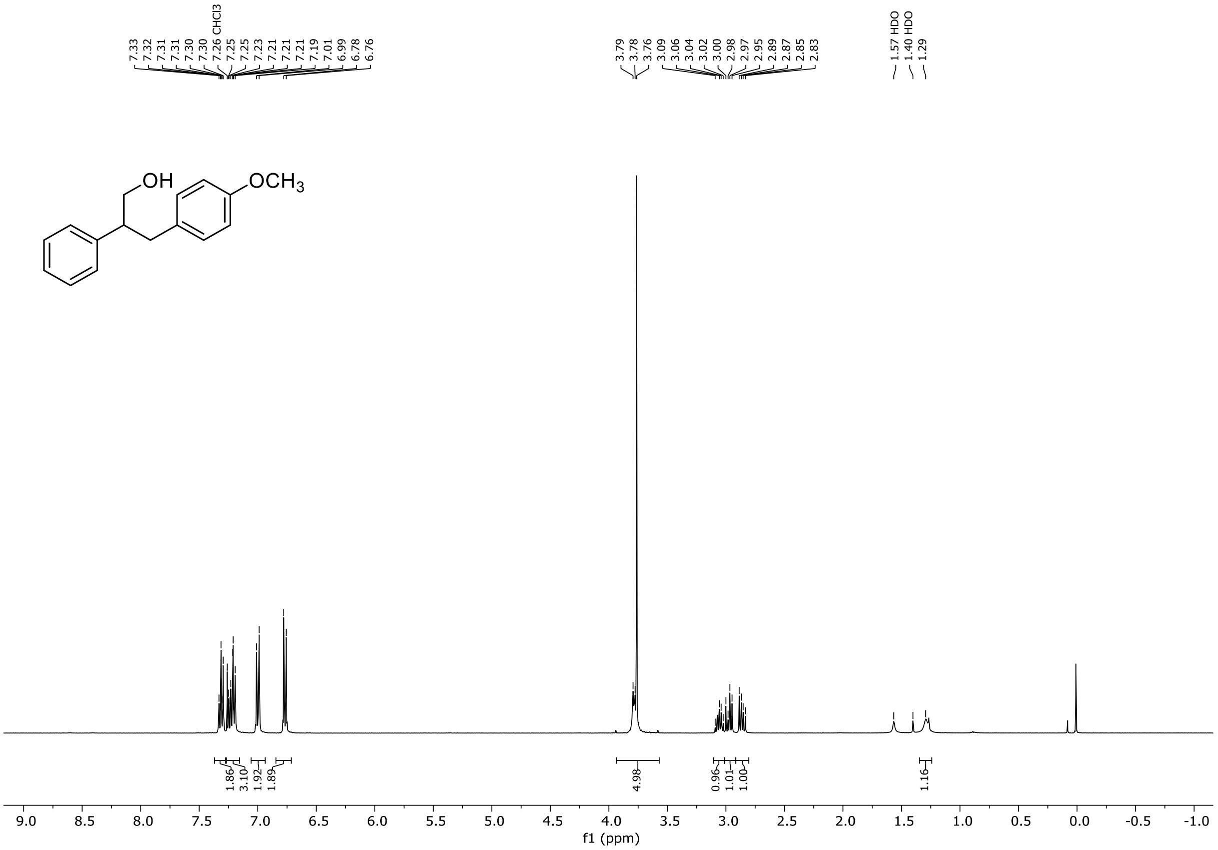

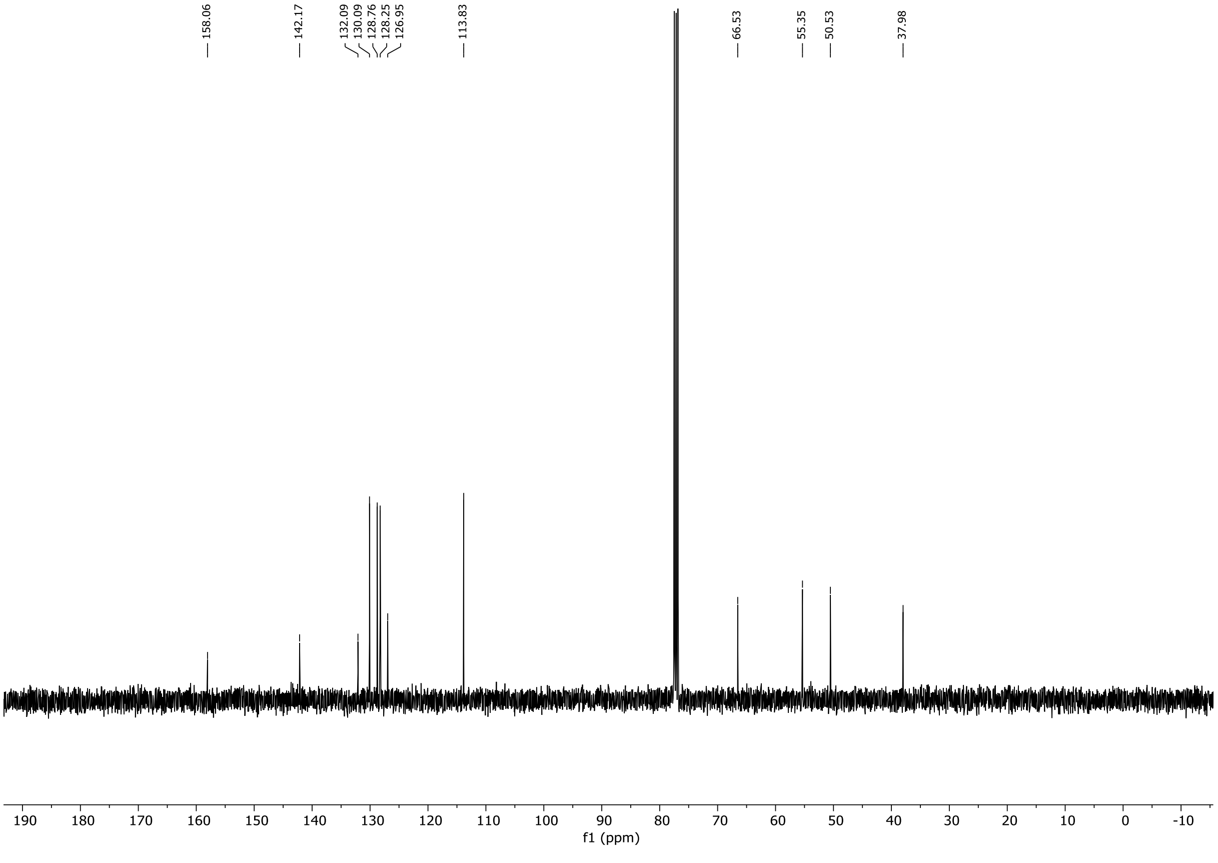

^{1}H NMR (400 MHz, $CDCl_3$) and ^{13}C NMR (100 MHz, $CDCl_3$); 4-(3-hydroxy-2-phenylpropyl)benzonitrile (12b)

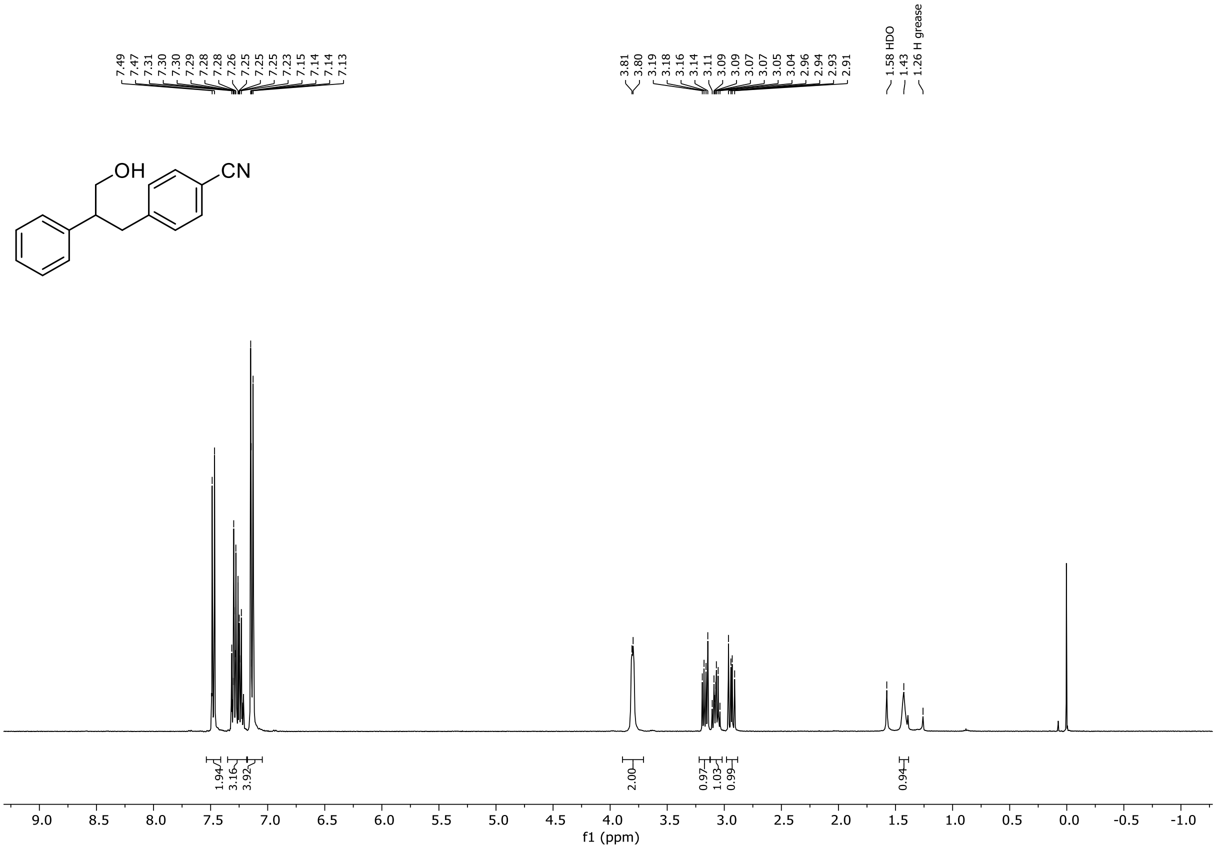

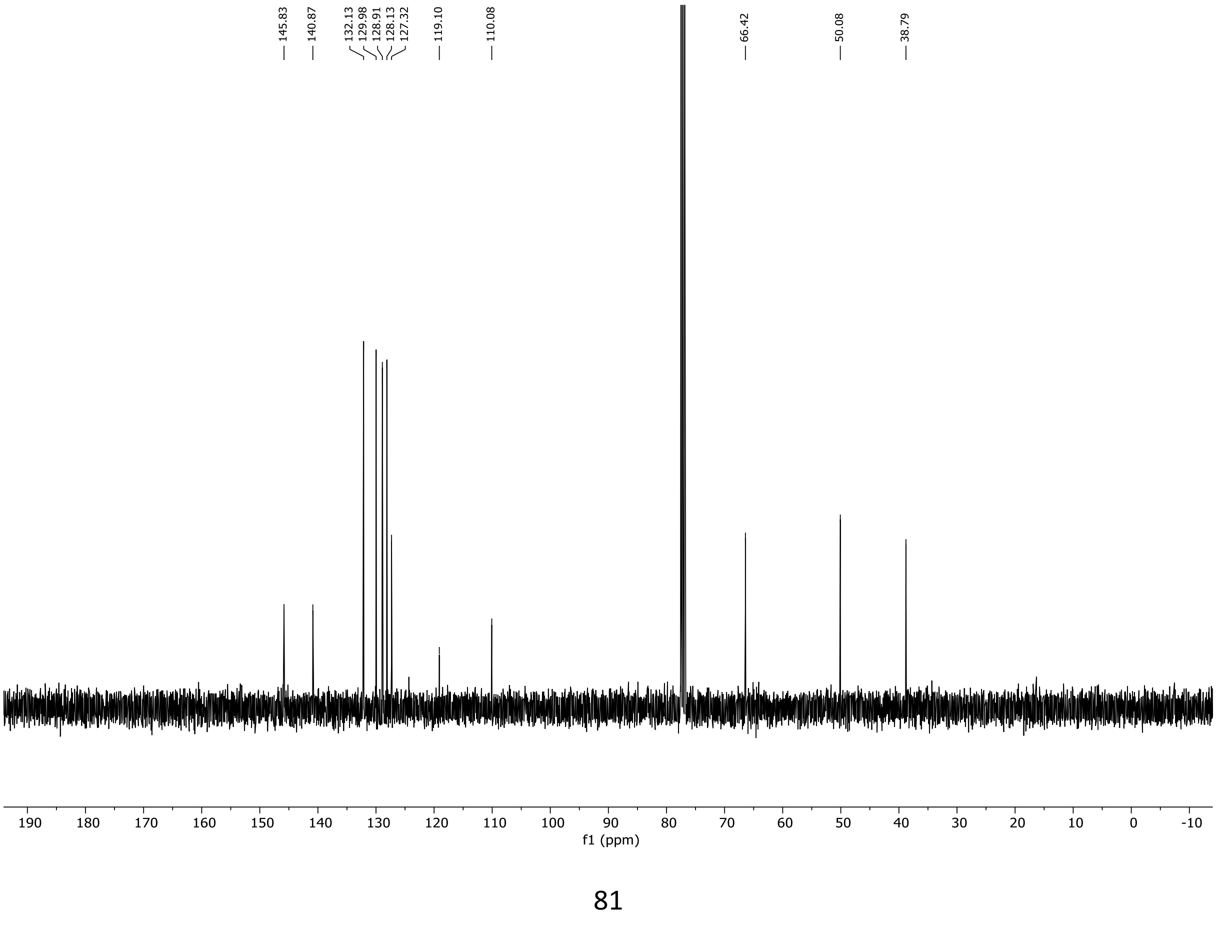

^{1}H NMR (400 MHz, CDCl3), ^{13}C NMR (100 MHz, CDCl3) and ^{19}F NMR (376 MHz, CDCl3); 2-phenyl-3-(4-(trifluoromethyl)phenyl)propan-1-ol (12c)

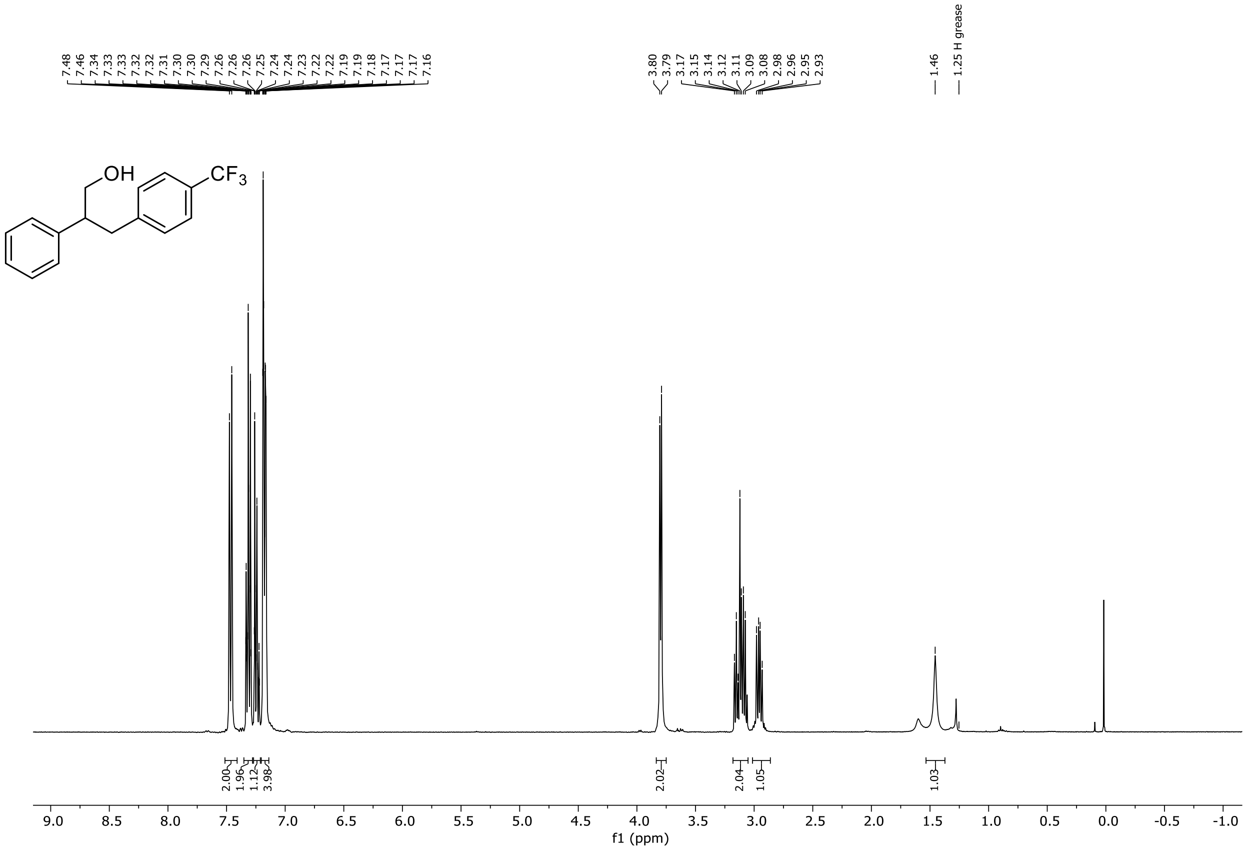

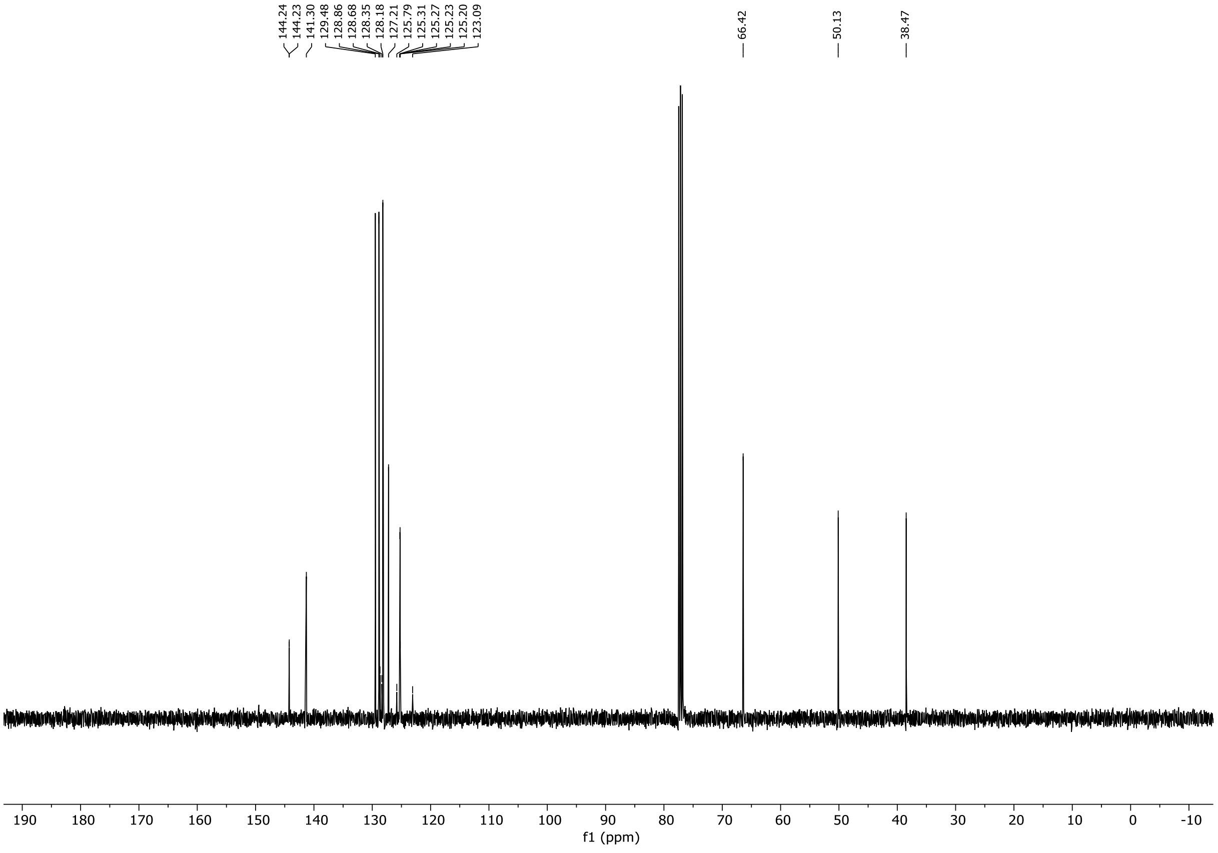

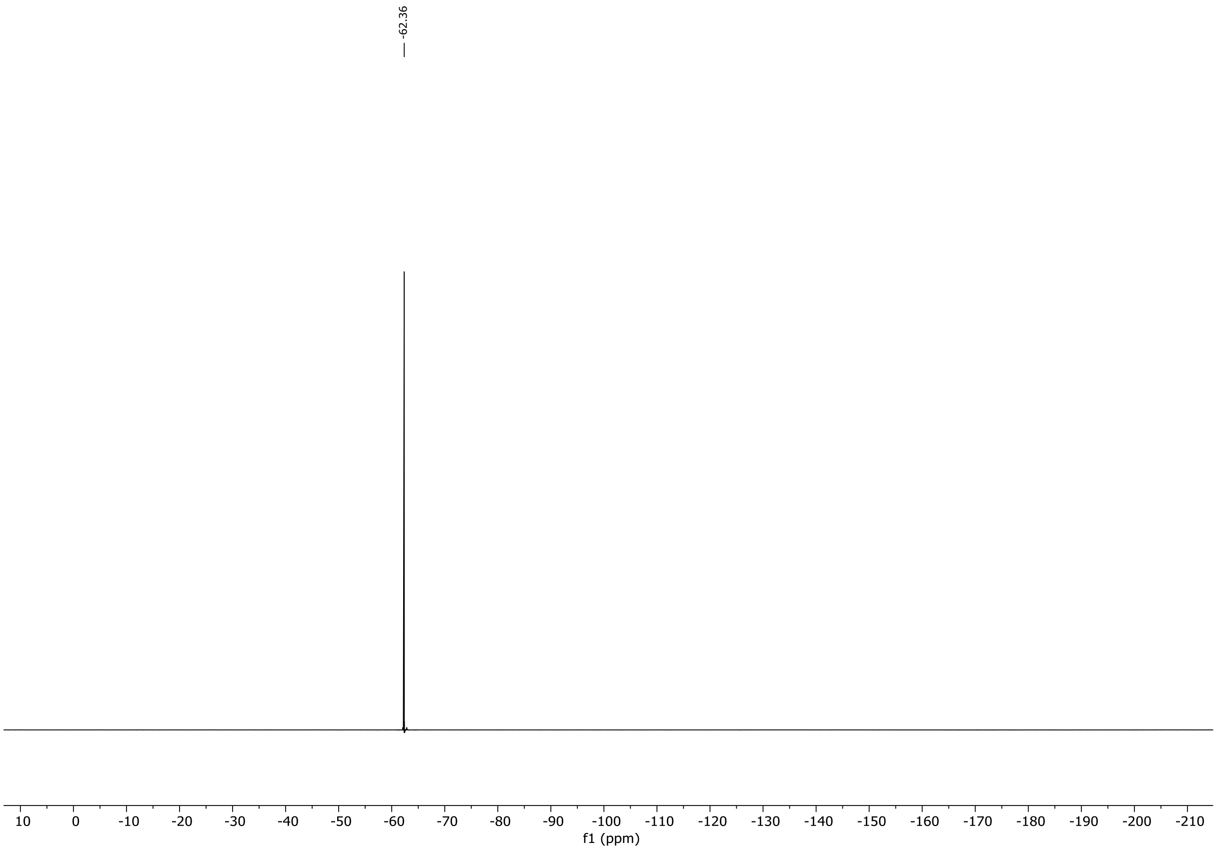
-62.36
10
0
-10
-20
-30
-40
-50
-60
-70
-80
-90
-100
-110
-120
-130
-140
-150
-160
-170
-180
-190
-200
-210
f1 (ppm)

^{1}H NMR (400 MHz, $CDCl_3$) and ^{13}C NMR (100 MHz, $CDCl_3$); 1-(4-(3-hydroxy-2-phenylpropyl)phenyl)ethanone (12d)

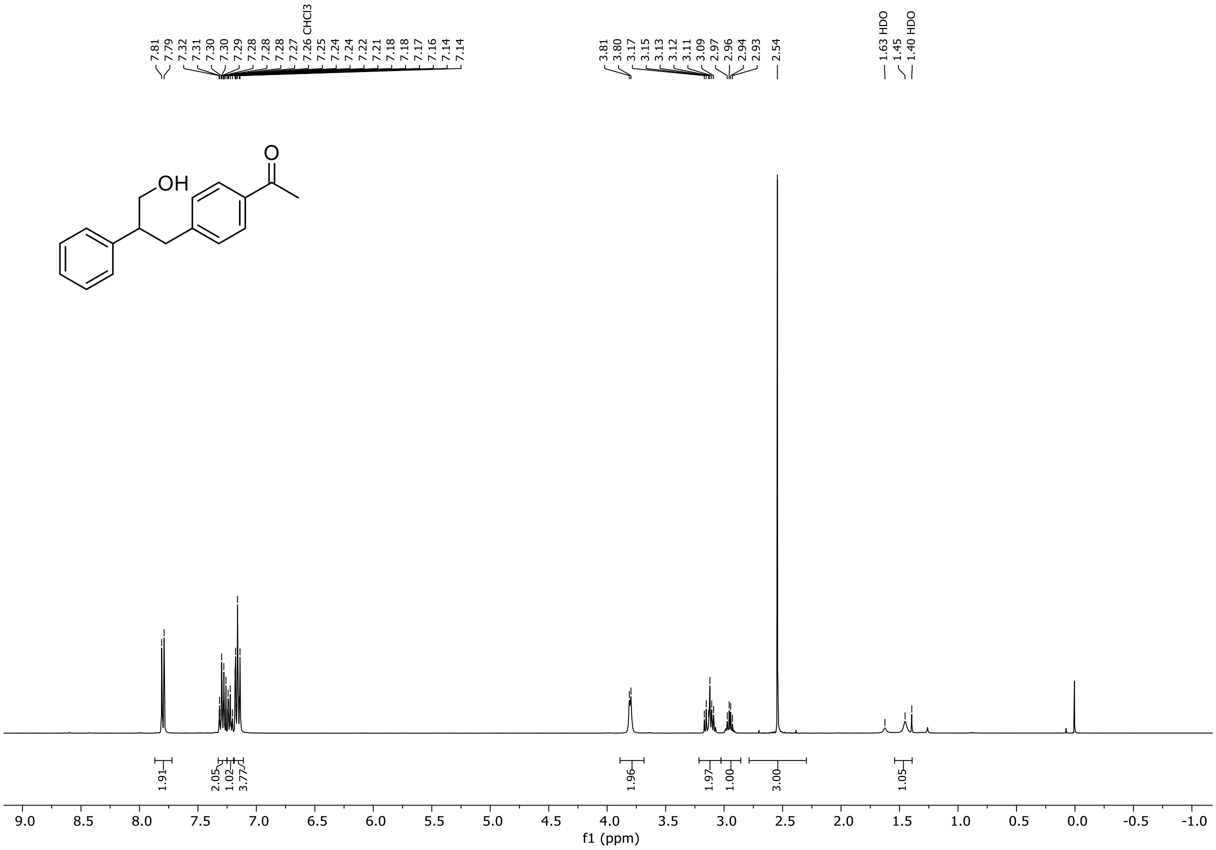

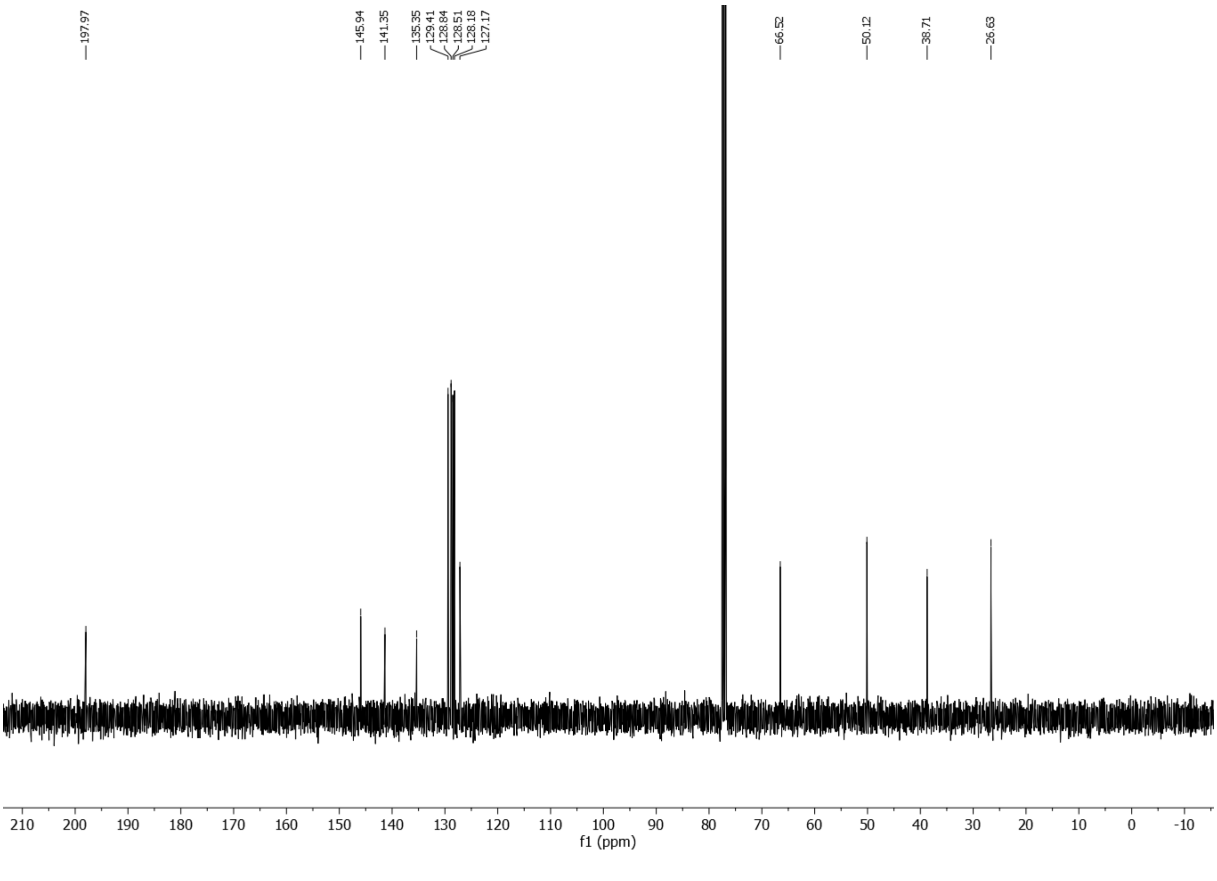

^{1}H NMR (400 MHz, CDCl3) and ^{13}C NMR (100 MHz, CDCl3); methyl 4-(3-hydroxy-2-phenylpropyl)benzoate (12e)

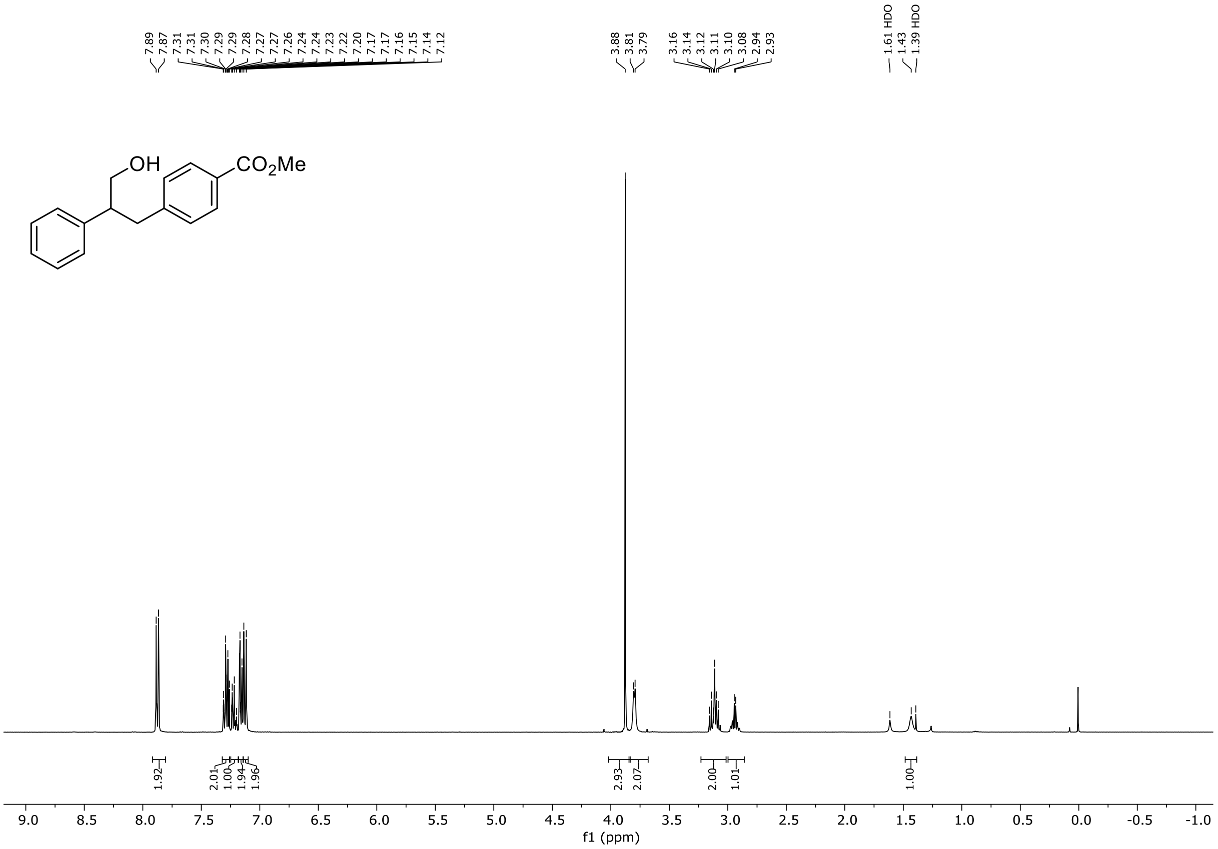

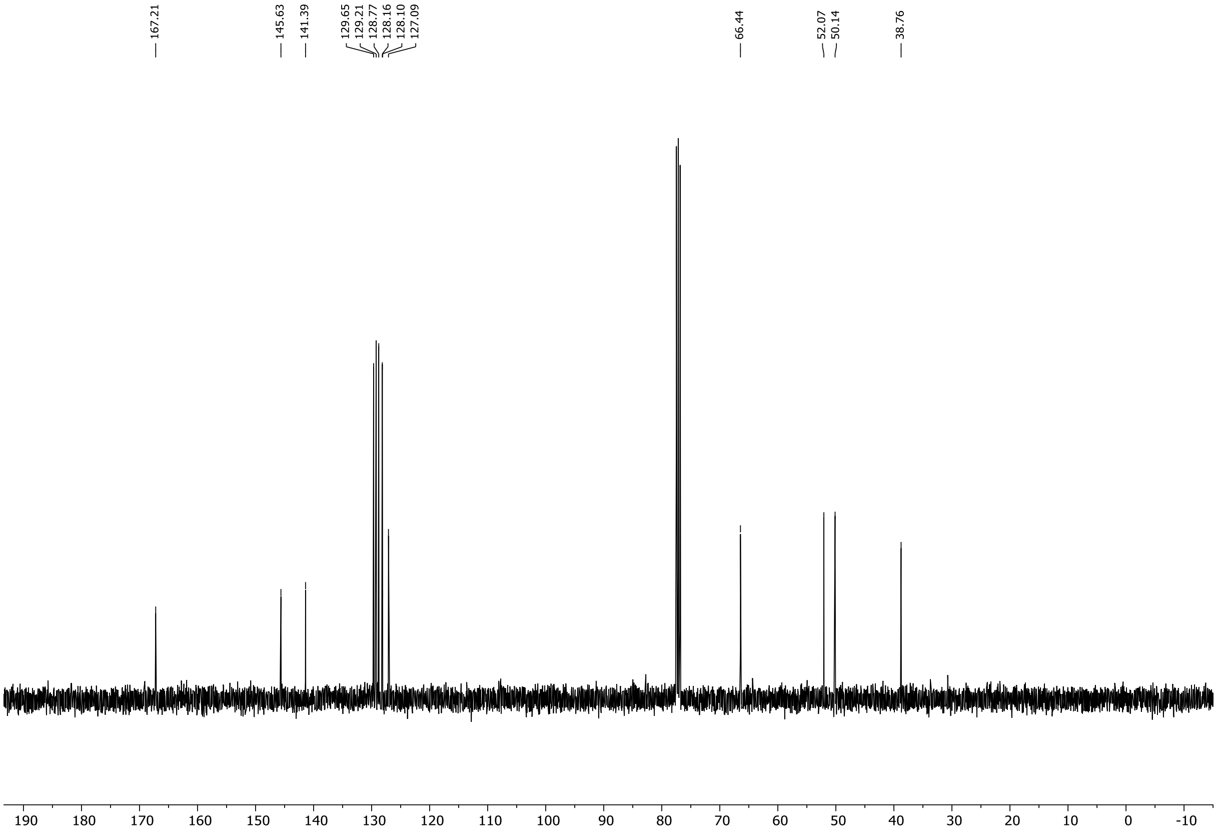

^{1}H NMR (400 MHz, $CDCl_3$) and ^{13}C NMR (100 MHz, $CDCl_3$); 3-(4-chlorophenyl)-2-phenylpropan-1-ol (12f)

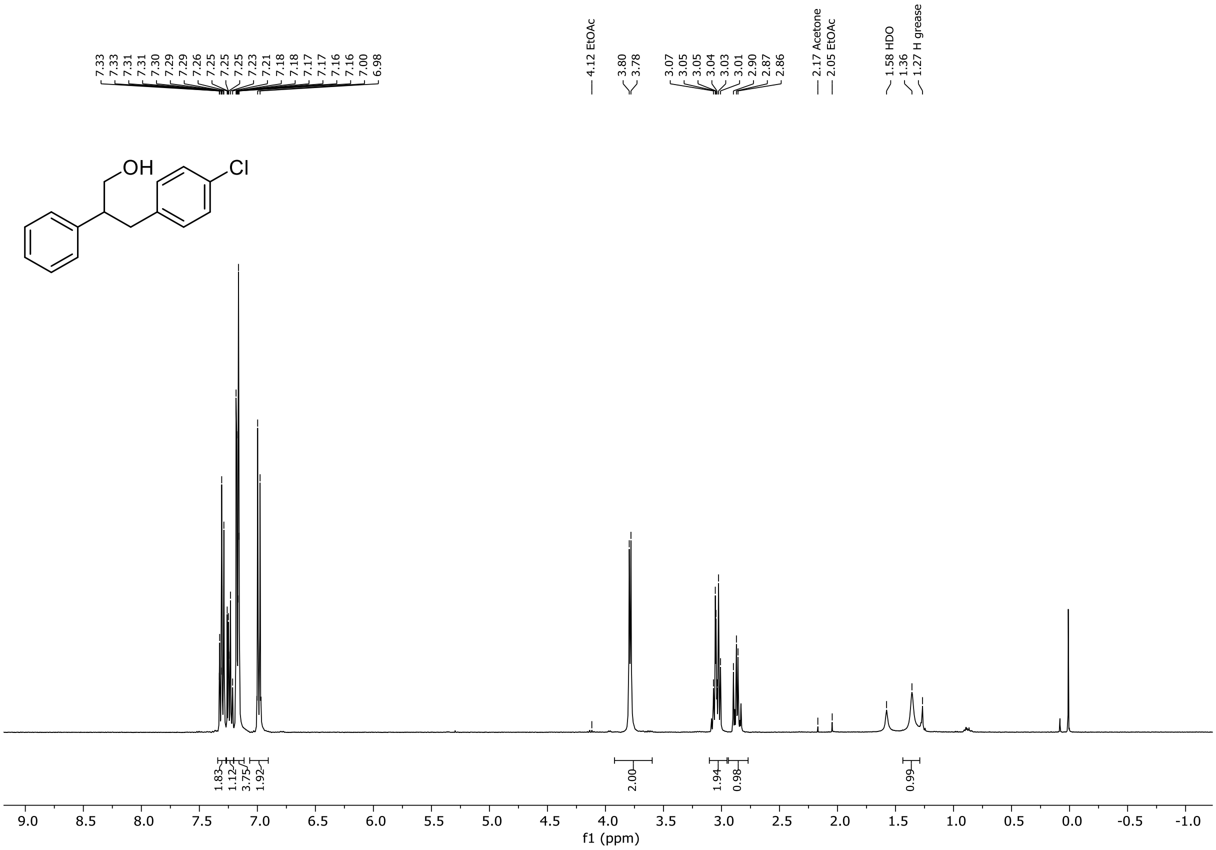

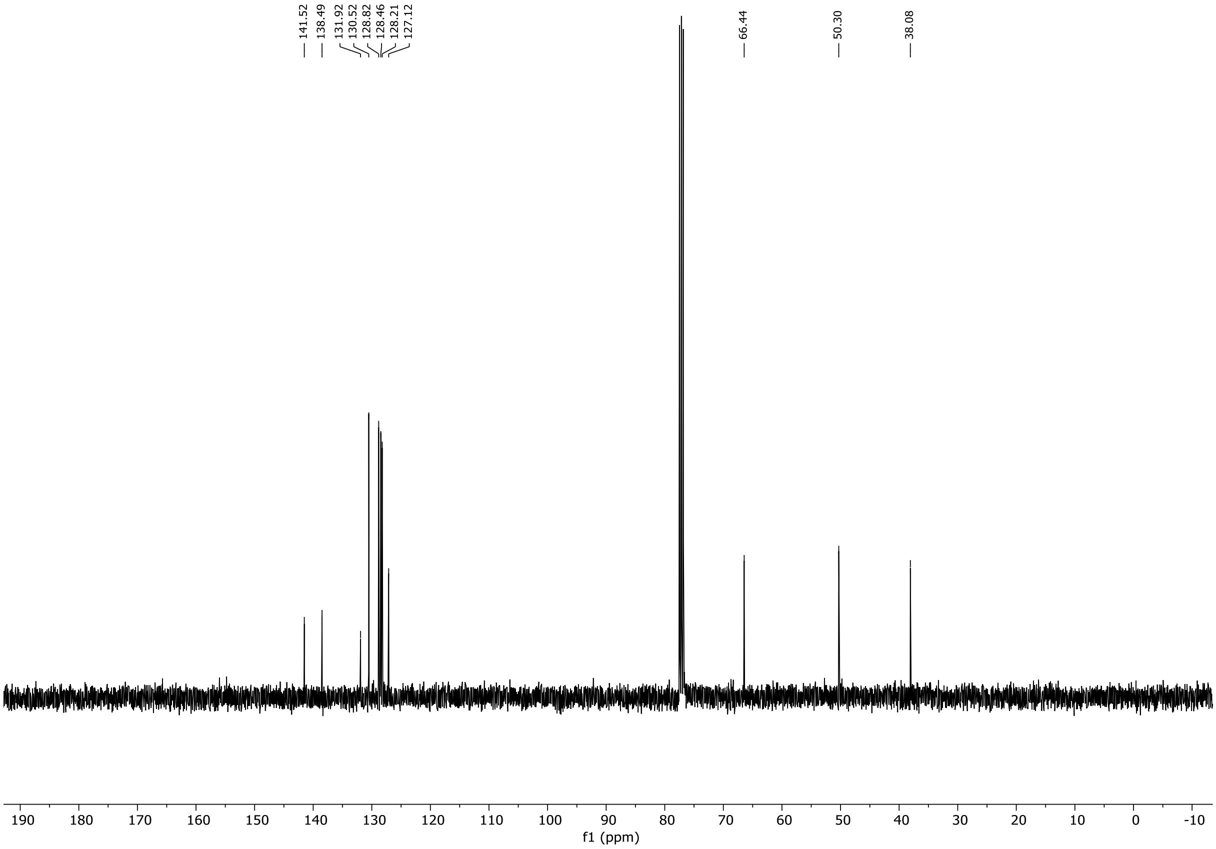

^{1}H NMR (400 MHz, $CDCl_3$) and ^{13}C NMR (100 MHz, $CDCl_3$); 3-(4-bromophenyl)-2-phenylpropan-1-ol (12g)

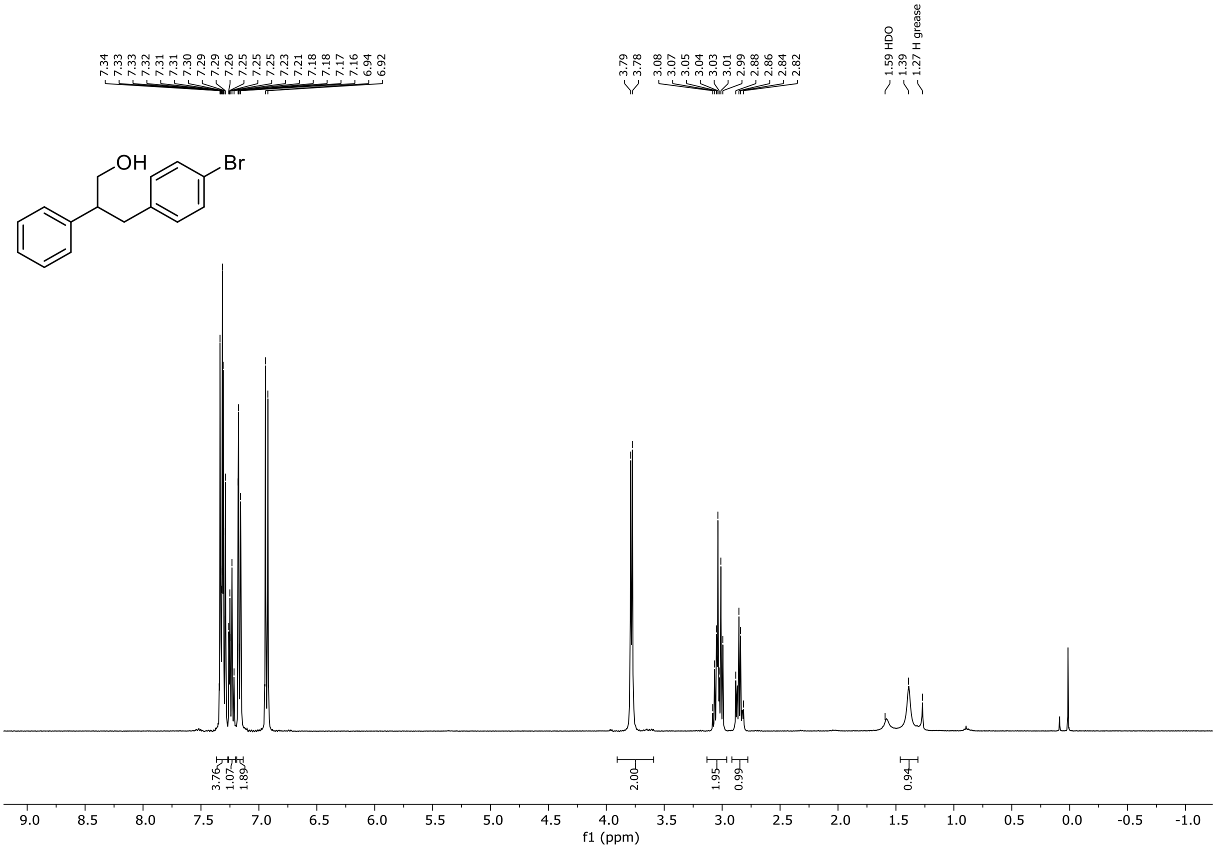

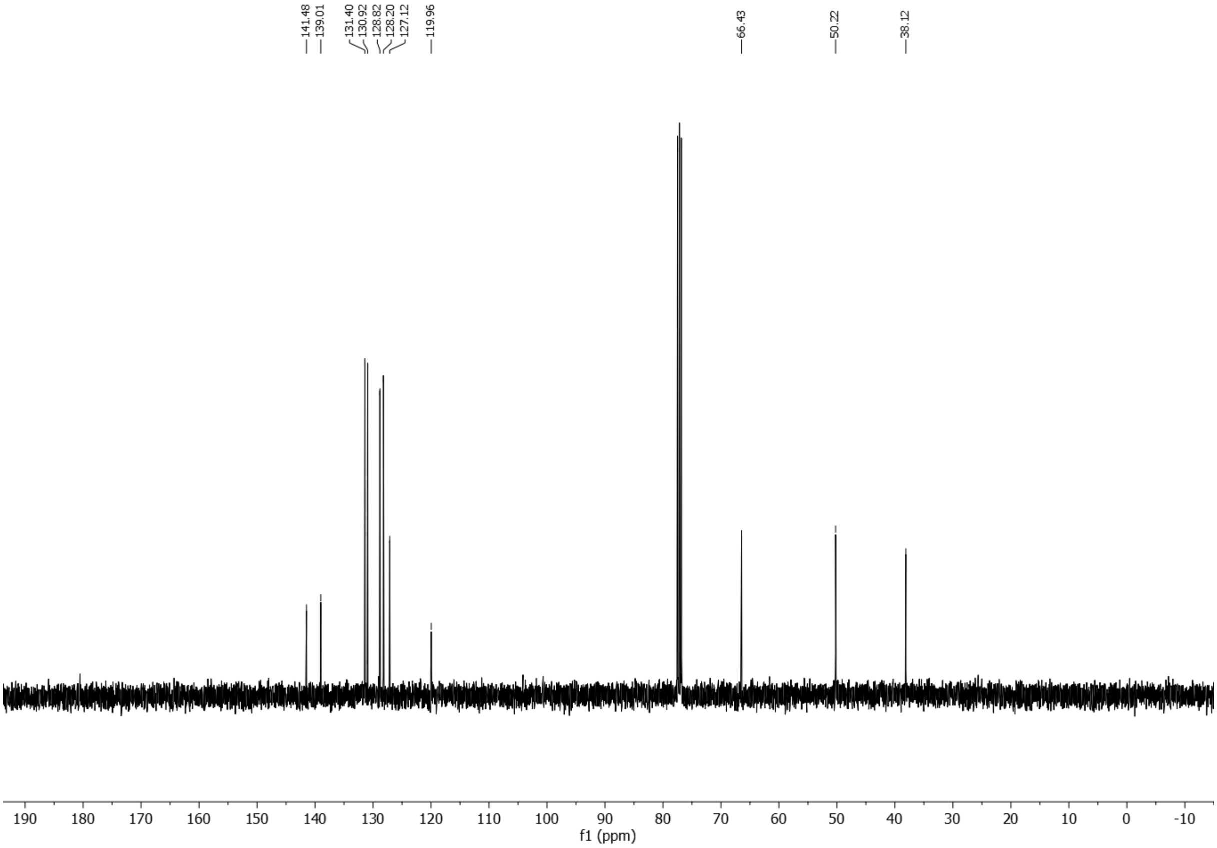

^{1}H NMR (400 MHz, $CDCl_3$), ^{13}C NMR (100 MHz, $CDCl_3$) and ^{19}F NMR (376 MHz, $CDCl_3$); 3-(4-fluorophenyl)-2-phenylpropan-1-ol (12h)

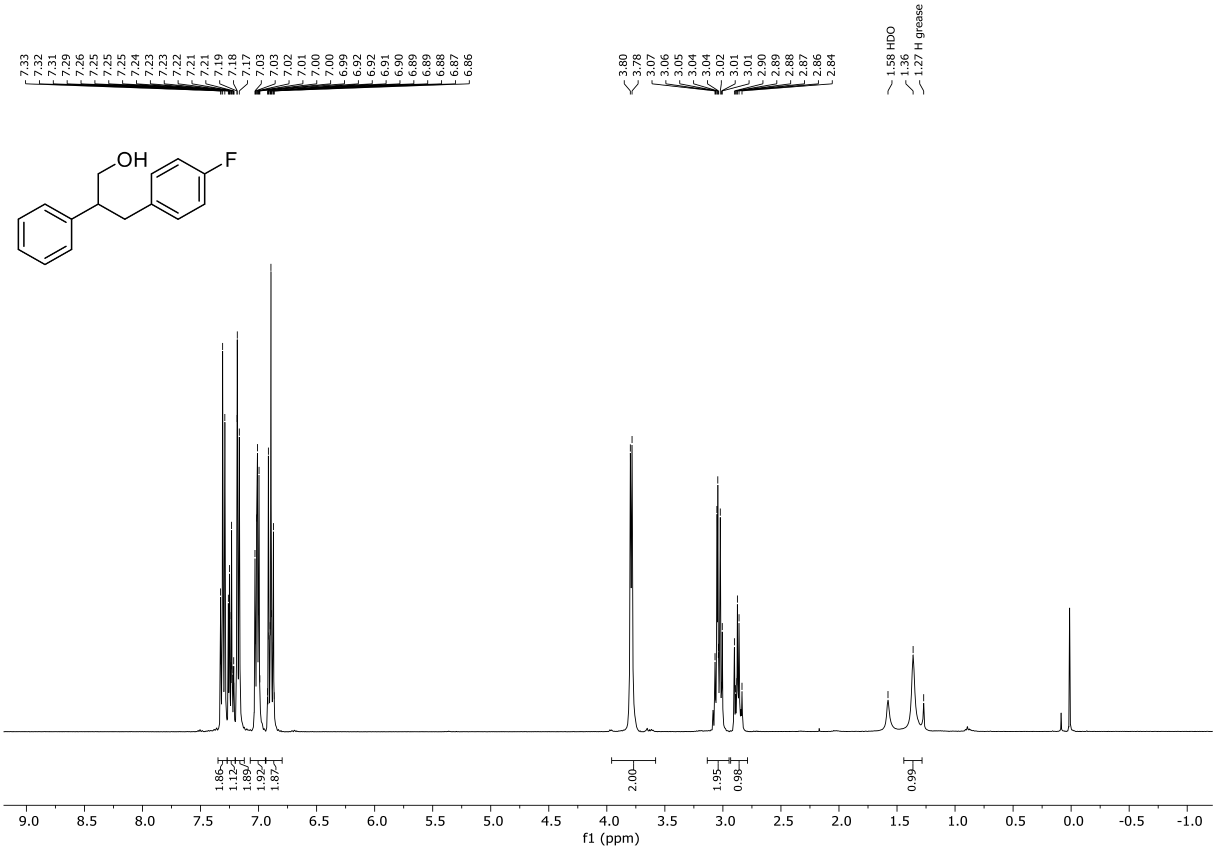

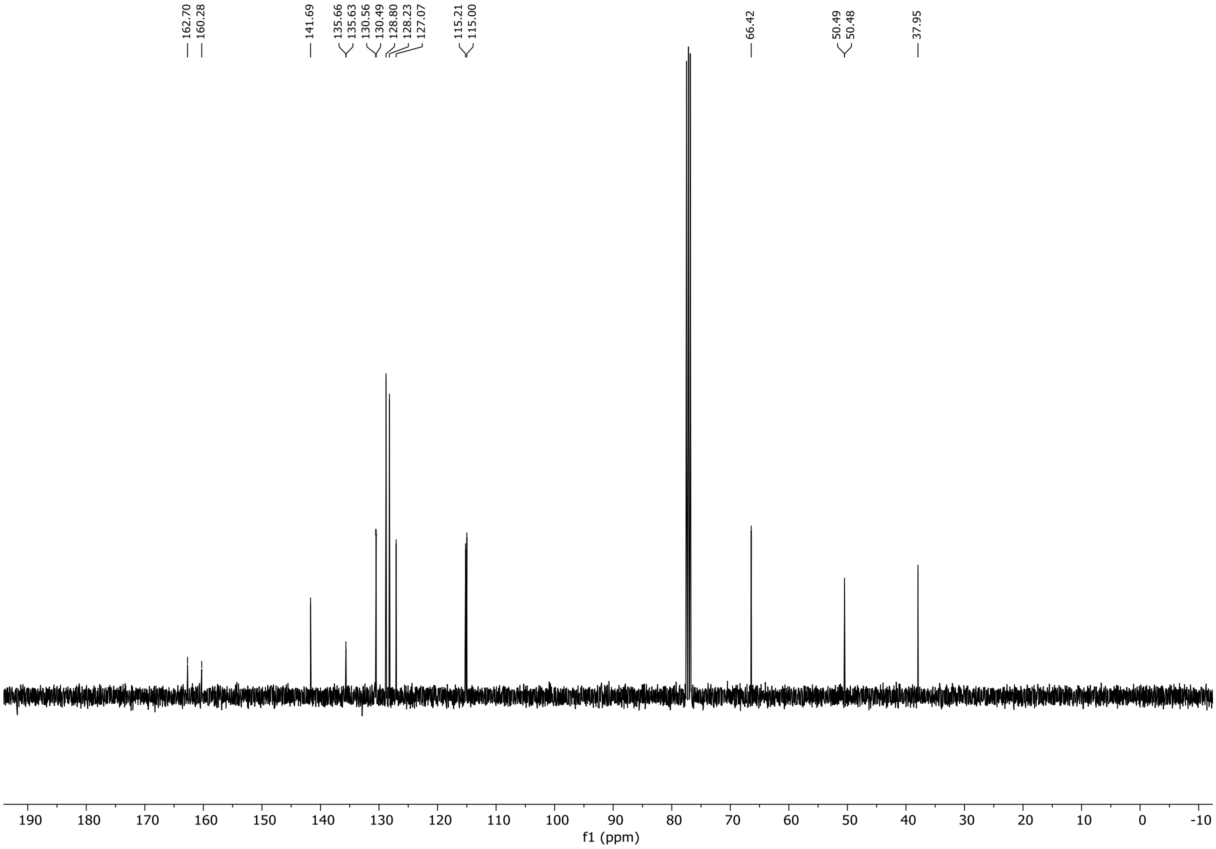

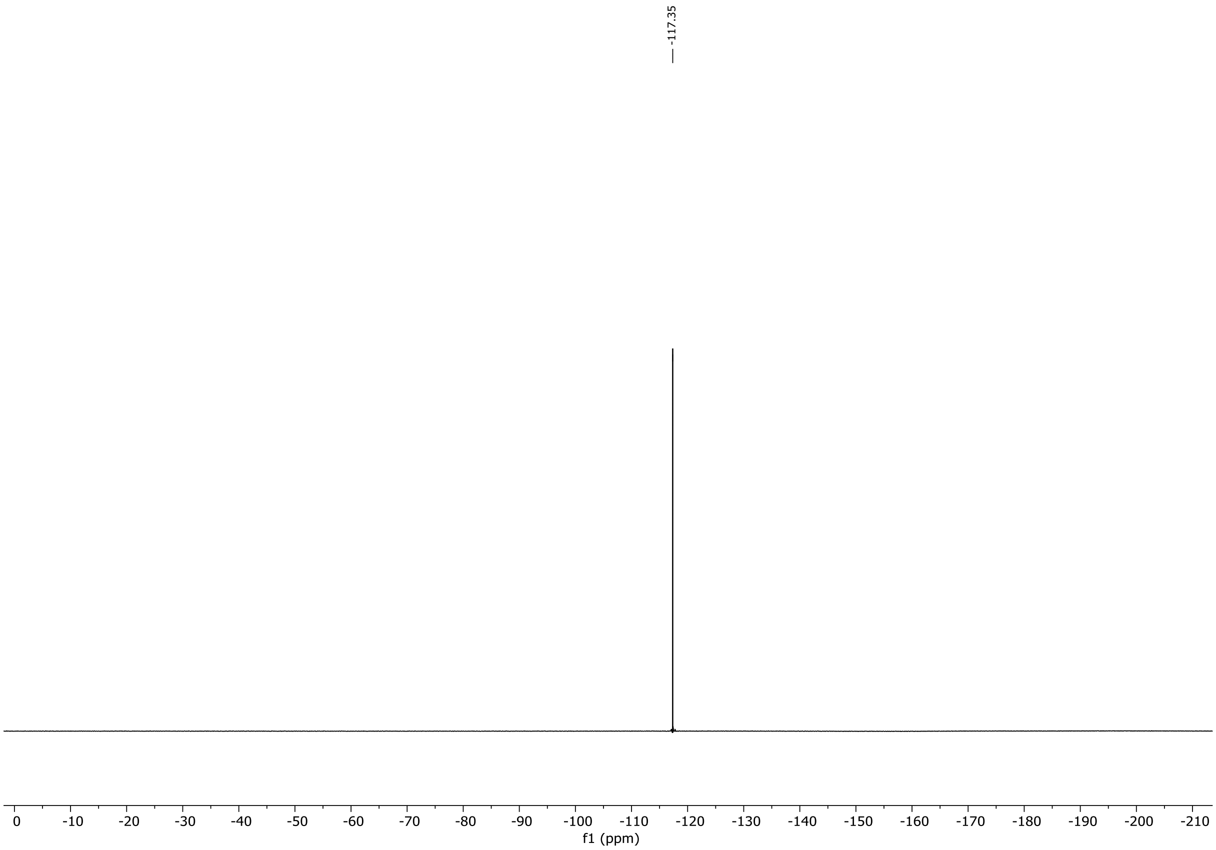
-117.35
0
-10
-20
-30
-40
-50
-60
-70
-80
-90
-100
-110
-120
-130
-140
-150
-160
-170
-180
-190
-200
-210
f1 (ppm)

^{1}H NMR (400 MHz, $CDCl_3$) and ^{13}C NMR (100 MHz, $CDCl_3$); 3-(4-(hydroxymethyl)phenyl)-2-phenylpropan-1-ol (13)

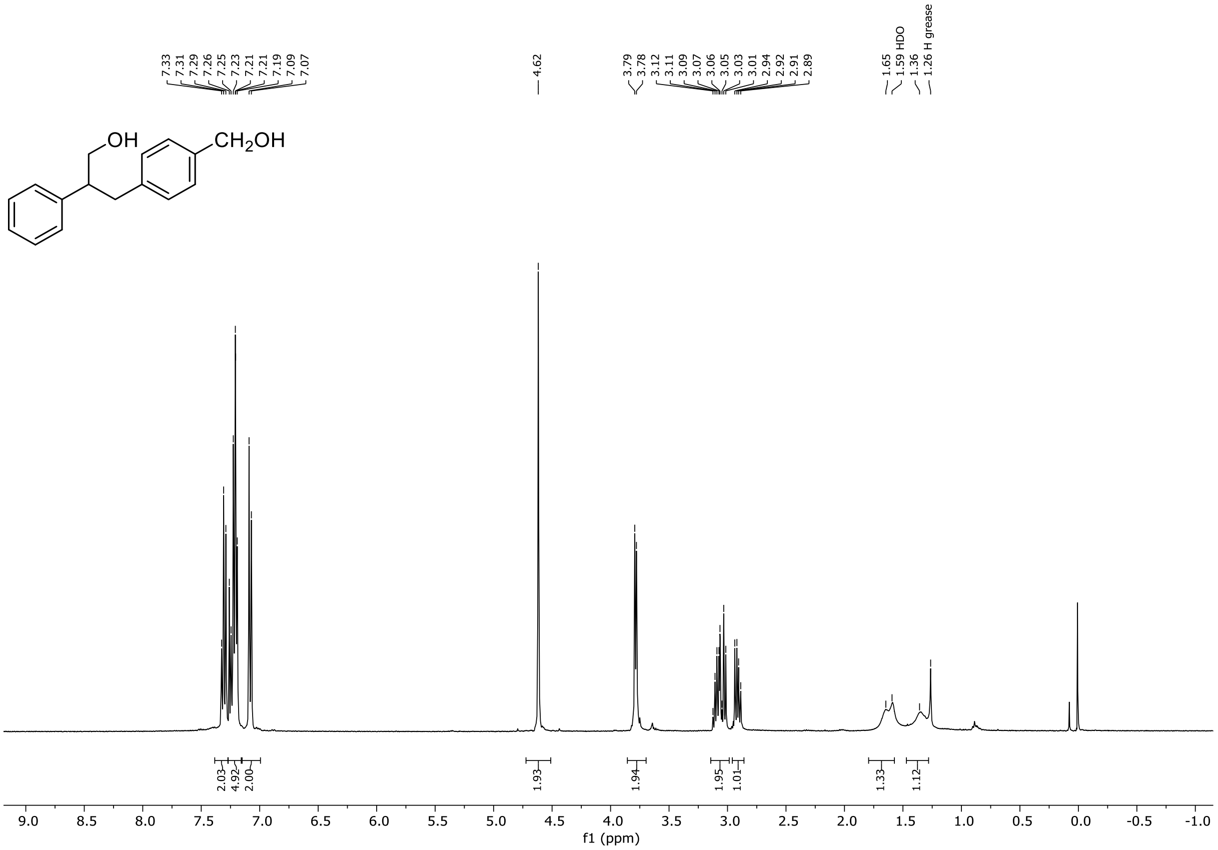

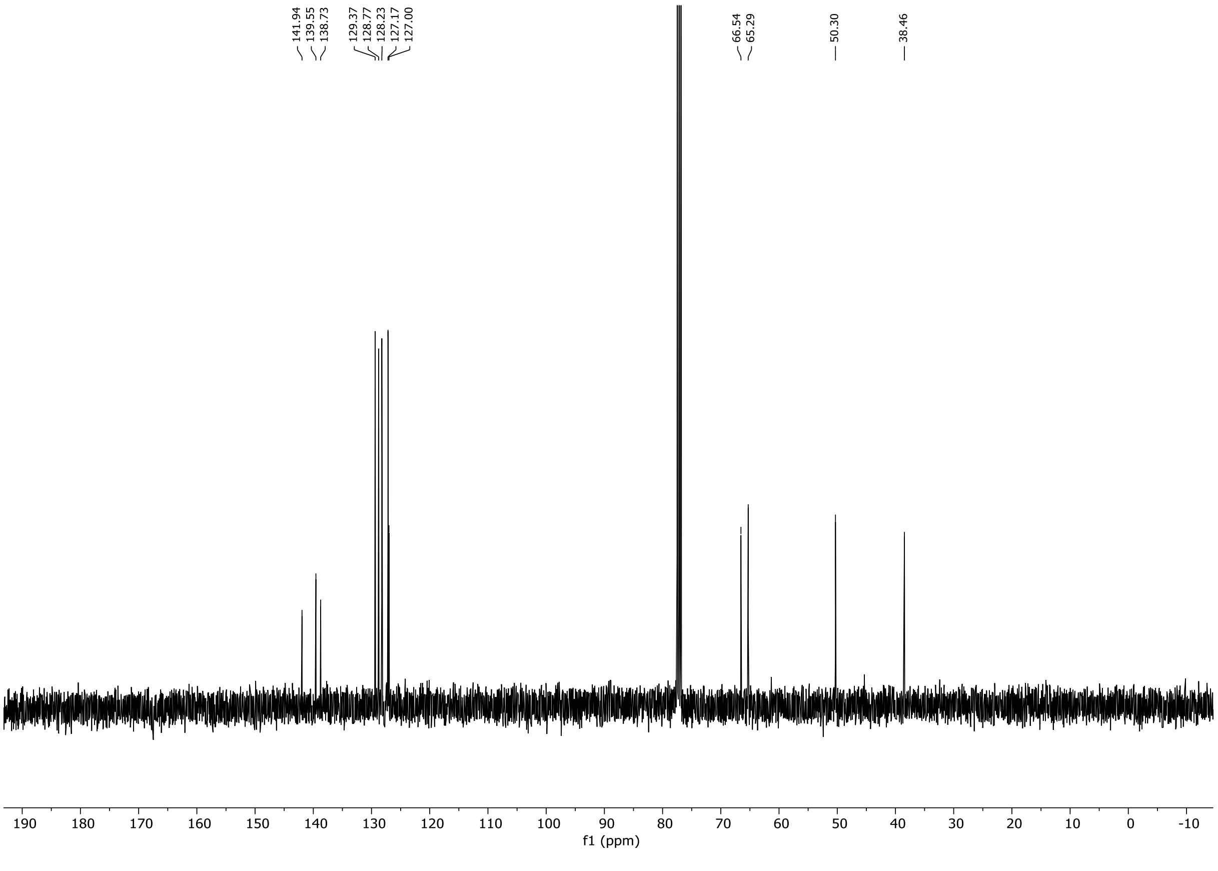

^{1}H NMR (400 MHz, $CDCl_3$) and ^{13}C NMR (100 MHz, $CDCl_3$); 2-phenyl-3-(1-tosyl-1*H*-indol-5-yl)propan-1-ol (14)

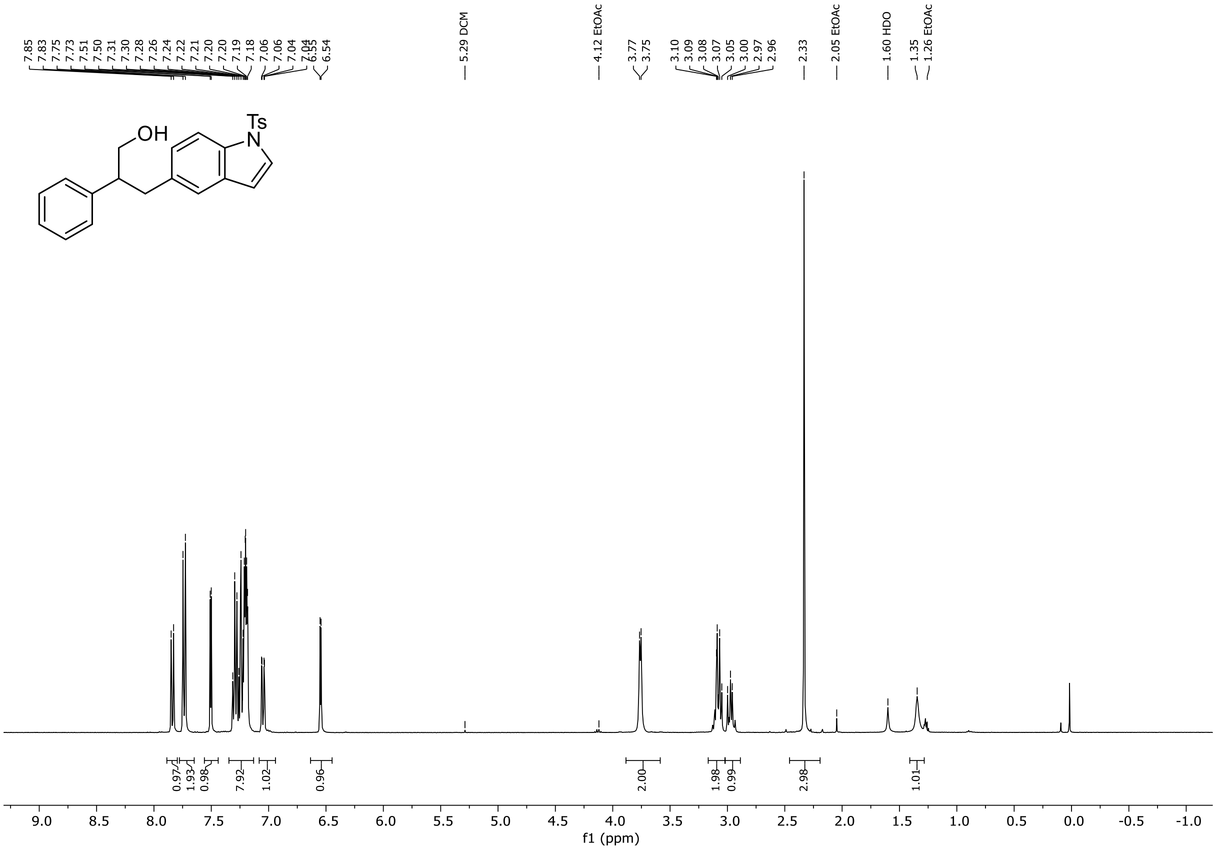

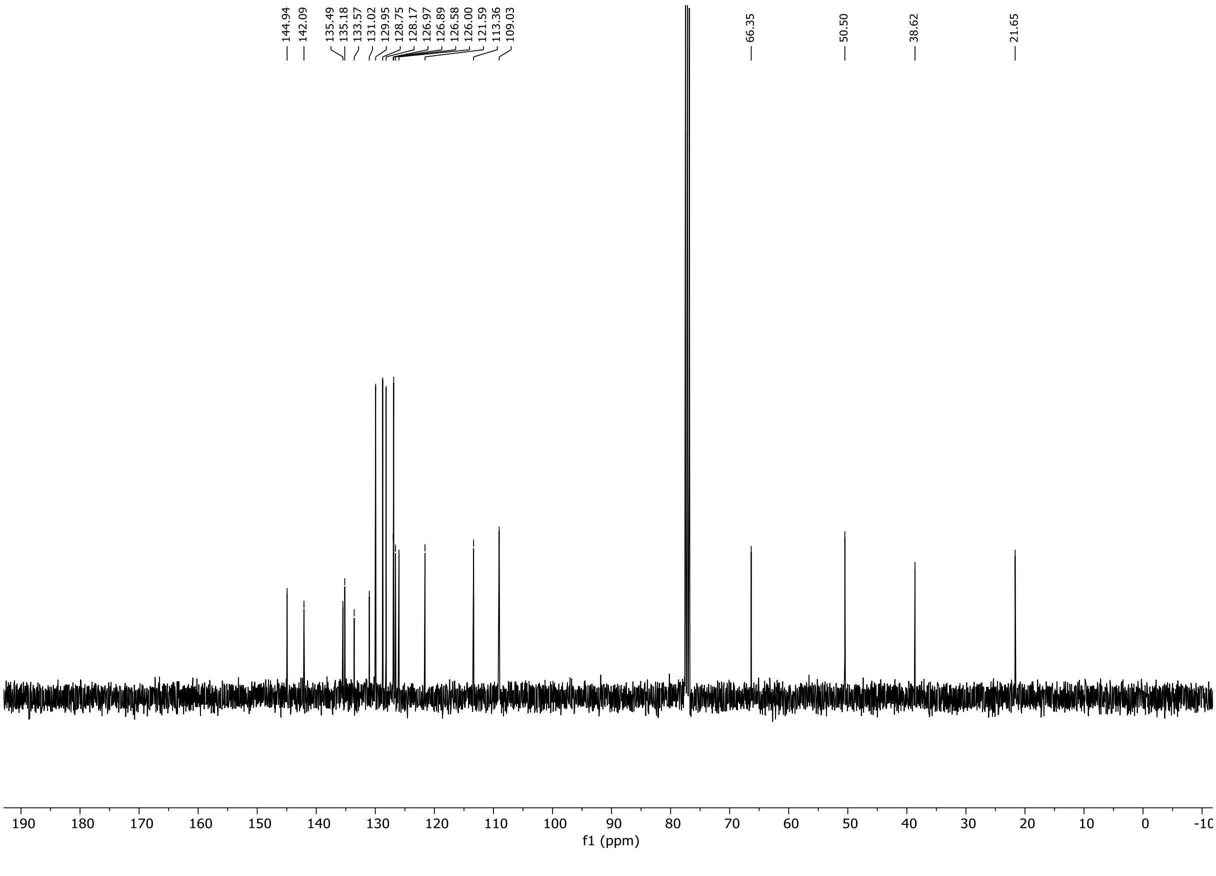

^{1}H NMR (400 MHz, $CDCl_3$) and ^{13}C NMR (100 MHz, $CDCl_3$); 3-(naphthalen-1-yl)-2-phenylpropan-1-ol (15)

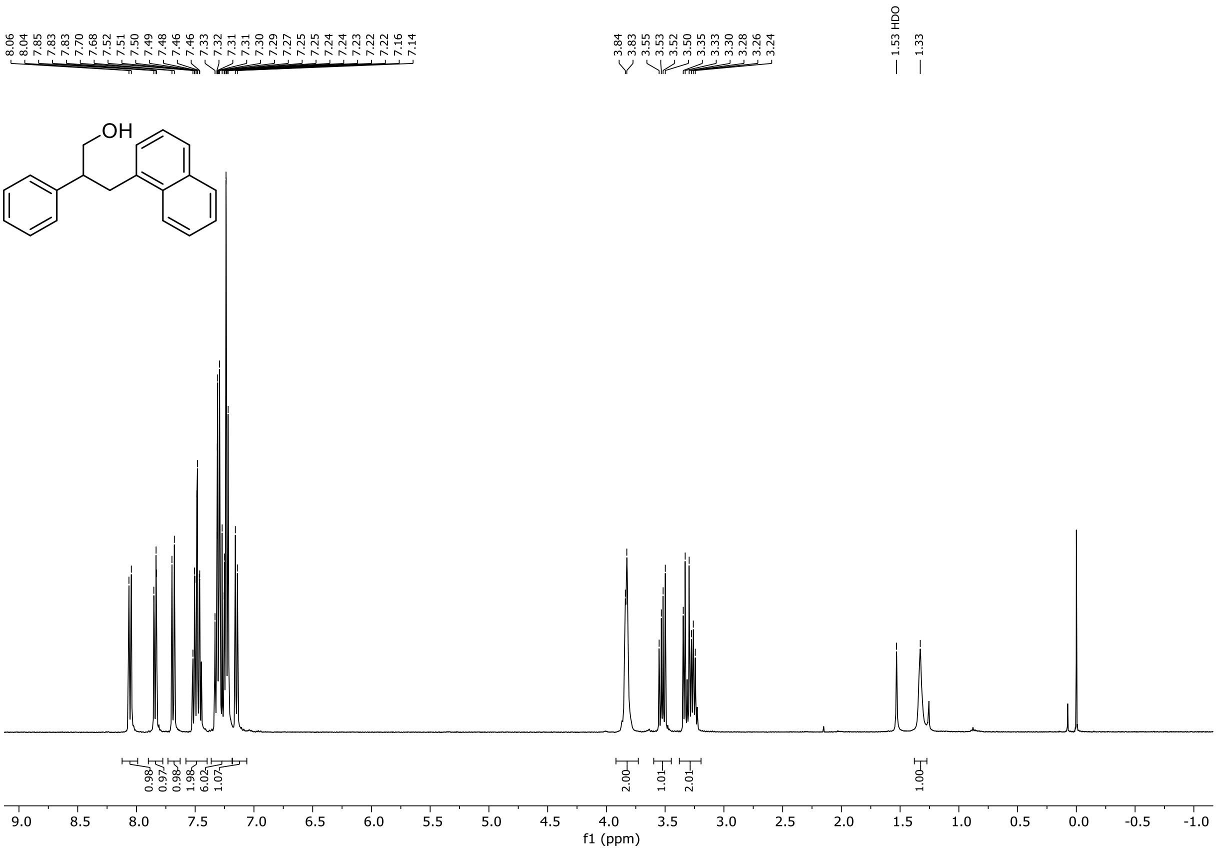

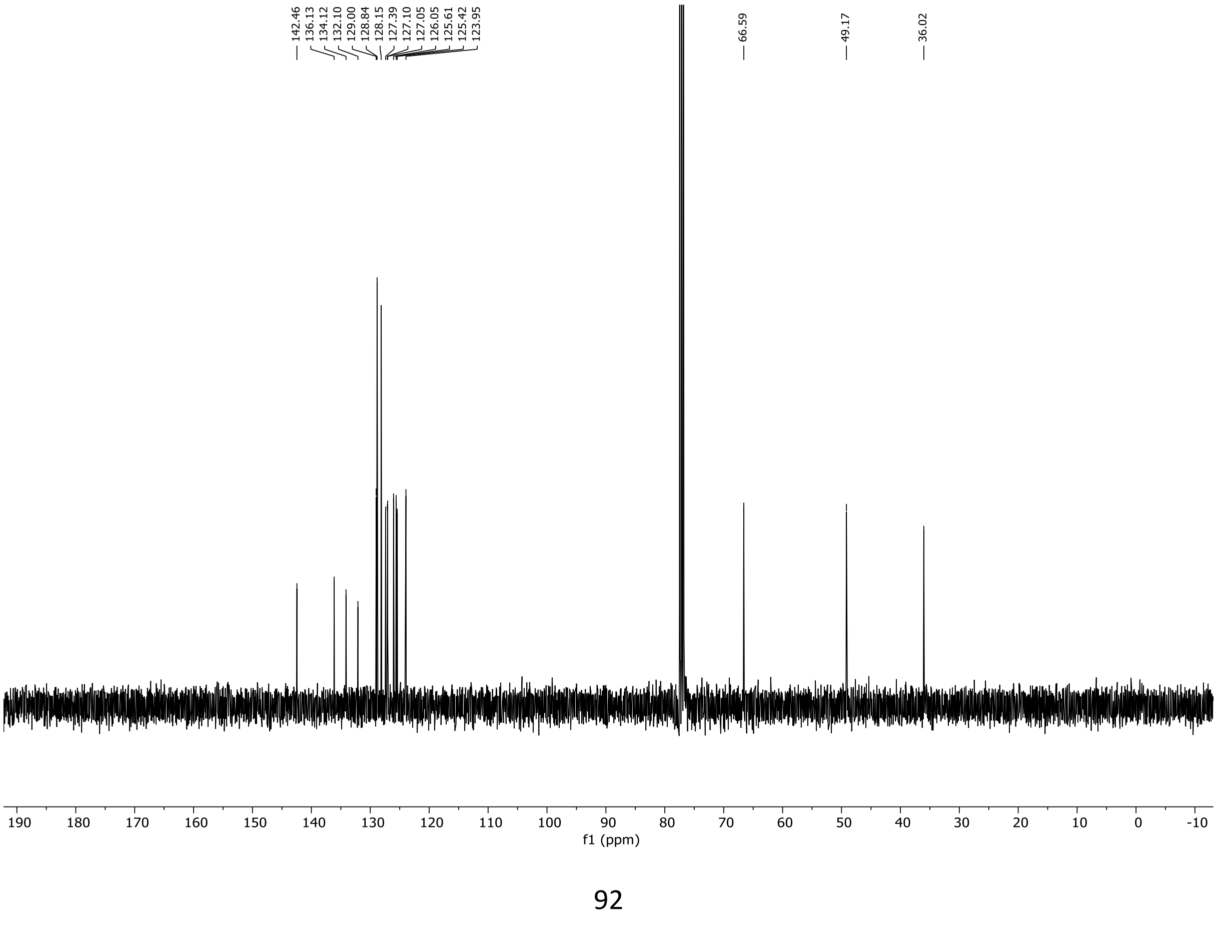

^{1}H NMR (400 MHz, $CDCl_3$) and ^{13}C NMR (100 MHz, $CDCl_3$); 2-phenyl-3-(4-((trimethylsilyl)ethynyl)phenyl)propan-1-ol (16)

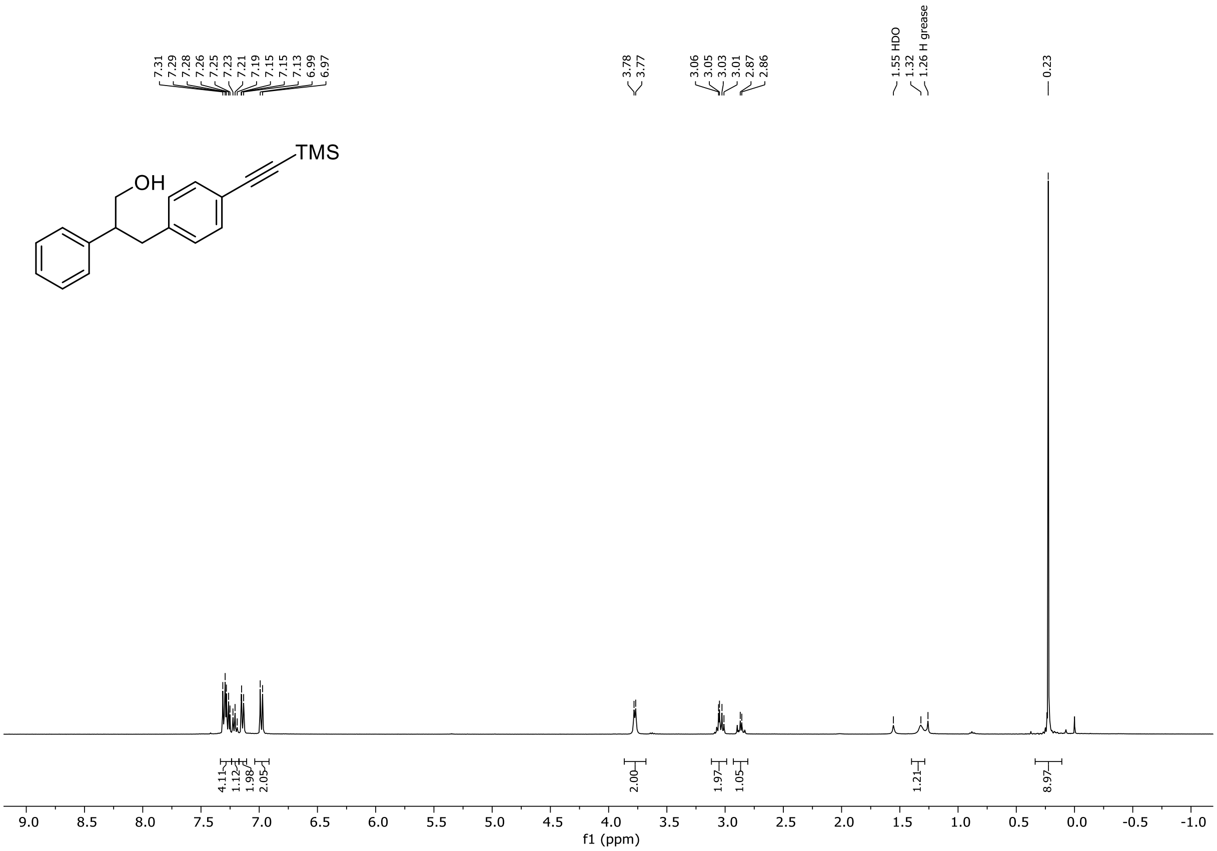

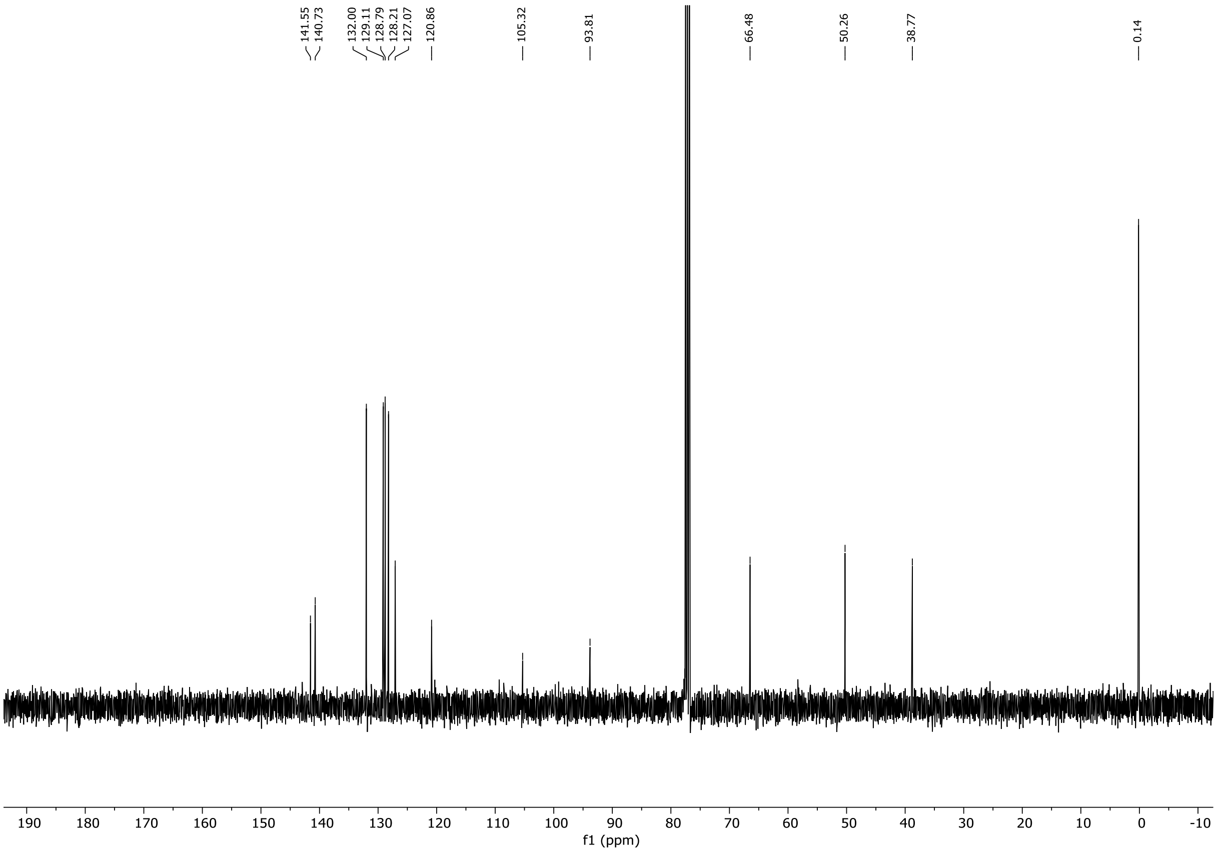

^{1}H NMR (400 MHz, $CDCl_3$) and ^{13}C NMR (100 MHz, $CDCl_3$); 2-phenyl-3-(2-methylphenyl)propan-1-ol (17)

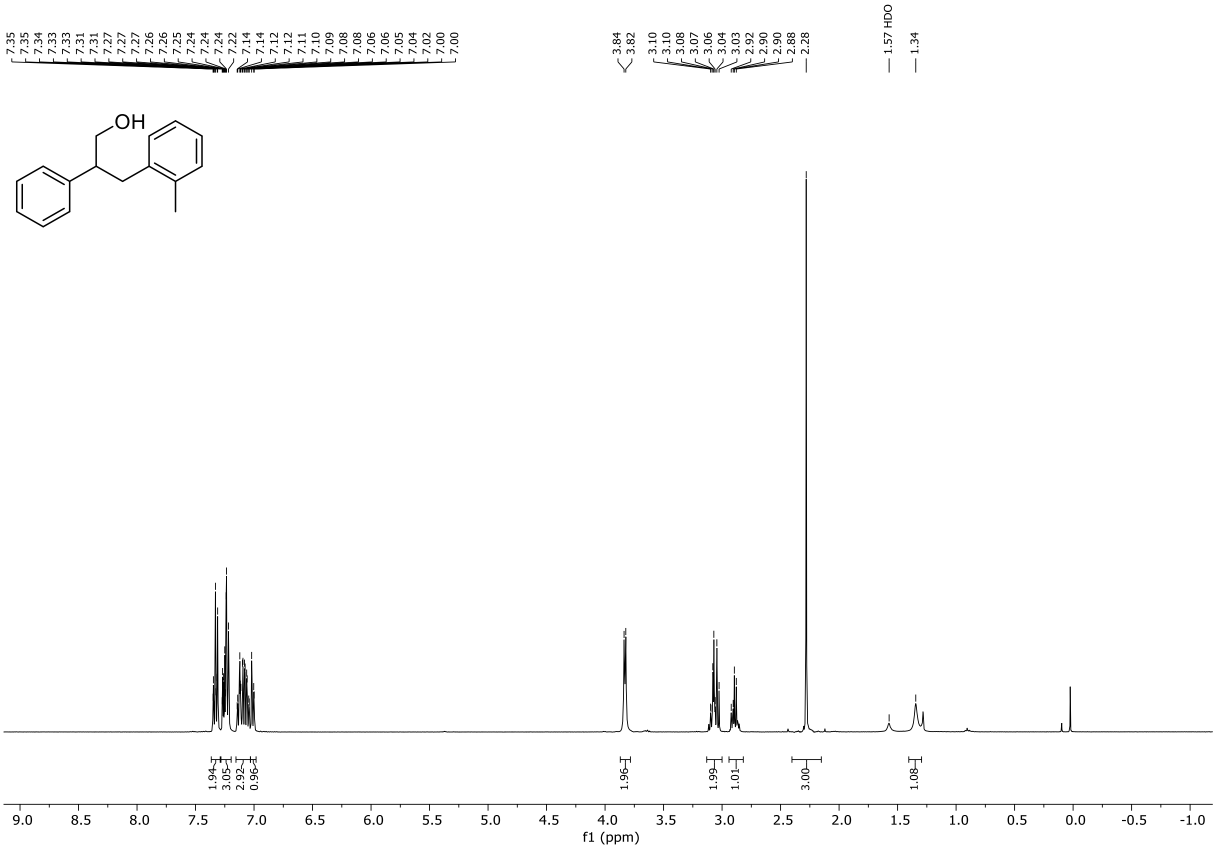

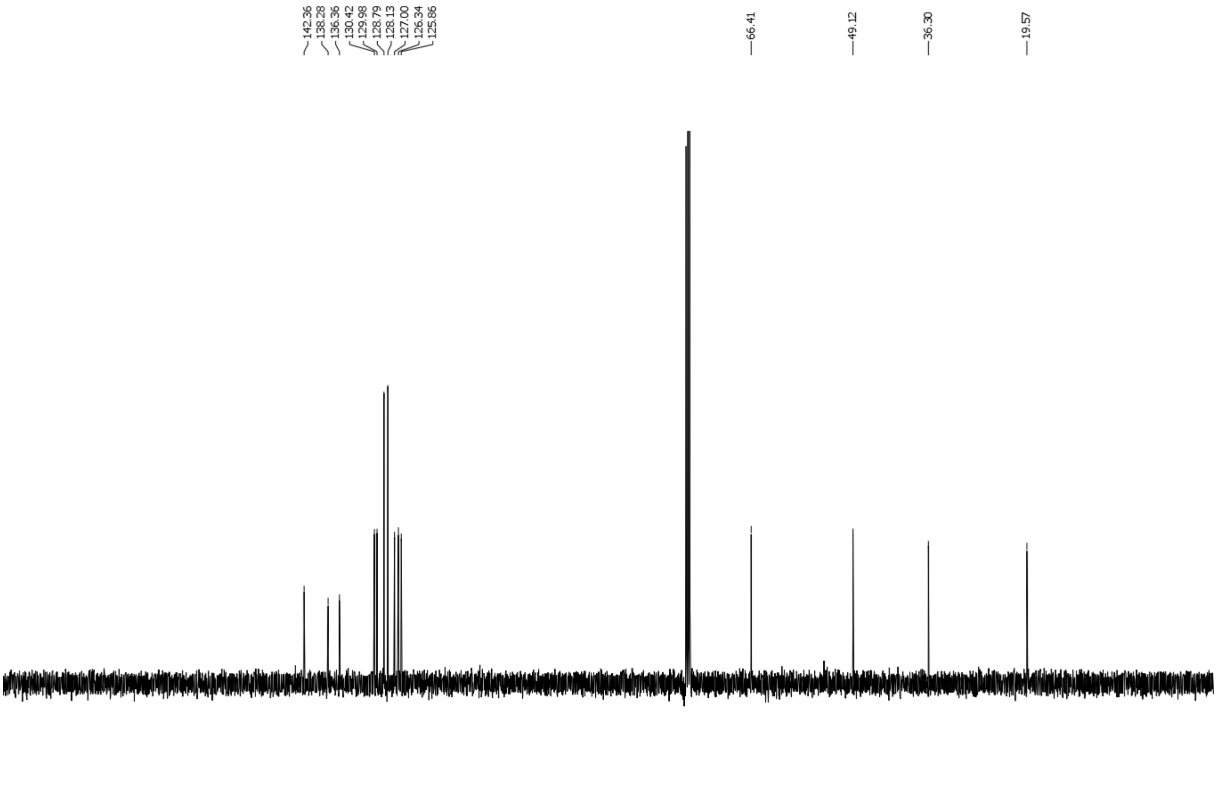

^{1}H NMR (400 MHz, CDCl$_3$) and ^{13}C NMR (100 MHz, CDCl$_3$); 2-phenyl-3-(3-methylphenyl)propan-1-ol (18)

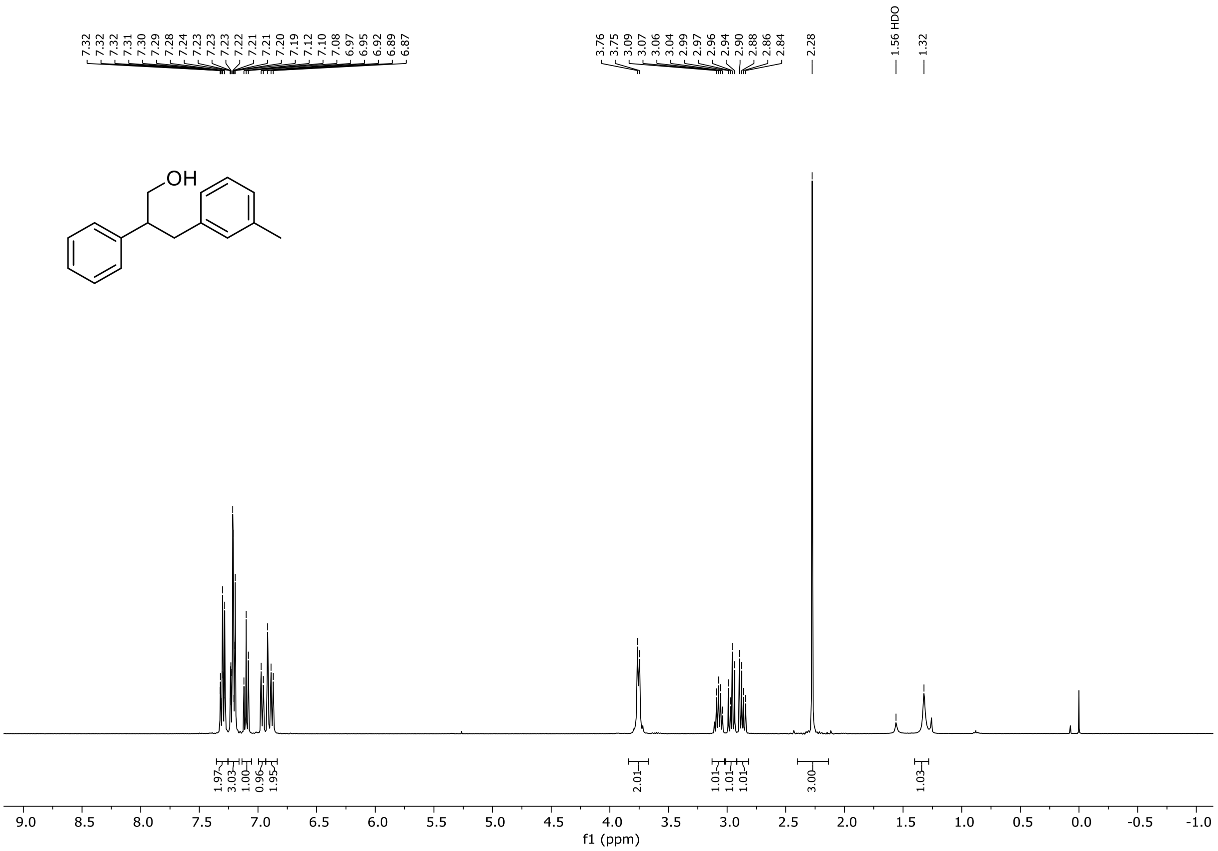

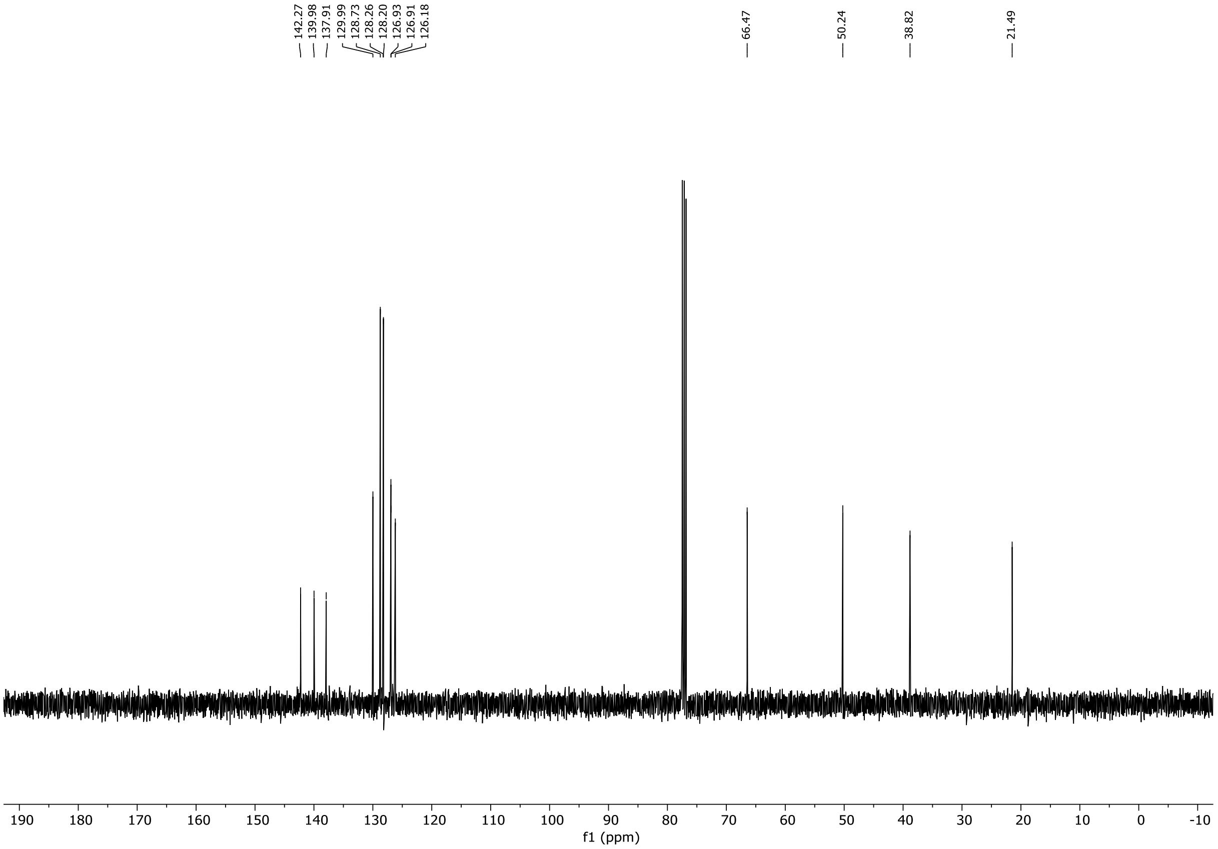

^{1}H NMR (400 MHz, CDCl$_3$) and ^{13}C NMR (100 MHz, CDCl$_3$); 3-(benzo[*d*][1,3]dioxol-5-yl)-2-phenylpropan-1-ol (19)

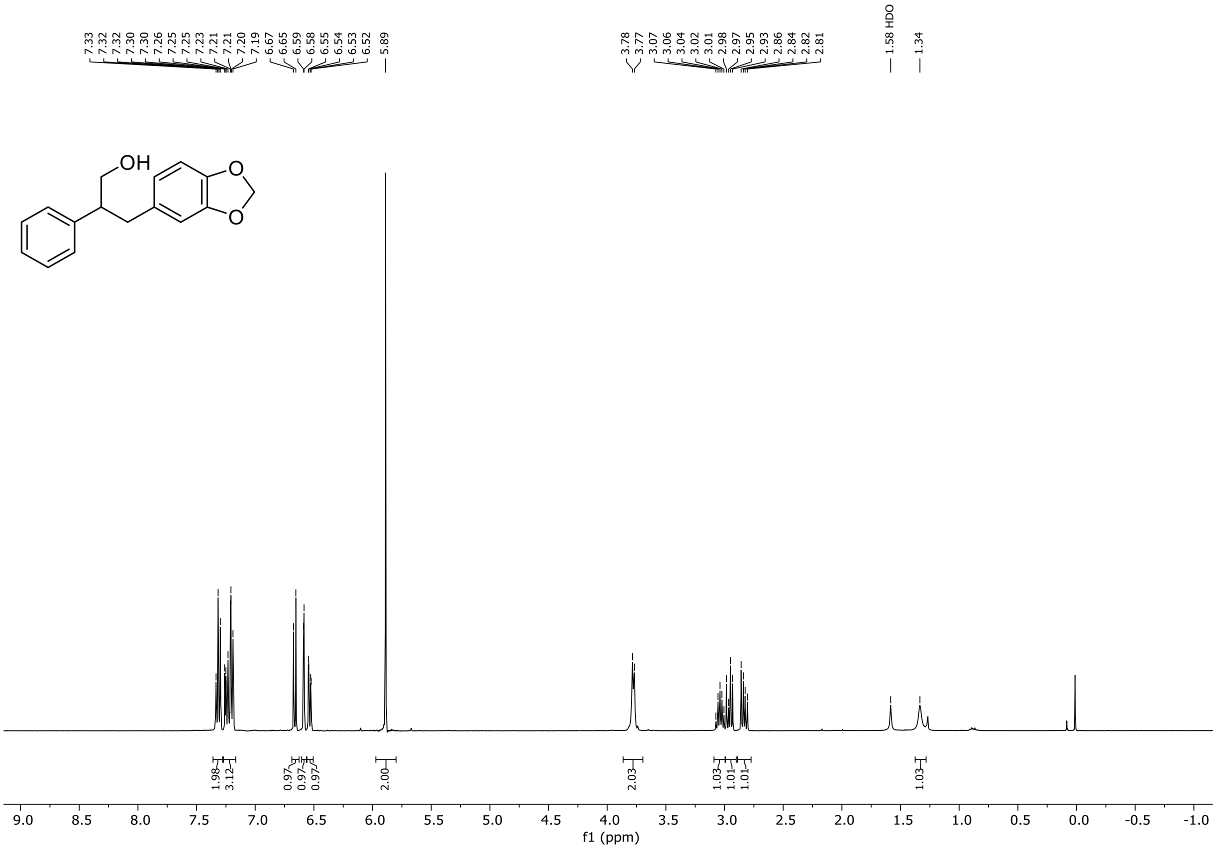

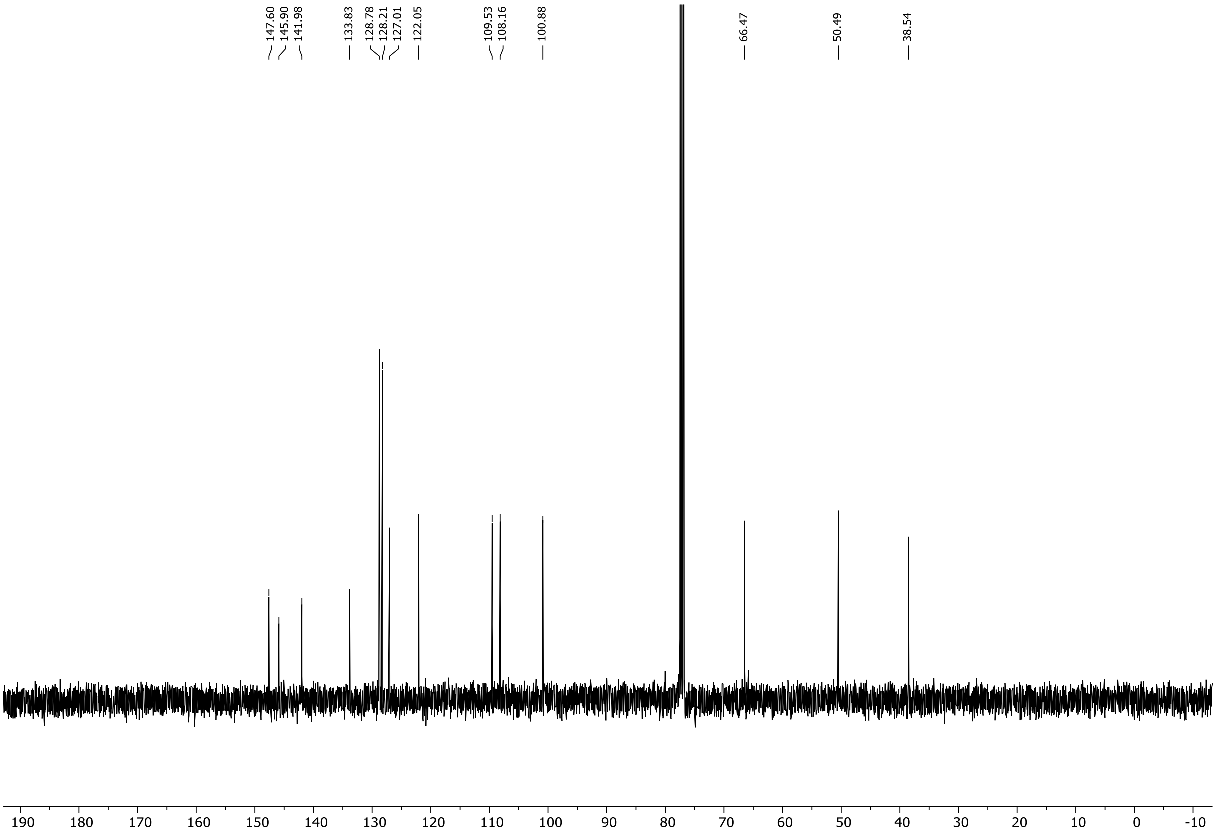

^{1}H NMR (500 MHz, $CDCl_3$) and ^{13}C NMR (125 MHz, $CDCl_3$)6-hydroxy-5-phenylhexanenitrile (22a)

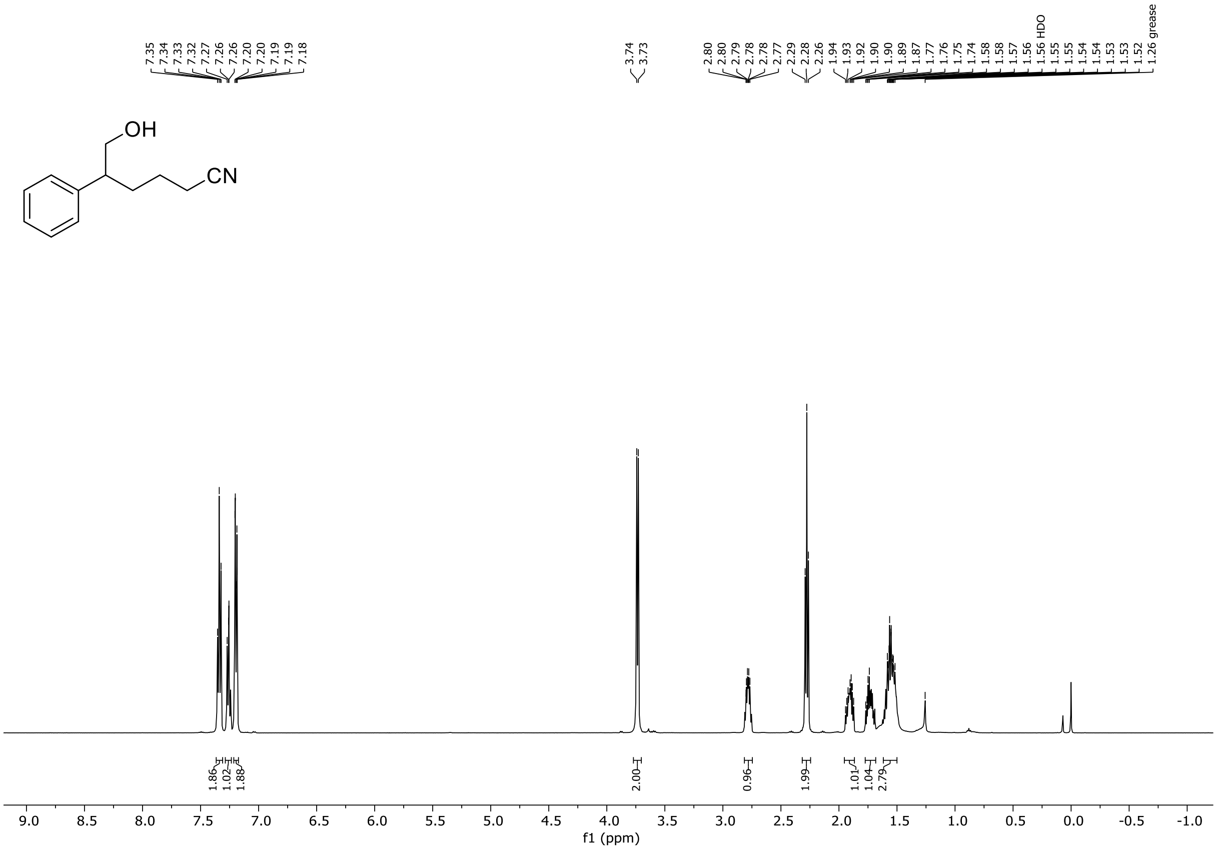

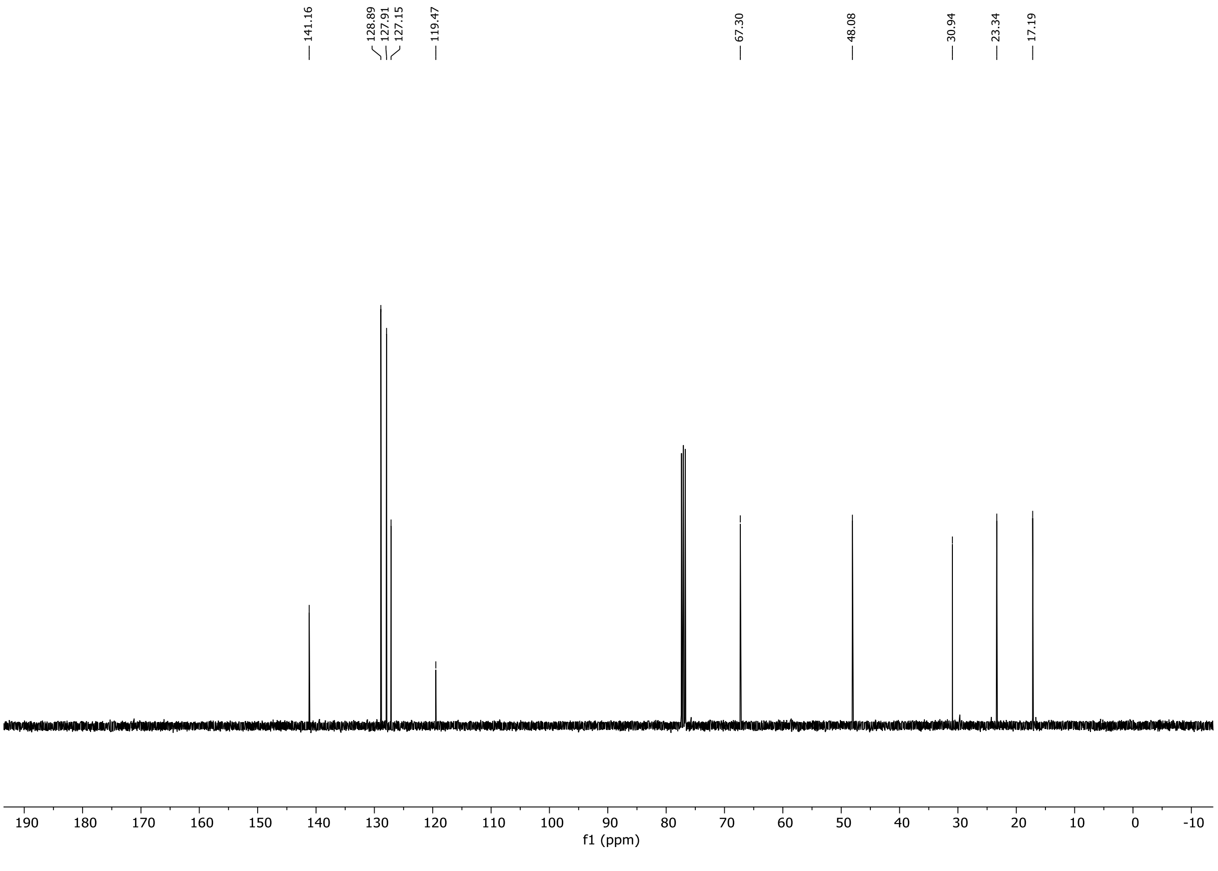

^{1}H NMR (400 MHz, CDCl$_3$) and ^{13}C NMR (100 MHz, CDCl$_3$); 6-hydroxy-5-(4-methoxyphenyl)hexanenitrile (22b)

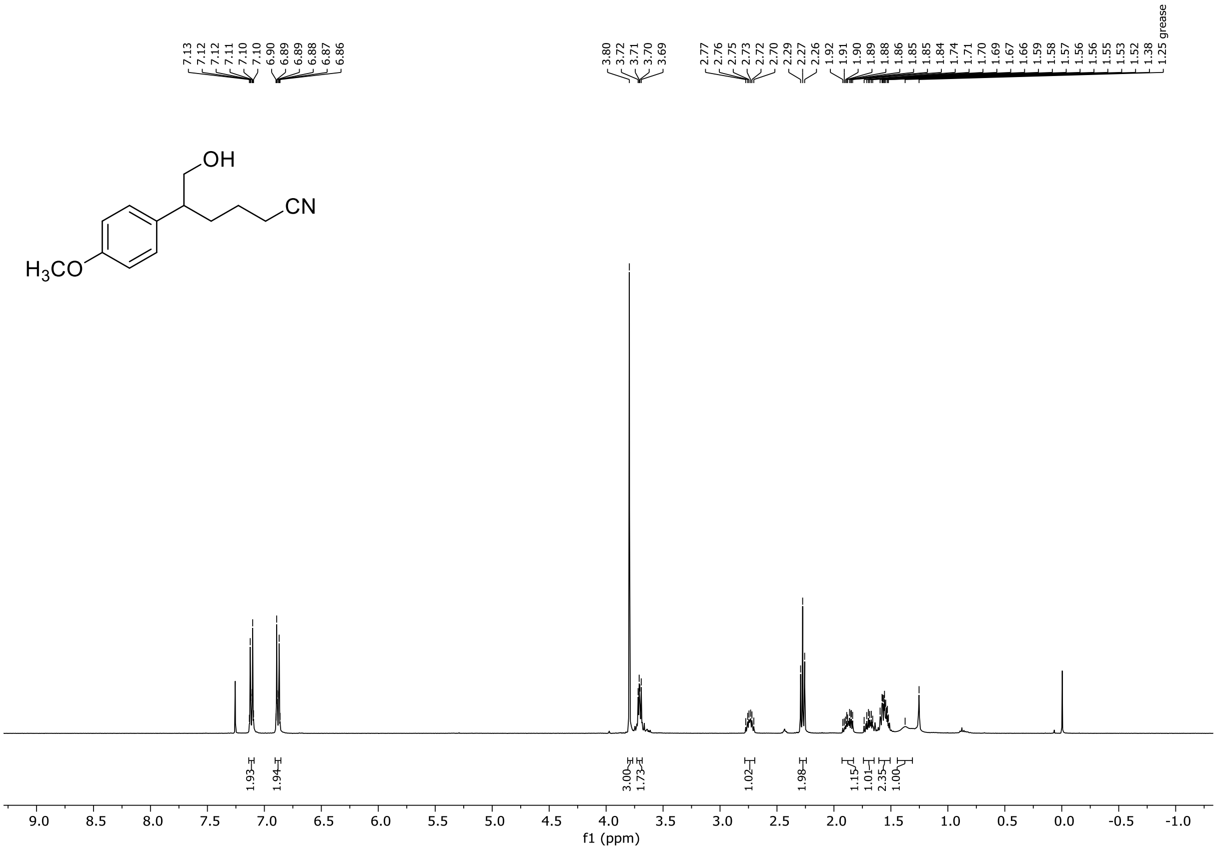

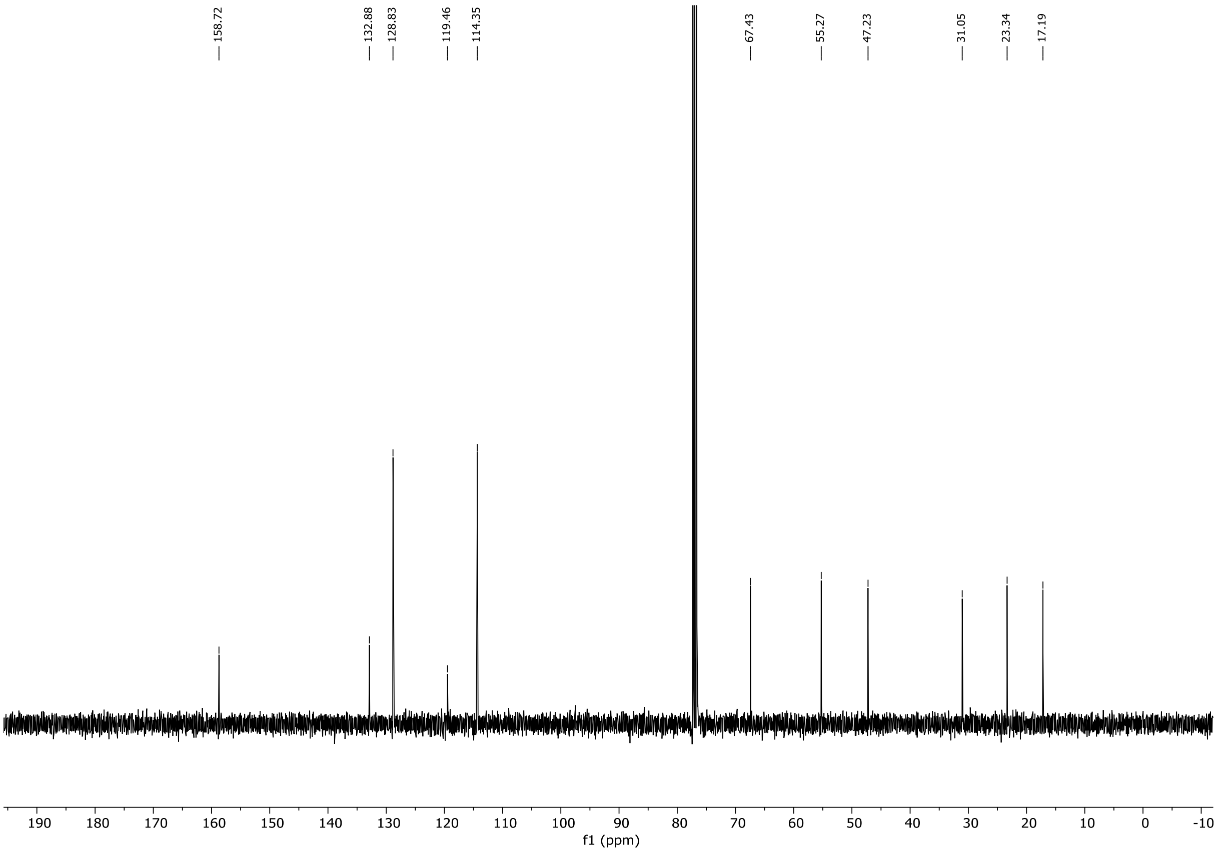

^{1}H NMR (400 MHz, $CDCl_3$) and ^{13}C NMR (100 MHz, $CDCl_3$); 4-(5-cyano-1-hydroxypentan-2-yl)benzonitrile (22c)

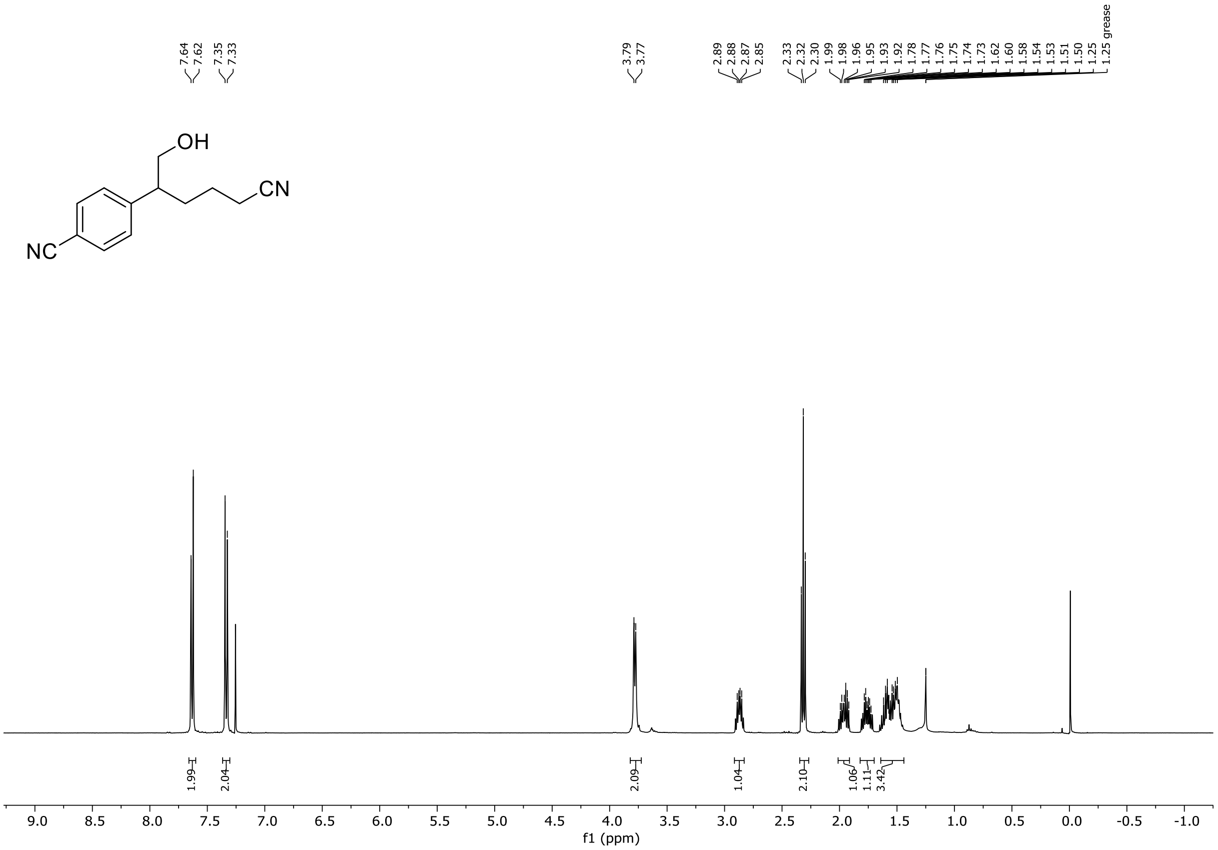

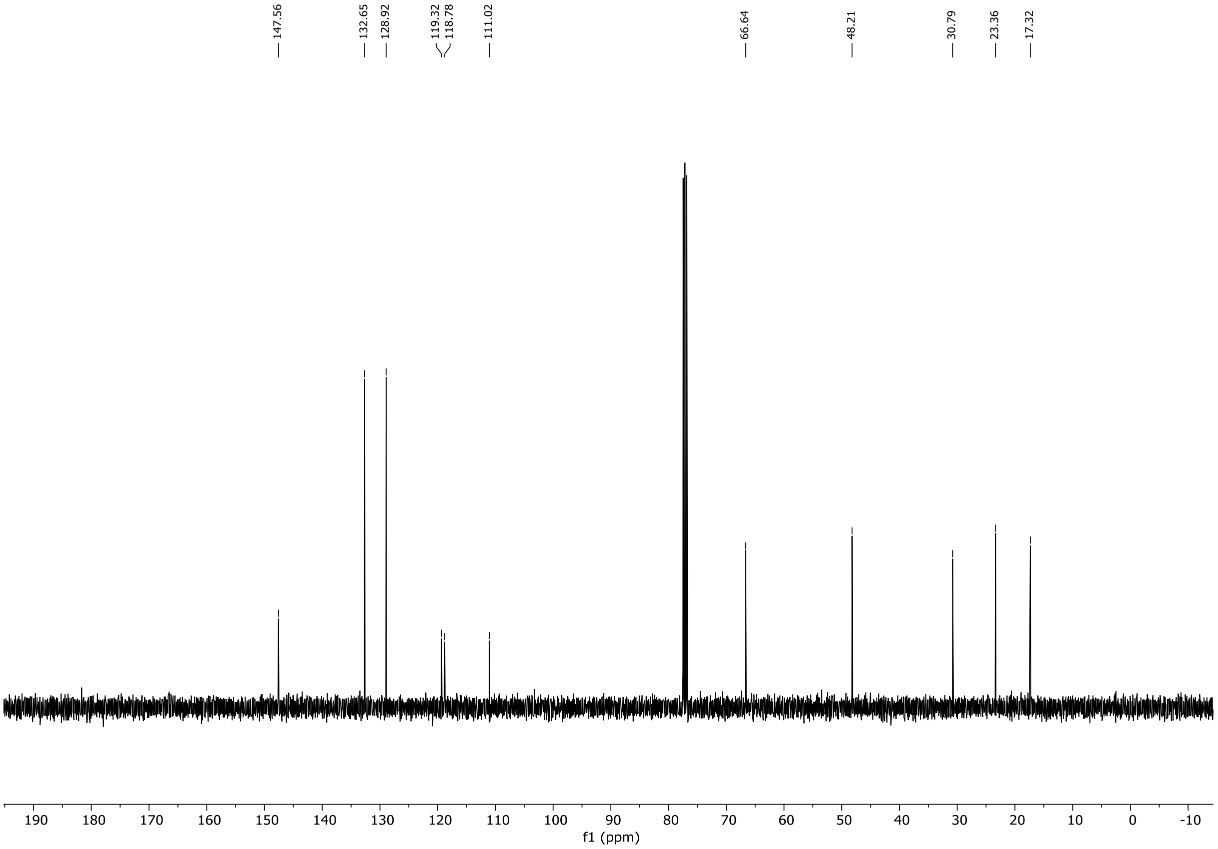

^{1}H NMR (400 MHz, CDCl$_3$) and ^{13}C NMR (100 MHz, CDCl$_3$); 6-hydroxy-5-(2-methylphenyl)hexanenitrile (22d)

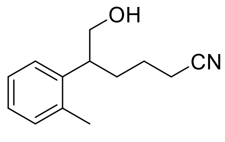

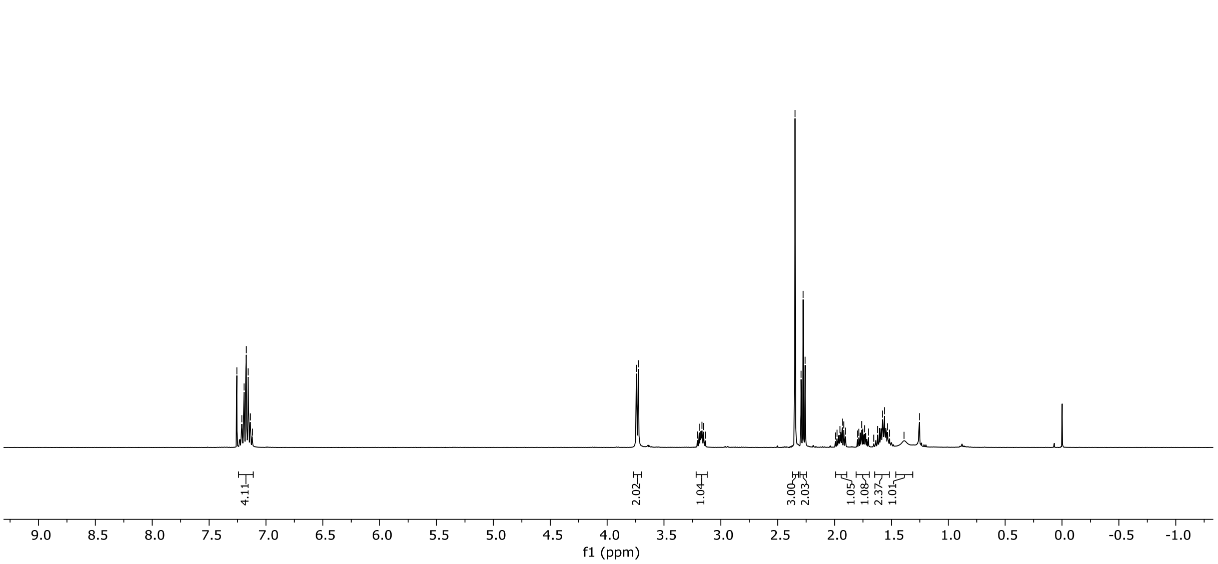

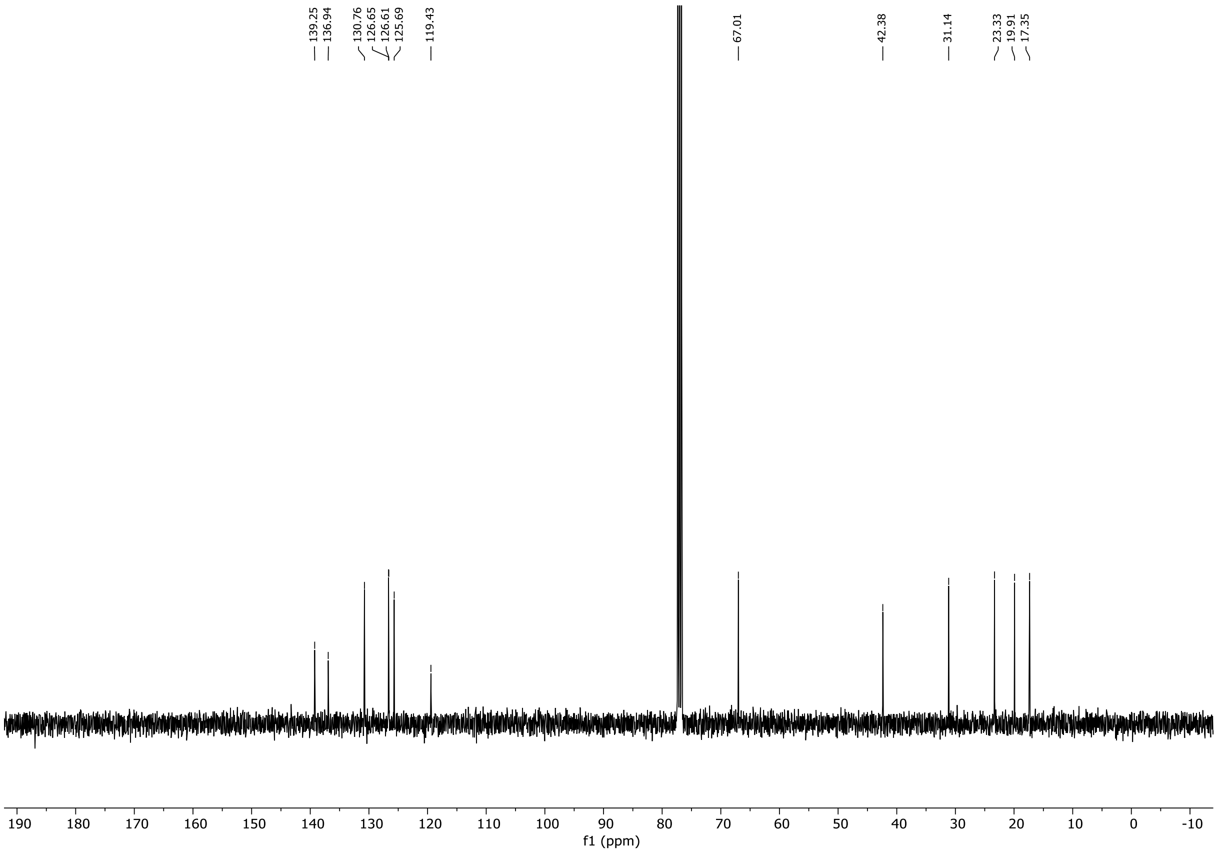

^{1}H NMR (400 MHz, CDCl$_3$) and ^{13}C NMR (100 MHz, CDCl$_3$); 6-hydroxy-5-(1*H*-indol-1-yl)hexanenitrile (23)

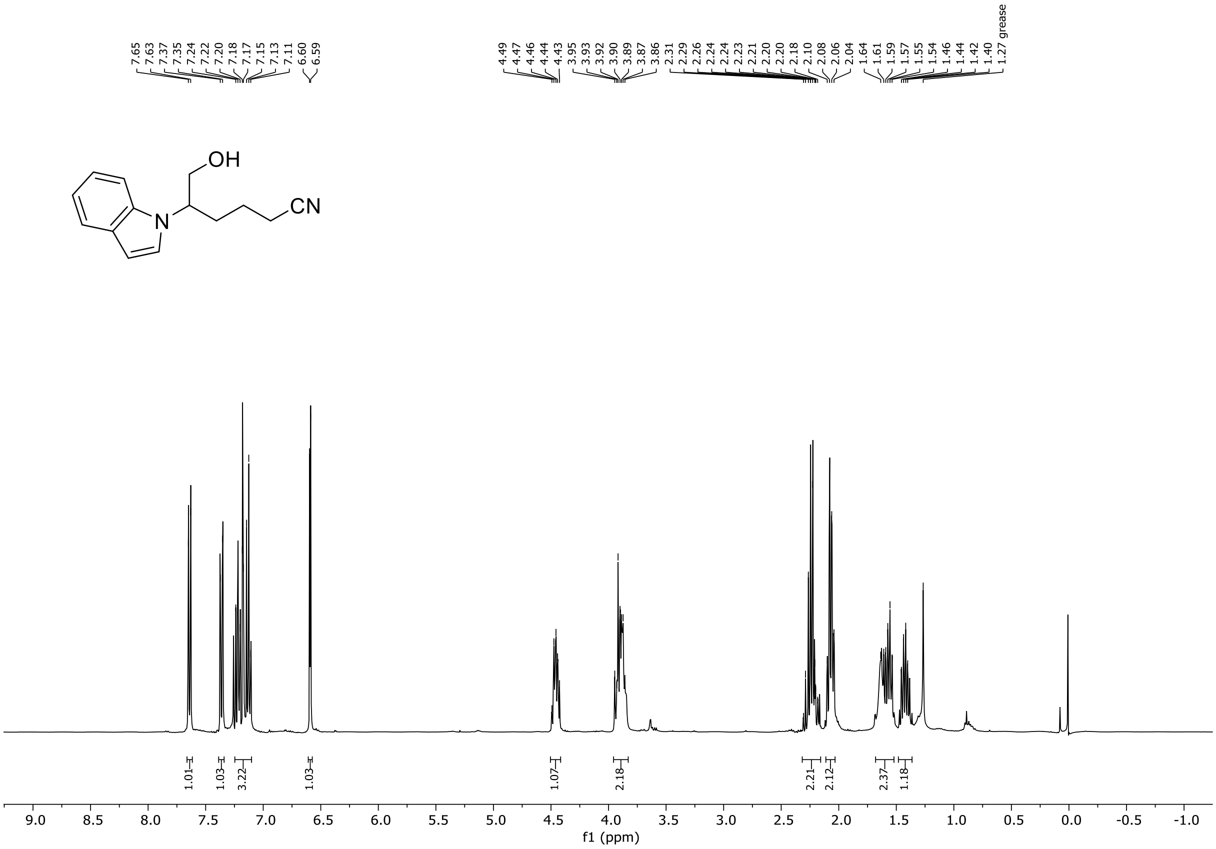

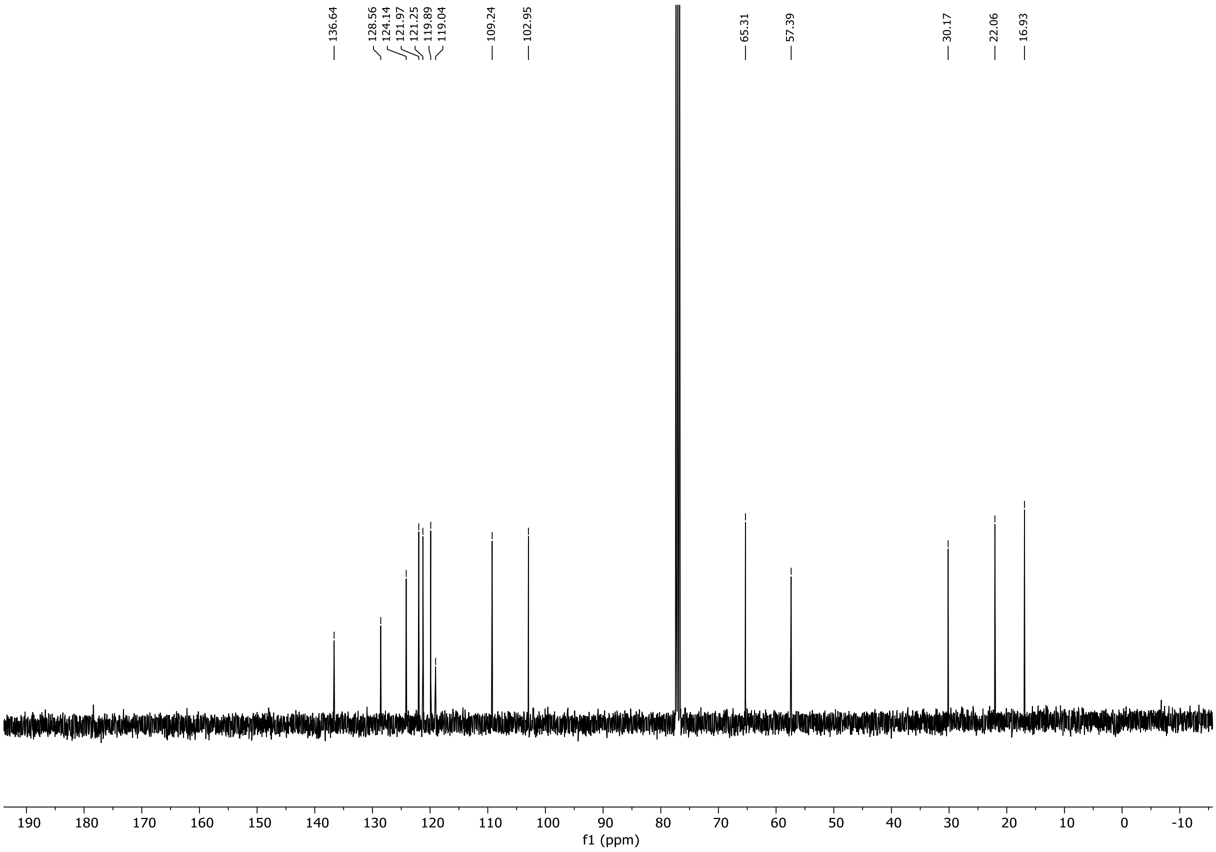

^{1}H NMR (500 MHz, $CDCl_3$) and ^{13}C NMR (125 MHz, $CDCl_3$); 5-(9*H*-carbazol-9-yl)-6-hydroxyhexanenitrile (24)

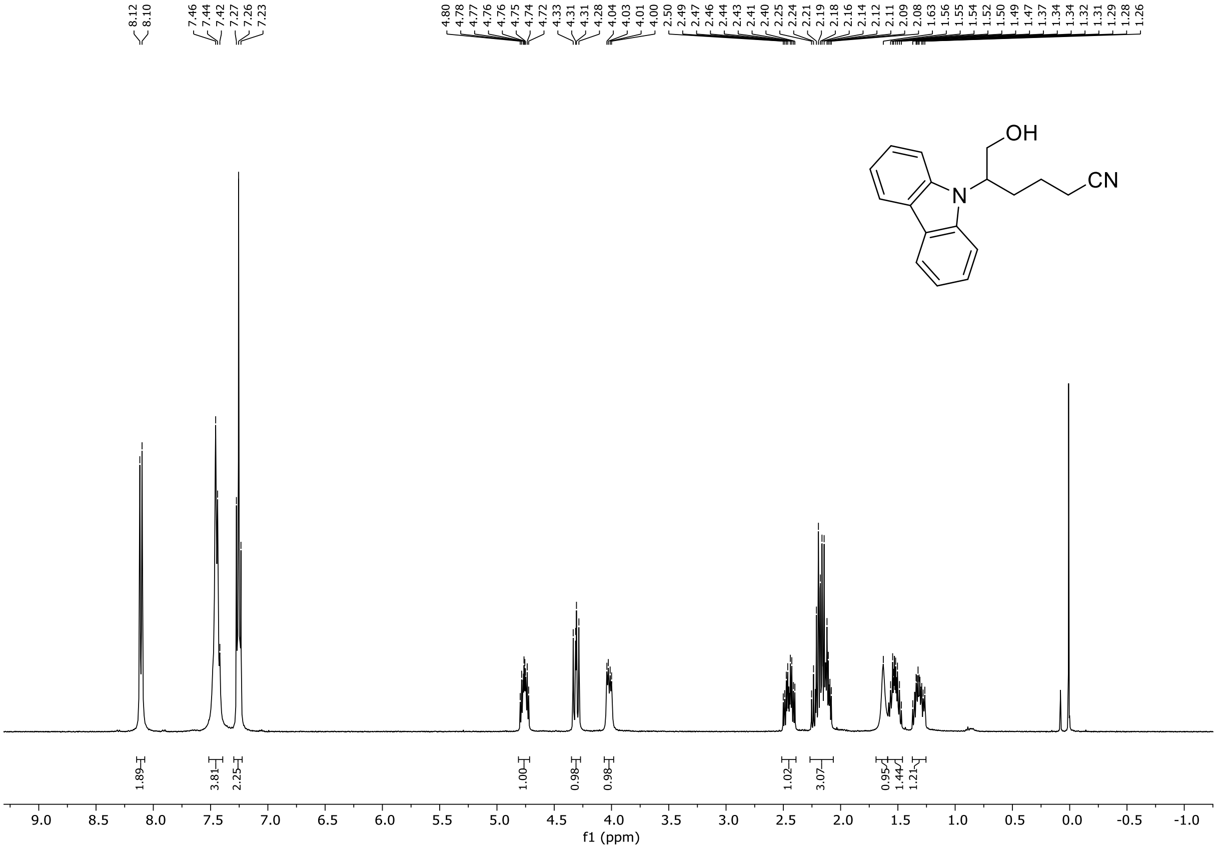

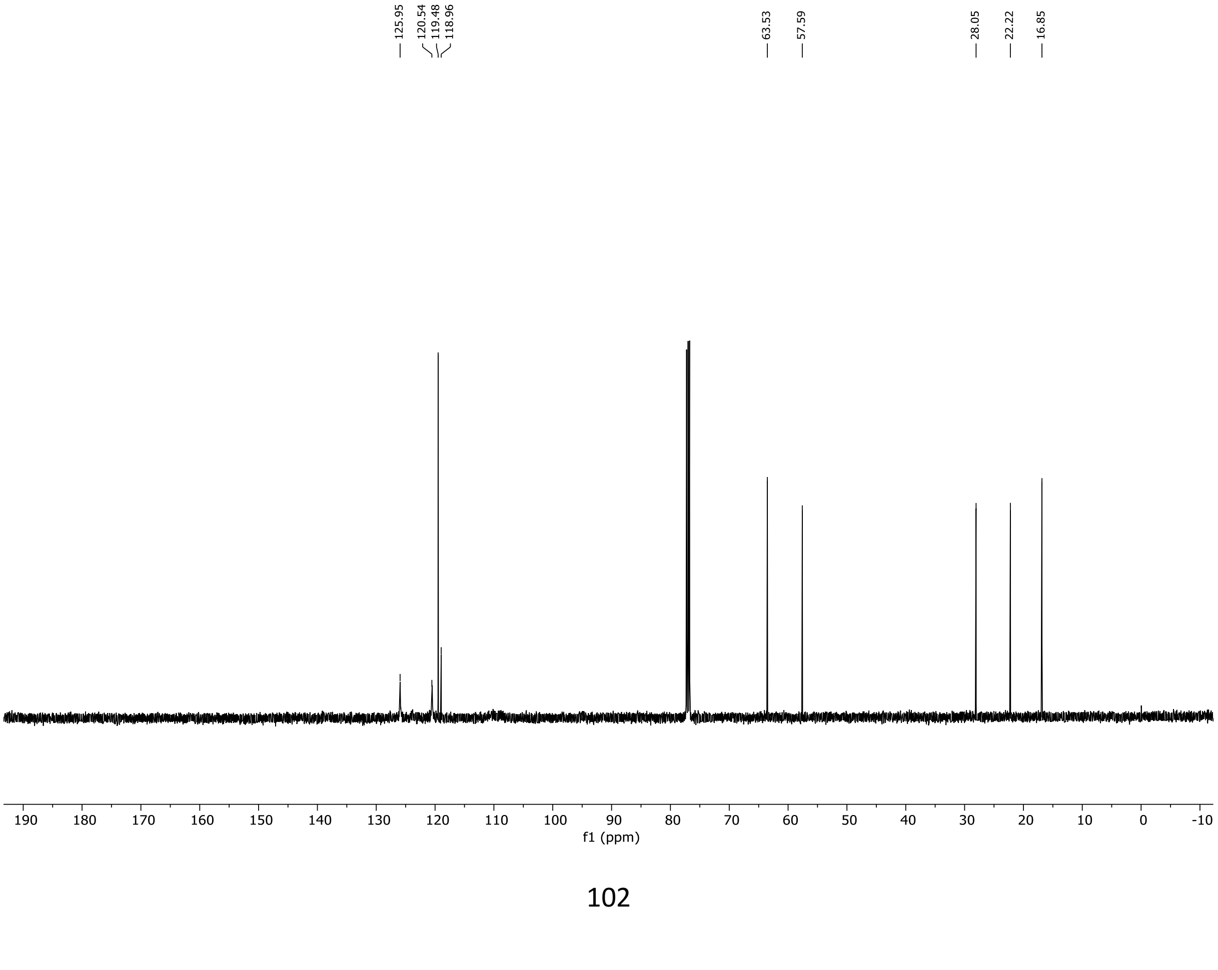

^{1}H NMR (500 MHz, $CDCl_3$) and ^{13}C NMR (125 MHz, $CDCl_3$); 6-hydroxy-5-(4-methoxyphenoxy)hexanenitrile (25)

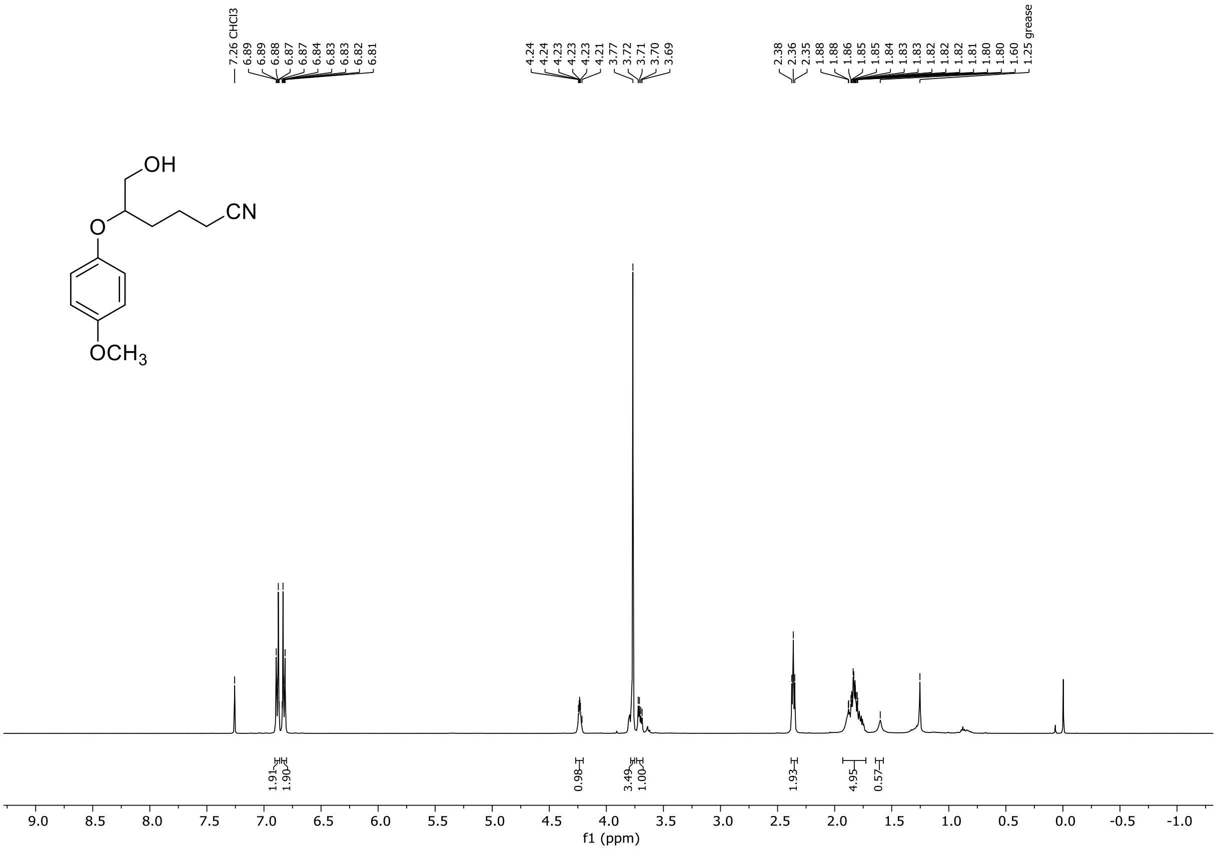

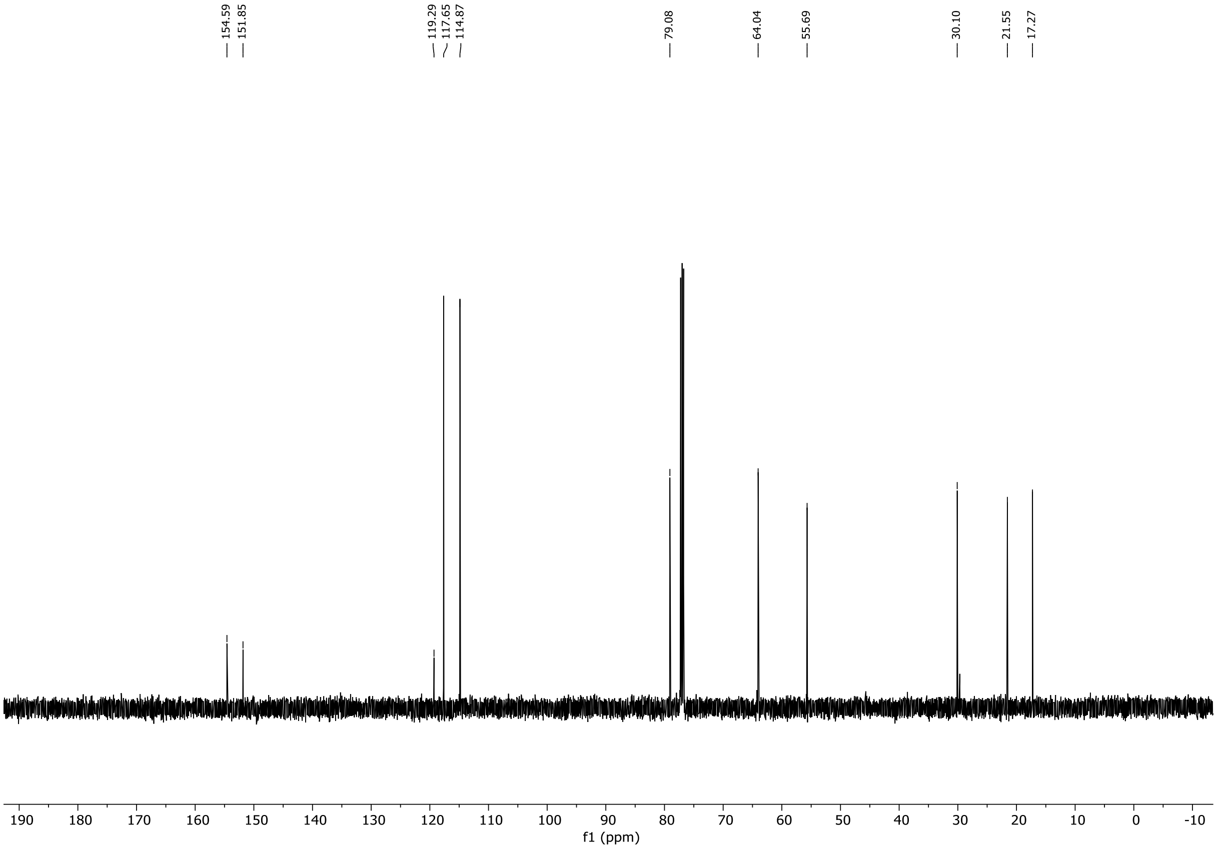

^{1}H NMR (400 MHz, $CDCl_3$) and ^{13}C NMR (125 MHz, $CDCl_3$); 6-(benzyloxy)-5-(hydroxymethyl)hexanenitrile (26)

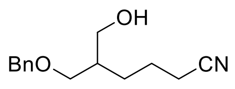

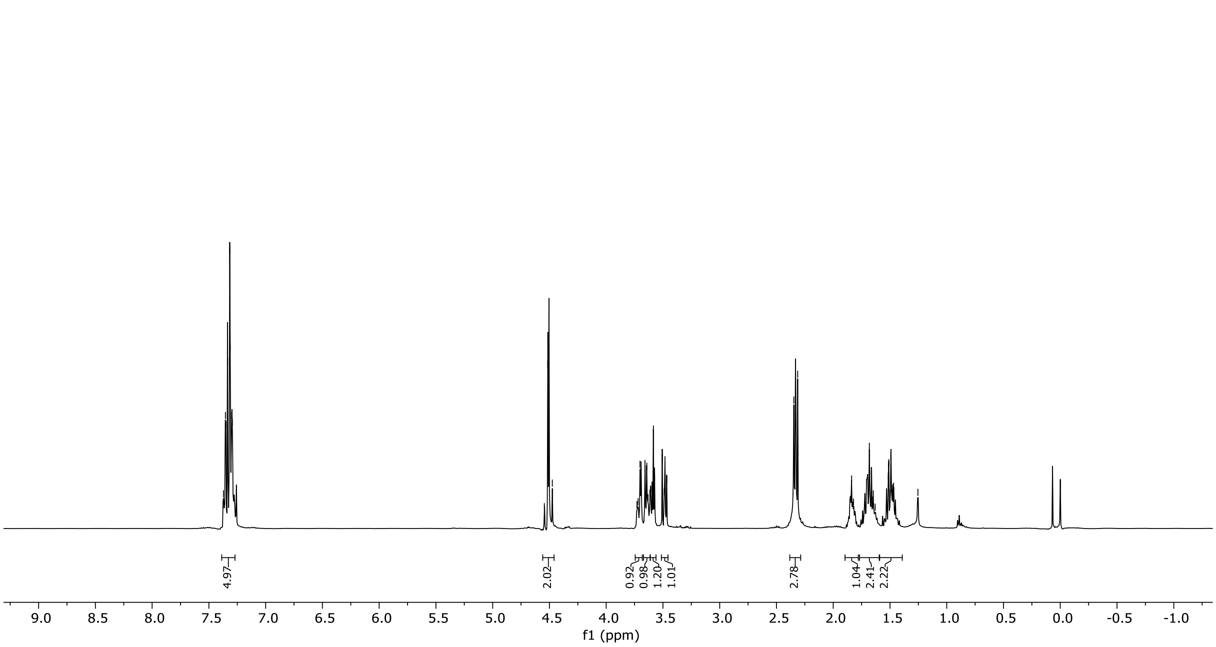

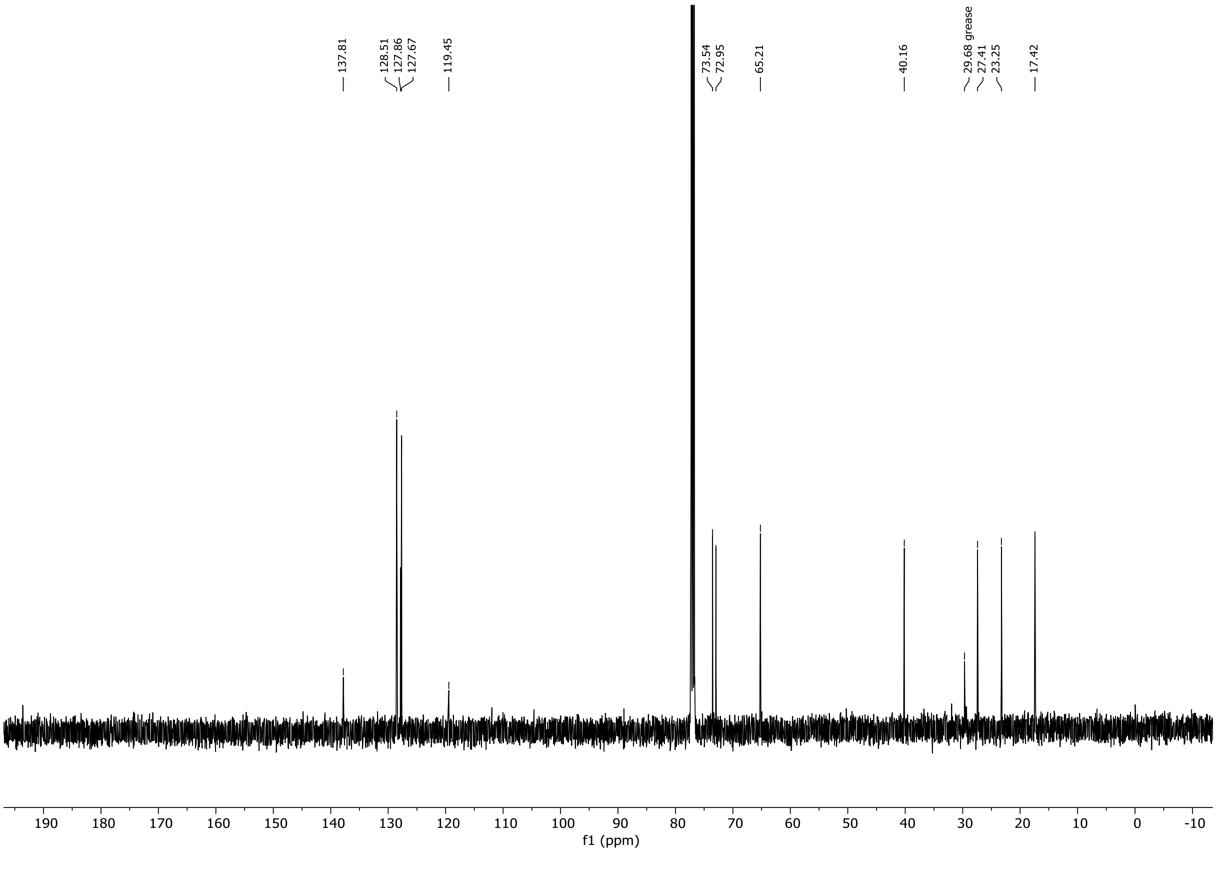

^{1}H NMR (400 MHz, $CDCl_3$) and ^{13}C NMR (100 MHz, $CDCl_3$); 8-(benzyloxy)-6-hydroxyoctanenitrile (28)

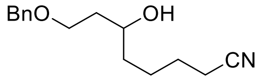

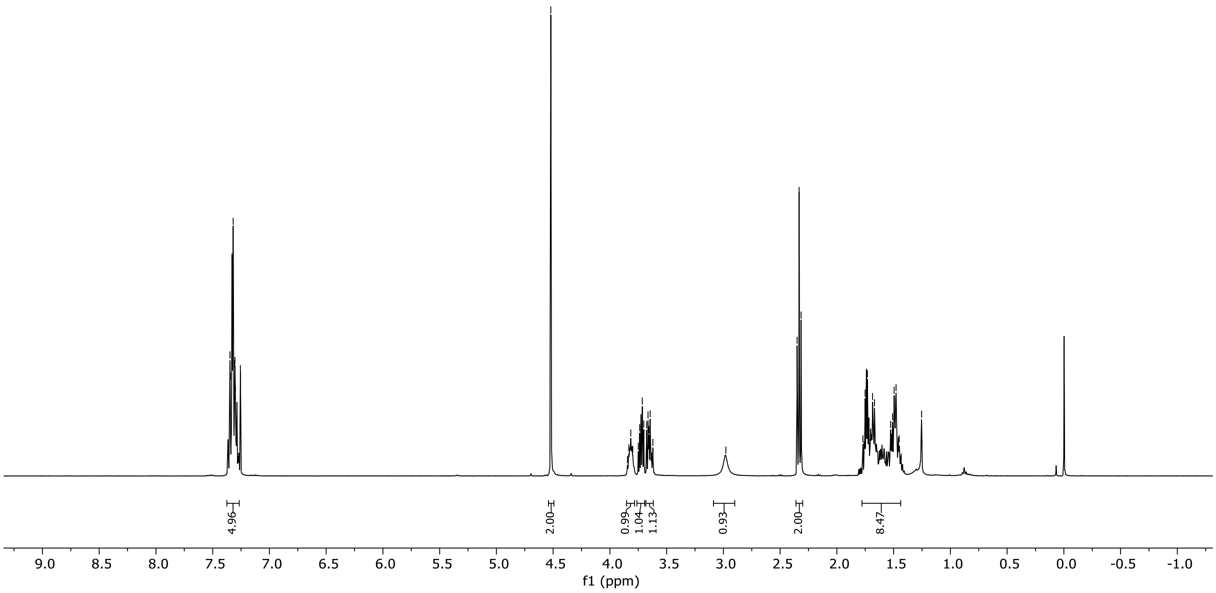

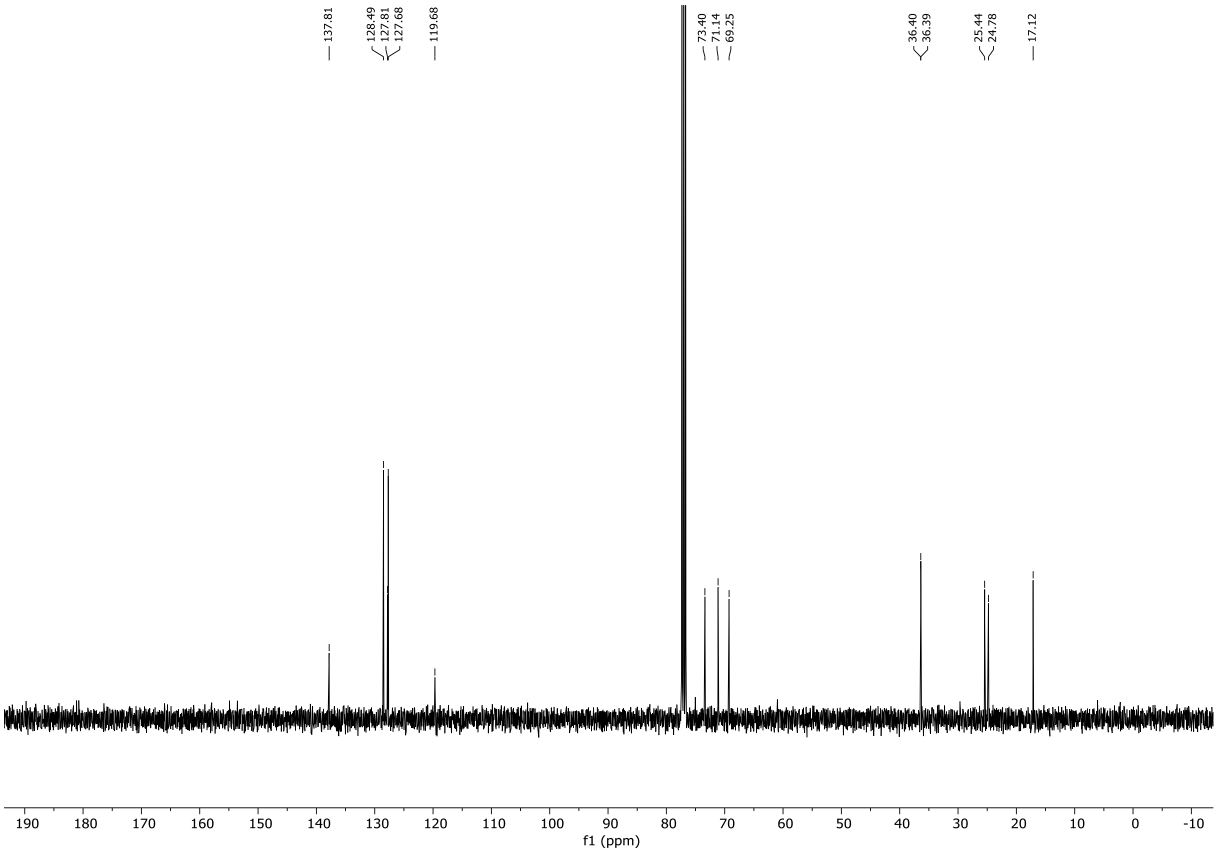

^{1}H NMR (500 MHz, CDCl$_3$) and ^{13}C NMR (125 MHz, CDCl$_3$); 6-hydroxy-8-((triisopropylsilyl)oxy)octanenitrile (29)

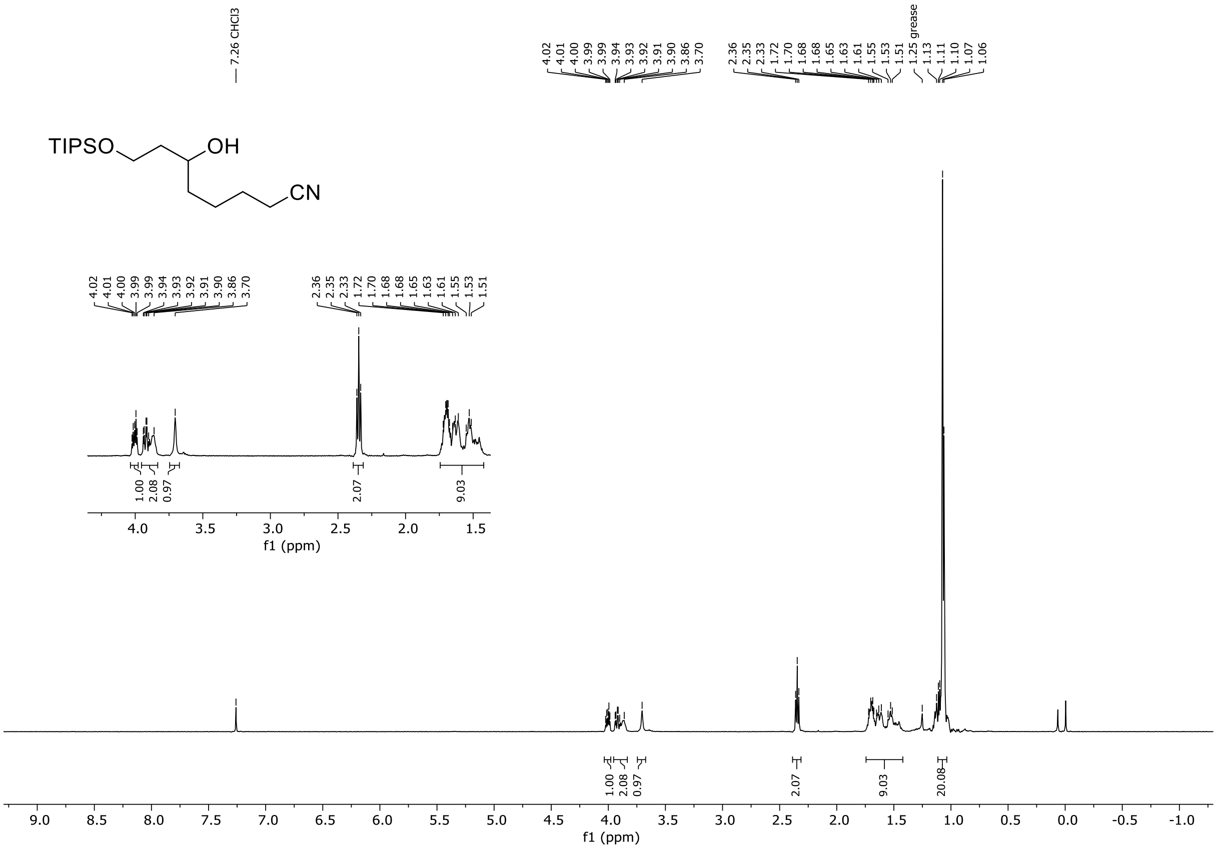

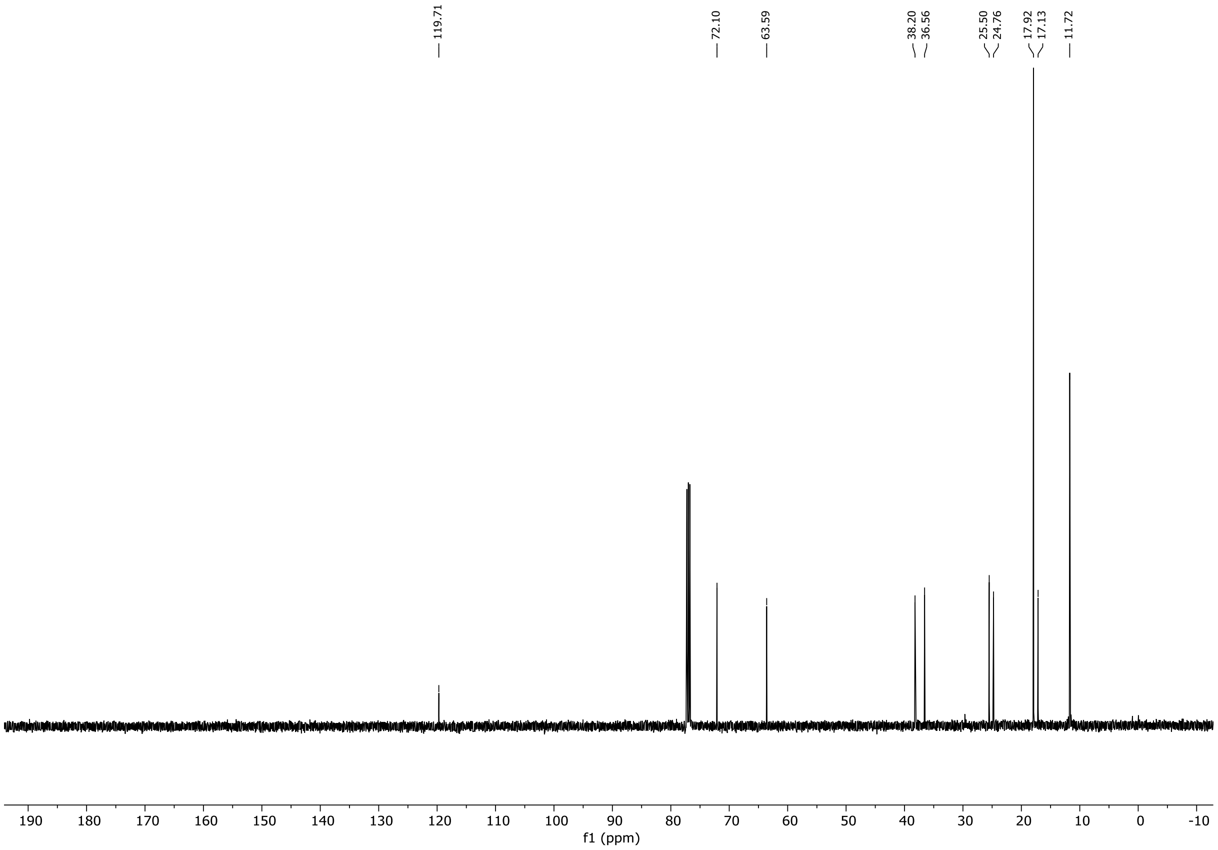

^{1}H NMR (400 MHz, CDCl$_3$) and ^{13}C NMR (100 MHz, CDCl$_3$); methyl 6-hydroxy-5-phenylhexanoate (21)

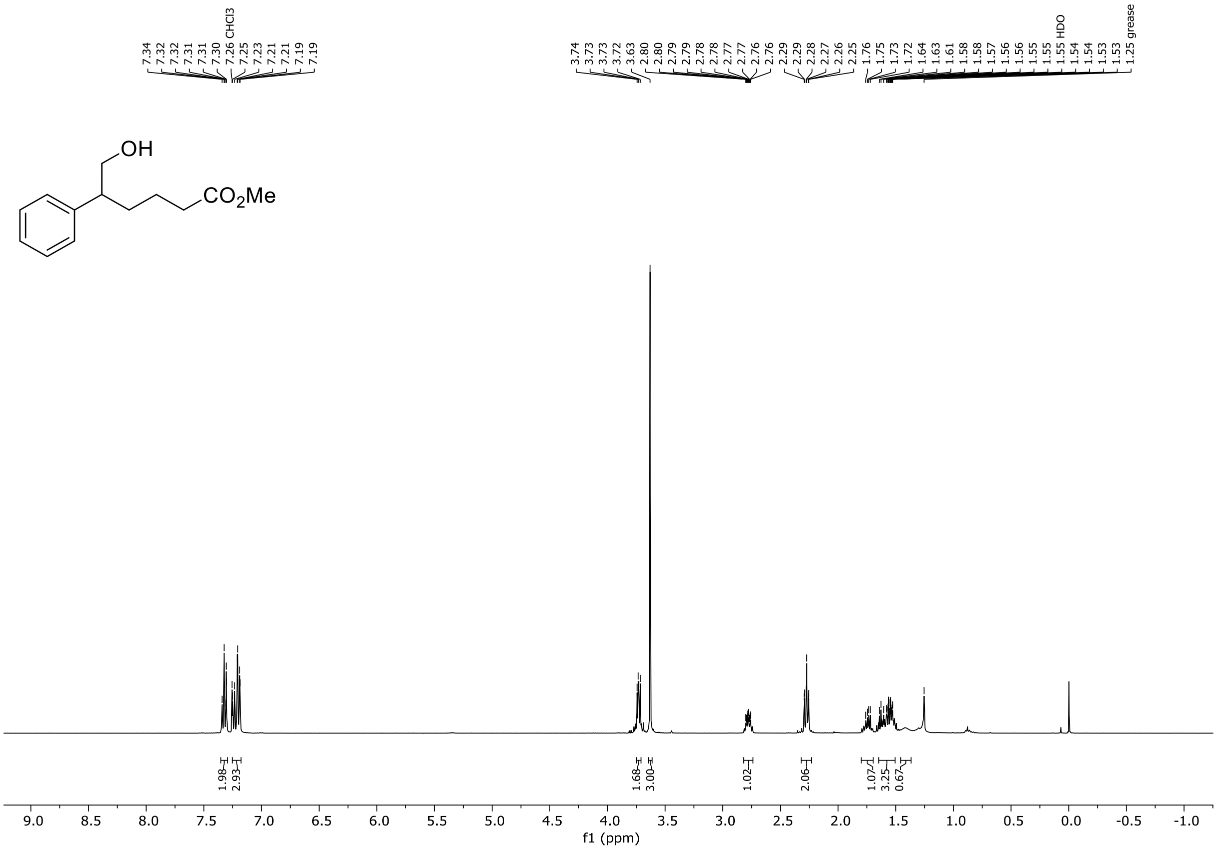

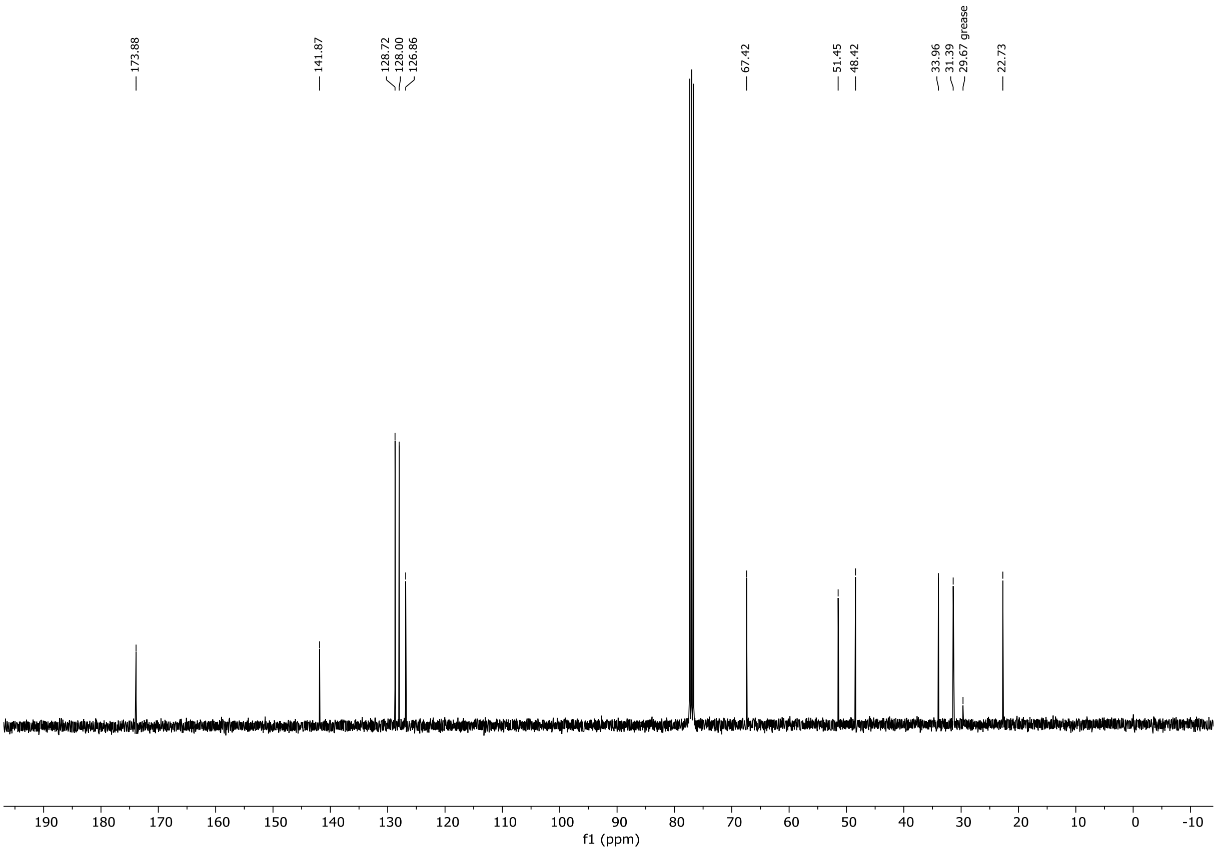

^{1}H NMR (400 MHz, CDCl$_3$) and ^{13}C NMR (125 MHz, CDCl$_3$); benzyl 6-hydroxy-5-phenylhexanoate (30)

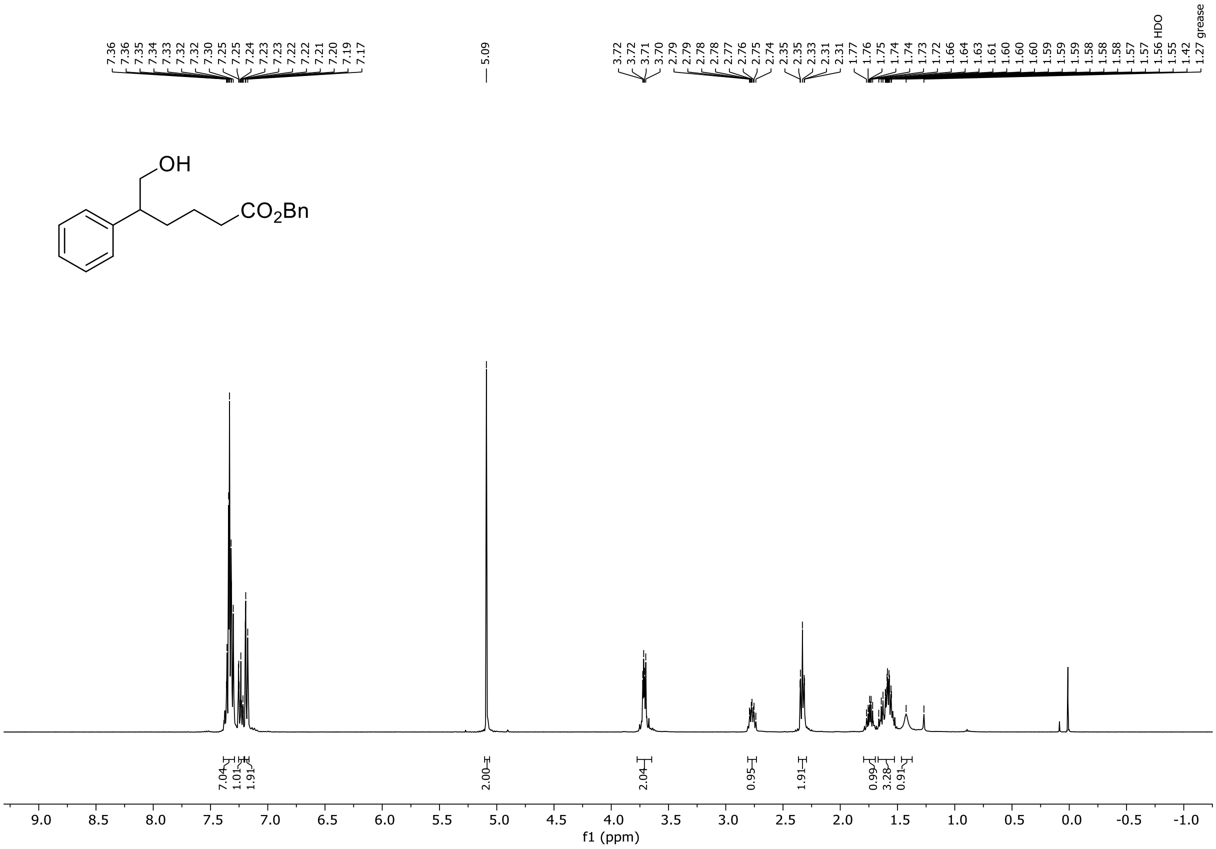

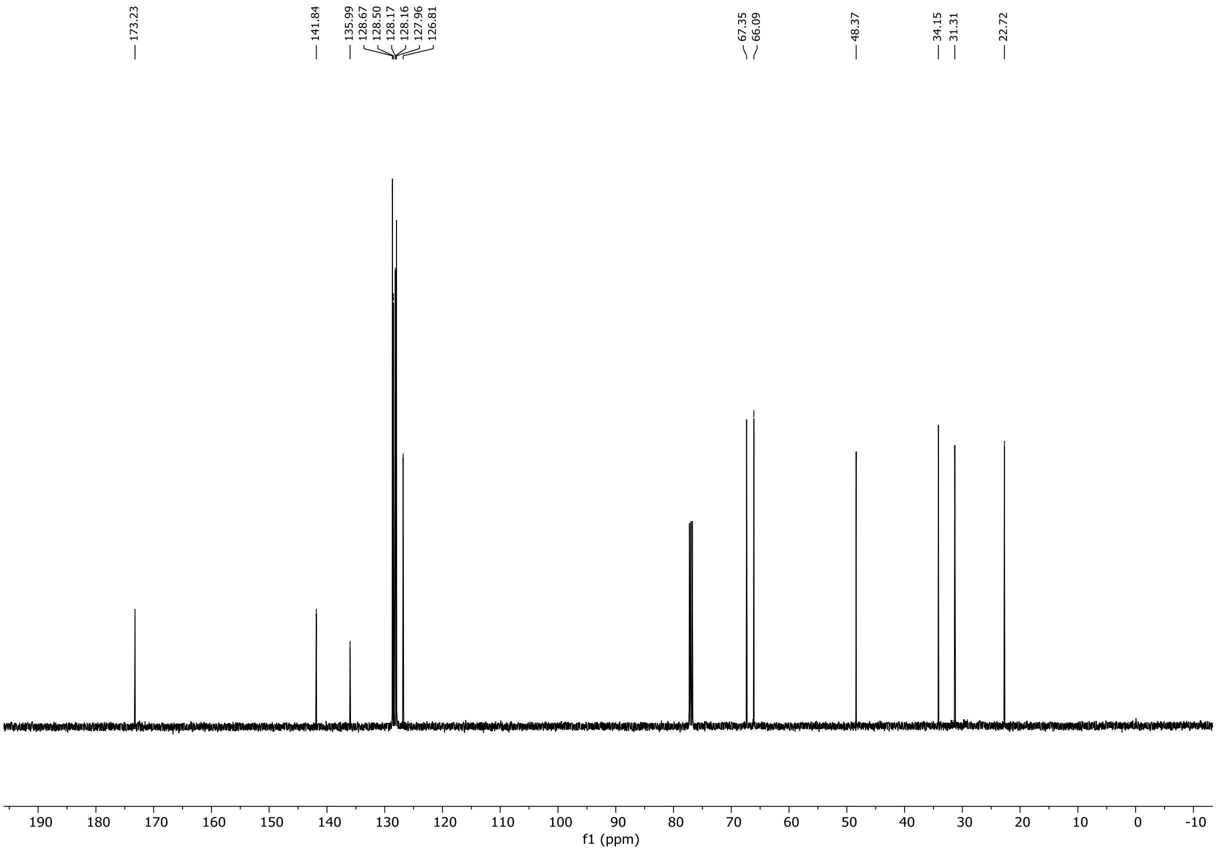

^{1}H NMR (500 MHz, $CDCl_3$) and ^{13}C NMR (125 MHz, $CDCl_3$); 4-methoxyphenyl 6-hydroxy-5-phenylhexanoate (31a)

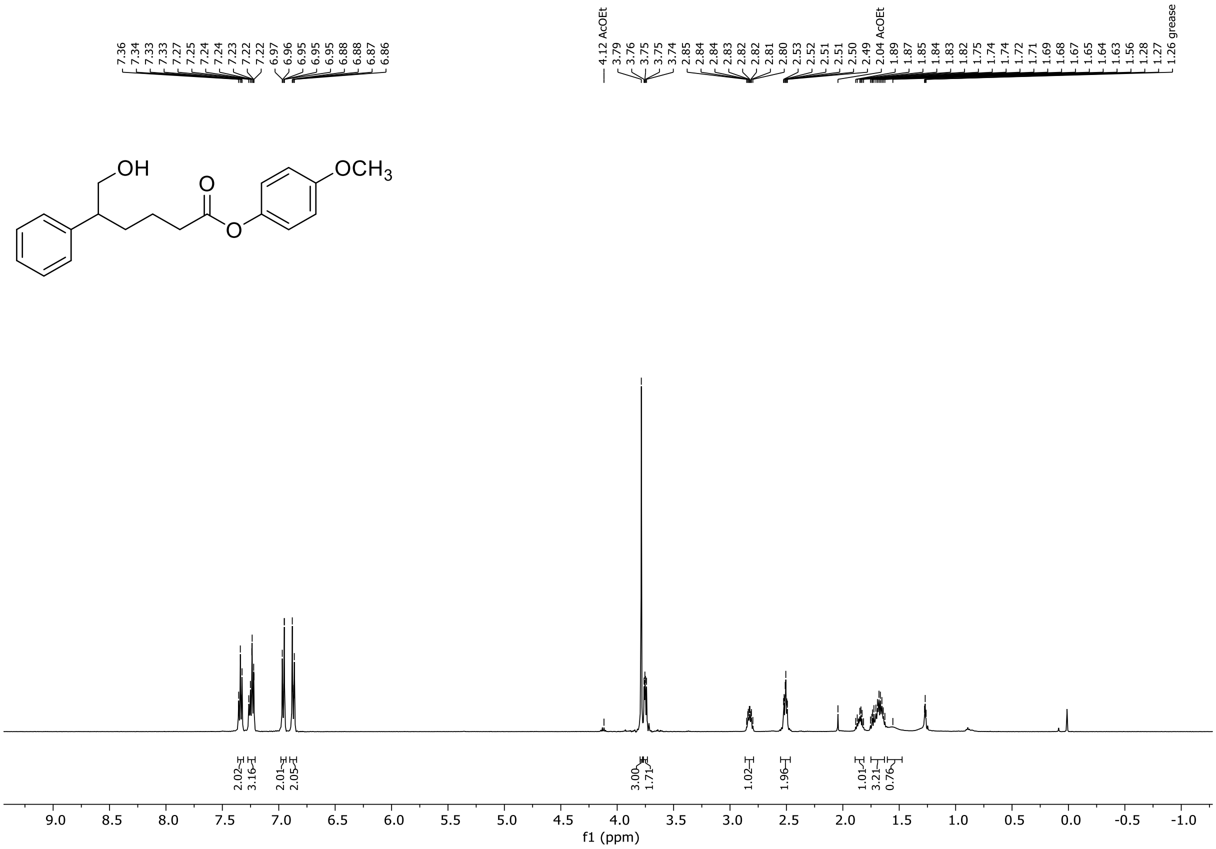

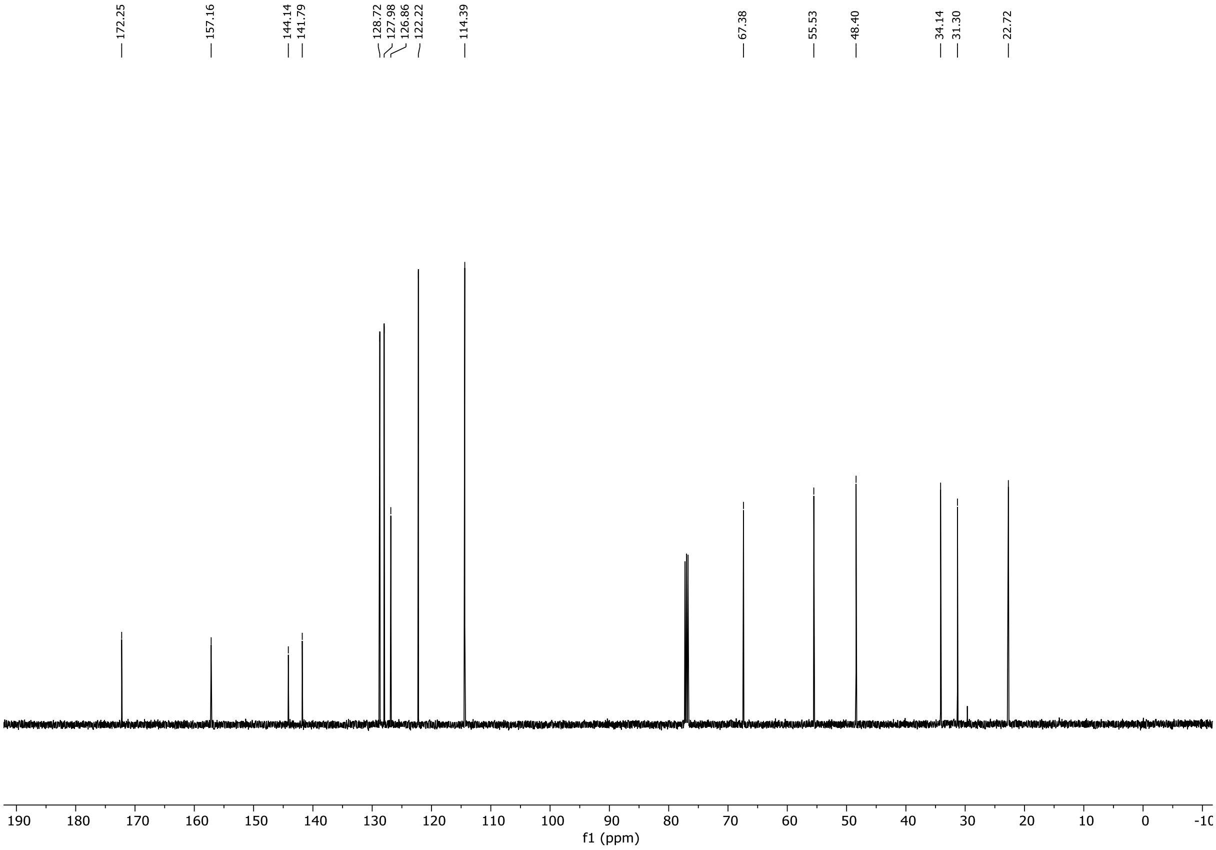

^{1}H NMR (500 MHz, CDCl$_3$) and ^{13}C NMR (125 MHz, CDCl$_3$); 4-cyanophenyl 6-hydroxy-5-phenylhexanoate (31b)

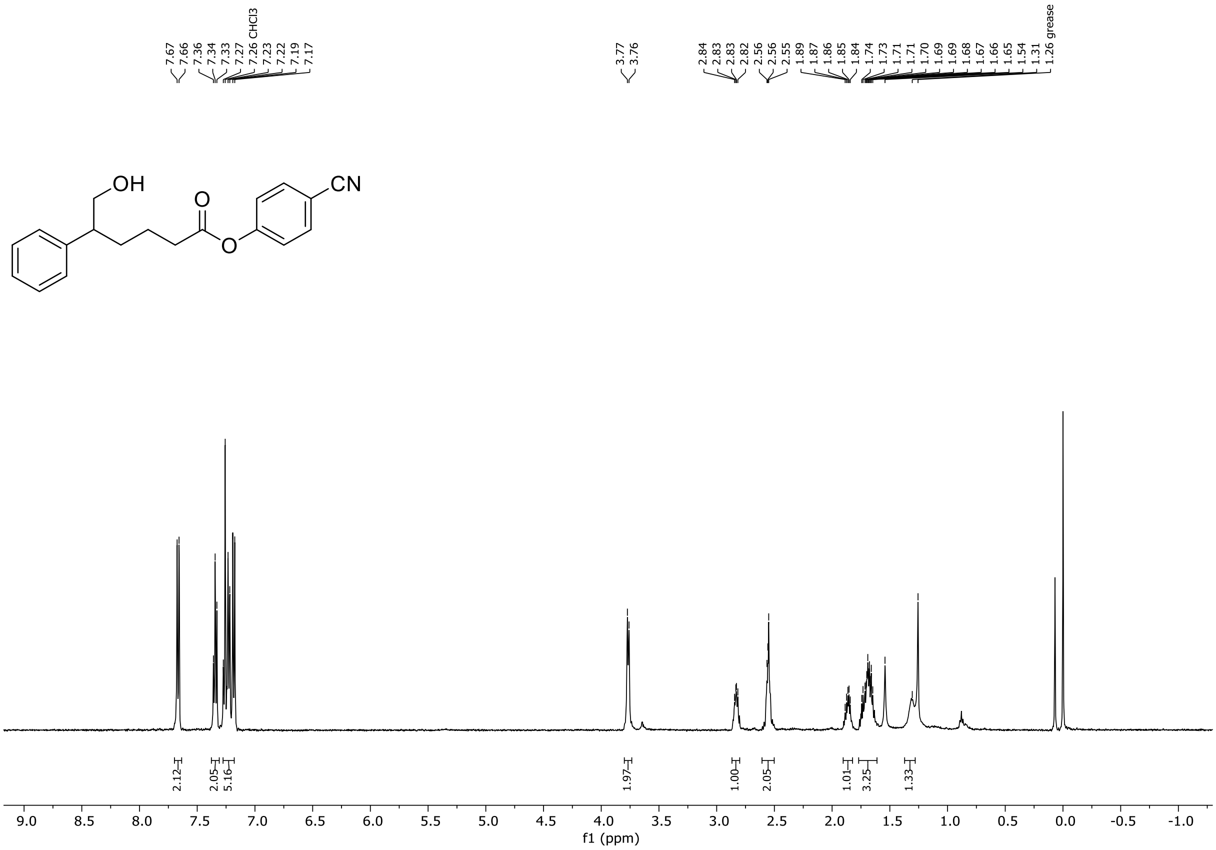

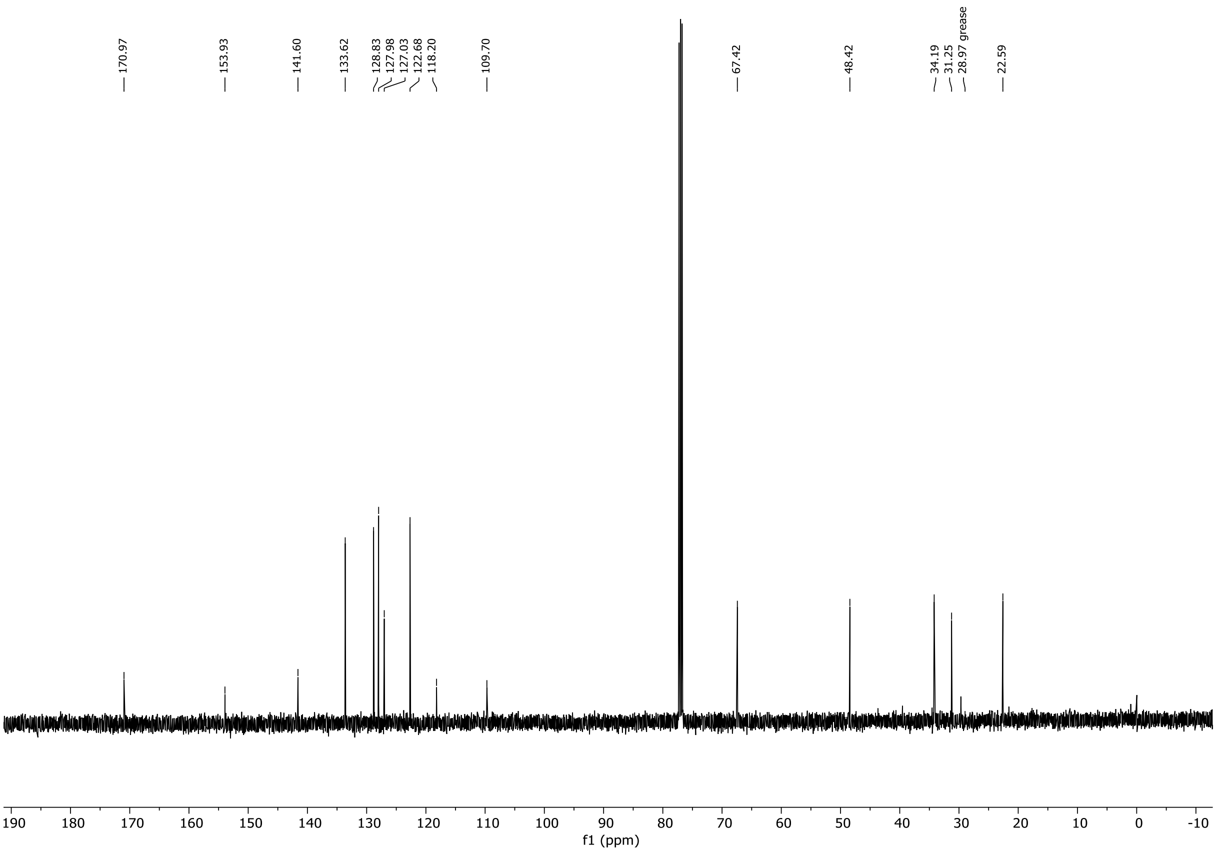

^{1}H NMR (400 MHz, CDCl3) and ^{13}C NMR (125 MHz, CDCl3)pent-4-en-1-yl 6-hydroxy-5-phenylhexanoate (32)

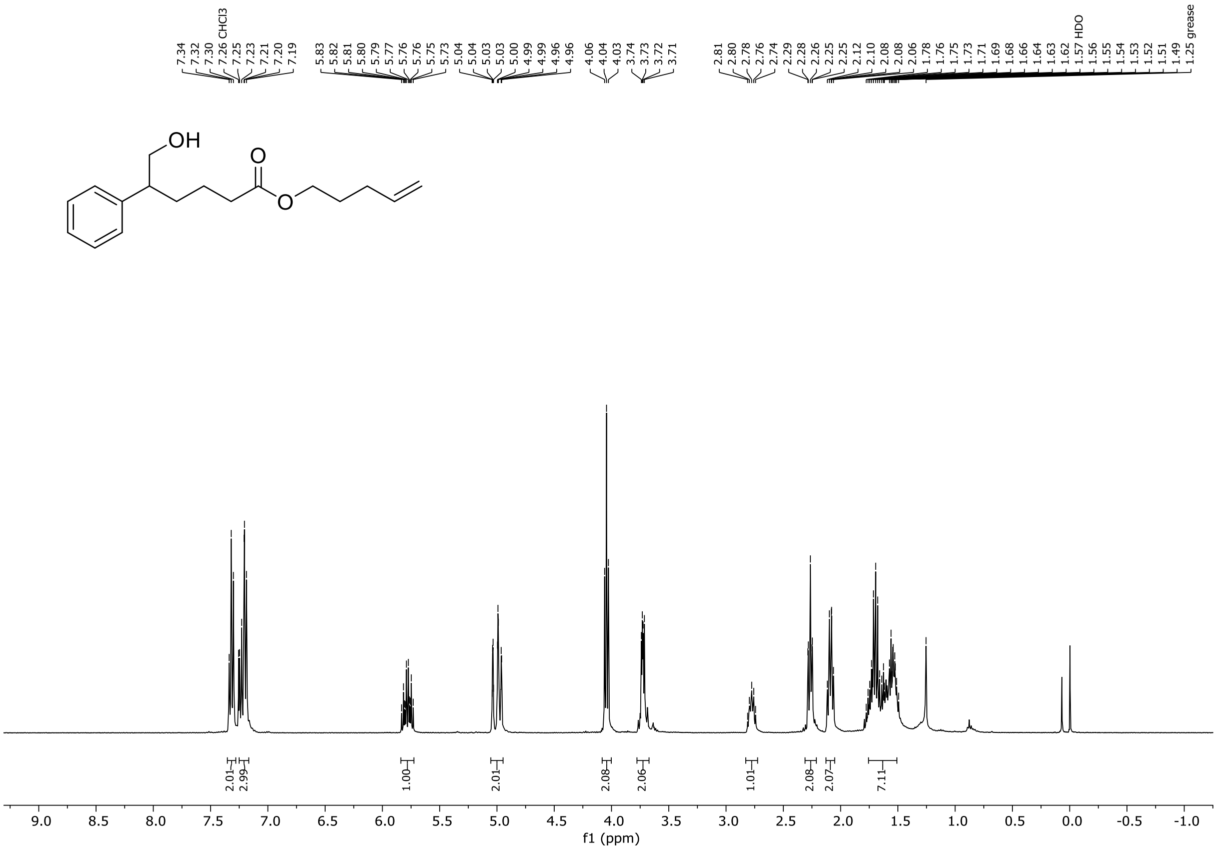

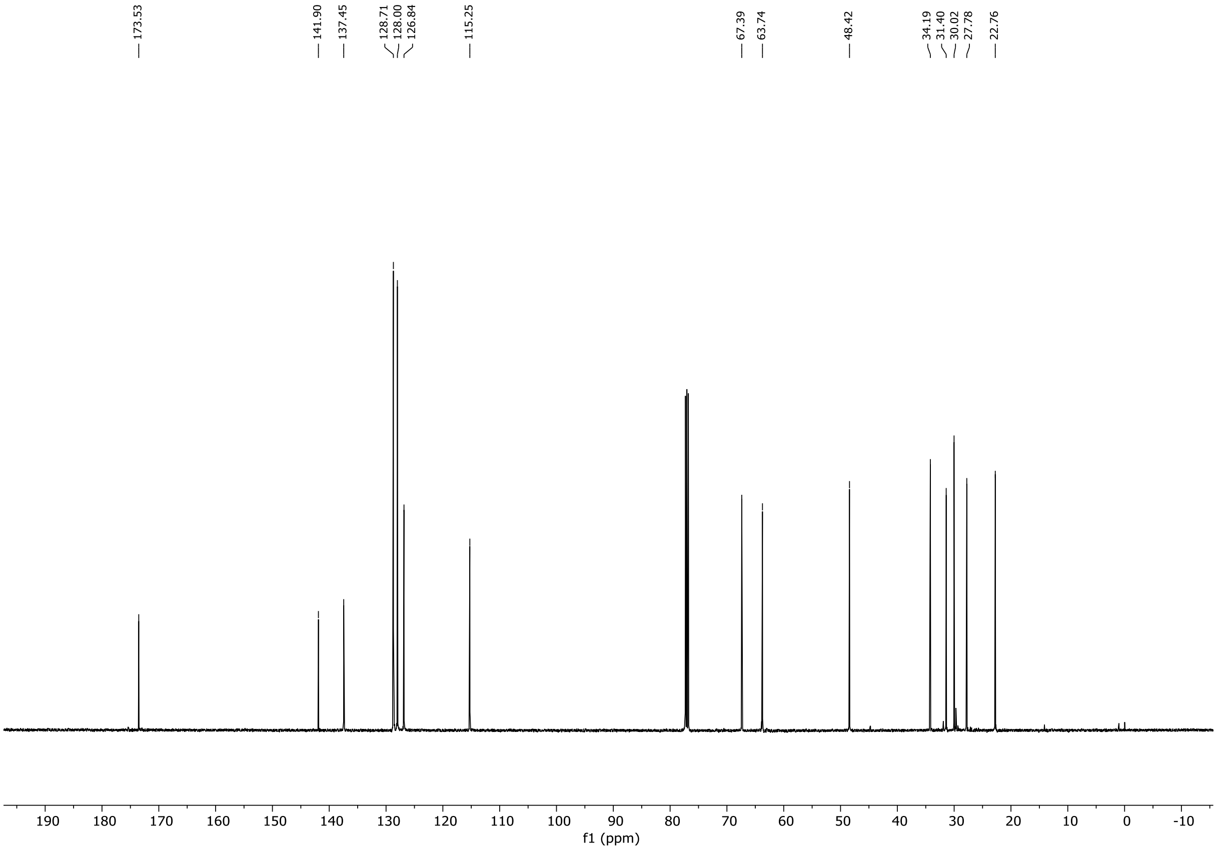

^{1}H NMR (500 MHz, CDCl$_3$) and ^{13}C NMR (125 MHz, CDCl$_3$); 2-phenyl-5-(phenylsulfonyl)pentan-1-ol (33)

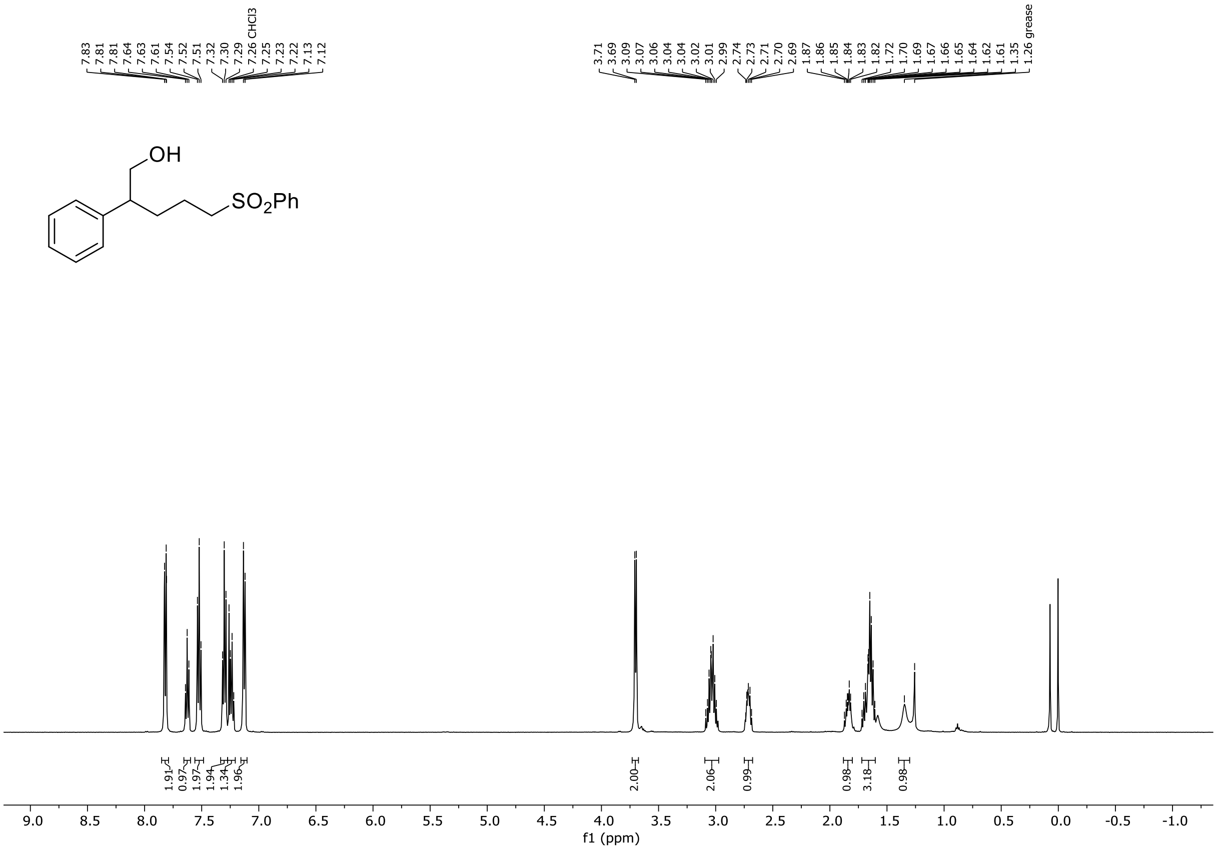

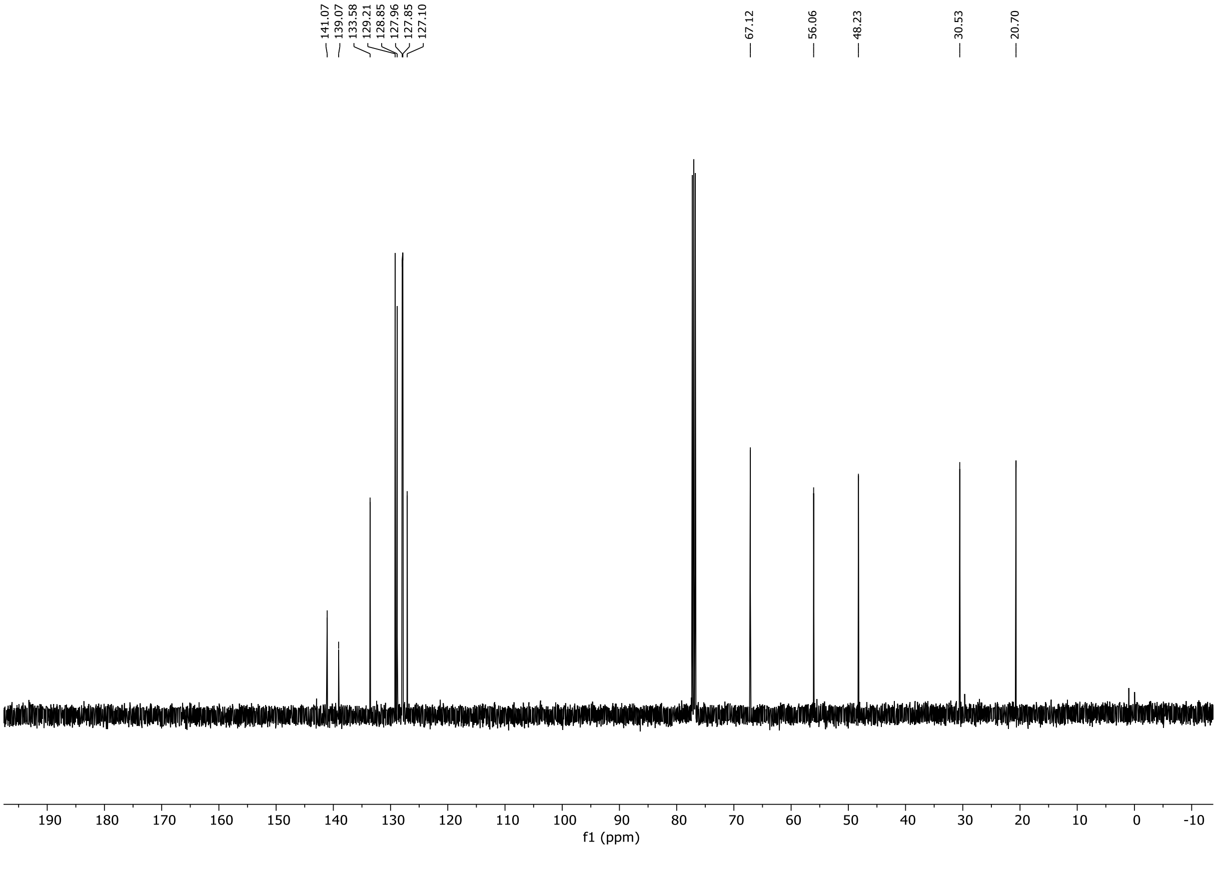

^{1}H NMR (500 MHz, CDCl$_3$) and ^{13}C NMR (125 MHz, CDCl$_3$); methyl 6-hydroxy-2-methyl-5-phenylhexanoate (34)

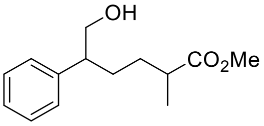

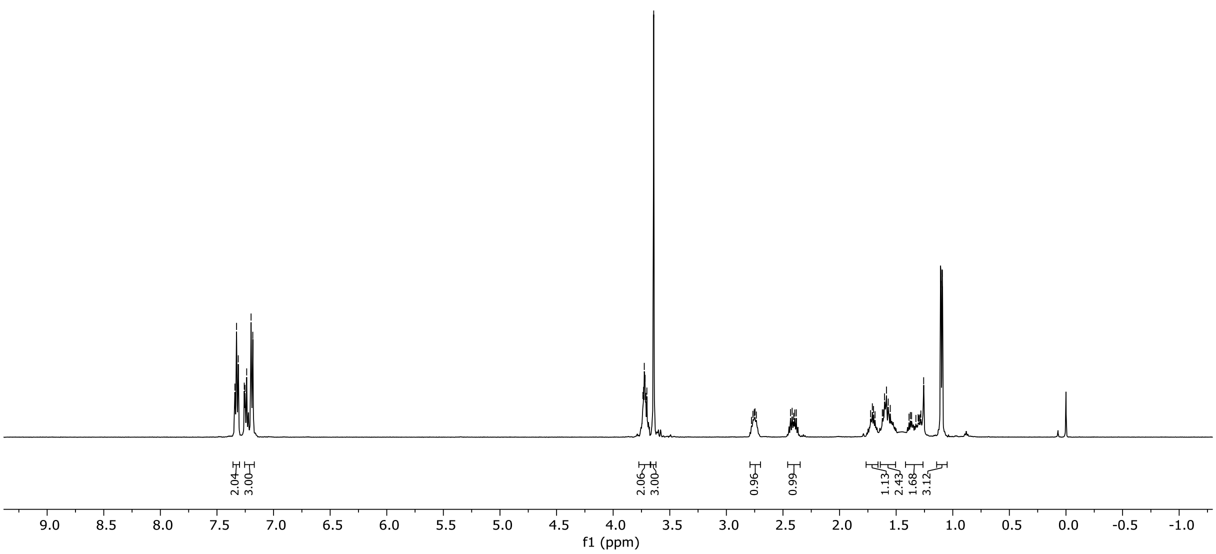

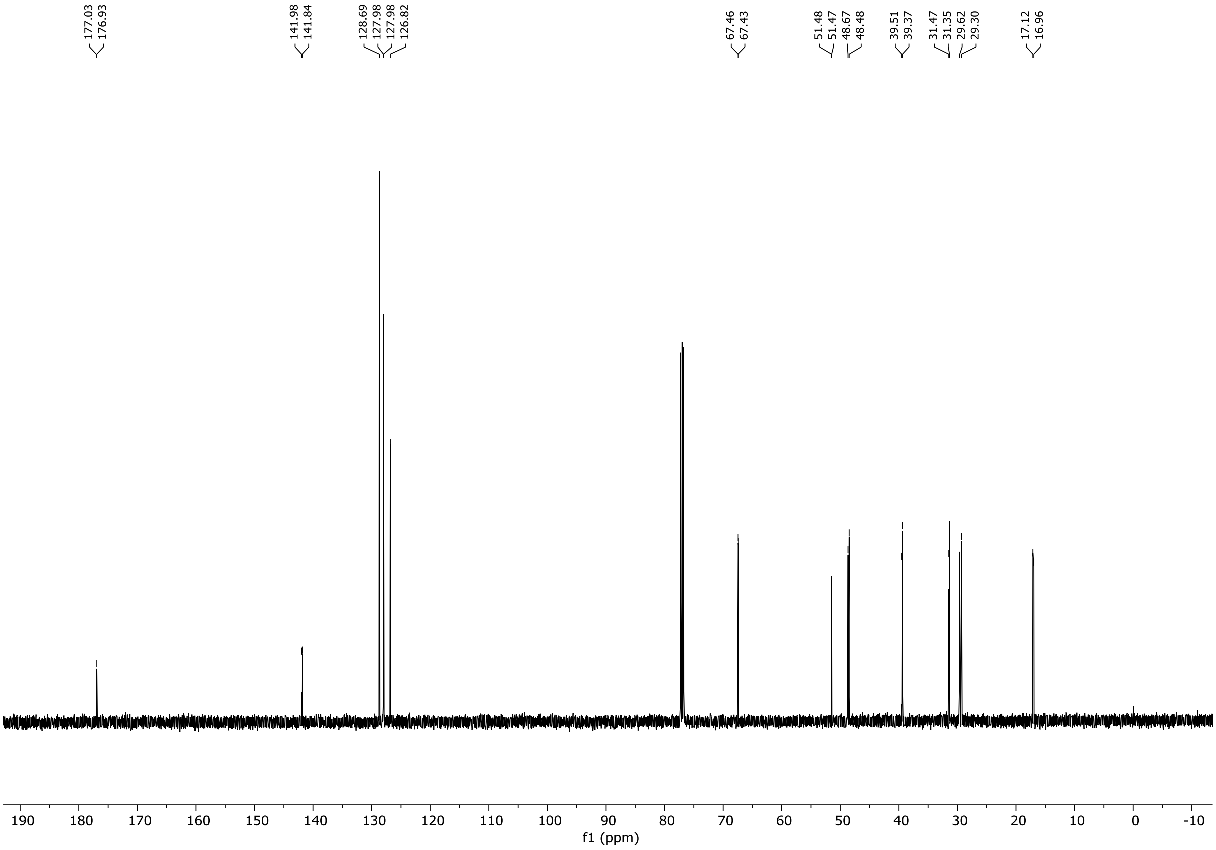

^{1}H NMR (500 MHz, $CDCl_3$) and ^{13}C NMR (125 MHz, $CDCl_3$); methyl 6-hydroxy-2,5-diphenylhexanoate (35)

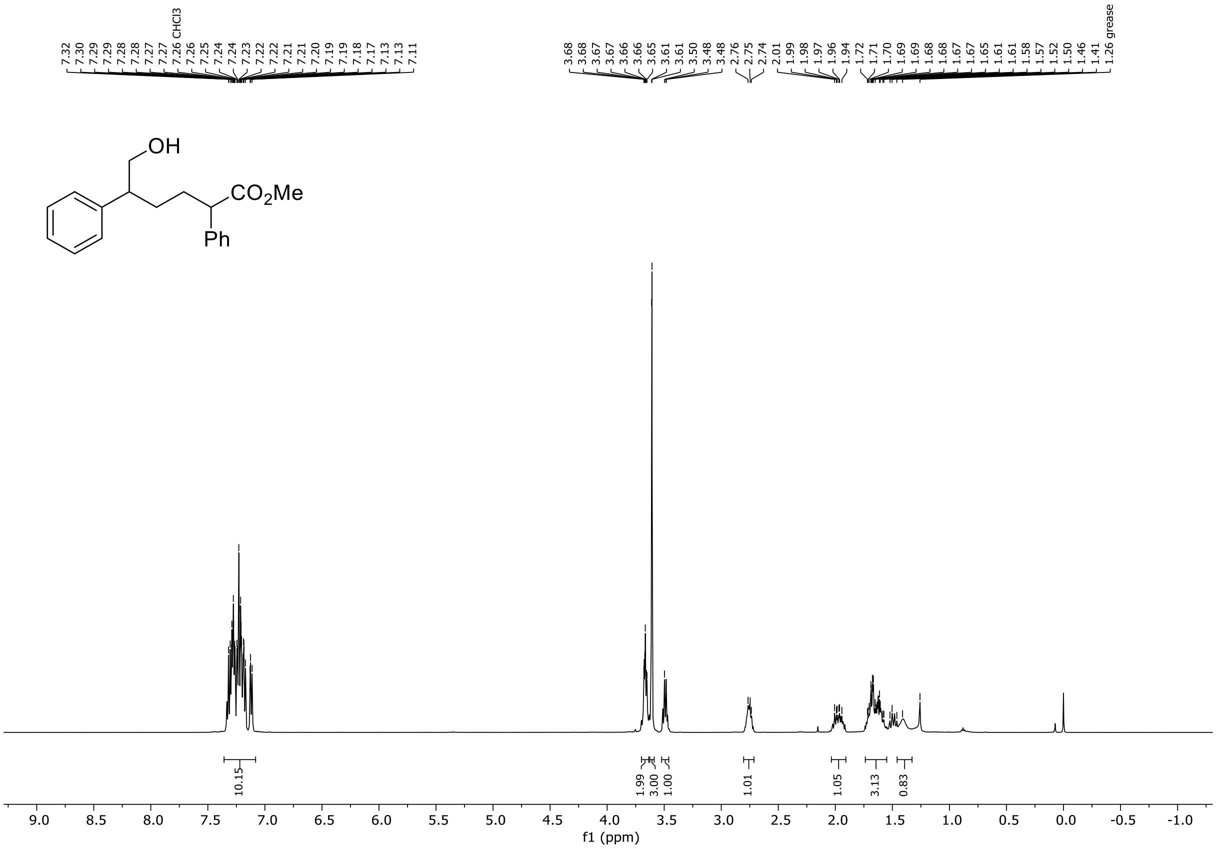

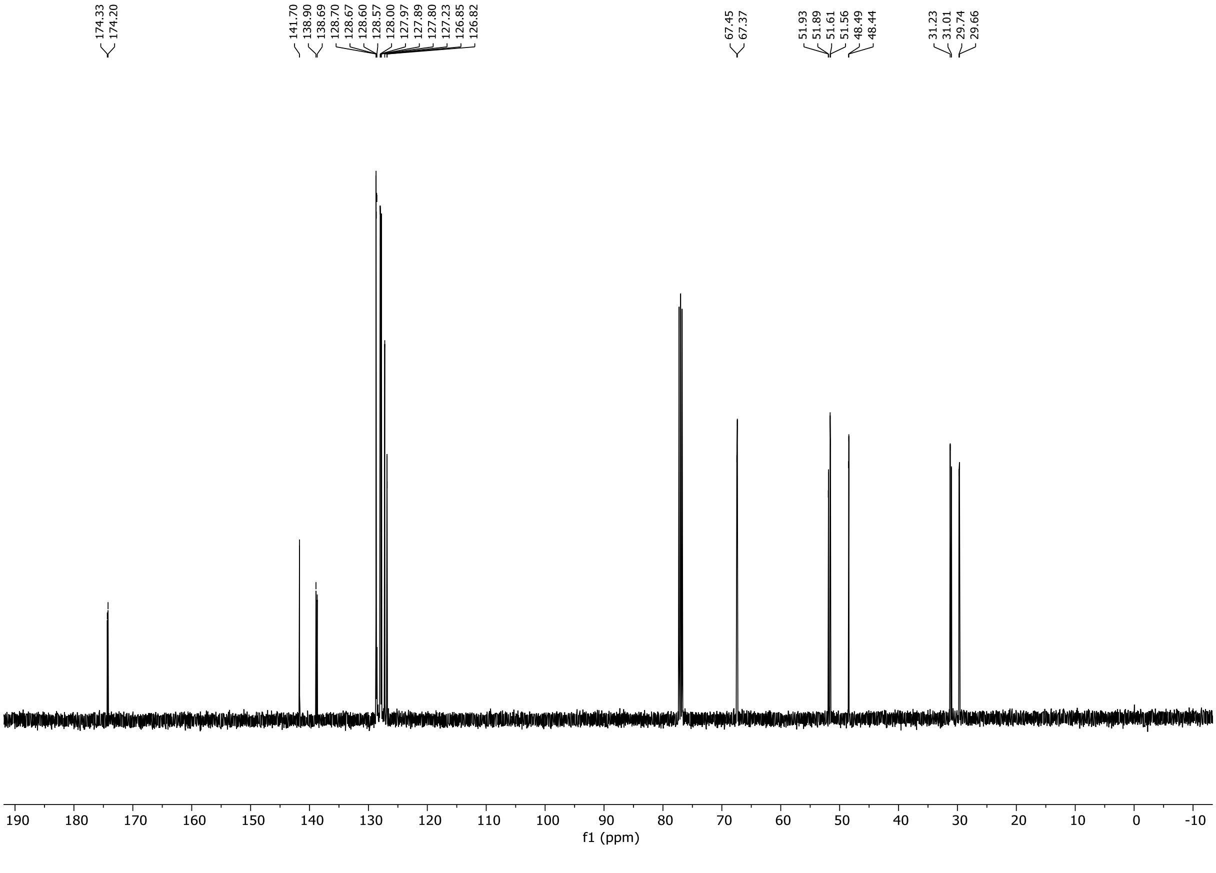

^{1}H NMR (500 MHz, $CDCl_3$) and ^{13}C NMR (125 MHz, $CDCl_3$); pent-4-yn-1-yl 6-hydroxy-5-phenylhexanoate (36)

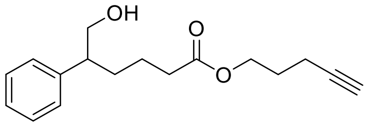

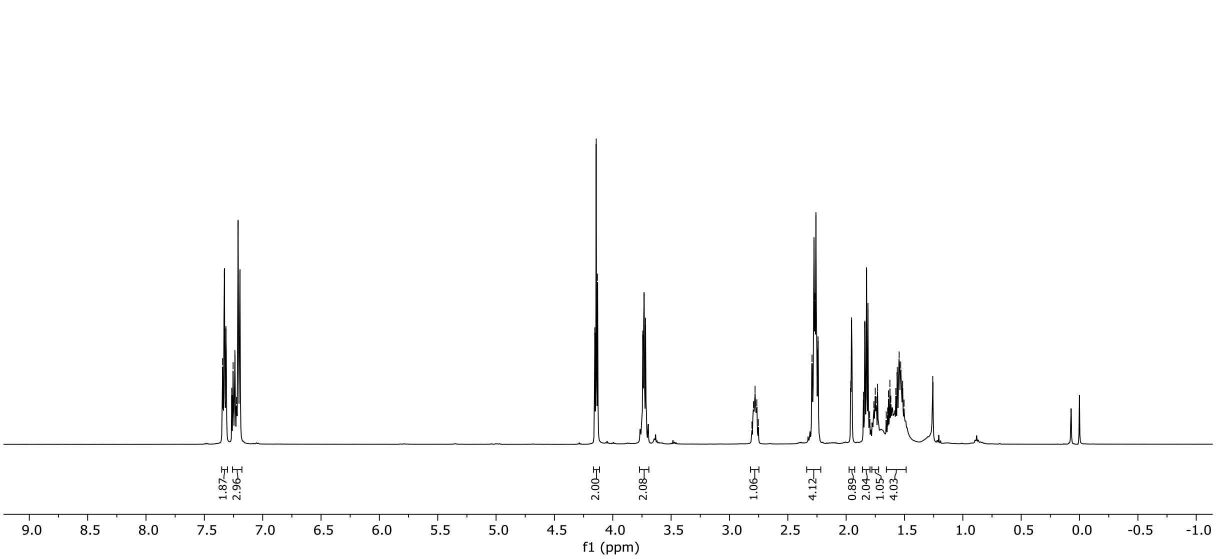

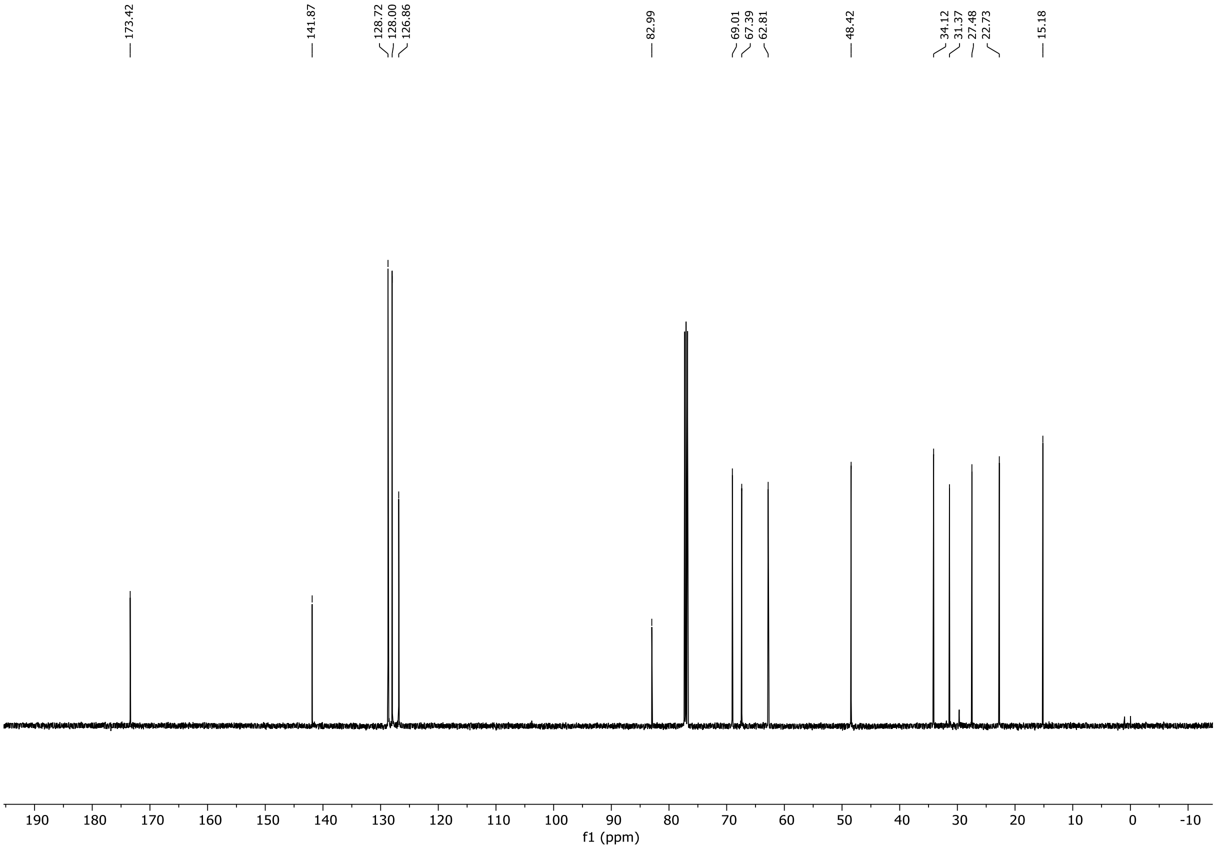

^{1}H NMR (500 MHz, CDCl$_3$) and ^{13}C NMR (125 MHz, CDCl$_3$); (1*R*,2*S*,5*R*)-2-isopropyl-5-methylcyclohexyl 6-hydroxy-5-phenylhexanoate (37)

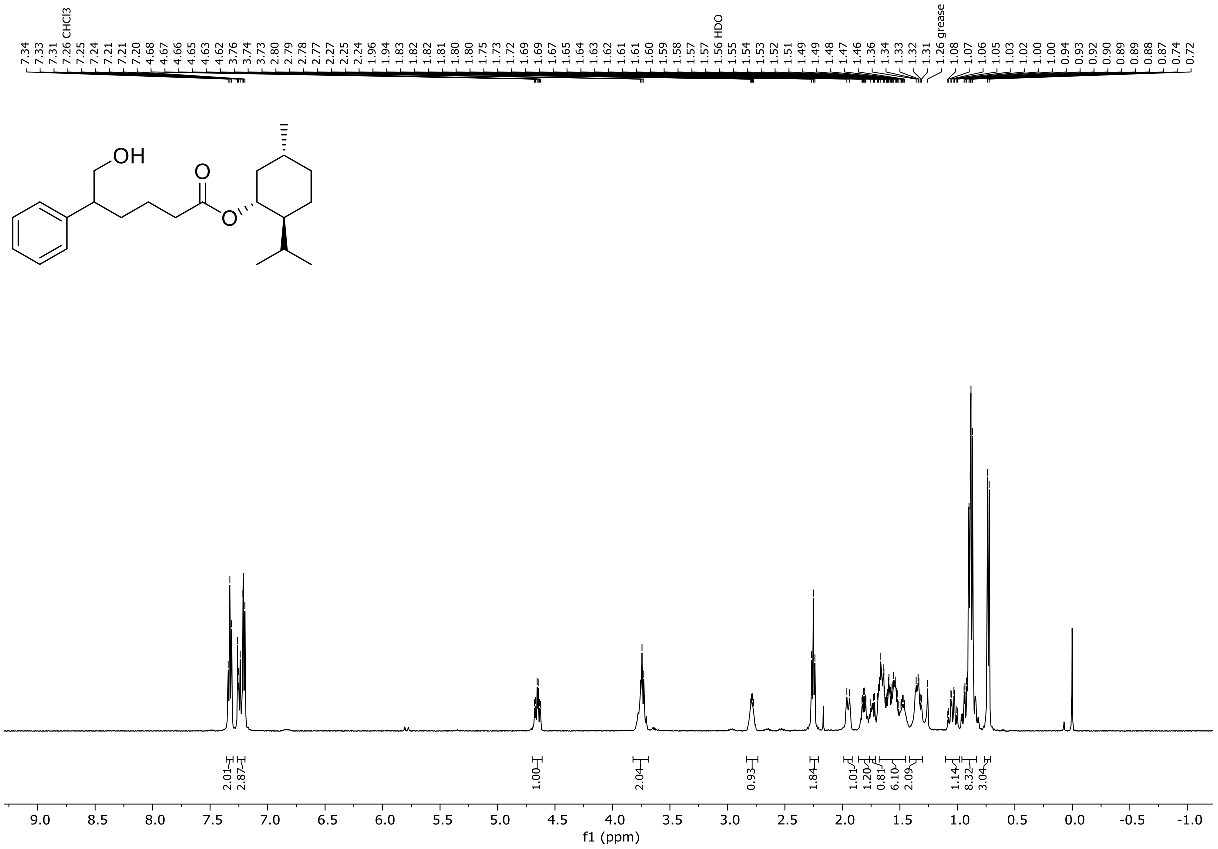

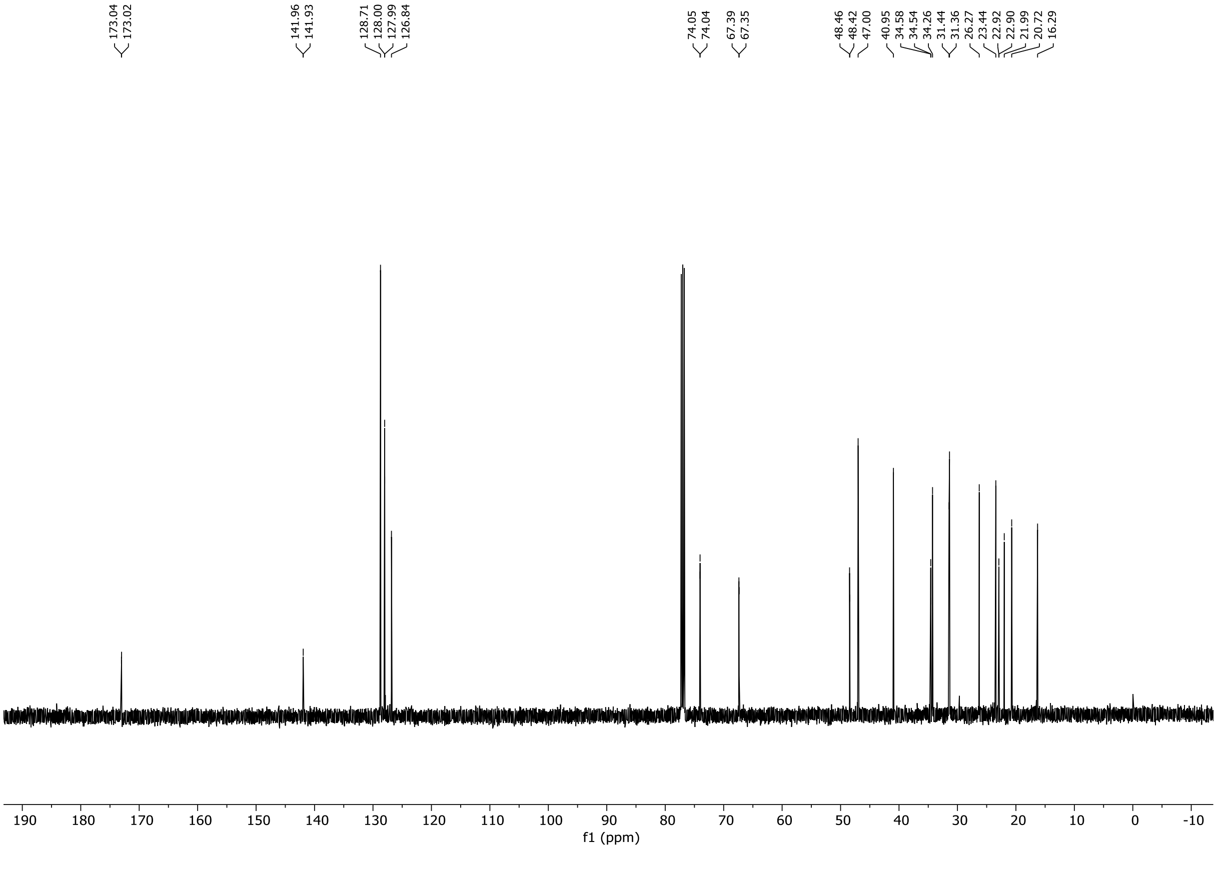

^{1}H NMR (500 MHz, $CDCl_3$) and ^{13}C NMR (125 MHz, $CDCl_3$); (8*R*,9*S*,13*S*,14*S*)-13-methyl-17-oxo-7,8,9,11,12,13,14,15,16,17-decahydro-6*H*-cyclopenta[a]phenanthren-3-yl 6-hydroxy-5-phenylhexanoate (38)

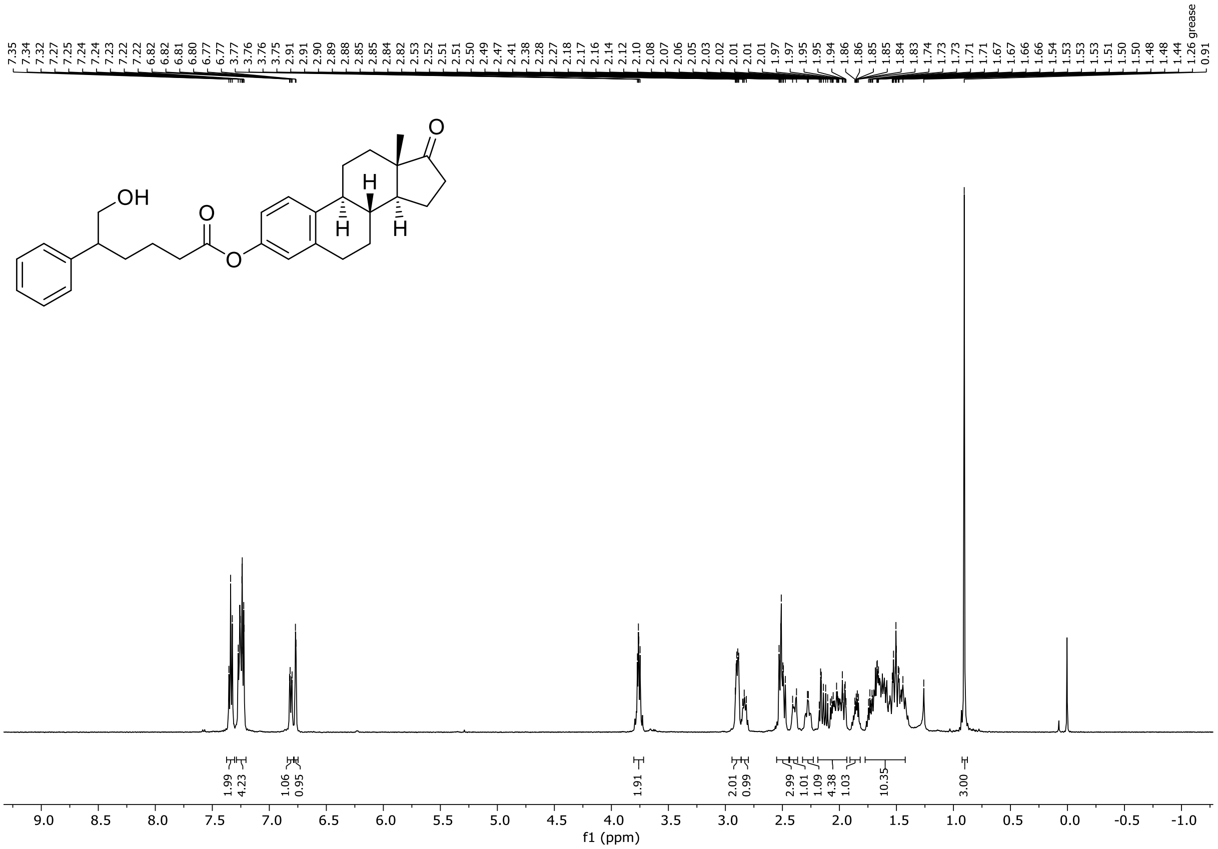

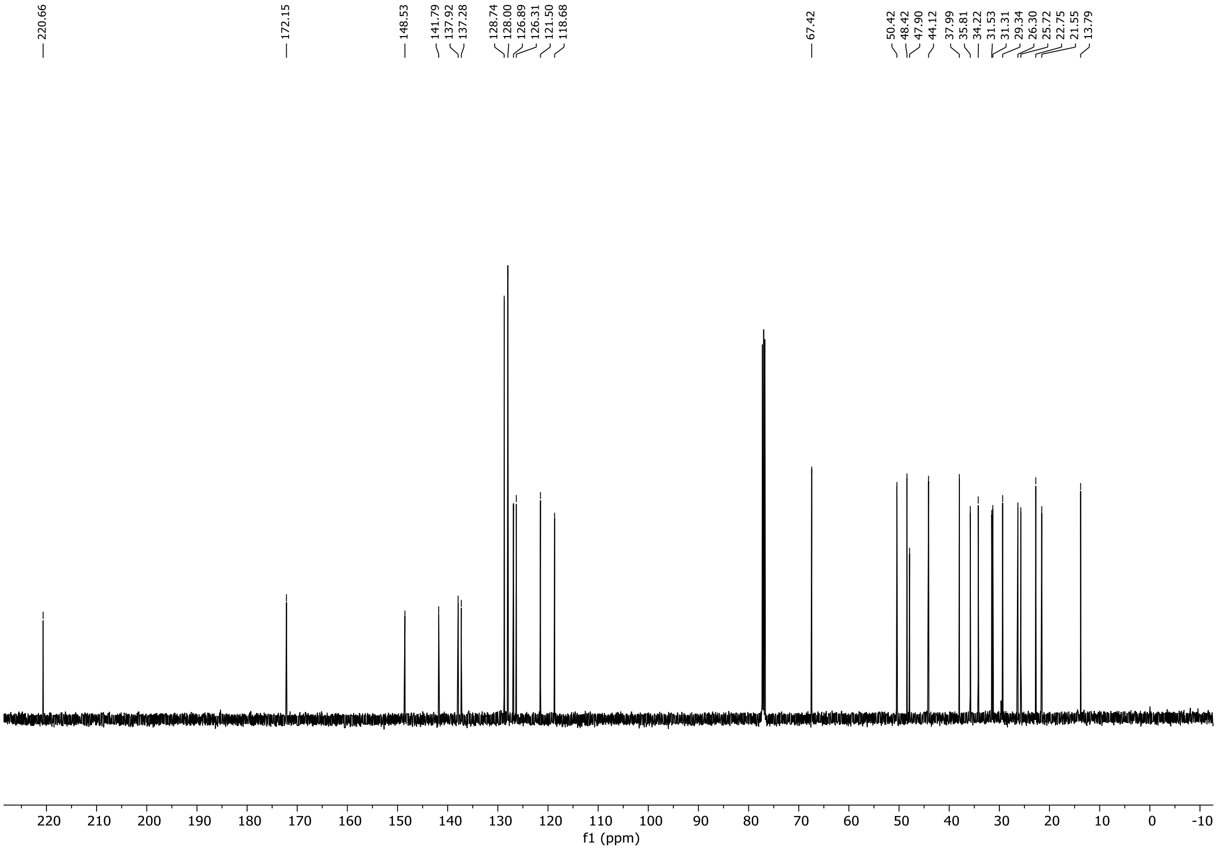

Supplement: Supplementary file 1 — ol2c00355_si_001.pdf [file ol2c00355_si_001.pdf]
